# Exposome-wide ranking of modifiable risk factors for cardiometabolic disease traits

**DOI:** 10.1038/s41598-022-08050-1

**Published:** 2022-03-08

**Authors:** Alaitz Poveda, Hugo Pomares-Millan, Yan Chen, Azra Kurbasic, Chirag J. Patel, Frida Renström, Göran Hallmans, Ingegerd Johansson, Paul W. Franks

**Affiliations:** 1 Department of Clinical Sciences, Genetic and Molecular Epidemiology Unit, Lund University, 214 28 Malmö, Sweden; 2 Cardiovascular Medicine Unit, Department of Medicine Solna, Karolinska Institutet, Stockholm, Sweden; 3 Department of Medical Epidemiology and Biostatistics, Karolinska Institutet, Stockholm, Sweden; 4 Department of Biomedical Informatics, Harvard Medical School, Boston, MA USA; 5 Department of Public Health and Clinical Medicine, Umeå University, Umeå, Sweden; 6 Division of Endocrinology and Diabetes, Cantonal Hospital St. Gallen, St. Gallen, Switzerland; 7 Department of Odontology, Umeå University, Umeå, Sweden; 8 Department of Nutrition, Harvard T.H. Chan School of Public Health, Boston, MA USA

**Keywords:** Hypertension, Obesity, Diabetes, Dyslipidaemias, Obesity, Lifestyle modification, Nutrition, Risk factors, Epidemiology

## Abstract

The present study assessed the temporal associations of ~ 300 lifestyle exposures with nine cardiometabolic traits  to identify exposures/exposure groups that might inform lifestyle interventions for the reduction of cardiometabolic disease risk. The analyses were undertaken in a longitudinal sample comprising > 31,000 adults living in northern Sweden. Linear mixed models were used to assess the average associations of lifestyle exposures and linear regression models were used to test associations with 10-year change in the cardiometabolic traits. ‘Physical activity’ and ‘General Health’ were the exposure categories containing the highest number of ‘tentative signals’ in analyses assessing the average association of lifestyle variables, while ‘Tobacco use’ was the top category for the 10-year change association analyses. Eleven modifiable variables showed a consistent average association among the majority of cardiometabolic traits. These variables belonged to the domains: (i) Smoking, (ii) Beverage (filtered coffee), (iii) physical activity, (iv) alcohol intake, and (v) specific variables related to Nordic lifestyle (hunting/fishing during leisure time and boiled coffee consumption). We used an agnostic, data-driven approach to assess a wide range of established and novel risk factors for cardiometabolic disease. Our findings highlight key variables, along with their respective effect estimates, that might be prioritised for subsequent prediction models and lifestyle interventions.

## Introduction

The majority of non-communicable diseases are caused by the complex interplay of genetic and environmental factors. In the last decades, major progress has been made in discovering genetic loci predisposing to these diseases, facilitated by genome-wide association studies (GWAS). These studies allow high-throughput and systematic screening of millions of variants against quantitative traits or hard disease endpoints. Unlike population genetics, there are no standard environment ‘chips’ that capture multiple environment exposures simultaneously. Therefore, environmental epidemiology typically involves approaches where hypothesized associations between specific environmental exposures and disease traits are separately tested. These studies are limited by the expectations and knowledge about the hypothesized relationships they seek to test, which may cause bias and inhibit discovery^1^.

Environment-wide association studies (EWAS) represent an approach through which multiple environmental factors can be systematically screened for their associations with disease traits in a manner that is to a large degree agnostic to prior knowledge about disease associations; in this sense, the EWAS approach is similar to GWAS. EWAS was first described in the published literature in a 2010 paper reporting associations analyses between metabolites and type 2 diabetes^2^. Later, EWAS was used to identify nutrients, environmental contaminants, and prescribed drugs^3–9^ associated with disease and disease complications. Almost all published EWAS have used cross-sectional epidemiological data to assess exposures at a fixed time point without consideration of the impact of exposures throughout an individual's lifetime. Longitudinal data analyses may help us understand the associations among exposures and changes in cardiometabolic traits over time.

The present study sought to assess the temporal relationships of more than 300 lifestyle exposures (e.g. food items, sleep habits, physical activity, psychosocial factors) with nine cardiometabolic traits (i.e. BMI, blood lipids, blood glucose, and blood pressure) and use these results to identify target lifestyle exposures/exposure groups that could inform lifestyle interventions focused on controlling cardiometabolic diseases.

## Methods

### Participants

The analyses reported here were undertaken using data from the Västerbotten Health Survey (Västerbottens hälsoundersökning; VHU)^10^. VHU is a prospective, population-based cohort study originally designed as a long-term project intended for health promotion among the general population in Västerbotten county (approx. 254,000 inhabitants), northern Sweden. Since 1985, adults residing in Västerbotten have been invited to undergo a clinical examination and complete lifestyle questionnaires during the years of their 30th, 40th, 50th, and 60th birthdays.

A sub-cohort of VHU (*n* = 88,614) was used in the present analyses. Participants with non-Swedish origin (*n* = 14,629) were excluded from the analyses as the different cultural and lifestyle habits and disease predisposition of non-Swedish participants may cause confounding by population stratification in EWAS analyses. Participants with diagnosed diabetes and cardiovascular diseases (*n* = 3025) were also excluded to minimize bias attributable to diagnostic labelling and medications. The final dataset comprised 31,362 participants including 67,738 health examinations performed between 1990 and 2013. Written informed consent was obtained from all living participants as part of the VHU. The study was approved by the Regional Ethical Review Board in Umeå, and all research was conducted in accordance to this ethical approval and with the Declaration of Helsinki and other relevant guidelines and regulations.

### Clinical measurements

Nine cardiometabolic traits were analysed in the study: body mass index (BMI), systolic and diastolic blood pressures (SBP and DBP, respectively), fasting and 2 h glucose, total cholesterol, triglycerides, HDL cholesterol and LDL cholesterol. Clinical measures in VHU are described in detail elsewhere^10^. In brief, participants’ weight (in kg) and height (in cm) were measured using calibrated scale and stadiometer, with participants wearing light clothing and no shoes. BMI was calculated as body weight in kilograms divided by height in meters squared. SBP and DBP were measured once, after 5-min rest, with the participant in a recumbent position using either manual or automated sphygmomanometers. Capillary blood was drawn after overnight fasting and a second blood sample was drawn two hours after the administration of a 75-g oral glucose load. Blood glucose, total cholesterol and triacylglycerol levels were then measured using a Reflotron bench-top analyser (Roche Diagnostics Scandinavia AB). HDL cholesterol was measured in a subgroup of participants and LDL cholesterol was estimated using the Friedewald formula^11^. The measurement for lipids and blood pressure changed in September 2009. From this date onwards, blood pressure was measured twice in a sitting position and averaged, and total cholesterol and triglyceride levels were analysed using clinical chemical analysis in the laboratory. Thus, validated conversion equations were used to align the lipid and blood pressure measurements taken before and after September 2009^12^. For participants on lipid and/or blood pressure lowering medications, lipid and/or blood pressure levels were corrected by adding published constants (+ 0.208 mmol/l for triglycerides, + 1.347 mmol/l for total cholesterol, − 0.060 mmol/l for HDL cholesterol, + 1.290 mmol/l for LDL cholesterol, + 15 mmHg for SBP and + 10 mmHg for DBP)^13,14^. Values of cardiometabolic traits located outside the normal range suggested by VHU data managers (see Supplementary Material) were considered outliers and excluded.

### Lifestyle assessments

Participants were asked to complete a self-administered questionnaire during each visit that included questions about socio-economic factors, physical/mental health, quality of life, social network and support, working conditions, and alcohol/tobacco consumption. Physical activity was assessed through a modified version of the International Physical Activity Questionnaire^15,16^. A validated semi-quantitative food frequency questionnaire (FFQ) designed to capture habitual diet over the last year was used to capture information on various dietary factors^17^. Up to the mid-1990s, the FFQ consisted of 84 different foods items/groups, but it was reduced to 66 items in 1996 by combining similar line items and by removing items that provided minimal unique information. For the current analysis, matching food items from different FFQ versions were combined in new variables and all analyses including dietary variables were adjusted for FFQ version. In the FFQ, participants indicated how often they consumed foods and beverages on a nine-point frequency scale. Information on average portion size of meat and fish, vegetables, potatoes, rice and pasta was also gathered. Nutrient and energy content were calculated based on the Swedish Food Composition Database^18^ based on meal frequency and portion size. Food intake level (FIL) was calculated as total energy intake divided by estimated basal metabolic rate. Participants with more than 10% FFQ data missing, one or more portion indication missing, or a seemingly implausible total energy intake (the top 2.5% and bottom 5% of FIL in the original VHU dataset) were excluded from the analyses. Implausible values for other lifestyle variables (see Supplementary Material) were also removed from the analyses. Lifestyle variables were grouped in 10 different categories to facilitate understanding of the results: (i) alcohol consumption, (ii) non-alcoholic beverage consumption, (iii) food, (iv) nutrients; (v) general health, (vi) physical activity and fitness, (vii) psychosocial, (viii) sleep, (ix) social conditions, (x) tobacco use.

### Statistical analysis

**Figure 1:**
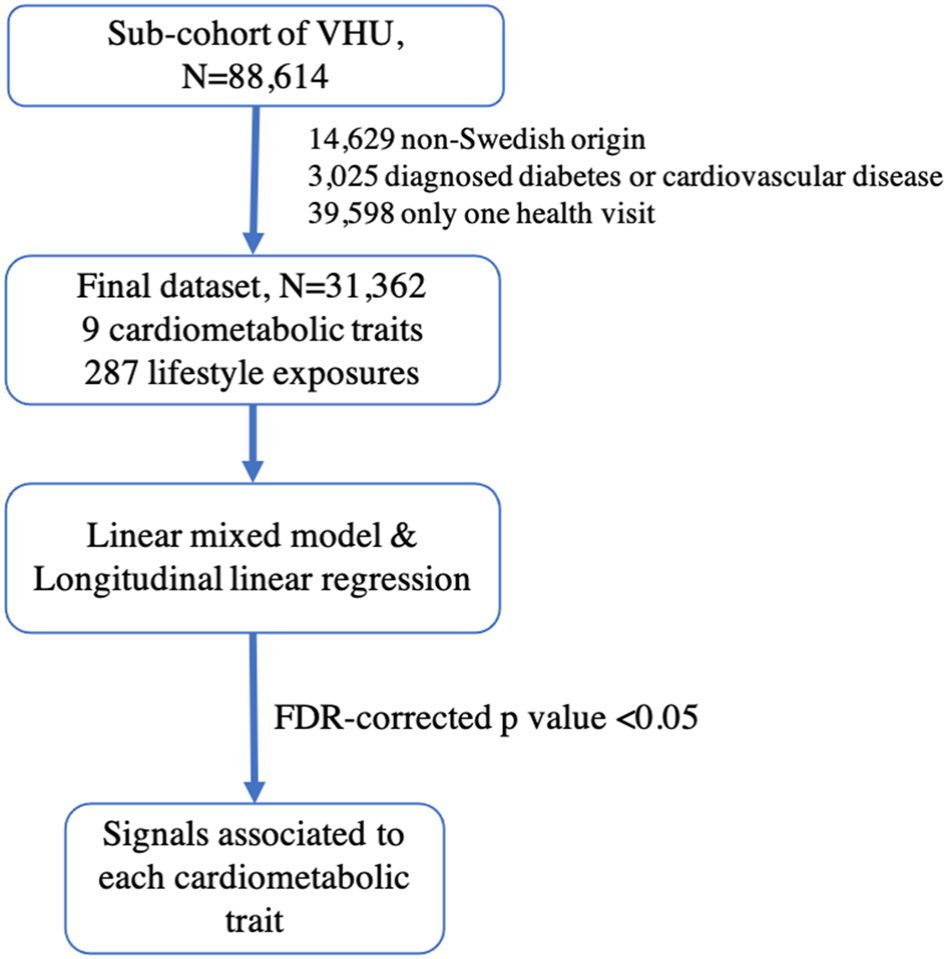
Flow chart of the method followed in the study.

The flowchart of the study is shown in Fig. 1. Lifestyle variables were treated either as continuous or as categorical variables; thus, ordinal variables were treated as continuous variables. For categorical variables with more than two levels dummy variables were created and dichotomized. All numeric lifestyle variables were inverse normalized in order to address skewness and scaled for comparability. Similarly, for categorical variables, levels were harmonized from low to high, using the lowest one as reference. Thirty-eight categorical variables that had 90% of the observations belonging to one category were excluded from the analyses. In total, the analyses included 242 numeric and 45 categorical lifestyle variables. Dietary variables were regressed on total energy intake and their residuals along with total energy intake were included in the analyses of these variables to account for potential confounding by total energy intake^19^. Models with glycaemic or lipid traits as the dependent variables were additionally adjusted for fasting status. All models (except models having BMI as outcome) were adjusted for BMI.

#### Average lifestyle associations

Linear mixed models were used to estimate an average linear effect of the lifestyle exposures on the cardiometabolic traits. The models were adjusted for age, age^2^, sex, educational level, follow-up time, FFQ version (where appropriate), total energy intake (TEI; where appropriate), BMI (where appropriate) and fasting status (where appropriate).

1 $$\begin{aligned} {\gamma}_{{{\text{ij}}}} & = \, (\beta_{00} + {\mu}_{{0{\text{j}}}} ) \, + \beta_{{{1}0}} {\text{age}}_{{{\text{ij}}}} + \beta_{{{2}0}} {\text{age}}^{{2}}_{{{\text{ij}}}} + \beta_{{{3}0}} {\text{sex}}_{{{\text{ij}}}} + \beta_{{{4}0}} {\text{follow}} \\ & \,\,\,\, - {\text{up time}}_{{{\text{ij}}}} + \beta_{{{5}0}} {\text{FFQ version}}_{{{\text{ij}}}} + \beta_{{{6}0}} {\text{TEI}}_{{{\text{ij}}}} + \beta_{{{7}0}} {\text{BMI}}_{{{\text{ij}}}} \\ & \,\,\,\, + \beta_{{{8}0}} {\text{fasting status}}_{{{\text{ij}}}} + \beta_{{{9}0}} {\text{lifestyle variable}}_{{{\text{ij}}}} + \varepsilon_{{{\text{ij}}}} \\ \end{aligned}$$

where *γ*_*i*j_ represents a cardiometabolic trait value at visit i for participant j, β00 is the fixed intercept, *μ*_0*j*_ represents different random intercepts for each participant, the rest of the *β* estimates are the estimated fixed effect size parameters for each corresponding variable, and ε represents error.

#### Long-term lifestyle associations

Linear regression models were used to test if the lifestyle variables were associated with 10-year changes in the cardiometabolic traits:

2 $$\begin{aligned} {\gamma}_{{\text{F}}} & = \alpha + \beta_{{1}} {\text{age}}_{{\text{B}}} + \beta_{{2}} {\text{age}}_{{\text{B}}}^{2} + \beta_{{3}} {\text{sex}} \\ & \,\,\,\, + \beta_{{4}} {\text{follow up time}}_{ + } \beta_{{6}} {\gamma}_{{{\text{B }} + }} \beta_{{7}} {\text{FFQ version}}_{{\text{B}}} + \beta_{{8}} {\text{TEI}}_{{\text{B}}} + \beta_{{9}} {\text{meanBMI}} \\ & \,\,\,\, + \beta_{{{1}0}} {\text{fasting status}}_{{\text{B}}} + \beta_{{{11}}} {\text{fasting status}}_{{\text{F}}} + \beta_{{{12}}} {\text{lifestyle variable}}_{{\text{B}}} + \varepsilon \\ \end{aligned}$$

where *γ*_*F*_ represents the value of the cardiometabolic trait at follow-up and *γ*_*B*_ the value at baseline, *α* is the intercept, *β*_*i*_ represent the estimated effect size parameter for each corresponding variable. Age_B_, FFQ version_B_, TEI_B_, fasting status_B_ and lifestyle variable_B_ are the age, FFQ version, TEI, fasting status and lifestyle variable values at baseline; fasting status_F_ is the fasting status value at follow up; meanBMI is the average BMI of the baseline and follow-up BMI values, and ε represents error.

#### Tentative signals

The Benjamini and Hochberg^20^ False Discovery Rate (FDR) was used to correct for multiple testing. Associations of lifestyle variables were considered “tentative signals” if they achieved significance at *P*_*FDR*_ < 0.05 after multiple testing correction. Overall estimates were used in the description of the results and effect estimates are reported in Supplementary material.

#### Correlation patterns

Correlations between ‘tentative signals’ on the linear mixed and/or longitudinal linear regression analyses were calculated and visualized using a heatmap. A hierarchical clustering algorithm was used to arrange lifestyle variables, so that the pair of variables with higher correlations appear closer in the heatmap.

#### Prioritization of modifiable lifestyle variables

Tentative signals for each of the cardiometabolic traits were gathered and prioritized to identify target lifestyle exposures and exposure groups in which lifestyle interventions aiming at controlling cardiometabolic diseases may focus. First, variance explained for each lifestyle variable (and covariates) was estimated and variables were rank-ordered within each lifestyle category for each of the nine outcome traits. In the linear mixed models, marginal (fixed terms) variance explained was used. The top-ranked variables (five per category per trait) were identified, and the topranked variables represented in the majority of the cardiometabolic traits (at least five traits) were prioritized. Target groups were evaluated using a hierarchical clustering algorithm based on correlations between the prioritized variables and visualized in a heatmap. Non-modifiable variables were excluded from the prioritization and clustering step as these variables could not be affected by a lifestyle intervention.

Statistical analyses and data visualization were performed using *R* software versions 3.5.2 and 3.6.1^21^ (see Supplementary Material for the specific packages used for analyses).

## Results

Descriptive characteristics of the study population are summarized in Tables 1, S1 and S2. Mean age of participants was 47.7 years and 50.6% were women.

**Table 1:** Summary of participant characteristics.

Variable	Number of observations	mean	SD
Age (years)	67,738	47.72	8.92
Height (cm)	67,476	172.04	9.21
BMI (kg/m2)	67,413	25.73	3.99
Waist circumference (cm)	28,621	92.27	12.13
Total cholesterol (mmol/L)	67,181	5.51	1.09
HDL-C (mmol/L)	27,803	1.40	0.47
LDL-C (mmol/L)	27,649	3.86	1.03
Triglycerides (mmol/L)	58,905	1.40	0.78
Fasting glucose (mmol/L)	67,339	5.39	0.75
2-h glucose (mmol/L)	64,951	6.57	1.50
SBP (mmHg)	67,193	126.42	17.54
DBP (mmHg)	67,160	78.83	11.29

*BMI* Body mass index, *HDL-C* High-density lipoprotein cholesterol, *LDL-C* Low-density lipoprotein cholesterol, *SBP* Systolic blood pressure, *DBP* Diastolic blood pressure, *SD* Standard deviation.

### Average lifestyle associations

**Figure 2:**
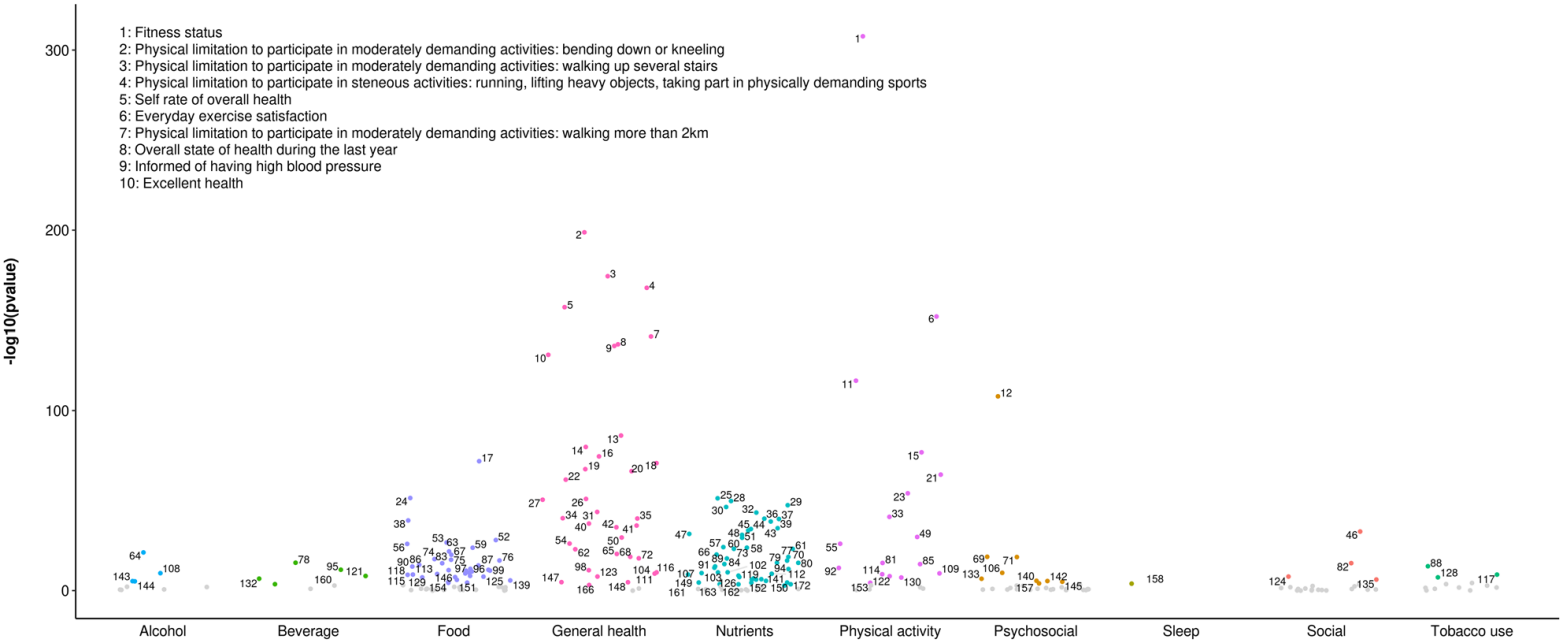
Manhattan plot representing the distribution of *P* values of the association of lifestyle variables and BMI by lifestyle category. Tentative signals are coloured, and number labelled in the figure and the top 10 variables are spelled out. See S25 for references to the labels.

**Figure 3:**
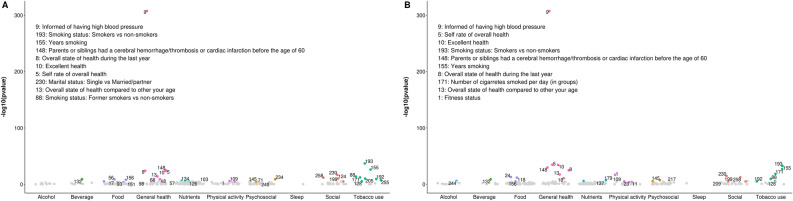
Manhattan plot representing the distribution of *P* values of the association of lifestyle variables and blood pressure traits by lifestyle category. (**A**) Systolic blood pressure and (**B**) Diastolic blood pressure. ‘Tentative signals’ are coloured, and number labelled in the figure and the top 10 variables are spelled out. See S25 for references to the labels.

**Figure 4:**
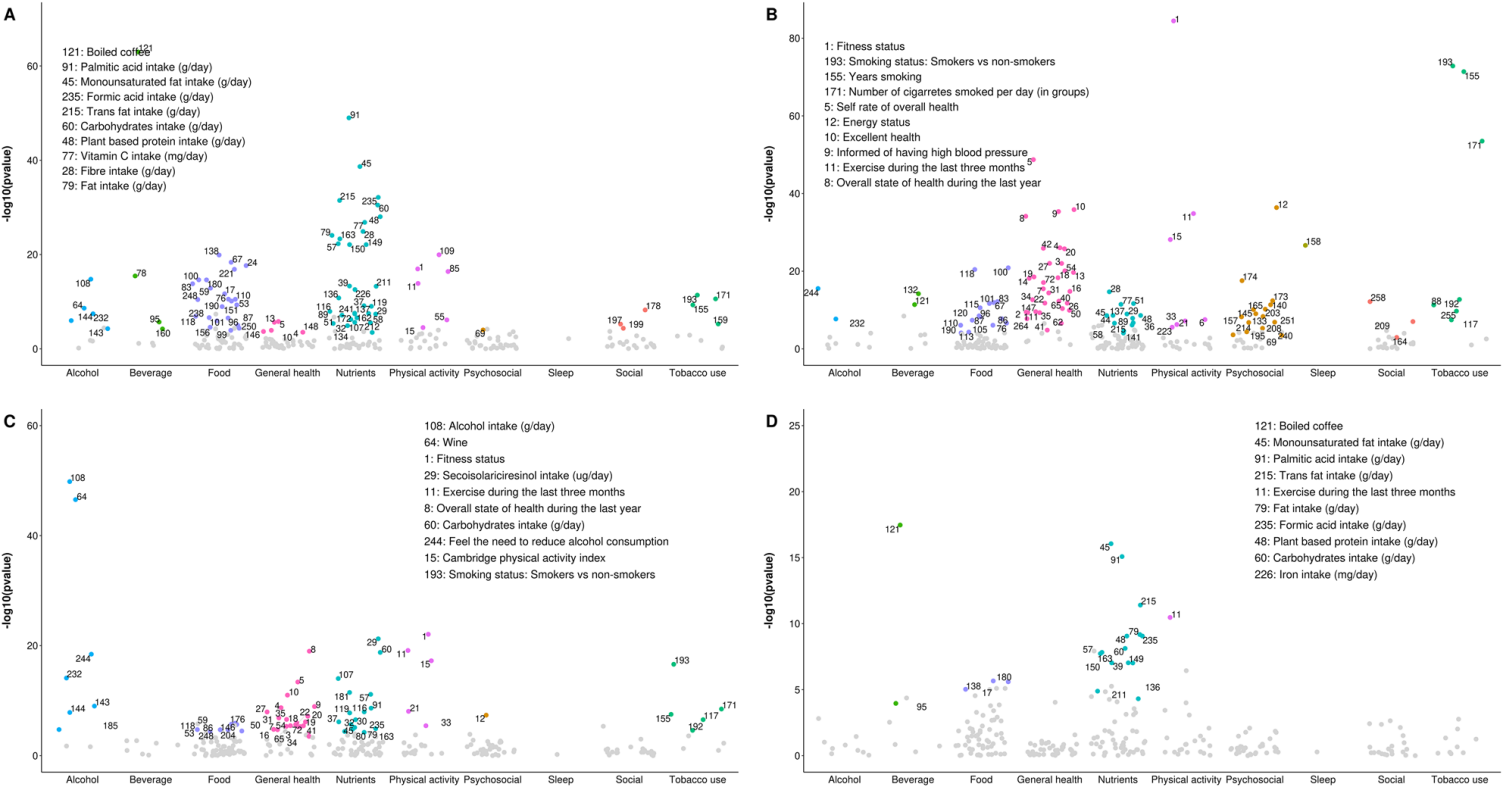
Manhattan plot representing the distribution of *P* values of the association of lifestyle variables and lipid traits by lifestyle category. (**A**) Total cholesterol, (**B**) Triglycerides, (**C**) HDL cholesterol and (**D**) LDL cholesterol. Tentative signals are coloured, and number labelled in the figure and the top 10 variables are spelled out. See S25 for references to the labels.

**Figure 5:**
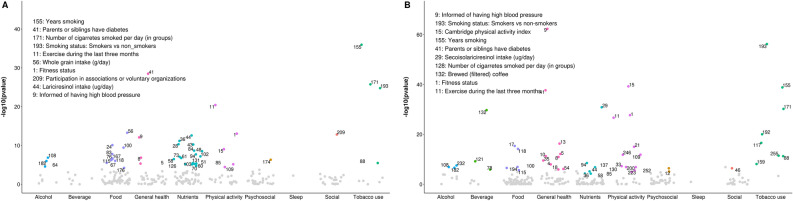
Manhattan plot representing the distribution of *P* values of the association of lifestyle variables and glucose traits by lifestyle category. (**A**) Fasting glucose and (**B**) 2 h glucose. Tentative signals are coloured, and number labelled in the figure and the top 10 variables are spelled out. See S25 for references to the labels.

164 out of 286 lifestyle variables were considered tentative signals for BMI (S3), 37 for SBP (S4), 30 for DBP (S5), 84 for total cholesterol (S6), 96 for triglycerides (S7), 46 for HDL cholesterol (S8), 20 for LDL cholesterol (S9), 44 for fasting glucose (S10) and 43 for 2 h glucose (S11). ‘Physical activity’ and ‘General health’ were the top categories for BMI (Fig. 2) and ‘General health’ for blood pressure traits (Fig. 3). Regarding lipids, ‘Beverage’, ‘Nutrients’ and ‘ Physical activity’ were the categories with the highest number of ‘tentative signals’ for total and LDL cholesterol (Figs. 4A and D), while ‘Physical activity’, ‘Tobacco use’ and ‘General health’ were the top categories for triglycerides (Fig. 4B), and ‘Alcohol’ for HDL cholesterol (Fig. 4C). For glucose traits, ‘Physical activity’, ‘General health’ and ‘Tobacco use’ were the top categories (Fig. 5).

### Long-term lifestyle associations

After multiple testing correction, 35 lifestyle variables showed a tentative association with 10-year change in BMI (S12), 3 with change in SBP and DBP (S13-S14), 15 with change in total cholesterol (S15), 10 in triglycerides (S16), none in HDL and LDL cholesterol (S17-S18), 5 in fasting glucose (S19) and 8 in 2 h glucose (S20). The majority of the ‘tentative signals’ were in the ‘Tobacco use’ category for BMI, lipids and fasting glucose, while for blood pressure traits the top category was ‘General health’ and for 2 h glucose, ‘Physical activity’, ‘Food’, and ‘General health’ were the top categories. There were no material changes in key outcome variables during the 9-year follow-up period (see Supplementary Material).

### Correlation patterns

**Figure 6:**
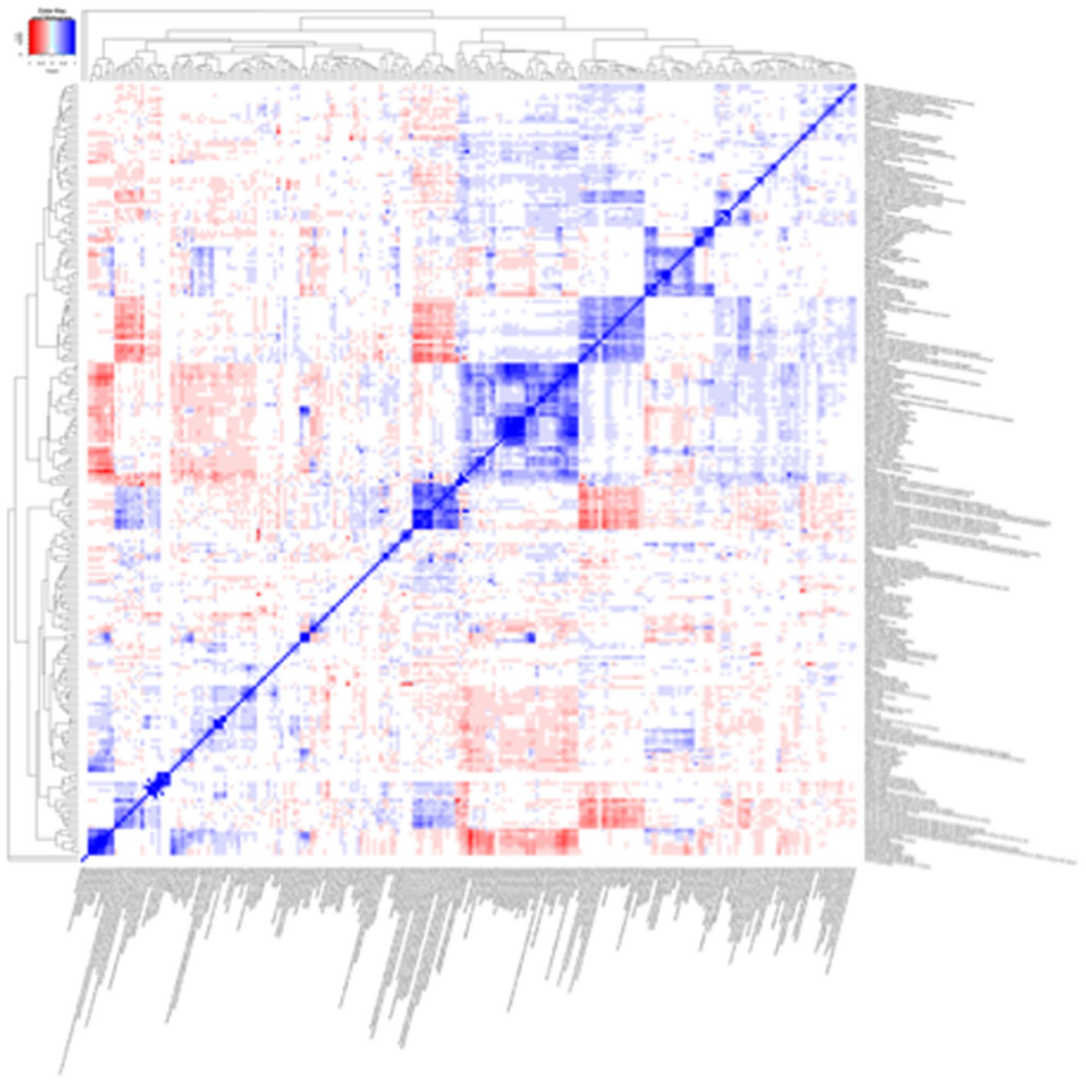
Heat map showing all the correlations for tentative signals. Pairs of factors where correlations could not be computed are shown in white. Figures were plotted using ‘ggplot2’, ‘ggrepel’, ‘gridExtra’, ‘RColorBrewer’ and ‘gplots’ packages in *R* software versions 3.5.2 and 3.6.1^21^.

Patterns of correlations were identified among lifestyle variables showing tentative association with any of the cardiometabolic traits based on the correlation heatmap (Fig. 6). Variables related to meat and fish consumption, sodium, calcium, vitamin B12, and total and animal based protein intake appeared in close proximity showing correlations around 0.5. Variables describing fat consumption and fatty acid intakes were grouped together showing a high positive correlation. Variables assessing vegetable, fibre and fruit intake, plant lignans, whole grain intake, and carbohydrates intake also appear near each other in the heatmap showing high positive correlations between them and negative correlations with fat related variables. Variables in ‘Psychosocial’ category and ‘General health’ variables were grouped together.

### Prioritization of modifiable lifestyle variables

#### Average lifestyle associations

**Figure 7:**
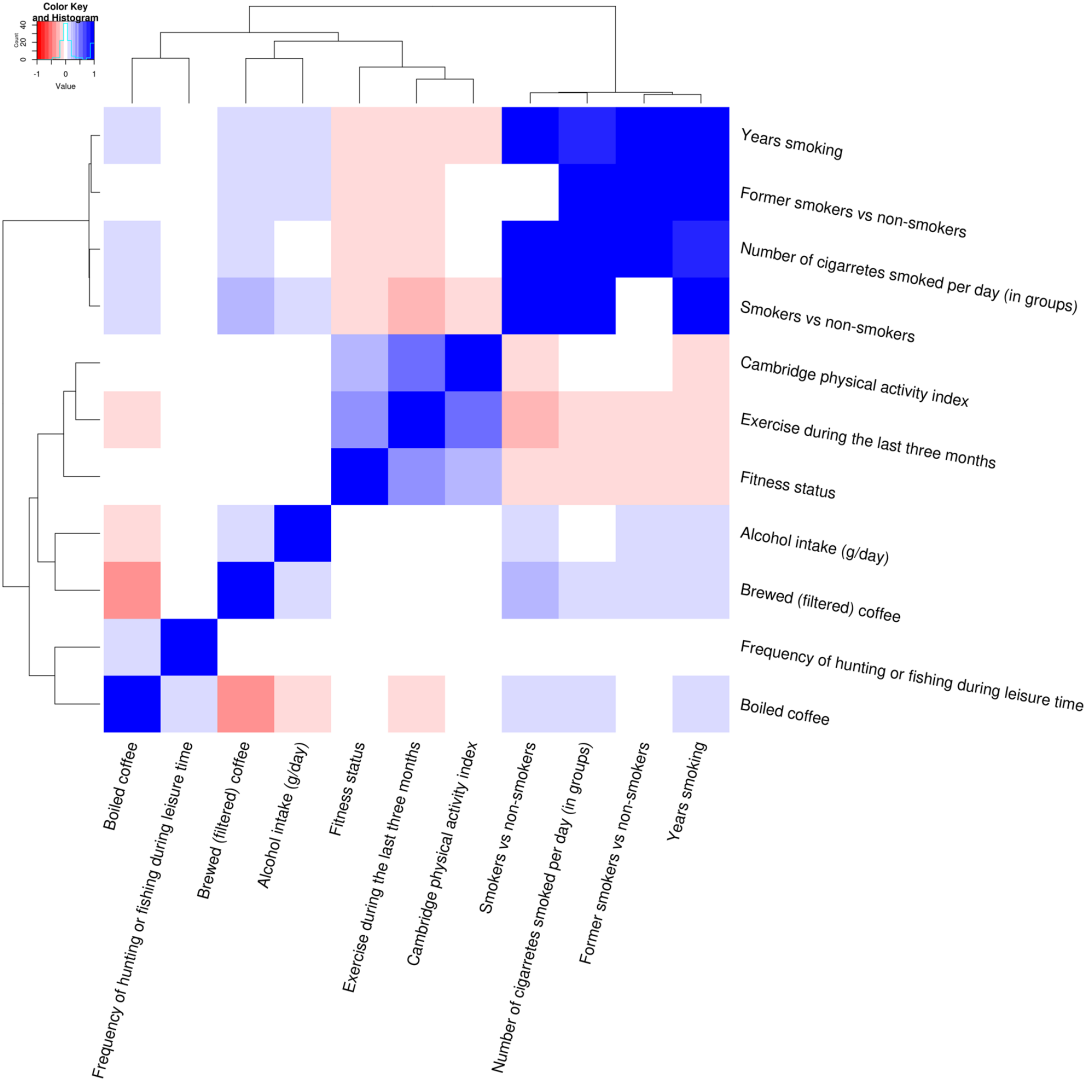
Heat map showing clusters of correlations between top-ranked modifiable lifestyle variables. Figures were plotted using ‘ggplot2’, ‘ggrepel’, ‘gridExtra’, ‘RColorBrewer’ and ‘gplots’ packages in *R* software versions 3.5.2 and 3.6.1^21^.

Thirteen variables were prioritized among all the ‘tentative signals’ as they showed the most consistent associations across all the cardiometabolic traits (top-ranked in at least 5 out of 9 cardiometabolic traits) (S21). Two of these variables (‘Informed of having a high blood pressure’ and ‘Overall state of health during the last year’) were considered non-modifiable and excluded (S26 for modifiable and non-modifiable variables). The eleven remaining variables were included in a hierarchical clustering algorithm which identified four main targets suitable for interventions (Fig. 7). The first group included tobacco use/smoking related variables and were in general positively associated with BMI, fasting glucose, total cholesterol and triglycerides and negatively with blood pressure traits, HDL cholesterol and 2 h glucose (S21). The second included ‘Brewed (filtered) coffee’, which was negatively associated with BMI, blood pressure traits, triglycerides and 2 h glucose. The third group included physical activity related variables (e.g. ‘Exercise during the last three months’). The fourth included the variable ‘alcohol intake (g/day)’. These variables were in general negatively associated with all cardiometabolic traits except with HDL-C with which they showed a positive association. The fifth group was a composite of lifestyle variables which could be linked to the Swedish lifestyle (especially northern Swedish lifestyle), ‘Frequency of hunting or fishing during leisure time’ and ‘Boiled coffee’ (S26). These two variables did not show a clear common pattern of associations with cardiometabolic traits.

In general, BMI showed more shared tentative signals with 2 h glucose and HDL-cholesterol than with the rest of cardiometabolic traits and triglycerides, BMI and 2 h glucose were the cardiometabolic traits sharing the highest number of tentative signals with the rest of cardiometabolic traits (S22).

#### Long-term lifestyle associations

None of the ‘tentative signals’ showed a consistent association with the majority of cardiometabolic traits (5 out 9 traits) (S23). However, four variables in the ‘Tobacco use’ category showed a consistent positive association with 10-year changes in at least three cardiometabolic traits (BMI, total cholesterol, triglycerides and/or fasting glucose).

Among all the cardiometabolic traits BMI and lipid traits shared the highest number of tentative signals (S24).

## Discussion

Although EWAS analyses have been reported previously, this is the first study to integrate repeated exposures and outcome assessments, which allows inferences about long-term exposure to these risk factors to be made. Here, we systematically and agnostically assessed average (across the study’s follow-up time) and ~ 10-year associations between 286 lifestyle variables and 9 cardiometabolic traits. In analyses assessing average association of lifestyle variables, ‘Physical activity’ and ‘General Health’ were the categories containing the highest number of tentative signals and 11 modifiable variables were prioritized for lifestyle interventions focused on controlling cardiometabolic diseases. A cluster analyses grouped these 11 variables into five main target groups: (i) Smoking, (ii) Beverage (filtered coffee), (iii) physical activity, (iv) alcohol intake, and (v) specific variables related to Swedish lifestyle (hunting/fishing during leisure time and boiled coffee).

For 10-year associations, ‘Tobacco use’ was the category including the highest number of tentative signals for the majority of the cardiometabolic traits. No modifiable lifestyle variable was consistently associated with the majority of cardiometabolic traits but four variables in the ‘Tobacco use’ category were consistently associated with at least three of the analysed cardiometabolic traits (BMI, total cholesterol, triglycerides and/or fasting glucose).

Smoking and physical activity correspond to two of the most well-known modifiable risk factors for cardiometabolic diseases. According to a study analysing the burden of disease caused by physical inactivity, worldwide, 6% of the burden of coronary heart disease and 7% of type 2 diabetes was caused by physical inactivity^22^. On the other hand, smoking alters lipid metabolism and glucose homeostasis through the increase in lipolysis, insulin resistance and tissue lipotoxicity^23,24^ and smoking cessation restores, at least in part, these metabolic alterations. However, in our study the association of smoking with cardiometabolic traits was not only restricted to the average effect across the studied period but we also found a remarkable association of variables included in the ‘Tobacco use’ category and cardiometabolic traits in the 10 years of follow-up.

Among the prioritized dietary variables, boiled (unfiltered) coffee but not brewed (filtered) coffee was found positively associated with lipid traits, specifically with total cholesterol, triglycerides, and LDL cholesterol. Previous studies have also identified associations between unfiltered coffee and dose-dependent increase of plasma concentrations of total and LDL cholesterol^25,26^. The effects of coffee in the lipid profile are probably caused by two diterpenes (i.e. kahweol and cafestol), which sometimes get trapped in the filter used to make coffee which can explain the differential effects of filtered and unfiltered coffee^26^. On the other hand, brewed (filtered) coffee was found negatively associated with BMI, blood pressure, triglycerides, and 2 h glucose in the present study which is in agreement with previous studies showing an inverse association between habitual coffee intake and risk of several cardiometabolic diseases^27,28^.

Plant lignans (biphenolic compounds found in tea, coffee, whole-grain products, berries, vegetables, fruit, nuts and seeds) were among the top tentative signals for fasting and 2 h glucose, showing a negative association with both traits. Previous studies have suggested that lignans and their metabolites may protect against cardiovascular disease and metabolic syndrome by reducing lipid concentrations, lowering blood pressure, and decreasing oxidative stress and inflammation^29^. A study conducted in Finland found that men with high serum concentrations of enterolactone (a lignan produced by the intestinal microflora) had a lower risk of acute coronary events than men with lower concentrations^30^.

An interesting observation emerging from our analysis is that several variables that are featured in public health recommendations were not broadly associated with the cardiometabolic traits studied here. Recommended dietary patterns emphasize the importance of limiting the consumption of sugar-rich products, particularly sweet drinks^31^. However, variables related to sweets and sweet drink consumption (e.g. “Sodas, soft drinks, juice” and “Sweets”) were not identified as tentative signals for any of the cardiometabolic traits. Salt content is also usually limited in diets recommended to lower risk of cardiometabolic diseases but “Sodium intake” was not consistently associated with cardiometabolic traits, being identified as a tentative signal only for BMI, total and HDL cholesterol. In the same way, fish and shellfish are frequently recommended in healthy dietary patterns but “Lean fish” and “Shellfish” variables were not tentative signals for any cardiometabolic traits, and “Fatty fish” was associated with lipid traits except for LDL cholesterol.

There are also limitations to the present study. EWAS and GWAS are not entirely analogous. However, both are experiment-wide association studies that adopt a so called ‘agnostic’ approach to consider a multitude of exposure-outcome relationships in parallel. This is hence a ‘data-driven’ approach that contrasts traditional association studies, where specific hypotheses are formulated and only those relationships consistent with the hypothesis are tested. The present sample is limited to a Swedish population between 30–70 years and thus caution should be used when extrapolating the findings to other countries and age groups, especially since lifestyle variables affecting cardiometabolic traits in Swedish population might differ from other populations. Dietary variables were characterized using an FFQ, which suffer from systematic and random measurement errors. However, to minimize this source of error the FFQ used in this study was validated against repeated 24 h recalls^17^. VHU cohort is exceptionally well-powered for analyses of the nature performed here and there were, consequently, a large number of associations that passed conventional statistical thresholds. Most of these statistically robust associations emerged due to the complex correlation structure (Fig. 6) found within the set of exposure variables. The EWAS analyses undertaken here, like those reported elsewhere, involve parallel tests of association with cardiometabolic traits for an array of variables, in this case modifiable lifestyle exposures. As with all observational analyses in free-living populations, including EWAS, there is a risk that the relationships observed are prone to confounding and reverse-causality. To mitigate these risks, we adjusted the regression models for putative confounding variables and assessed the key findings in both average and long-term models. Even with these attempts, it is important to highlight that one or more of the findings are false-positive owing to residual confounding. To assess this thoroughly requires appropriately designed experimental studies. Our findings highlight key variables, along with their respective effect estimates, that might be prioritised for subsequent prediction models and lifestyle interventions. However, it is important to keep in mind that epidemiological associations of this nature may not be causal. Thus, intervention studies are needed to test the causal nature of these associations.

In conclusion, using an EWAS approach in a large prospective Swedish cohort a large number of associations between lifestyle exposures and cardiometabolic traits were identified. Eleven modifiable exposures were consistently top-ranked among the majority of cardiometabolic traits and were identified as target lifestyle exposures that could inform lifestyle interventions aiming at controlling cardiometabolic diseases. These variables belonged to four target groups: (i) Smoking, (ii) Beverage (specifically brewed (filtered) coffee) and (iii) Leisure time physical activity and (iv) a group of lifestyles more specific to the Swedish lifestyle.

## Supplementary Information

Supplementary Information 1.

Supplementary Information 2.
