# Supplementary material for: Exposome-wide ranking of modifiable risk factors for cardiometabolic disease traits
Source: Sci Rep. 2022 Mar 8;12:4088. doi: 10.1038/s41598-022-08050-1 (PMC8904494; doi:10.1038/s41598-022-08050-1)
Supplement: Supplementary file 1 — Supplementary Information 1. [file 41598_2022_8050_MOESM1_ESM.pdf]

Supplementary Material

Limit values proposed by VIP for exclusions on cardiometabolic traits

Height: <130 cm or >210 cm

Weight: <35 kg

BMI: <15 kg/m^2 or >70 kg/m^2

Systolic blood pressure: <20 or >300

Diastolic blood pressure: <20 or >250

Total cholesterol: <0.5 mmol/l or >15 mmol/l

Triglycerides: <0.15 mmol/l or >20 mmol/l. Triglycerides values lower than 0.8 mmol/l were additionally excluded due to the sensitivity of the Reflotron benchtop analyser.

HDL-cholesterol: <0.15 mmol/l or>7 mmol/l

LDL-cholesterol: Not defined. LDL cholesterol values lower than 0.5 mmol/l and higher than 13 mmol/l were excluded.

Fasting glucose: <1 mmol/l or >25 mmol/l. Fasting glucose values lower than 2 mmol/l were additionally excluded as they were considered biologically implausible.

2h glucose: <1 mmol/l or >35 mmol/l. 2h glucose values lower than 2 mmol/l were additionally excluded as they were considered biologically implausible.

Values considered implausible for certain lifestyle variables

Distance to work in kilometres (one way): All answers beyond 200 km were excluded.

Grams of tobacco smoked per week: All answers equal or beyond 350 gr/week were excluded.

Arachidonic acid (ARA) intake (g/day): All answers equal or beyond 0.9 gr/day were excluded.

Eicosapentaenoic acid (EPA) intake (g/day): All answers equal or beyond 2 gr/day were excluded.

Sodium intake (mg/day): All answers equal or beyond 10000 mg/day were excluded.

R packages used in the analyses

Data manipulation: psych (1), data.table (2), plyr (3), dplyr (4) and tidyverse (5)

EWAS analyses: getopt (6) and nlme (7)

Metanalysis: meta (8)

R^2 estimation: piecewiseSEM (9)

Correlation estimation: polycor (10)

Data visualization: ggplot2 (11), ggrepel (12), gridExtra (13), RColorBrewer (14) and gplots (15)

SUPPLEMENTARY FIGURE

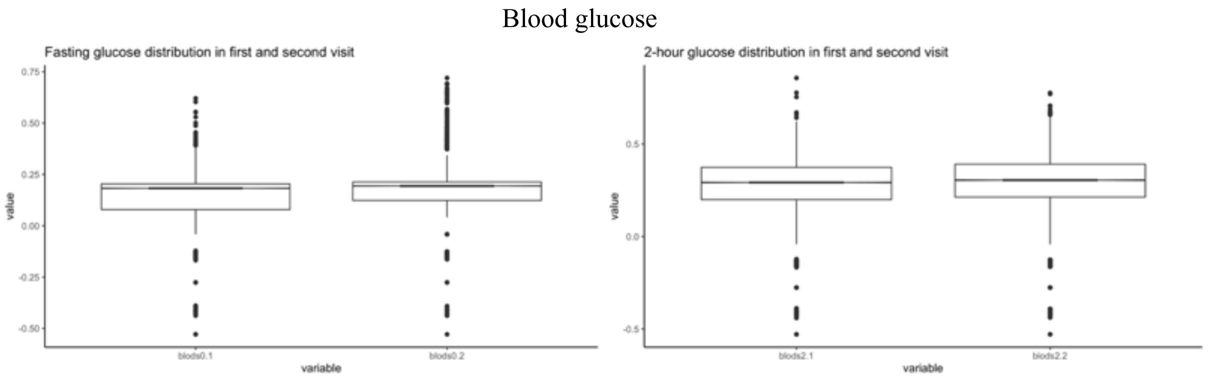

Figure 1. Boxplots representing the distribution of Fasting glucose (left hand) and Two-hour glucose (right hand) at baseline and follow-up visit

Blood pressure

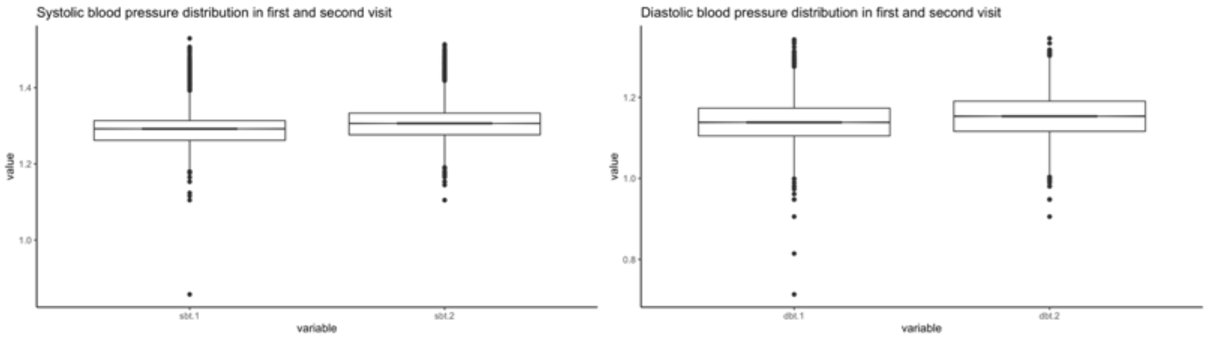

Figure 2. Boxplots representing the distribution of Systolic blood pressure (left hand) and Diastolic blood pressure (right hand) at baseline and follow-up visit

Blood lipids

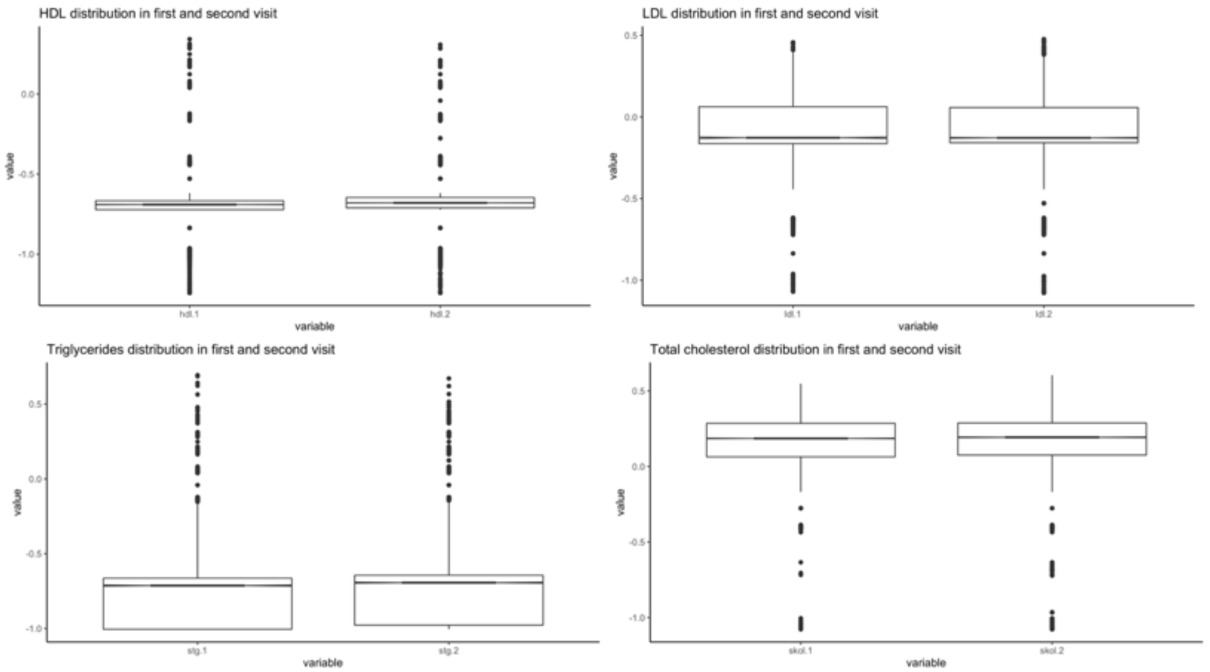

Figure 3. Boxplots representing the distribution of high-density cholesterol (upper left hand), low-density cholesterol (upper right hand), triglycerides (lower left hand), and total cholesterol (lower right hand) at baseline and follow-up visit

BMI

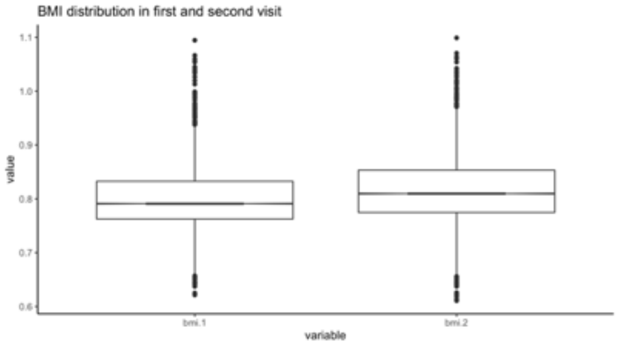

Figure 4. Boxplots representing the distribution of body mass index at baseline and follow-up visit

References:

1. Revelle W. psych: Procedures for Personality and Psychological Research. Evanston, Illinois, 2019.
2. Dowle M, Srinivasan A. data.table: Extension of `data.frame`. 2019.
3. Wickham H. The Split-Apply-Combine Strategy for Data Analysis. *Journal of Statistical Software* 2011;40(1):1-29.
4. Wickham H, Francois R, Henry L, et al. dplyr: A Grammar of Data Manipulation., 2020.
5. Wickham H, Averick M, Bryan J, et al. Welcome to the tidyverse. *Journal of Open Source Software* 2019;4(43):1686.
6. Davis TL, Day A. getopt: C-Like 'getopt' Behavior. 2019.
7. Pinheiro J, Bates D, DebRoy S, et al. nlme: Linear and Nonlinear Mixed Effects Models. 2020.
8. Balduzzi S, Rücker G, Schwarzer G. How to perform a meta-analysis with {R}: a practical tutorial. *Evidence-Based Mental Health* 2019.
9. Lefcheck JS. piecewiseSEM: Piecewise structural equation modelling in r for ecology, evolution, and systematics. *Methods in Ecology and Evolution* 2016;7(5):573-9.

10. Fox J. polycor: Polychoric and Polyserial Correlations. 2019.

11. Wickham H. *ggplot2: Elegant Graphics for Data Analysis*. Springer-Verlag New York; 2016.

12. Slowikowski K. ggrepel: Automatically Position Non-Overlapping Text Labels with 'ggplot2'. 2020.

13. Auguie B. gridExtra: Miscellaneous Functions for "Grid" Graphics. 2017.

14. Neuwirth E. RColorBrewer: ColorBrewer Palettes. 2014.

15. Warnes GR, Bolker B, Bonebakker L, et al. gplots: Various R Programming Tools for Plotting Data. 2020.
