# Supplementary material for: Exposome-wide ranking of modifiable risk factors for cardiometabolic disease traits
Source: Sci Rep. 2022 Mar 8;12:4088. doi: 10.1038/s41598-022-08050-1 (PMC8904494; doi:10.1038/s41598-022-08050-1)
Supplement: Supplementary file 2 — Supplementary Information 2. [file 41598_2022_8050_MOESM2_ESM.pdf]

Supplementary Table 1. Descriptive statistics of the numeric lifestyle variables

Variables	Description	N of observations	mean	SD	median	min	max
age	age (years)	67738	47.72	8.92	49.90	21.50	70.80
langd	height (cm)	67476	172.04	9.21	172.00	130.00	203.00
bmi	body mass index (kg/m2)	67413	25.73	3.99	25.17	15.21	68.78
midja	waist circumference (cm)	28621	92.27	12.13	92.00	60.00	160.00
skol	total cholesterol (mmol/L)	67181	5.51	1.09	5.44	0.50	14.98
hdl	HDL (mmol/L)	27803	1.40	0.47	1.33	0.15	7.00
ldl	LDL (mmol/L)	27649	3.86	1.03	3.80	0.50	10.02
stg	triglycerides (mmol/L)	58905	1.40	0.78	1.17	0.80	18.97
blods0	fasting glucose (mmol/L)	67339	5.39	0.75	5.40	2.00	20.20
blods2	2-hour glucose (mmol/L)	64951	6.57	1.50	6.50	2.00	28.90
sbt	systolic blood pressure (mmHg)	67193	126.42	17.54	124.00	29.00	240.00
dbt	diastolic blood pressure (mmHg)	67160	78.83	11.29	79.00	20.00	147.00
antal_km	Distance to work in kilometers (one way)	57476	13.99	17.48	6.00	0.00	188.00
sm_num_cig	Number of cigarretes smoked per day	43292	0.31	2.14	0.00	0.00	50.00
sm_num_cigar	Number of cigars smoked per day	42164	0.01	0.27	0.00	0.00	25.00
sm_gr_tobacco	Grams of tobacco smoked per week	42200	0.09	2.75	0.00	0.00	275.00
sm_duration	Years smoking	58487	9.42	13.13	0.00	0.00	52.00
sn_time	Years using snuff	62969	4.51	9.41	0.00	0.00	51.00
year	year	65366	2002.10	6.49	2002.00	1990.00	2013.00
FIL	Food intake level	63312	1.08	0.38	1.03	0.09	17.97
ensum1	Total energy intake (kcal/day)	58812	1754.64	540.19	1672.45	663.16	4363.79
protsum1	Total protein intake (g/day)	58812	63.87	20.43	60.86	11.28	201.49
protsum1_anim	Animal based protein intake (g/day)	58812	45.40	16.45	42.90	0.13	178.47
protsum1_veg	Plant based protein intake (g/day)	58812	18.47	6.95	17.39	0.49	72.43
kolhsum1	Carbohydrates intake (g/day)	58812	209.44	70.20	199.29	17.72	663.42
sacksum1	Sucrose intake (g/day)	58812	27.42	16.17	23.71	0.77	178.93
DISAsum1	Disaccharides intake (g/day)	58812	50.58	22.74	46.53	4.05	242.42
MOSAsum1	Monosaccharides intake (g/day)	58812	25.05	11.80	23.05	1.87	135.93
fibesum1	Fibre intake (g/day)	58812	18.82	7.09	17.85	1.31	78.20
FULLKsum1	Whole grain intake (g/day)	58812	69.00	35.06	63.66	0.00	307.12
alkosum1	Alcohol intake (g/day)	58812	4.43	4.74	3.21	0.00	123.62
fettsum1	Fat intake (g/day)	58812	68.49	26.40	63.85	10.52	297.24
mfetsum1	Saturated fat intake (g/day)	58812	28.63	12.21	26.39	2.97	151.84
MONOsum1	Monounsaturated fat intake (g/day)	58812	23.44	9.29	21.70	3.05	101.43
POLYsum1	Polyunsaturated fat intake (g/day)	58812	10.80	5.09	9.72	1.90	74.51
TRANSsum1	Trans fat intake (g/day)	58812	1.75	1.24	1.41	0.01	15.33
kolesum1	Cholesterol intake (g/day)	58812	0.20	0.08	0.18	0.01	0.90
FA140_sum1	Formic acid intaje (g/day)	58812	3.26	1.66	2.93	0.11	21.71
FA160_sum1	Palmitic acid intake (g/day)	58812	14.53	5.82	13.44	1.97	73.01
FA182_sum1	Linoleic acid intake (g/day)	58812	7.83	4.06	6.93	1.47	59.71
FA183_sum1	Linolenic acid intake (g/day)	58812	1.60	0.73	1.46	0.26	9.48
FA204_sum1	Arachidonic acid (ARA) intake (g/day)	58810	0.09	0.05	0.08	0.00	0.68
FA205_sum1	Eicosapentaenoic acid (EPA) intake (g/day)	58811	0.07	0.06	0.05	0.00	1.43
FA226_sum1	Docosahexaenoic acid (DHA) intake (g/day)	58812	0.14	0.12	0.12	0.00	4.40
FA150_sum1	Pentadecanoic acid intake (g/day)	58812	0.23	0.15	0.20	0.00	2.03
FA170_sum1	Heptadecanoic acid intake (g/day)	58812	0.13	0.09	0.11	0.00	1.18
MAGNsum1	Magnesium intake (mg/day)	58812	292.36	88.25	280.19	76.03	1154.65
FOSFsum1	Phosphate intake (mg/day)	58812	1178.85	393.86	1121.77	249.07	3757.68
NIACsum1	Vitamin B3 intake (mg/day)	58812	15.10	5.05	14.23	3.08	61.00
selesum1	Selenium intake (ug/day)	58812	22.69	8.24	21.45	3.00	128.78
ZINCsum1	Zinc intake (mg/day)	58812	8.19	2.67	7.79	1.36	28.59
retisum1	Vitamin A intake (mg/day)	58812	0.61	0.32	0.54	0.03	5.98
karosum1	Beta-carotene intake (mg/day)	58812	4.25	4.57	2.72	0.07	45.85
TIAMsum1	Tiamin intake (mg/day)	58812	1.21	0.41	1.15	0.17	4.38
Folasum1	Folic acid intake (ug/day)	58812	233.07	90.70	218.31	42.18	1052.67
B2sum1	Vitamin B2 intake (ug/day)	58812	1.43	0.52	1.36	0.26	5.20
B6sum1	Vitamin B6 intake (mg/day)	58812	1.91	0.65	1.81	0.45	9.29
B12sum1	Vitamin B12 intake (ug/day)	58812	4.67	2.12	4.28	0.03	28.27
askosum1	Vitamin C intake (mg/day)	58812	73.97	45.45	63.21	0.71	535.69
Dsum1	Vitamin D intake (ug/day)	58812	5.39	2.17	5.04	0.63	33.19
tokosum1	Vitamin E intake (mg/day)	58812	6.37	2.32	5.99	1.61	31.05
VITKsum1	Vitamin K intake (ug/day)	58812	3.91	2.35	3.32	0.00	29.11
jernsum1	Iron intake (mg/day)	58812	12.23	4.31	11.51	1.88	46.73
JODIsum1	Iodine intake (ug/day)	58812	121.31	50.57	113.11	5.53	649.79
kalcsum1	Calcium intake (mg/day)	58812	784.82	341.04	732.30	92.10	3244.76
KALIsum1	Potassium intake (mg/day)	58812	3187.33	996.55	3049.86	685.88	14480.73
Bstrsum1	Beta-sitosterol intake (mg/day)	58812	147.05	51.89	139.45	8.44	603.68
Bstnsum1	Beta-sitostanol intake (mg/day)	58812	8.37	3.62	7.73	0.14	34.95
Cstrsum1	Campesterol intake (mg/day)	58812	56.04	22.79	52.11	2.54	256.68
Cstnsum1	Campestanol intake (mg/day)	58812	5.45	2.64	4.96	0.05	25.75
Sstrsum1	Stigmasterol intake (mg/day)	58812	11.50	4.15	10.88	0.76	50.25
Tstrsum1	Sum of phytosterols intake (mg/day)	58812	228.41	81.20	216.20	11.92	923.76
Lig_Endsum1	Enterodiol intake (ug/day)	58812	0.01	0.02	0.00	0.00	0.16
Lig_Enlsum1	Enterolactone intake (ug/day)	58812	5.52	5.67	3.94	0.00	62.29
Lig_Equsum1	Equol intake (ug/day)	58812	0.25	0.38	0.06	0.00	3.72
Lig_Larsum1	Lariciresinol intake (ug/day)	58812	507.92	211.94	475.34	42.82	2638.93
Lig_Matsum1	Matairesinol intake (ug/day)	58812	40.73	19.60	37.68	1.63	178.21
Lig_Medsum1	Medioresinol intake (ug/day)	58812	507.99	288.98	452.54	0.23	2726.08
Lig_Pinsum1	Pinoresinol intake (ug/day)	58812	393.45	189.62	363.68	11.70	2372.68
Lig_Secsum1	Secoisolariciresinol intake (ug/day)	58812	140.35	48.21	134.69	13.07	725.99
Lig_Syrsum1	Syringaresinol intake (ug/day)	58812	1329.07	700.77	1199.79	0.23	6603.59
Lig_Sumsum1	Sum of all lignans intake (ug/day)	58812	2925.30	1373.35	2696.83	174.30	12827.25
Lig4sumsum1	Sum of Lariciresinol, Matairesinol, Pinoresinol, Secoisolariciresinol intake (ug/day)	58812	1082.46	448.37	1013.66	87.72	5749.52
NATRsum1	Sodium intake (mg/day)	58810	2070.02	728.49	1950.90	446.15	8629.67
gramnew1	Bregott on bread	58812	9.40	10.93	5.00	0.00	60.00

gramnew2	Butter on bread	58812	1.19	6.08	0.00	0.00	80.00
gramnew3	Low fat margarine on bread	58812	6.62	9.92	0.56	0.00	60.00
gramnew4	Margarine on bread	58812	0.60	3.35	0.00	0.00	60.00
gramnew5	Butter for cooking	58812	2.60	6.00	0.06	0.00	100.00
gramnew6	Margarine for cooking	58812	8.24	9.69	7.00	0.00	100.00
gramnew7	Oil for cooking	58812	4.53	6.52	2.00	0.00	80.00
gramnew8	Salad dressing with oil	58812	3.09	5.24	1.82	0.00	96.00
gramnew9	Cream, creme fraiche, sour cream	58812	5.51	6.50	3.50	0.00	160.00
gramnew10	Whole grain crisp bread	58812	27.93	21.57	21.00	0.00	104.00
gramnew11	Whole grain soft bread	58812	31.44	34.67	20.52	0.00	264.00
gramnew12	White (soft) bread, thin crisp bread	58812	22.26	27.27	12.78	0.00	298.00
gramnew13	Coffee rolls/buns, rusk	58812	11.94	15.63	5.88	0.00	176.00
gramnew14	Cheese 28%	58812	12.02	13.22	7.20	0.00	96.00
gramnew15	Cheese 10-17%	58812	5.32	9.93	1.52	0.00	84.00
gramnew16	Soft cheese	14073	0.97	3.14	0.05	0.00	72.00
gramnew17	Soft whey cheese	14073	0.84	3.32	0.00	0.00	60.00
gramnew18	Sausage, liver pate on bread	58812	4.80	6.82	2.13	0.00	152.10
gramnew19	Meat on bread	58812	4.57	6.16	2.10	0.00	112.00
gramnew20	Oatflake, whole wheat, rye or barley porridge	58812	35.36	55.11	16.00	0.00	800.60
gramnew21	Rosehip, sweet syrup soup	58812	9.22	21.05	0.53	0.00	700.00
gramnew22	Sour milk, yoghurt (3% fat)	58812	94.67	107.26	39.20	0.00	1200.00
gramnew23	Sour milk, yoghurt (low fat)	58812	53.86	90.71	18.00	0.00	1120.00
gramnew24	Fiber cereals	58812	10.61	13.95	3.60	0.00	180.00
gramnew25	Corn flakes	58812	3.44	5.76	1.12	0.00	88.00
gramnew26	Berries (fresh or frozen)	58812	8.70	14.04	5.20	0.00	260.00
gramnew27	Apple, pear, peach, orange, mandarin and grapefruit	58812	108.80	101.72	80.50	0.00	920.00
gramnew28	Banana	58812	42.35	45.35	37.80	0.00	420.00
gramnew29	Root vegetables and carrot	58812	34.83	44.91	18.00	0.00	416.00
gramnew30	Tomato and cucumber	58812	37.03	42.05	23.40	0.00	380.00
gramnew31	White cabbage, lettuce, lettuce cabbage, spinach, borecole and broccoli	58812	27.37	35.76	17.16	0.00	483.40
gramnew32	Mixed frozen vegetables	14073	7.91	12.60	4.80	0.00	250.00
gramnew33	Boiled or baked potato	58812	115.95	95.14	86.40	0.00	1280.00
gramnew34	Fried potatoes and pommes frites	58812	13.40	16.01	10.72	0.00	600.00
gramnew35	Mashed potato	14073	13.68	14.33	14.00	0.00	300.00
gramnew36	Potato salad	11239	3.67	6.06	0.44	0.00	190.00
gramnew37	Rice	58812	30.25	28.75	19.60	0.00	720.00
gramnew38	Pasta	58812	42.89	36.45	28.00	0.00	750.00
gramnew39	Brown beans and pea soup	58812	10.85	18.96	0.83	0.00	687.50
gramnew40	Blota (broth + bread)	14073	0.69	2.18	0.24	0.00	80.00
gramnew41	Pancake, waffle and Swedish dumpling	58812	23.39	19.09	21.20	0.00	375.00
gramnew42	Pizza	58812	15.63	14.83	12.00	0.00	375.00
gramnew43	Minced meat dishes	58812	20.86	17.23	17.50	0.00	500.00
gramnew44	Meat stew	58812	17.54	15.94	14.00	0.00	700.00
gramnew45	Steak, chop, etc.	58812	15.34	12.82	12.00	0.00	250.00
gramnew46	Bacon	58812	3.74	4.23	2.72	0.00	150.00
gramnew47	Sausage as main dish	58812	11.99	10.76	10.50	0.00	300.00
gramnew48	Hamburger	58812	6.95	6.63	8.80	0.00	300.00
gramnew49	White meat (poultry)	58812	15.13	13.96	12.00	0.00	375.00
gramnew50	Blood based food	14073	7.80	8.60	0.57	0.00	132.30
gramnew51	Liver and kidney	14073	2.26	4.04	0.23	0.00	52.50
gramnew52	Lean fish (e.g. perch, bass, cod)	58812	11.42	10.18	11.20	0.00	275.00
gramnew53	Fatty fish (e.g. herring, whitefish, salmon)	58812	7.47	8.91	4.80	0.00	300.00
gramnew54	Shellfish (e.g. shrimps, scallops)	14073	2.05	2.84	0.21	0.00	69.00
gramnew55	Salty fish	58812	1.09	2.58	0.18	0.00	250.00
gramnew56	Smoked fish/meat	58812	1.05	2.40	0.08	0.00	62.50
gramnew57	Ice cream	58812	6.57	7.62	5.20	0.00	260.00
gramnew58	Sweets	58812	5.67	6.96	3.36	0.00	160.00
gramnew59	Sugar, honey, marmelade and jam	58812	10.92	15.11	4.48	0.00	115.00
gramnew60	Cookies and pastry	58812	8.72	11.86	4.48	0.00	184.00
gramnew61	Chips, popcorn and salted nuts	58812	2.71	3.41	2.16	0.00	87.50
gramnew62	Low fat milk (0.5%)	58812	55.39	129.43	0.00	0.00	1040.00
gramnew63	Milk, sour milk (1.5%)	58812	138.69	170.25	68.40	0.00	1040.00
gramnew64	Milk, sour milk (3%)	58812	25.63	94.19	0.00	0.00	1400.00
gramnew65	Sodas, soft drinks and juice	58812	86.64	127.00	37.33	0.00	2108.00
gramnew66	Brewed (filtered) coffee	58812	307.81	205.50	375.00	0.00	800.00
gramnew67	Boiled coffee	58812	98.47	175.06	0.45	0.00	800.00
gramnew68	Tea	58812	151.81	198.32	90.00	0.00	1000.00
gramnew69	Light beer	58812	26.88	64.05	0.99	0.00	1320.00
gramnew70	Medium beer	58812	21.67	45.75	0.99	0.00	1400.00
gramnew71	Strong beer	58812	19.32	32.86	1.20	0.00	1250.00
gramnew72	Wine	58812	18.88	26.41	18.00	0.00	687.50
gramnew73	Liquor and spirits	58812	2.29	4.46	0.25	0.00	210.00
year2	year2	67738	2001.70	6.72	2002.00	1990.00	2013.00
age2	age2	67738	2356.26	836.30	2490.01	462.25	5012.64
followup	followup	67738	6.33	6.52	10.00	0.00	22.00

Supplementary Table 2. Descriptive statistics of the categorical and ordinal lifestyle variables

Variables	Categories	N of observations
Gender	1= Male	33432
	2= Female	34306
Fasting status	1= 8 hours or more	58925
	2= less than 8 hours	1718
Educatiol level	1= Elementary school + nine-year (compulsory) school	10871
	2= Folk high school equivalent to nine-year (compulsory) school + junior secondary school + girls' school + vocatiol (training) school	13292
	3= Folk high school equivalent to upper secondary school + girls' school equivalent to upper secondary school	24510
	4= University education/college	18533
Work shifts/weekends	1= No	47054
	2= Yes	17048
Long-term sickness	1= No	51628
	2= Yes	10665
Permanent employment	1= No	16674
	2= Yes	41321
Self-employed	1= No	51175
	2= Yes	6056
Overall state of health compared to other your age	1= Worse	2264
	2= About the same	27037
	3= Better	3977
Overall state of health during the last year	1= Poor	727
	2= Fairly poor	3075
	3= Tolerably	12563
	4= Fairly good	31443
	5= Very good	18681
Parents or siblings had a cerebral hemorrhage/thrombosis or cardiac infarction before the age of 60	1= No	52348
	2= Yes	13029
Parents or siblings have diabetes	1= No	52045
	2= Yes	13389
Informed of having high blood pressure	1= No	52024
	2= Yes	14839
Self rate of overall health	1= Poor	425
	2= Fairly good	4086
	3= Good	10194
	4= Very good	9800
	5= Excellent	4286
Self rate of overall health compared to a year ago	1= Much worse than a year ago	314
	2= A little worse than a year ago	2992
	3= About the same	20844
	4= A little better than a year ago	3274
	5= Much better than a year ago	1123
Physical limitation to participate in steneous activities: running, lifting heavy objects, taking part in physically demanding sports	1= No, not limited at all	12253
	2= Yes, a little limited	11548
	3= Yes, very limited	4932
Physical limitation to participate in moderately demanding activities: moving a table, vacuuming, walking in the forest or gardening	1= No, not limited at all	23665
	2= Yes, a little limited	4405
	3= Yes, very limited	657
Physical limitation to participate in moderately demanding activities: lifting or carrying grocery bags	1= No, not limited at all	23192
	2= Yes, a little limited	4597
	3= Yes, very limited	915
Physical limitation to participate in moderately demanding activities: walking up several stairs	1= No, not limited at all	23670
	2= Yes, a little limited	4272
	3= Yes, very limited	771
Physical limitation to participate in moderately demanding activities: bending down or kneeling	1= No, not limited at all	21445
	2= Yes, a little limited	6092
	3= Yes, very limited	1148

Physical limitation to participate in moderately demanding activities: walking more than 2 km	1= No, not limited at all	24638
	2= Yes, a little limited	3077
	3= Yes, very limited	1011
Physical limitation that reduced the normal time spent at work or in other activities during the last four weeks	1= No	25557
	2= Yes	3194
Physical limitation that made you do less than you wanted during the last four weeks	1= No	23073
	2= Yes	5663
Physical limitation that made you not being able to perform certain work tasks or other activities during the last four weeks	1= No	24339
	2= Yes	4285
Physical limitation that limited your ability to perform certain work tasks or other activities during the last four weeks	1= No	24719
	2= Yes	3938
Emotiol problems that made you do less than you wanted during the last four weeks	1= No	25150
	2= Yes	3602
Extent to what your physical and emotiol health disrupted your usual social life during the last four weeks	1= Not al all	22236
	2= A little	3718
	3= Moderately	1970
	4= Much	676
	5= Very much	135
Pain during the last four weeks	1= None	9538
	2= Very little	5698
	3= Little	4369
	4= Moderate	7168
	5= Severe	1740
	6= Very severe	187
How much has the pain during the last four weeks disturbed your normal work?	1= Not at all	16023
	2= A little	6109
	3= Moderately	4519
	4= Much	1641
	5= Very much	344
For how much of the time during the last four weeks have you felt really alert and strong?	1= None of the time	1305
	2= A little of the time	2392
	3= Part of the time	4220
	4= Much of the time	4975
	5= Most of the time	11941
	6= All of the time	3813
For how much of the time during the last four weeks have you felt very nervous?	1= None of the time	21308
	2= A little of the time	5404
	3= Part of the time	1212
	4= Much of the time	525
	5= Most of the time	115
	6= All of the time	131
For how much of the time during the last four weeks have you felt so depressed that nothing could cheer you up?	1= None of the time	24047
	2= A little of the time	3074
	3= Part of the time	949
	4= Much of the time	400
	5= Most of the time	131
	6= All of the time	123
For how much of the time during the last four weeks have you felt calm and serene?	1= None of the time	567
	2= A little of the time	1410
	3= Part of the time	2781
	4= Much of the time	4115
	5= Most of the time	13250
	6= All of the time	6577
For how much of the time during the last four weeks have you felt full of energy?	1= None of the time	1465
	2= A little of the time	2951
	3= Part of the time	4921
	4= Much of the time	6836
	5= Most of the time	9792
	6= All of the time	2697
For how much of the time during the last four weeks have you felt gloomy and sad?	1= None of the time	17496
	2= A little of the time	7904
	3= Part of the time	2087
	4= Much of the time	762

	5= Most of the time	260
	6= All of the time	159
For how much of the time during the last four weeks have you felt worn out?	1= None of the time	11255
	2= A little of the time	9381
	3= Part of the time	4281
	4= Much of the time	2298
	5= Most of the time	986
	6= All of the time	426
For how much of the time during the last four weeks have you felt happy?	1= None of the time	528
	2= A little of the time	1934
	3= Part of the time	3936
	4= Much of the time	5364
	5= Most of the time	12854
	6= All of the time	4076
For how much of the time during the last four weeks have you felt tired?	1= None of the time	3967
	2= A little of the time	11263
	3= Part of the time	6439
	4= Much of the time	4404
	5= Most of the time	1807
	6= All of the time	835
For how much of the time during the last four weeks has your physical health or your emotiol problems limited your ability to interact with others?	1= None of the time	21216
	2= A little of the time	4244
	3= Part of the time	2387
	4= Most of the time	566
	5= All of the time	135
Get sick more often than other people	1= Not at all true	21337
	2= Not very true	4772
	3= Unsure	1689
	4= Mostly true	725
	5= Altogether true	149
As healthy as anyone	1= Not at all true	1257
	2= Not very true	2079
	3= Unsure	2929
	4= Mostly true	9145
	5= Totally true	13178
Worsen in health in the future	1= Not at all true	10253
	2= Not very true	6455
	3= Unsure	8796
	4= Mostly true	2546
	5= Totally true	527
Excellent health	1= Not at all true	1454
	2= Not very true	3089
	3= Unsure	3324
	4= Mostly true	13707
	5= Totally true	7101
Satisfaction with home and family situation	1= Very poor … 7= Excellent	177
		517
		1224
		2577
		5534
		12618
		26220
Satisfaction with accomodation	1= Very poor … 7= Excellent	76
		227
		733
		2017
		4911
		11972
		28974
Satisfaction with work situation	1= Very poor … 7= Excellent	1663
		1833
		3282
		6575
		10748
		12483
		11665
Satisfaction with economy	1= Very poor … 7= Excellent	696
		1250
		2492
		5760

		10019
		14014
		14627
Satisfaction with leisure time	1= Very poor … 7= Excellent	400
		1072
		2246
		4811
		8962
		13802
		17500
Hearing status	1= Very poor … 7= Excellent	342
		1209
		3683
		5482
		9849
		13235
		15099
Vision status	1= Very poor … 7= Excellent	247
		1077
		4857
		10338
		14862
		11287
		6167
Memory status	1= Very poor … 7= Excellent	223
		726
		2457
		6200
		12679
		16961
		9515
Fitness status	1= Very poor … 7= Excellent	880
		2268
		5577
		11384
		14730
		9721
		4272
Appetite status	1= Very poor … 7= Excellent	30
		128
		536
		2181
		5176
		12538
		28271
Mood status	1= Very poor … 7= Excellent	99
		561
		1801
		5137
		10705
		17004
		13520
Energy status	1= Very poor … 7= Excellent	461
		1637
		3623
		7904
		14015
		13971
		7182
Patience status	1= Very poor … 7= Excellent	214
		871
		2597
		6144
		12353
		16252
		10403
Confidence status	1= Very poor … 7= Excellent	253
		830
		2010
		5507
		11794
		17078
		11332
Sleep status	1= Very poor … 7= Excellent	1013

		2227
		4099
		6019
		8995
		11486
		15068
Do you feel important and appreciated outside your home?	1= Very poor … 7= Excellent	246
		521
		1272
		4701
		12002
		18719
		11430
Do you feel important and appreciated in your home?	1= Very poor … 7= Excellent	210
		320
		790
		2730
		6293
		15520
		22667
Number of social contacts with the same interests as you	1= No one	430
	2= 1-2 persons	4065
	3= 3-5 persons	16078
	4= 6-10 persons	19219
	5= 11-15 persons	8049
	6= >15 persons	19218
Number of social interactions during a normal week	1= No one	76
	2= 1-2 persons	2442
	3= 3-5 persons	10961
	4= 6-10 persons	16008
	5= 11-15 persons	9781
	6= >15 persons	27177
Would you say that the number of people that you meet in your everyday life is enough or would you like to meet more or fewer people?	1= Fewer	880
	2= Sufficiently enough	57292
	3= More	9005
Number of friends that can come to your home at any time and feel at home	1= No one	1295
	2= 1-2 persons	7378
	3= 3-5 persons	22167
	4= 6-10 persons	20609
	5= 11-15 persons	6712
	6= >15 persons	8067
Number of people with whom you can speak openly	1= No one	1034
	2= 1-2 persons	11764
	3= 3-5 persons	27277
	4= 6-10 persons	17417
	5= 11-15 persons	4883
	6= >15 persons	4764
Support from others	1= No	2416
	2= Yes, but I do not need it	7804
	3= Yes	57012
Close relationship with anyone	1= No	1489
	2= Not sure	6046
	3= Yes	58833
Receive hugs to comfort and support you	1= No	11107
	2= Yes	54839
People to ask for help apart from the ones at home	1= No	6866
	2= Yes	60111
Participation in associations or voluntary organisations	1= No	24692
	2= Yes	41404
Frequency of engaging in clubs, associations or study circles	1= 1-2 times per year	7817
	2= 1-2 times per month	13988
	3= 1-2 times per week	19533
	4= Every day	1090
Participation in sports or physical exercise associations	1= No	9888
	2= Yes	11235
Participation in study circles	1= No	18026
	2= Yes	3097
Participation in other association	1= No	11371
	2= Yes	9752
High physical demand from job	1= No as good as never	19496

	2= No rarely	15394
	3= Yes sometimes	22570
	4= Yes often	8984
Job demands to work very fast	1= No as good as never	3534
	2= No rarely	12641
	3= Yes sometimes	37509
	4= Yes often	12609
High mental demand from job	1= No as good as never	10791
	2= No rarely	32597
	3= Yes sometimes	15954
	4= Yes often	6477
Enough time for job assignments	1= No as good as never	2332
	2= No rarely	12135
	3= Yes sometimes	26046
	4= Yes often	25480
Contradictory demands in job	1= No as good as never	9620
	2= No rarely	23783
	3= Yes sometimes	26145
	4= Yes often	6262
Learn new things at job	1= No as good as never	1725
	2= No rarely	7664
	3= Yes sometimes	35769
	4= Yes often	20997
Skill demand from job	1= No as good as never	898
	2= No rarely	4041
	3= Yes sometimes	28725
	4= Yes often	32414
Ingenuity or creativity demand from job	1= No as good as never	1059
	2= No rarely	5366
	3= Yes sometimes	29615
	4= Yes often	29948
Repetitive job	1= No as good as never	2181
	2= No rarely	13124
	3= Yes sometimes	24588
	4= Yes often	26281
Control over planning and execution of the workday	1= No as good as never	1260
	2= No rarely	5246
	3= Yes sometimes	22349
	4= Yes often	37448
Control over own work assignment	1= No as good as never	3582
	2= No rarely	13014
	3= Yes sometimes	25840
	4= Yes often	23824
Possibility to speak with colleagues during breaks	1= No, I do not have breaks with colleagues	6909
	2= No, I do not have breaks	1444
	3= Yes, most of the time	23246
	4= Yes, always	32152
Possibility to leave your work for a while to speak with a colleague	1= No, it is totally impossible	3480
	2= Only for urgent matters	6226
	3= Yes, sometimes	19100
	4= Yes, most of the time	34536
Frequent social contacts with colleagues during work	1= Seldom or never	2000
	2= No, I mostly work alone	5685
	3= One or a few times per month	2565
	4= Yes, a lot	53036
Frequncy of social contacts with colleagues during leisure time	1= Seldom or never	16362
	2= One or more times per year	24066
	3= One or more times per month	15604
	4= One or more times per week	6183
Last time a colleague visited you at home	1= I have never been visited by a colleague	7106
	2= More than a year ago	9851
	3= One to twelve months ago	20959
	4= One to four weeks ago	25149
Sedentary or standing work	1= No	47155
	2= Yes	17279
Light but partly physically active work	1= No	52343
	2= Yes	12091
Light and physically active work	1= No	48917
	2= Yes	15517
Sometimes physically straining work	1= No	45793

	2= Yes	18641
Frequency of walking during leisure time	1= Never	2686
	2= 1-2 times a month	9304
	3= 3-4 times a month	12663
	4= 2-3 times a week	24753
	5= Every day	14597
Frequency of cycling during leisure time	1= Never	11228
	2= 1-2 times a month	14079
	3= 3-4 times a month	9981
	4= 2-3 times a week	11996
	5= Every day	6965
Frequency of dancing during leisure time	1= Never	23960
	2= 1-2 times a month	11065
	3= 3-4 times a month	2507
	4= 2-3 times a week	861
	5= Every day	8
Frequency of shoverling snow during leisure time	1= Never	4805
	2= 1-2 times a month	9235
	3= 3-4 times a month	11473
	4= 2-3 times a week	13712
	5= Every day	2340
Frequency of gardening during leisure time	1= Never	4100
	2= 1-2 times a month	8088
	3= 3-4 times a month	10981
	4= 2-3 times a week	16246
	5= Every day	1904
Frequency of hunting or fishing during leisure time	1= Never	14822
	2= 1-2 times a month	12232
	3= 3-4 times a month	8778
	4= 2-3 times a week	4172
	5= Every day	237
Frequency of picking berries or mushrooms during leisure time	1= Never	6806
	2= 1-2 times a month	16907
	3= 3-4 times a month	10809
	4= 2-3 times a week	6018
	5= Every day	569
Changed everyday exercise during the last year	1= Decreased a lot	2951
	2= Decreased somewhat	7701
	3= As before	23940
	4= Increased somewhat	7914
	5= Increased a lot	1293
Everyday exercise satisfaction	1= Not at all	5663
	2= Rather poorly	9456
	3= Partly	20985
	4= Completely	7564
Exercise during the last three months	1= Never	26025
	2= Every now and then- not regularly	15508
	3= 1-2 times/week	11001
	4= 2-3 times/week	9275
	5= More than 3 times/week	3911
If you exercise, change in exercise habits during the last year	1= Decreased a lot	3548
	2= Decreased somewhat	6836
	3= As before	19762
	4= Increased somewhat	6330
	5= Increased a lot	1140
Amount of exercise during the last 12 months	1= Sedentary leisure time	1841
	2= Moderate exercise in leisure time	11787
	3= Moderate, regular exercise in leisure time	5965
	4= Regular exercise	3109
Time spent in a week in moderately steneous activities	1= No time at all	561
	2= Not more than 1 hour per week	2432
	3= 1-3 hours per week	7779
	4= More than 3 hours, but less than 5 hours per week	6016
	5= 5 hours per week or more	5207
Risk of sleeping while sitting and reading	1= None	10470
	2= Little	6914
	3= Moderate	3807
	4= Big	1104
Risk of sleeping while watching TV	1= None	3471

	2= Little	7165
	3= Moderate	7767
	4= Big	4133
Risk of sleeping while sitting ictive in a public place	1= None	12669
	2= Little	6991
	3= Moderate	2278
	4= Big	448
Risk of sleeping as a passenger in a car for one hour without break	1= None	9280
	2= Little	6897
	3= Moderate	4118
	4= Big	2008
Risk of sleeping while lying down resting in the afternoon	1= None	2126
	2= Little	4562
	3= Moderate	7064
	4= Big	8705
Risk of sleeping while sitting still after having lunch	1= None	13910
	2= Little	5657
	3= Moderate	2419
	4= Big	486
Snore during sleep	1= No, never	1664
	2= No, almost never	4386
	3= Yes, sometimes	10744
	4= Yes, almost always	2932
	5= Yes, always	1309
Breath-holds during sleep	1= No, never	11408
	2= No, almost never	965
	3= Yes, sometimes	2601
	4= Yes, almost always	370
	5= Yes, always	370
Teetotaler	1= No	38729
	2= Yes	4295
Feel the need to reduce alcohol consumption	1= No	52545
	2= Yes	7755
Feel uneasy or guilty because of your way of drinking	1= No	34297
	2= Yes	4439
Frequency of alcohol consumption	1= Never	2055
	2= 1 time/month or more seldom	6241
	3= 2-4 times/month	11244
	4= 2-3 times/week	3048
	5= 4 times/week or more	234
Amount of alcohol drunk in a day	1= 0-2 glasses	11731
	2= 3-4 glasses	7925
	3= 5-6 glasses	1767
	4= 7-9 glasses	266
	5= 10 glasses or more	44
Frequency of drinking six or more glasses at the same occasion	1= Never	12674
	2= More seldom than once a month	7378
	3= Every month	1679
	4= Every week	415
	5= Daily or almost daily	7
Times during last year that you felt guilty because of your drinking	1= Never	19967
	2= More seldom than once a month	1962
	3= Every month	129
	4= Every week	39
	5= Daily or almost daily	16
Number of cigarretes smoked per day (in groups)	1=0	42124
	2= 1-4	2189
	3= 5-14	6528
	4= 15-24	2961
	5= >25	238
Number of snuff boxes per week	1= 0	53505
	2= Less than 2	3788
	3= 2 to 4	4660
	4= More than 4 but less than 7	2169
	5= 7 or more	642
Cambridge physical activity index	1= Ictive	9576
	2= Moderatively ictive	19900
	3= Moderatively active	18509
	4= Active	15636
FFQ version	AC00= Optically readable; 66 food items	6653

	AC03= Optically readable; 66 food items	6095
	AC05= Optically readable; 66 food items	16628
	AC11= Optically readable; 66 food items	6254
	AC4= Optically readable; 64 food items	5234
	AC5= Optically readable; 65 food items	2932
	AC6= Optically readable; 65 food items	5422
	APRI= Manually readable; 84 food items	3753
	BAS6= Optically readable; 84 food items	2279
	BASG= Optically readable; 84 food items	4365
	BASN= Optically readable; 84 food items	5751
Average portion size of potatoes/rice/pasta based on photographic illustration of four sizes (smallest to largest)	1= A	3197
	2= B	23748
	3= C	26649
	4= D	5218
Average portion size of meat/fish based on photographic illustration of four sizes (smallest to largest)	1= A	2293
	2= B	26044
	3= C	26307
	4= D	4168
Average portion size of vegetables based on photographic illustration of four sizes (smallest to largest)	1= A	8245
	2= B	16661
	3= C	19955
	4= D	13951
Eat breakfast from 2000	1= No	2997
	2= Yes	30866
Marital status	1= Single	6360
	2= Married/partner	55303
	3= Divorced/separated	4792
	4= Widow/widower	799
Cohabitation	1= Live alone	6822
	2= Only one adult (spouse, partner)	23617
	3= Only children	3092
	4= Adult and children	31935
	5= Other/others	896
Smoking status	1= Non smokers	33051
	2= Smokers	9915
	3= Former smokers	14620
	4= Former occasiol smokers	6680
Snuff status	1= Non-snuff users	45948
	2= Snuff users	11259
	3= Former snuff users	7557
Breakfast habits	1= Not breakfast at all	2086
	2= Only coffee/tea for breakfast	1838
	3= Coffee/tea and wheat buns and rusk for breakfast	1131
	4= Porridge w/o sandwich for breakfast	6493
	5= Gruel w/o sandwich for breakfast	905
Travel to work	1= Passive travel (by car or bus)	37009
	2= Walk	5834
	3= Cycle	13115
	4= Irregular travel mode to work	5960

Supplementary Table 3. Linear mixed model association results for BMI

Description	Group	N	Effect estimate	S.E.	p-value	I^2	Q p-value	marginal R2	conditional R2	R2 rank
Alcohol intake (g/day)	Alcohol	58295.00	-0.08	0.01	0.00	0.00	0.85	0.06	0.84	71.00
Permanent employment	Psychosocial	57370.00	-0.24	0.03	0.00	0.00	0.78	0.07	0.82	4.00
Self-employed	Psychosocial	56724.00	0.03	0.04	0.52	0.00	0.42	-	-	-
Distance to work in kilometers (one way)	Physical activity	56951.00	0.08	0.01	0.00	0.00	0.90	0.06	0.83	21.00
Last time a colleague visited you at home	Psychosocial	62537.00	0.01	0.01	0.26	0.00	0.98	-	-	-
Job demands to work very fast	Psychosocial	65578.00	-0.05	0.01	0.00	0.00	0.92	0.06	0.83	22.00
Frequency of social contacts with colleagues during leisure time	Psychosocial	61690.00	0.01	0.01	0.17	0.66	0.09	-	-	-
High physical demand from job	Physical activity	65728.00	-0.01	0.01	0.43	0.00	0.67	-	-	-
Enough time for job assignments	Psychosocial	65284.00	-0.02	0.01	0.11	0.00	0.99	-	-	-
Control over planning and execution of the workday	Psychosocial	65589.00	0.02	0.01	0.03	0.00	0.63	-	-	-
Ingenuity or creativity demand from job	Psychosocial	65282.00	0.01	0.01	0.53	0.66	0.09	-	-	-
Frequent social contacts with colleagues during work	Psychosocial	62761.00	-0.03	0.01	0.00	0.00	0.60	-	-	-
Contradictory demands in job	Psychosocial	65113.00	0.01	0.01	0.17	0.58	0.12	-	-	-
Possibility to leave your work for a while to speak with a colleague	Psychosocial	62811.00	-0.01	0.01	0.28	0.00	0.37	-	-	-
Learn new things at job	Psychosocial	65442.00	-0.01	0.01	0.40	0.27	0.24	-	-	-
High mental demand from job	Psychosocial	65110.00	-0.02	0.01	0.14	0.00	0.75	-	-	-
Repetitive job	Psychosocial	65463.00	0.03	0.01	0.01	0.52	0.15	-	-	-
Skill demand from job	Psychosocial	65370.00	-0.02	0.01	0.09	0.00	0.67	-	-	-
Possibility to speak with colleagues during breaks	Psychosocial	63211.00	-0.07	0.01	0.00	0.00	0.84	0.06	0.83	37.00
Control over own work assignment	Psychosocial	65550.00	0.03	0.01	0.00	0.00	0.44	-	-	-
Vitamin C intake (mg/day)	Nutrients	58295.00	-0.09	0.01	0.00	0.48	0.17	0.06	0.84	77.00
Vitamin B12 intake (ug/day)	Nutrients	58295.00	0.10	0.01	0.00	0.00	0.97	0.06	0.83	65.00
Vitamin B2 intake (ug/day)	Nutrients	58295.00	0.03	0.01	0.00	0.00	0.95	0.06	0.84	120.00
Vitamin B6 intake (mg/day)	Nutrients	58295.00	0.07	0.01	0.00	0.25	0.25	0.06	0.83	80.00
Informed of having high blood pressure	General health	66202.00	0.67	0.03	0.00	0.00	0.99	0.07	0.83	8.00
Beta-sitostanol intake (mg/day)	Nutrients	58295.00	-0.06	0.01	0.00	0.00	0.34	0.06	0.84	106.00
Beta-sitosterol intake (mg/day)	Nutrients	58295.00	-0.09	0.01	0.00	0.00	0.34	0.06	0.84	82.00
Marital status: Single vs Married/partner	Social	61061.00	-0.05	0.04	0.29	0.09	0.29	-	-	-
Marital status: Single vs Divorced/separated	Social	11005.00	-0.45	0.08	0.00	0.00	0.78	0.07	0.81	6.00
Marital status: Single vs Widow/widower	Social	7042.00	-0.22	0.19	0.24	0.00	0.90	-	-	-
Campestanol intake (mg/day)	Nutrients	58295.00	-0.05	0.01	0.00	0.00	0.35	0.06	0.84	105.00
Campesterol intake (mg/day)	Nutrients	58295.00	-0.01	0.01	0.37	0.00	0.49	-	-	-
Parents or siblings have diabetes	General health	64826.00	0.40	0.03	0.00	0.20	0.26	0.06	0.83	19.00
Disaccharides intake (g/day)	Nutrients	58295.00	-0.03	0.01	0.01	0.44	0.18	-	-	-
Vitamin D intake (ug/day)	Nutrients	58295.00	0.14	0.01	0.00	0.00	0.98	0.06	0.83	47.00
Total energy intake (kcal/day)	Nutrients	58295.00	0.07	0.01	0.00	0.62	0.10	0.06	0.84	125.00
Formic acid intaje (g/day)	Nutrients	58295.00	-0.01	0.01	0.32	0.00	0.92	-	-	-
Pentadecanoic acid intake (g/day)	Nutrients	58295.00	-0.05	0.01	0.00	0.00	0.50	0.06	0.84	117.00

Palmitic acid intake (g/day)	Nutrients	58295.00	0.08	0.01	0.00	0.00	0.57	0.06	0.84	75.00
Heptadecanoic acid intake (g/day)	Nutrients	58295.00	-0.05	0.01	0.00	0.00	0.50	0.06	0.84	116.00
Linoleic acid intake (g/day)	Nutrients	58295.00	0.01	0.01	0.61	0.00	0.61	-	-	-
Linolenic acid intake (g/day)	Nutrients	58295.00	0.02	0.01	0.13	0.00	0.43	-	-	-
Arachidonic acid (ARA) intake (g/day)	Nutrients	58293.00	0.16	0.01	0.00	0.07	0.30	0.06	0.83	41.00
Eicosapentaenoic acid (EPA) intake (g/day)	Nutrients	58294.00	0.07	0.01	0.00	0.00	0.44	0.06	0.84	88.00
Docosahexaenoic acid (DHA) intake (g/day)	Nutrients	58295.00	0.06	0.01	0.00	0.00	0.40	0.06	0.84	93.00
Fat intake (g/day)	Nutrients	58295.00	0.09	0.01	0.00	0.00	0.54	0.06	0.84	73.00
Fibre intake (g/day)	Nutrients	58295.00	-0.19	0.01	0.00	0.00	0.53	0.06	0.84	42.00
Folic acid intake (ug/day)	Nutrients	58295.00	-0.14	0.01	0.00	0.27	0.24	0.06	0.84	60.00
Phosphate intake (mg/day)	Nutrients	58295.00	0.03	0.01	0.00	0.00	0.98	-	-	-
Whole grain intake (g/day)	Food	58295.00	-0.11	0.01	0.00	0.30	0.23	0.06	0.84	63.00
Travel to work: Walk to work vs passive travel to work	Physical activity	42426.00	-0.27	0.04	0.00	0.00	0.34	0.06	0.82	30.00
Travel to work: Cycle to work vs passive travel to work	Physical activity	49670.00	-0.56	0.03	0.00	0.00	0.87	0.07	0.83	7.00
Travel to work: Irregular travel mode to work vs passive travel to work	Physical activity	42564.00	-0.16	0.04	0.00	0.19	0.27	0.06	0.83	18.00
Time spent in a week in moderately steneous activities	Physical activity	21881.00	-0.56	0.03		0.00	0.98	-	-	-
Sedentary or standing work	Physical activity	63808.00	0.14	0.02	0.00	0.65	0.09	0.06	0.83	29.00
Light but partly physically active work	Physical activity	63808.00	0.06	0.02	0.01	0.00	0.48	-	-	-
Light and physically active work	Physical activity	63808.00	-0.13	0.02	0.00	0.00	0.56	0.06	0.83	28.00
Sometimes physically straining work	Physical activity	63808.00	-0.04	0.02	0.07	0.00	0.94	-	-	-
Frequency of walking during leisure time	Physical activity	63385.00	-0.17	0.01	0.00	0.00	0.74	0.06	0.83	20.00
Frequency of cycling during leisure time	Physical activity	53763.00	-0.17	0.01	0.00	0.66	0.09	0.06	0.82	16.00
Frequency of dancing during leisure time	Physical activity	37969.00	-0.13	0.02	0.00	0.00	0.57	0.06	0.82	33.00
Frequency of shoveling snow during leisure time	Physical activity	41082.00	-0.02	0.02	0.21	0.05	0.31	-	-	-
Frequency of gardening during leisure time	Physical activity	40839.00	-0.05	0.01	0.00	0.64	0.10	-	-	-
Frequency of hunting or fishing during leisure time	Physical activity	39785.00	0.11	0.02	0.00	0.00	0.56	0.06	0.83	27.00
Frequency of picking berries or mushrooms during leisure time	Physical activity	40647.00	-0.13	0.02	0.00	0.18	0.27	0.06	0.83	35.00
Changed everyday exercise during the last year	Physical activity	43264.00	-0.14	0.01	0.00	0.00	0.66	0.06	0.83	25.00
Everyday exercise satisfaction	Physical activity	43136.00	-0.36	0.01	0.00	0.00	0.49	0.07	0.83	5.00
Exercise during the last three months	Physical activity	65068.00	-0.24	0.01	0.00	0.24	0.25	0.06	0.83	12.00
If you exercise, change in exercise habits during the last year	Physical activity	37199.00	-0.15	0.01	0.00	0.00	0.59	0.06	0.83	17.00
Amount of exercise during the last 12 months	Physical activity	22578.00	-0.74	0.03		0.00	0.48	-	-	-
Bregott on bread	Food	58295.00	-0.01	0.01	0.18	0.00	0.82	-	-	-
Whole grain crisp bread	Food	58295.00	-0.05	0.01	0.00	0.35	0.21	-	-	-
Whole grain soft bread	Food	58295.00	-0.03	0.01	0.00	0.00	0.68	-	-	-
White (soft) bread, thin crisp bread	Food	58295.00	0.02	0.01	0.09	0.21	0.26	-	-	-
Coffee rolls/buns, rusk	Food	58295.00	-0.03	0.01	0.00	0.00	0.50	-	-	-

Cheese 28%	Food	58295.00	-0.02	0.01	0.09	0.00	0.96	-	-	-
Cheese 10-17%	Food	58295.00	-0.01	0.01	0.42	0.00	0.35	-	-	-
Soft cheese	Food	13823.00	0.10	0.03		0.00	0.52	-	-	-
Soft whey cheese	Food	13823.00	-0.10	0.03		0.20	0.26	-	-	-
Sausage, liver pate on bread	Food	58295.00	0.07	0.01	0.00	0.00	0.54	0.06	0.84	90.00
Meat on bread	Food	58295.00	0.01	0.01	0.33	0.00	0.82	-	-	-
Butter on bread	Food	58295.00	-0.01	0.01	0.28	0.63	0.10	-	-	-
Oatflake, whole wheat, rye or barley porridge	Food	58295.00	-0.09	0.01	0.00	0.00	0.89	0.06	0.84	72.00
Rosehip, sweet syrup soup	Food	58295.00	-0.02	0.01	0.03	0.61	0.11	-	-	-
Sour milk, yoghurt (3% fat)	Food	58295.00	-0.07	0.01	0.00	0.00	0.99	0.06	0.84	91.00
Sour milk, yoghurt (low fat)	Food	58295.00	-0.01	0.01	0.35	0.00	0.93	-	-	-
Fiber cereals	Food	58295.00	-0.09	0.01	0.00	0.00	0.38	0.06	0.84	68.00
Corn flakes	Food	58295.00	0.01	0.01	0.35	0.00	0.89	-	-	-
Berries (fresh or frozen)	Food	58295.00	-0.06	0.01	0.00	0.00	0.55	0.06	0.84	103.00
Apple, pear, peach, orange, mandarin and grapefruit	Food	58295.00	-0.07	0.01	0.00	0.00	0.70	0.06	0.84	102.00
Ba	Food	58295.00	-0.05	0.01	0.00	0.67	0.08	0.06	0.84	108.00
Root vegetables and carrot	Food	58295.00	-0.09	0.01	0.00	0.00	0.90	0.06	0.84	94.00
Low fat margarine on bread	Food	58295.00	0.04	0.01	0.00	0.00	0.35	0.06	0.84	115.00
Tomato and cucumber	Food	58295.00	-0.08	0.01	0.00	0.00	0.58	0.06	0.84	97.00
White cabbage, lettuce, lettuce cabbage, spich, borecole and broccoli	Food	58295.00	-0.09	0.01	0.00	0.40	0.20	0.06	0.84	89.00
Mixed frozen vegetables	Food	13823.00	0.21	0.03		0.00	0.75	0.05	0.74	129.00
Boiled or baked potato	Food	58295.00	0.06	0.01	0.00	0.00	0.37	0.06	0.84	98.00
Fried potatoes and pommes frites	Food	58295.00	0.07	0.01	0.00	0.00	0.78	0.06	0.84	84.00
Mashed potato	Food	13823.00	0.22	0.03		0.27	0.24	0.05	0.75	127.00
Potato salad	Food	11113.00	0.03	0.03	0.40	0.35	0.21	-	-	-
Rice	Food	58295.00	0.01	0.01	0.50	0.79	0.03	-	-	-
Pasta	Food	58295.00	0.04	0.01	0.00	0.00	0.56	0.06	0.84	124.00
Brown beans and pea soup	Food	58295.00	-0.05	0.01	0.00	0.00	0.81	0.06	0.84	114.00
Margarine on bread	Food	58295.00	0.00	0.01	0.96	0.00	0.97	-	-	-
Blota (broth + bread)	Food	13823.00	0.06	0.03		0.00	0.91	-	-	-
Pancake, waffle and Swedish dumpling	Food	58295.00	0.07	0.01	0.00	0.00	0.46	0.06	0.84	83.00
Pizza	Food	58295.00	0.06	0.01	0.00	0.00	0.39	0.06	0.84	100.00
Minced meat dishes	Food	58295.00	0.10	0.01	0.00	0.73	0.06	0.06	0.83	62.00
Meat stew	Food	58295.00	0.11	0.01	0.00	0.00	0.46	0.06	0.83	56.00
Steak, chop, etc.	Food	58295.00	0.12	0.01	0.00	0.68	0.08	0.06	0.83	55.00
Bacon	Food	58295.00	0.11	0.01	0.00	0.78	0.03	0.06	0.84	58.00
Sausage as main dish	Food	58295.00	0.14	0.01	0.00	0.78	0.03	0.06	0.84	45.00
Hamburger	Food	58295.00	0.10	0.01	0.00	0.88	0.00	0.06	0.84	69.00
White meat (poultry)	Food	58295.00	0.08	0.01	0.00	0.08	0.30	0.06	0.83	87.00
Butter for cooking	Food	58295.00	-0.01	0.01	0.49	0.00	0.81	-	-	-
Blood based food	Food	13823.00	0.01	0.03		0.00	0.65	-	-	-
Liver and kidney	Food	13823.00	0.05	0.03		0.00	0.69	-	-	-
Lean fish (e.g. perch, bass, cod)	Food	58295.00	0.03	0.01	0.01	0.00	0.72	-	-	-
Fatty fish (e.g. herring, whitefish, salmon)	Food	58295.00	0.03	0.01	0.02	0.00	0.48	-	-	-
Shellfish (e.g. shrimps, scallops)	Food	13823.00	0.06	0.03		0.00	0.70	-	-	-
Salty fish	Food	58295.00	0.07	0.01	0.00	0.23	0.25	0.06	0.84	85.00
Smoked fish/meat	Food	58295.00	0.02	0.01	0.02	0.00	0.51	-	-	-
Ice cream	Food	58295.00	0.02	0.01	0.10	0.00	0.79	-	-	-
Sweets	Food	58295.00	0.02	0.01	0.15	0.00	0.87	-	-	-
Sugar, honey, marmelade and jam	Food	58295.00	-0.05	0.01	0.00	0.00	0.34	0.06	0.84	113.00
Margarine for cooking	Food	58295.00	0.07	0.01	0.00	0.00	0.69	0.06	0.84	95.00
Cookies and pastry	Food	58295.00	-0.02	0.01	0.09	0.00	0.53	-	-	-
Chips, popcorn and salted nuts	Food	58295.00	0.00	0.01	0.89	0.00	0.97	-	-	-
Low fat milk (0.5%)	Beverage	58295.00	0.08	0.01	0.00	0.00	0.95	0.06	0.83	79.00
Milk, sour milk (1.5%)	Beverage	58295.00	0.03	0.01	0.00	0.00	0.39	-	-	-
Milk, sour milk (3%)	Beverage	58295.00	-0.04	0.01	0.00	0.00	0.63	0.06	0.84	123.00
Sodas, soft drinks and juice	Beverage	58295.00	0.02	0.01	0.10	0.83	0.02	-	-	-
Brewed (filtered) coffee	Beverage	58295.00	-0.06	0.01	0.00	0.00	0.36	0.06	0.84	104.00

Boiled coffee	Beverage	58295.00	0.07	0.01	0.00	0.84	0.01	0.06	0.84	76.00
Tea	Beverage	58295.00	-0.10	0.01	0.00	0.00	0.79	0.06	0.83	61.00
Light beer	Alcohol	58295.00	-0.03	0.01	0.01	0.00	0.55	-	-	-
Oil for cooking	Food	58295.00	-0.01	0.01	0.29	0.00	0.37	-	-	-
Medium beer	Alcohol	58295.00	-0.05	0.01	0.00	0.00	0.56	0.06	0.84	109.00
Strong beer	Alcohol	58295.00	0.01	0.01	0.29	0.00	0.45	-	-	-
Wine	Alcohol	58295.00	-0.12	0.01	0.00	0.00	0.36	0.06	0.84	53.00
Liquor and spirits	Alcohol	58295.00	0.05	0.01	0.00	0.00	0.66	0.06	0.84	112.00
Salad dressing with oil	Food	58295.00	-0.08	0.01	0.00	0.00	0.96	0.06	0.84	81.00
Cream, creme fraiche, sour cream	Food	58295.00	-0.03	0.01	0.01	0.00	0.78	-	-	-
Average portion size of vegetables based on photographic illustration of four sizes (smallest to largest)	Food	58295.00	-0.07	0.01	0.00	0.00	0.47	0.06	0.84	48.00
Overall state of health during the last year	General health	65859.00	-0.26	0.01	0.00	0.00	0.93	0.07	0.83	10.00
Overall state of health compared to others your age	General health	32888.00	-0.37	0.02	0.00	0.00	0.67	0.07	0.83	11.00
Parents or siblings had a cerebral hemorrhage/thrombosis or cardiac infarction before the age of 60	General health	64771.00	0.14	0.03	0.00	0.00	0.87	0.06	0.83	23.00
Teetotaler	Alcohol	42541.00	0.15	0.06	0.01	0.00	0.95	-	-	-
Feel the need to reduce alcohol consumption	Alcohol	59795.00	0.03	0.03	0.41	0.67	0.08	-	-	-
Feel uneasy or guilty because of your way of drinking	Alcohol	38346.00	-0.06	0.05	0.22	0.00	0.98	-	-	-
Frequency of alcohol consumption	Alcohol	22695.00	-0.34	0.03		0.00	0.68	-	-	-
Amount of alcohol drunk in a day	Alcohol	21620.00	0.45	0.03		0.00	0.80	-	-	-
Frequency of drinking six or more glasses at the same occasion	Alcohol	22039.00	0.37	0.03		0.00	0.39	-	-	-
Times during last year that you felt guilty because of your drinking	Alcohol	22004.00	-0.03	0.03		0.00	0.65	-	-	-
Iron intake (mg/day)	Nutrients	58295.00	0.01	0.01	0.24	0.64	0.10	-	-	-
Iodine intake (ug/day)	Nutrients	58295.00	0.03	0.01	0.00	0.17	0.27	-	-	-
Calcium intake (mg/day)	Nutrients	58295.00	-0.02	0.01	0.13	0.00	0.36	-	-	-
Potassium intake (mg/day)	Nutrients	58295.00	0.00	0.01	0.93	0.00	0.57	-	-	-
Beta-carotene intake (mg/day)	Nutrients	58295.00	-0.09	0.01	0.00	0.00	0.96	0.06	0.84	96.00
Cholesterol intake (g/day)	Nutrients	58295.00	0.11	0.01	0.00	0.52	0.15	0.06	0.84	57.00
Carbohydrates intake (g/day)	Nutrients	58295.00	-0.11	0.01	0.00	0.00	0.34	0.06	0.84	64.00
Average portion size of meat/fish based on photographic illustration of four sizes (smallest to largest)	Food	58295.00	0.21	0.01	0.00	0.76	0.04	0.06	0.84	36.00
Breakfast habits: Only coffee/tea for breakfast vs not breakfast at all	Food	3893.00	-0.33	0.13	0.01	0.80	0.02	-	-	-
Breakfast habits: Coffee/tea and wheat buns or rusk for breakfast vs not breakfast at all	Food	3190.00	-0.84	0.15	0.00	0.00	0.94	0.05	0.78	126.00
Breakfast habits: Porridge w/o sandwich for breakfast vs not breakfast at all	Food	8515.00	-1.03	0.11	0.00	0.00	0.46	0.05	0.82	130.00
Breakfast habits: Gruel w/o sandwich for breakfast vs not breakfast at all	Food	2963.00	-1.70	0.19	0.00	0.00	0.73	0.09	0.87	1.00
Eat breakfast from 2000	Food	33669.00	-0.47	0.07	0.00	0.62	0.10	0.03	0.85	153.00
Enterodiol intake (ug/day)	Nutrients	58295.00	0.04	0.01	0.00	0.00	0.75	0.06	0.84	119.00

Enterolactone intake (ug/day)	Nutrients	58295.00	-0.01	0.01	0.46	0.00	0.77	-	-	-
Equol intake (ug/day)	Nutrients	58295.00	0.05	0.01	0.00	0.00	0.97	0.06	0.83	101.00
Lariciresinol intake (ug/day)	Nutrients	58295.00	-0.14	0.01	0.00	0.47	0.17	0.06	0.84	52.00
Matairesinol intake (ug/day)	Nutrients	58295.00	-0.11	0.01	0.00	0.00	0.53	0.06	0.84	59.00
Medioresinol intake (ug/day)	Nutrients	58295.00	-0.05	0.01	0.00	0.00	0.36	0.06	0.84	111.00
Pinoresinol intake (ug/day)	Nutrients	58295.00	-0.14	0.01	0.00	0.34	0.22	0.06	0.84	51.00
Secoisolariciresinol intake (ug/day)	Nutrients	58295.00	-0.17	0.01	0.00	0.00	0.72	0.06	0.84	44.00
Sum of all ligns intake (ug/day)	Nutrients	58295.00	-0.10	0.01	0.00	0.00	0.34	0.06	0.84	70.00
Syringaresinol intake (ug/day)	Nutrients	58295.00	-0.07	0.01	0.00	0.00	0.49	0.06	0.84	92.00
Sum of Lariciresinol, Matairesinol, Pinoresinol, Secoisolariciresinol intake (ug/day)	Nutrients	58295.00	-0.16	0.01	0.00	0.38	0.20	0.06	0.84	46.00
Satisfaction with home and family situation	Psychosocial	48573.00	0.01	0.01	0.36	0.14	0.28	-	-	-
Appetite status	Psychosocial	48563.00	0.04	0.01	0.00	0.00	0.77	-	-	-
Mood status	Psychosocial	48528.00	-0.05	0.01	0.00	0.00	0.90	0.04	0.84	137.00
Energy status	Psychosocial	48497.00	-0.32	0.01	0.00	0.00	0.55	0.04	0.84	131.00
Patience status	Psychosocial	48536.00	-0.06	0.01	0.00	0.00	0.54	0.04	0.84	138.00
Confidence status	Psychosocial	48510.00	-0.02	0.01	0.14	0.00	0.95	-	-	-
Sleep status	Sleep	48606.00	-0.05	0.01	0.00	0.00	0.63	0.04	0.84	139.00
Do you feel important and appreciated outside your home?	Psychosocial	48598.00	-0.02	0.01	0.18	0.00	0.74	-	-	-
Do you feel important and appreciated in your home?	Psychosocial	48239.00	-0.03	0.01	0.03	0.84	0.01	-	-	-
Satisfaction with accomodation	Psychosocial	48614.00	0.02	0.01	0.18	0.00	0.88	-	-	-
Satisfaction with work situation	Psychosocial	47966.00	-0.04	0.01	0.00	0.20	0.26	-	-	-
Satisfaction with economy	Psychosocial	48562.00	-0.06	0.01	0.00	0.00	1.00	0.04	0.84	135.00
Satisfaction with leisure time	Psychosocial	48499.00	-0.12	0.01	0.00	0.00	0.82	0.04	0.84	133.00
Hearing status	General health	48597.00	-0.05	0.02	0.00	0.00	0.92	0.04	0.84	136.00
Vision status	General health	48536.00	0.00	0.01	0.99	0.00	0.62	-	-	-
Memory status	Psychosocial	48463.00	0.01	0.01	0.31	0.00	0.48	-	-	-
Fitness status	Physical activity	48532.00	-0.86	0.01	0.00	0.00	0.88	0.09	0.84	2.00
Magnesium intake (mg/day)	Nutrients	58295.00	-0.08	0.01	0.00	0.07	0.30	0.06	0.84	107.00
Saturated fat intake (g/day)	Nutrients	58295.00	0.04	0.01	0.00	0.00	0.91	0.06	0.84	121.00
Monounsaturated fat intake (g/day)	Nutrients	58295.00	0.14	0.01	0.00	0.52	0.15	0.06	0.84	49.00
Monosaccharides intake (g/day)	Nutrients	58295.00	-0.12	0.01	0.00	0.00	0.69	0.06	0.84	66.00
Sodium intake (mg/day)	Nutrients	58293.00	0.15	0.01	0.00	0.48	0.17	0.06	0.83	40.00
Vitamin B3 intake (mg/day)	Nutrients	58295.00	0.15	0.01	0.00	0.66	0.09	0.06	0.83	43.00
Cambridge physical activity index	Physical activity	63007.00	-0.20	0.01	0.00	0.67	0.08	0.06	0.83	15.00
Polyunsaturated fat intake (g/day)	Nutrients	58295.00	0.04	0.01	0.00	0.00	0.82	0.06	0.84	122.00
Average portion size of potatoes/rice/pasta based on photographic illustration of four sizes (smallest to largest)	Food	58295.00	0.18	0.01	0.00	0.00	0.91	0.06	0.84	38.00
Total protein intake (g/day)	Nutrients	58295.00	0.13	0.01	0.00	0.64	0.10	0.06	0.83	50.00
Animal based protein intake (g/day)	Nutrients	58295.00	0.16	0.01	0.00	0.65	0.09	0.06	0.83	39.00
Plant based protein intake (g/day)	Nutrients	58295.00	-0.13	0.01	0.00	0.00	0.47	0.06	0.84	54.00
Vitamin A intake (mg/day)	Nutrients	58295.00	0.05	0.01	0.00	0.00	0.37	0.06	0.84	118.00
Sucrose intake (g/day)	Nutrients	58295.00	-0.07	0.01	0.00	0.00	0.62	0.06	0.84	86.00

Cohabitation: Live alone vs Only one adult (spouse, partner)	Social	30206.00	0.00	0.05	0.98	0.26	0.24	-	-	-
Cohabitation: Live alone vs Only children	Social	9821.00	-0.15	0.08	0.07	0.00	0.44	-	-	-
Cohabitation: Live alone vs Adult and children	Social	38384.00	0.01	0.05	0.80	0.45	0.18	-	-	-
Cohabitation: Live alone vs Other/others	Social	7607.00	0.14	0.13	0.31	0.63	0.10	-	-	-
Selenium intake (ug/day)	Nutrients	58295.00	0.09	0.01	0.00	0.00	0.71	0.06	0.83	67.00
Self rate of overall health	General health	28631.00	-0.79	0.02	0.00	0.00	0.62	0.06	0.81	32.00
For how much of the time during the last four weeks has your physical health or your emotiol problems limited your ability to interact with others?	General health	28393.00	0.23	0.02	0.00	0.00	0.51	0.03	0.80	157.00
Get sick more often than other people	General health	28516.00	0.34	0.02	0.00	0.00	0.63	0.03	0.81	150.00
As healthy as anyone	General health	28432.00	-0.47	0.02	0.00	0.00	0.63	0.04	0.81	141.00
Worsen in health in the future	General health	28422.00	0.28	0.02	0.00	0.00	0.86	0.03	0.80	154.00
Excellent health	General health	28519.00	-0.69	0.02	0.00	0.00	0.33	0.05	0.80	128.00
Self rate of overall health compared to a year ago	General health	28392.00	-0.15	0.02	0.00	0.14	0.28	0.03	0.81	162.00
Physical limitation to participate in steneous activities: running, lifting heavy objects, taking part in physically demanding sports	General health	28579.00	0.85	0.02	0.00	0.00	0.38	0.06	0.80	14.00
Physical limitation to participate in moderately demanding activities: moving a table, vacuuming, walking in the forest or gardening	General health	28569.00	0.51	0.02	0.00	0.84	0.01	0.04	0.81	134.00
Physical limitation to participate in moderately demanding activities: lifting or carrying grocery bags	General health	28548.00	0.28	0.02	0.00	0.00	0.41	0.03	0.81	155.00
Physical limitation to participate in moderately demanding activities: walking up several stairs	General health	28559.00	0.86	0.02	0.00	0.00	0.41	0.07	0.81	9.00
Physical limitation to participate in moderately demanding activities: bending down or kneeling	General health	28527.00	0.95	0.02	0.00	0.06	0.30	0.08	0.80	3.00
Physical limitation to participate in moderately demanding activities: walking more than two km	General health	28569.00	0.74	0.02	0.00	0.07	0.30	0.06	0.80	78.00
Physical limitation that reduced the normal time spent at work or in other activities during the last four weeks	General health	28595.00	0.68	0.07	0.00	0.28	0.24	0.03	0.80	159.00
Physical limitation that made you do less than you wanted during the last four weeks	General health	28579.00	1.08	0.06	0.00	0.48	0.17	0.04	0.80	145.00
Physical limitation that made you not being able to perform certain work tasks or other activities during the last four weeks	General health	28468.00	0.94	0.07	0.00	0.46	0.18	0.03	0.81	151.00

Physical limitation that limited your ability to perform certain work tasks or other activities during the last four weeks	General health	28501.00	0.93	0.07	0.00	0.56	0.13	0.03	0.80	152.00
Emotiol problems that made you do less than you wanted during the last four weeks	General health	28595.00	0.66	0.07	0.00	0.00	0.64	0.03	0.80	158.00
Extent to what your physical and emotiol health disrupted your usual social life during the last four weeks	General health	28576.00	0.25	0.02	0.00	0.69	0.07	0.03	0.80	156.00
Pain during the last four weeks	General health	28538.00	0.49	0.02	0.00	0.00	0.96	0.04	0.80	140.00
How much has the pain during the last four weeks disturbed your normal work?	General health	28476.00	0.46	0.02	0.00	0.00	1.00	0.04	0.80	144.00
For how much of the time during the last four weeks have you felt really alert and strong?	General health	28488.00	-0.45	0.02	0.00	0.00	0.92	0.04	0.81	142.00
For how much of the time during the last four weeks have you felt very nervous?	General health	28535.00	0.04	0.02	0.07	0.60	0.11	-	-	-
For how much of the time during the last four weeks have you felt so depressed that nothing could cheer you up?	General health	28565.00	0.14	0.02	0.00	0.00	0.59	0.03	0.81	163.00
For how much of the time during the last four weeks have you felt calm and serene?	General health	28541.00	-0.10	0.02	0.00	0.00	0.87	0.03	0.81	164.00
For how much of the time during the last four weeks have you felt full of energy?	General health	28505.00	-0.39	0.02	0.00	0.15	0.28	0.03	0.81	148.00
For how much of the time during the last four weeks have you felt gloomy and sad?	General health	28513.00	0.17	0.02	0.00	0.00	0.89	0.03	0.81	160.00
For how much of the time during the last four weeks have you felt worn out?	General health	28470.00	0.38	0.02	0.00	0.00	0.79	0.03	0.81	147.00
For how much of the time during the last four weeks have you felt happy?	General health	28532.00	-0.15	0.02	0.00	0.00	0.71	0.03	0.81	161.00
For how much of the time during the last four weeks have you felt tired?	General health	28557.00	0.36	0.02	0.00	0.00	0.45	0.03	0.80	149.00
Long-term sickness	General health	61894.00	0.37	0.03	0.00	0.00	0.82	0.06	0.83	34.00
Work shifts/weekends	Psychosocial	63530.00	0.12	0.03	0.00	0.00	0.67	0.06	0.83	31.00
Risk of sleeping while sitting and reading	Sleep	22177.00	0.08	0.03		0.00	0.33	-	-	-
Risk of sleeping while watching TV	Sleep	22415.00	0.24	0.03		0.52	0.15	-	-	-
Risk of sleeping while sitting ictive in a public place	Sleep	22266.00	0.13	0.03		0.75	0.05	-	-	-
Risk of sleeping as a passenger in a car for one hour without break	Sleep	22183.00	0.08	0.03		0.00	0.47	-	-	-
Risk of sleeping while lying down resting in the afternoon	Sleep	22336.00	0.12	0.03		0.00	0.77	-	-	-
Risk of sleeping while sitting still after having lunch	Sleep	22350.00	0.16	0.03		0.00	0.66	-	-	-
Snore during sleep	Sleep	20925.00	1.03	0.03		0.00	0.85	-	-	-
Breath-holds during sleep	Sleep	15639.00	0.73	0.03		0.60	0.11	-	-	-

Number of cigarretes smoked per day (in groups)	Tobacco use	53516.00	0.05	0.02	0.00	0.49	0.16	-	-	-
Years smoking	Tobacco use	57869.00	0.08	0.02	0.00	0.43	0.18	-	-	-
Grams of tobacco smoked per week	Tobacco use	41780.00	-0.04	0.02	0.02	0.78	0.03	-	-	-
Number of cigarretes smoked per day	Tobacco use	42809.00	-0.10	0.02	0.00	0.00	0.67	0.06	0.84	13.00
Number of cigars smoked per day	Tobacco use	41744.00	-0.01	0.02	0.76	0.00	0.75	-	-	-
Smoking status: Smokers vs non-smokers	Tobacco use	42499.00	-0.12	0.06	0.03	0.00	0.43	-	-	-
Smoking status: Former smokers vs non-smokers	Tobacco use	47161.00	0.36	0.05	0.00	0.30	0.23	0.06	0.84	26.00
Smoking status: Former occasiol smokers vs non-smokers	Tobacco use	39339.00	-0.08	0.05	0.10	0.00	0.65	-	-	-
Number of snuff boxes per week	Tobacco use	64065.00	0.00	0.01	0.76	0.00	0.98	-	-	-
Snuff status: Snuff users vs non-snuff users	Tobacco use	56571.00	0.11	0.05	0.02	0.54	0.14	-	-	-
Snuff status: Former snuff users vs non-snuff users	Tobacco use	52930.00	0.19	0.05	0.00	0.52	0.15	-	-	-
Years using snuff	Tobacco use	62318.00	0.11	0.02	0.00	0.00	0.65	0.06	0.83	24.00
Participation in associations or voluntary organisations	Social	65490.00	-0.03	0.02	0.12	0.84	0.01	-	-	-
Participation in sports or physical exercise associations	Social	21027.00	-0.61	0.05	0.00	0.00	0.88	0.04	0.85	132.00
Participation in study circles	Social	21027.00	0.33	0.07	0.00	0.00	0.37	0.04	0.85	146.00
Participation in other association	Social	21027.00	0.40	0.05	0.00	0.32	0.22	0.04	0.85	143.00
People to ask for help apart from the ones at home	Social	66248.00	-0.02	0.03	0.49	0.66	0.09	-	-	-
Number of friends that can come to your home at any time and feel at home	Social	65618.00	0.03	0.01	0.00	0.00	0.42	-	-	-
Number of social contacts with the same interests as you	Social	66329.00	0.02	0.01	0.03	0.31	0.23	-	-	-
Would you say that the number of people that you meet in your everyday life is enough or would you like to meet more or fewer people?	Social	66441.00	-0.03	0.01	0.00	0.52	0.15	-	-	-
Close relationship with anyone	Social	65759.00	0.00	0.01	0.79	0.00	0.37	-	-	-
Frequency of engaging in clubs, associations or study circles	Social	41973.00	0.00	0.01	0.80	0.00	0.68	-	-	-
Number of social interactions during a normal week	Social	65825.00	-0.02	0.01	0.12	0.41	0.19	-	-	-
Support from others	Social	66493.00	-0.02	0.01	0.01	0.07	0.30	-	-	-
Number of people with whom you can speak openly	Social	66405.00	0.01	0.01	0.36	0.28	0.24	-	-	-
Receive hugs to comfort and support you	Social	65341.00	-0.10	0.03	0.00	0.19	0.27	-	-	-
Stigmasterol intake (mg/day)	Nutrients	58295.00	-0.10	0.01	0.00	0.00	0.61	0.06	0.84	74.00
Tiamin intake (mg/day)	Nutrients	58295.00	0.00	0.01	0.73	0.00	0.95	-	-	-
Vitamin E intake (mg/day)	Nutrients	58295.00	0.01	0.01	0.19	0.00	0.48	-	-	-
Trans fat intake (g/day)	Nutrients	58295.00	-0.02	0.01	0.15	0.00	0.60	-	-	-
Sum of phytosterols intake (mg/day)	Nutrients	58295.00	-0.07	0.01	0.00	0.00	0.34	0.06	0.84	99.00
Zinc intake (mg/day)	Nutrients	58295.00	0.05	0.01	0.00	0.17	0.27	0.06	0.84	110.00

Supplementary Table 4. Linear mixed model association results for SBP

Description	Group	N	Effect estimate	S.E.	p-value	I^2	Q p-value	marginal R2	conditional R2	R2 rank
Alcohol intake (g/day)	Alcohol	57971.00	0.04	0.07	0.61	0.00	0.52	-	-	-
Permanent employment	Psychosocial	57020.00	0.09	0.14	0.50	0.39	0.20	-	-	-
Self-employed	Psychosocial	56375.00	-0.61	0.22	0.01	0.00	0.39	-	-	-
Distance to work in kilometers (one way)	Physical activity	56646.00	0.01	0.06	0.81	0.00	0.58	-	-	-
Last time a colleague visited you at home	Psychosocial	62195.00	0.14	0.06	0.02	0.00	0.93	-	-	-
Job demands to work very fast	Psychosocial	65224.00	-0.07	0.06	0.21	0.00	0.88	-	-	-
Frequency of social contacts with colleagues during leisure time	Psychosocial	61357.00	0.22	0.06	0.00	0.00	0.59	-	-	-
High physical demand from job	Physical activity	65372.00	0.18	0.07	0.01	0.00	0.89	-	-	-
Enough time for job assignments	Psychosocial	64934.00	0.15	0.06	0.01	0.00	0.43	-	-	-
Control over planning and execution of the workday	Psychosocial	65234.00	-0.04	0.06	0.45	0.00	0.45	-	-	-
Ingenuity or creativity demand from job	Psychosocial	64930.00	-0.05	0.06	0.45	0.00	0.89	-	-	-
Frequent social contacts with colleagues during work	Psychosocial	62420.00	-0.02	0.06	0.68	0.00	0.62	-	-	-
Contradictory demands in job	Psychosocial	64761.00	-0.18	0.06	0.00	0.00	0.61	-	-	-
Possibility to leave your work for a while to speak with a colleague	Psychosocial	62467.00	-0.07	0.06	0.24	0.00	0.40	-	-	-
Learn new things at job	Psychosocial	65091.00	-0.15	0.06	0.01	0.00	0.90	-	-	-
High mental demand from job	Psychosocial	64759.00	0.19	0.06	0.00	0.00	0.79	-	-	-
Repetitive job	Psychosocial	65109.00	0.29	0.06	0.00	0.70	0.07	-	-	-
Skill demand from job	Psychosocial	65016.00	-0.07	0.06	0.24	0.00	0.34	-	-	-
Possibility to speak with colleagues during breaks	Psychosocial	62864.00	-0.01	0.06	0.87	0.15	0.28	-	-	-
Control over own work assignment	Psychosocial	65195.00	0.04	0.06	0.48	0.00	0.97	-	-	-
Vitamin C intake (mg/day)	Nutrients	57971.00	0.13	0.06	0.04	0.84	0.01	-	-	-
Vitamin B12 intake (ug/day)	Nutrients	57971.00	-0.04	0.06	0.54	0.00	0.67	-	-	-
Vitamin B2 intake (ug/day)	Nutrients	57971.00	-0.12	0.06	0.06	0.00	0.41	-	-	-
Vitamin B6 intake (mg/day)	Nutrients	57971.00	0.29	0.06	0.00	0.62	0.10	-	-	-
Informed of having high blood pressure	General health	65839.00	16.07	0.14	0.00	0.00	0.83	0.36	0.59	1.00
Beta-sitostanol intake (mg/day)	Nutrients	57971.00	0.27	0.06	0.00	0.00	0.81	0.21	0.62	17.00
Beta-sitosterol intake (mg/day)	Nutrients	57971.00	0.18	0.06	0.01	0.00	0.70	-	-	-
Marital status: Single vs Married/partner	Social	60722.00	-1.87	0.22	0.00	0.00	0.35	0.21	0.61	10.00
Marital status: Single vs Divorced/separated	Social	10949.00	-2.17	0.33	0.00	0.00	0.97	0.23	0.59	3.00
Marital status: Single vs Widow/widower	Social	7005.00	0.22	0.70	0.76	0.00	0.87	-	-	-
Campestanol intake (mg/day)	Nutrients	57971.00	0.21	0.06	0.00	0.00	0.91	-	-	-
Campesterol intake (mg/day)	Nutrients	57971.00	0.12	0.06	0.04	0.44	0.18	-	-	-
Parents or siblings have diabetes	General health	64464.00	0.59	0.16	0.00	0.33	0.22	-	-	-
Disaccharides intake (g/day)	Nutrients	57971.00	0.07	0.07	0.28	0.00	0.84	-	-	-
Vitamin D intake (ug/day)	Nutrients	57971.00	-0.05	0.06	0.43	0.17	0.27	-	-	-
Total energy intake (kcal/day)	Nutrients	57971.00	-0.24	0.07	0.00	0.00	0.75	-	-	-

Formic acid intaje (g/day)	Nutrients	57971.00	-0.22	0.06	0.00	0.00	0.57	-	-	-
Pentadecanoic acid intake (g/day)	Nutrients	57971.00	-0.23	0.06	0.00	0.00	0.91	-	-	-
Palmitic acid intake (g/day)	Nutrients	57971.00	-0.24	0.06	0.00	0.00	0.56	-	-	-
Heptadecanoic acid intake (g/day)	Nutrients	57971.00	-0.23	0.06	0.00	0.00	0.91	-	-	-
Linoleic acid intake (g/day)	Nutrients	57971.00	-0.07	0.06	0.23	0.18	0.27	-	-	-
Linolenic acid intake (g/day)	Nutrients	57971.00	-0.03	0.06	0.60	0.51	0.15	-	-	-
Arachidonic acid (ARA) intake (g/day)	Nutrients	57969.00	-0.04	0.06	0.55	0.00	0.61	-	-	-
Eicosapentaenoic acid (EPA) intake (g/day)	Nutrients	57970.00	-0.07	0.06	0.27	0.00	0.56	-	-	-
Docosahexaenoic acid (DHA) intake (g/day)	Nutrients	57971.00	-0.09	0.06	0.13	0.00	0.74	-	-	-
Fat intake (g/day)	Nutrients	57971.00	-0.28	0.06	0.00	0.68	0.08	-	-	-
Fibre intake (g/day)	Nutrients	57971.00	0.28	0.07	0.00	0.53	0.14	-	-	-
Folic acid intake (ug/day)	Nutrients	57971.00	0.14	0.07	0.04	0.73	0.05	-	-	-
Phosphate intake (mg/day)	Nutrients	57971.00	-0.07	0.06	0.23	0.49	0.16	-	-	-
Whole grain intake (g/day)	Food	57971.00	0.37	0.06	0.00	0.00	0.98	0.21	0.62	14.00
Travel to work: Walk to work vs passive travel to work	Physical activity	42220.00	0.41	0.22	0.06	0.00	0.61	-	-	-
Travel to work: Cycle to work vs passive travel to work	Physical activity	49399.00	-0.64	0.17	0.00	0.00	0.45	-	-	-
Travel to work: Irregular travel mode to work vs passive travel to work	Physical activity	42344.00	-0.03	0.21	0.91	0.00	0.35	-	-	-
Time spent in a week in moderately steneous activities	Physical activity	21836.00	-0.20	0.11		0.00	0.96	-	-	-
Sedentary or standing work	Physical activity	63460.00	-0.21	0.14	0.13	0.08	0.30	-	-	-
Light but partly physically active work	Physical activity	63460.00	-0.22	0.14	0.13	0.70	0.07	-	-	-
Light and physically active work	Physical activity	63460.00	0.23	0.13	0.09	0.00	0.68	-	-	-
Sometimes physically straining work	Physical activity	63460.00	0.08	0.13	0.55	0.31	0.23	-	-	-
Frequency of walking during leisure time	Physical activity	63031.00	0.20	0.06	0.00	0.00	0.47	-	-	-
Frequency of cycling during leisure time	Physical activity	53447.00	-0.10	0.07	0.15	0.00	0.62	-	-	-
Frequency of dancing during leisure time	Physical activity	37681.00	-0.27	0.08	0.00	0.42	0.19	-	-	-
Frequency of shoveling snow during leisure time	Physical activity	40776.00	-0.06	0.08	0.42	0.00	0.68	-	-	-
Frequency of gardening during leisure time	Physical activity	40535.00	0.09	0.07	0.25	0.00	0.32	-	-	-
Frequency of hunting or fishing during leisure time	Physical activity	39487.00	0.41	0.08	0.00	0.00	0.89	0.20	0.60	25.00
Frequency of picking berries or mushrooms during leisure time	Physical activity	40345.00	0.18	0.08	0.02	0.39	0.20	-	-	-
Changed everyday exercise during the last year	Physical activity	42948.00	0.26	0.07	0.00	0.36	0.21	-	-	-
Everyday exercise satisfaction	Physical activity	42823.00	0.14	0.07	0.05	0.00	0.45	-	-	-
Exercise during the last three months	Physical activity	64709.00	-0.12	0.06	0.04	0.00	0.52	-	-	-
If you exercise, change in exercise habits during the last year	Physical activity	36924.00	0.18	0.07	0.01	0.00	0.33	-	-	-
Amount of exercise during the last 12 months	Physical activity	22532.00	-0.27	0.11		0.00	0.65	-	-	-
Bregott on bread	Food	57971.00	-0.09	0.06	0.14	0.00	0.39	-	-	-

Whole grain crisp bread	Food	57971.00	0.37	0.06	0.00	0.00	0.60	0.21	0.62	13.00
Whole grain soft bread	Food	57971.00	0.07	0.06	0.26	0.00	0.80	-	-	-
White (soft) bread, thin crisp bread	Food	57971.00	0.05	0.06	0.43	0.00	0.47	-	-	-
Coffee rolls/buns, rusk	Food	57971.00	0.17	0.06	0.01	0.00	0.49	-	-	-
Cheese 28%	Food	57971.00	-0.14	0.06	0.02	0.00	0.83	-	-	-
Cheese 10-17%	Food	57971.00	0.09	0.06	0.14	0.55	0.13	-	-	-
Soft cheese	Food	13649.00	0.04	0.12		0.27	0.24	-	-	-
Soft whey cheese	Food	13649.00	-0.01	0.12		0.48	0.16	-	-	-
Sausage, liver pate on bread	Food	57971.00	0.17	0.06	0.01	0.00	0.38	-	-	-
Meat on bread	Food	57971.00	0.11	0.06	0.07	0.00	0.77	-	-	-
Butter on bread	Food	57971.00	-0.02	0.06	0.70	0.00	0.90	-	-	-
Oatflake, whole wheat, rye or barley porridge	Food	57971.00	-0.05	0.06	0.40	0.28	0.24	-	-	-
Rosehip, sweet syrup soup	Food	57971.00	0.08	0.06	0.15	0.40	0.20	-	-	-
Sour milk, yoghurt (3% fat)	Food	57971.00	-0.17	0.06	0.00	0.00	0.70	-	-	-
Sour milk, yoghurt (low fat)	Food	57971.00	0.05	0.06	0.37	0.00	0.85	-	-	-
Fiber cereals	Food	57971.00	-0.15	0.06	0.02	0.00	0.70	-	-	-
Corn flakes	Food	57971.00	-0.14	0.06	0.02	0.00	0.43	-	-	-
Berries (fresh or frozen)	Food	57971.00	-0.12	0.06	0.05	0.00	0.49	-	-	-
Apple, pear, peach, orange, mandarin and grapefruit	Food	57971.00	0.15	0.07	0.03	0.81	0.02	-	-	-
Ba	Food	57971.00	0.19	0.06	0.00	0.00	0.60	-	-	-
Root vegetables and carrot	Food	57971.00	0.00	0.07	0.97	0.00	0.91	-	-	-
Low fat margarine on bread	Food	57971.00	0.23	0.06	0.00	0.00	0.98	0.21	0.62	20.00
Tomato and cucumber	Food	57971.00	0.05	0.07	0.43	0.00	0.35	-	-	-
White cabbage, lettuce, lettuce cabbage, spich, borecole and broccoli	Food	57971.00	-0.02	0.07	0.76	0.26	0.25	-	-	-
Mixed frozen vegetables	Food	13649.00	0.18	0.12		0.00	0.37	-	-	-
Boiled or baked potato	Food	57971.00	0.25	0.06	0.00	0.00	0.37	-	-	-
Fried potatoes and pommes frites	Food	57971.00	-0.03	0.06	0.66	0.00	0.80	-	-	-
Mashed potato	Food	13649.00	-0.04	0.12		0.00	0.97	-	-	-
Potato salad	Food	10949.00	0.16	0.13		0.00	0.77	-	-	-
Rice	Food	57971.00	-0.13	0.06	0.03	0.00	0.71	-	-	-
Pasta	Food	57971.00	-0.17	0.06	0.01	0.00	0.82	-	-	-
Brown beans and pea soup	Food	57971.00	-0.01	0.06	0.85	0.00	0.56	-	-	-
Margarine on bread	Food	57971.00	-0.05	0.06	0.42	0.00	0.77	-	-	-
Blota (broth + bread)	Food	13649.00	0.26	0.12		0.83	0.01	-	-	-
Pancake, waffle and Swedish dumpling	Food	57971.00	-0.01	0.06	0.88	0.00	0.93	-	-	-
Pizza	Food	57971.00	-0.03	0.06	0.63	0.00	0.89	-	-	-
Minced meat dishes	Food	57971.00	-0.08	0.06	0.18	0.43	0.19	-	-	-
Meat stew	Food	57971.00	0.00	0.06	0.99	0.05	0.31	-	-	-
Steak, chop, etc.	Food	57971.00	-0.02	0.06	0.78	0.00	0.97	-	-	-
Bacon	Food	57971.00	-0.13	0.06	0.04	0.00	0.88	-	-	-
Sausage as main dish	Food	57971.00	-0.04	0.06	0.53	0.00	0.50	-	-	-
Hamburger	Food	57971.00	-0.09	0.06	0.18	0.00	0.77	-	-	-
White meat (poultry)	Food	57971.00	-0.24	0.06	0.00	0.00	0.96	0.21	0.62	22.00
Butter for cooking	Food	57971.00	-0.06	0.06	0.33	0.39	0.20	-	-	-
Blood based food	Food	13649.00	0.10	0.12		0.00	0.33	-	-	-
Liver and kidney	Food	13649.00	0.08	0.12		0.00	0.60	-	-	-
Lean fish (e.g. perch, bass, cod)	Food	57971.00	-0.03	0.06	0.60	0.80	0.02	-	-	-
Fatty fish (e.g. herring, whitefish, salmon)	Food	57971.00	-0.11	0.06	0.08	0.00	0.77	-	-	-
Shellfish (e.g. shrimps, scallops)	Food	13649.00	-0.14	0.12		0.00	0.64	-	-	-
Salty fish	Food	57971.00	0.02	0.06	0.70	0.07	0.30	-	-	-
Smoked fish/meat	Food	57971.00	0.02	0.06	0.75	0.00	0.57	-	-	-
Ice cream	Food	57971.00	-0.19	0.06	0.00	0.00	0.49	-	-	-
Sweets	Food	57971.00	-0.08	0.06	0.23	0.00	0.84	-	-	-
Sugar, honey, marmelade and jam	Food	57971.00	-0.12	0.06	0.06	0.00	0.94	-	-	-
Margarine for cooking	Food	57971.00	0.06	0.06	0.29	0.25	0.25	-	-	-
Cookies and pastry	Food	57971.00	-0.03	0.06	0.68	0.00	0.58	-	-	-

Chips, popcorn and salted nuts	Food	57971.00	-0.02	0.06	0.72	0.00	0.91	-	-	-
Low fat milk (0.5%)	Beverage	57971.00	0.06	0.06	0.30	0.00	0.56	-	-	-
Milk, sour milk (1.5%)	Beverage	57971.00	-0.13	0.06	0.03	0.00	0.91	-	-	-
Milk, sour milk (3%)	Beverage	57971.00	-0.03	0.06	0.65	0.63	0.10	-	-	-
Sodas, soft drinks and juice	Beverage	57971.00	0.20	0.06	0.00	0.00	0.54	-	-	-
Brewed (filtered) coffee	Beverage	57971.00	-0.40	0.07	0.00	0.00	0.52	0.21	0.62	15.00
Boiled coffee	Beverage	57971.00	0.16	0.07	0.02	0.00	0.46	-	-	-
Tea	Beverage	57971.00	-0.10	0.07	0.16	0.00	0.96	-	-	-
Light beer	Alcohol	57971.00	-0.13	0.06	0.05	0.00	0.40	-	-	-
Oil for cooking	Food	57971.00	-0.07	0.06	0.29	0.00	0.65	-	-	-
Medium beer	Alcohol	57971.00	-0.02	0.06	0.74	0.00	0.57	-	-	-
Strong beer	Alcohol	57971.00	0.22	0.07	0.00	0.00	0.60	-	-	-
Wine	Alcohol	57971.00	-0.03	0.07	0.69	0.00	0.36	-	-	-
Liquor and spirits	Alcohol	57971.00	0.03	0.07	0.68	0.00	0.39	-	-	-
Salad dressing with oil	Food	57971.00	-0.21	0.06	0.00	0.72	0.06	-	-	-
Cream, creme fraiche, sour cream	Food	57971.00	-0.15	0.06	0.01	0.00	0.42	-	-	-
Average portion size of vegetables based on photographic illustration of four sizes (smallest to largest)	Food	57971.00	-0.17	0.07	0.01	0.00	0.69	-	-	-
Overall state of health during the last year	General health	65496.00	-0.61	0.06	0.00	0.08	0.30	0.21	0.61	9.00
Overall state of health compared to others your age	General health	32593.00	-0.64	0.08	0.00	0.74	0.05	0.20	0.55	32.00
Parents or siblings had a cerebral hemorrhage/thrombosis or cardiac infarction before the age of 60	General health	64414.00	1.75	0.16	0.00	0.51	0.15	0.21	0.61	5.00
Teetotaler	Alcohol	42228.00	0.81	0.26	0.00	0.81	0.02	-	-	-
Feel the need to reduce alcohol consumption	Alcohol	59477.00	0.36	0.19	0.06	0.00	0.90	-	-	-
Feel uneasy or guilty because of your way of drinking	Alcohol	38073.00	-0.31	0.24	0.19	0.00	0.75	-	-	-
Frequency of alcohol consumption	Alcohol	22647.00	0.03	0.11		0.46	0.17	-	-	-
Amount of alcohol drunk in a day	Alcohol	21576.00	0.62	0.12		0.50	0.16	-	-	-
Frequency of drinking six or more glasses at the same occasion	Alcohol	21995.00	0.71	0.12		0.54	0.14	-	-	-
Times during last year that you felt guilty because of your drinking	Alcohol	21959.00	0.20	0.11		0.00	0.57	-	-	-
Iron intake (mg/day)	Nutrients	57971.00	0.13	0.06	0.05	0.00	0.42	-	-	-
Iodine intake (ug/day)	Nutrients	57971.00	-0.23	0.06	0.00	0.52	0.15	-	-	-
Calcium intake (mg/day)	Nutrients	57971.00	-0.16	0.06	0.01	0.00	0.70	-	-	-
Potassium intake (mg/day)	Nutrients	57971.00	0.16	0.07	0.02	0.77	0.04	-	-	-
Beta-carotene intake (mg/day)	Nutrients	57971.00	0.00	0.07	0.99	0.00	0.96	-	-	-
Cholesterol intake (g/day)	Nutrients	57971.00	-0.29	0.06	0.00	0.00	0.93	0.21	0.62	18.00
Carbohydrates intake (g/day)	Nutrients	57971.00	0.27	0.06	0.00	0.00	0.33	-	-	-
Average portion size of meat/fish based on photographic illustration of four sizes (smallest to largest)	Food	57971.00	-0.31	0.07	0.00	0.00	0.72	0.20	0.62	24.00
Breakfast habits: Only coffee/tea for breakfast vs not breakfast at all	Food	3880.00	0.23	0.59	0.70	0.00	0.35	-	-	-
Breakfast habits: Coffee/tea and wheat buns or rusk for breakfast vs not breakfast at all	Food	3168.00	-0.50	0.58	0.39	0.00	0.75	-	-	-

Breakfast habits: Porridge w/o sandwich for breakfast vs not breakfast at all	Food	8474.00	0.11	0.46	0.82	0.00	0.78	-	-	-
Breakfast habits: Gruel w/o sandwich for breakfast vs not breakfast at all	Food	2941.00	1.31	0.71	0.06	0.00	0.80	-	-	-
Eat breakfast from 2000	Food	33604.00	-0.98	0.32	0.00	0.00	0.56	-	-	-
Enterodiol intake (ug/day)	Nutrients	57971.00	0.04	0.06	0.56	0.00	0.61	-	-	-
Enterolactone intake (ug/day)	Nutrients	57971.00	-0.07	0.06	0.24	0.00	0.84	-	-	-
Equol intake (ug/day)	Nutrients	57971.00	0.08	0.06	0.19	0.00	0.72	-	-	-
Lariciresinol intake (ug/day)	Nutrients	57971.00	0.18	0.07	0.01	0.57	0.13	-	-	-
Matairesinol intake (ug/day)	Nutrients	57971.00	0.25	0.06	0.00	0.00	0.48	-	-	-
Medioresinol intake (ug/day)	Nutrients	57971.00	0.31	0.06	0.00	0.00	0.82	0.21	0.62	16.00
Pinoresinol intake (ug/day)	Nutrients	57971.00	0.16	0.07	0.02	0.00	0.35	-	-	-
Secoisolariciresinol intake (ug/day)	Nutrients	57971.00	-0.12	0.07	0.06	0.29	0.23	-	-	-
Sum of all ligns intake (ug/day)	Nutrients	57971.00	0.26	0.06	0.00	0.00	0.54	-	-	-
Syringaresinol intake (ug/day)	Nutrients	57971.00	0.27	0.06	0.00	0.00	0.79	0.21	0.62	19.00
Sum of Lariciresinol, Matairesinol, Pinoresinol, Secoisolariciresinol intake (ug/day)	Nutrients	57971.00	0.15	0.07	0.02	0.40	0.20	-	-	-
Satisfaction with home and family situation	Psychosocial	48413.00	0.26	0.07	0.00	0.00	0.97	0.20	0.64	31.00
Appetite status	Psychosocial	48403.00	-0.06	0.07	0.43	0.25	0.25	-	-	-
Mood status	Psychosocial	48368.00	0.22	0.07	0.00	0.00	0.78	-	-	-
Energy status	Psychosocial	48337.00	0.06	0.07	0.44	0.63	0.10	-	-	-
Patience status	Psychosocial	48376.00	0.07	0.07	0.37	0.00	0.51	-	-	-
Confidence status	Psychosocial	48350.00	-0.02	0.08	0.81	0.00	0.79	-	-	-
Sleep status	Sleep	48446.00	-0.05	0.07	0.46	0.62	0.10	-	-	-
Do you feel important and appreciated outside your home?	Psychosocial	48439.00	0.12	0.07	0.11	0.52	0.15	-	-	-
Do you feel important and appreciated in your home?	Psychosocial	48080.00	0.28	0.07	0.00	0.00	0.52	-	-	-
Satisfaction with accomodation	Psychosocial	48455.00	0.25	0.07	0.00	0.00	0.98	-	-	-
Satisfaction with work situation	Psychosocial	47813.00	-0.05	0.07	0.52	0.00	0.89	-	-	-
Satisfaction with economy	Psychosocial	48403.00	0.36	0.08	0.00	0.00	0.98	0.20	0.64	26.00
Satisfaction with leisure time	Psychosocial	48341.00	0.30	0.07	0.00	0.00	0.56	0.20	0.64	28.00
Hearing status	General health	48438.00	0.33	0.08	0.00	0.79	0.03	-	-	-
Vision status	General health	48376.00	0.06	0.07	0.39	0.00	0.84	-	-	-
Memory status	Psychosocial	48305.00	0.45	0.07	0.00	0.00	0.88	0.20	0.64	27.00
Fitness status	Physical activity	48372.00	-0.31	0.08	0.00	0.00	1.00	0.20	0.64	29.00
Magnesium intake (mg/day)	Nutrients	57971.00	0.14	0.07	0.03	0.75	0.04	-	-	-
Saturated fat intake (g/day)	Nutrients	57971.00	-0.28	0.06	0.00	0.14	0.28	-	-	-
Monounsaturated fat intake (g/day)	Nutrients	57971.00	-0.17	0.07	0.01	0.00	0.44	-	-	-
Monosaccharides intake (g/day)	Nutrients	57971.00	0.22	0.07	0.00	0.57	0.13	-	-	-
Sodium intake (mg/day)	Nutrients	57969.00	-0.07	0.06	0.24	0.00	0.98	-	-	-
Vitamin B3 intake (mg/day)	Nutrients	57971.00	-0.11	0.06	0.08	0.27	0.24	-	-	-
Cambridge physical activity index	Physical activity	62661.00	-0.02	0.06	0.75	0.00	0.60	-	-	-
Polyunsaturated fat intake (g/day)	Nutrients	57971.00	-0.07	0.06	0.28	0.19	0.27	-	-	-

Average portion size of potatoes/rice/pasta based on photographic illustration of four sizes (smallest to largest)	Food	57971.00	-0.14	0.07	0.04	0.00	0.54	-	-	-
Total protein intake (g/day)	Nutrients	57971.00	-0.17	0.06	0.01	0.00	0.34	-	-	-
Animal based protein intake (g/day)	Nutrients	57971.00	-0.23	0.06	0.00	0.00	0.44	-	-	-
Plant based protein intake (g/day)	Nutrients	57971.00	0.20	0.06	0.00	0.00	0.78	-	-	-
Vitamin A intake (mg/day)	Nutrients	57971.00	-0.02	0.07	0.72	0.00	0.74	-	-	-
Sucrose intake (g/day)	Nutrients	57971.00	0.07	0.06	0.30	0.00	0.77	-	-	-
Cohabitation: Live alone vs Only one adult (spouse, partner)	Social	30073.00	-0.28	0.25	0.26	0.00	0.34	-	-	-
Cohabitation: Live alone vs Only children	Social	9774.00	-1.67	0.37	0.00	0.00	0.96	0.23	0.59	2.00
Cohabitation: Live alone vs Adult and children	Social	38148.00	-1.57	0.22	0.00	0.00	0.74	0.20	0.59	30.00
Cohabitation: Live alone vs Other/others	Social	7568.00	-0.35	0.61	0.56	0.00	0.47	-	-	-
Selenium intake (ug/day)	Nutrients	57971.00	-0.20	0.06	0.00	0.16	0.28	-	-	-
Self rate of overall health	General health	28583.00	-1.06	0.10	0.00	0.00	0.86	0.20	0.66	33.00
For how much of the time during the last four weeks has your physical health or your emotiol problems limited your ability to interact with others?	General health	28345.00	-0.21	0.10	0.04	0.00	0.99	-	-	-
Get sick more often than other people	General health	28469.00	0.29	0.10	0.00	0.28	0.24	-	-	-
As healthy as anyone	General health	28384.00	-0.61	0.10	0.00	0.36	0.21	0.19	0.66	35.00
Worsen in health in the future	General health	28376.00	0.38	0.10	0.00	0.00	0.70	-	-	-
Excellent health	General health	28471.00	-1.06	0.10	0.00	0.00	0.88	0.20	0.66	34.00
Self rate of overall health compared to a year ago	General health	28343.00	-0.25	0.10	0.01	0.00	0.44	-	-	-
Physical limitation to participate in steneous activities: running, lifting heavy objects, taking part in physically demanding sports	General health	28530.00	0.28	0.10	0.01	0.00	0.85	-	-	-
Physical limitation to participate in moderately demanding activities: moving a table, vacuuming, walking in the forest or gardening	General health	28520.00	0.06	0.10	0.58	0.00	0.37	-	-	-
Physical limitation to participate in moderately demanding activities: lifting or carrying grocery bags	General health	28499.00	0.01	0.10	0.95	0.00	0.98	-	-	-
Physical limitation to participate in moderately demanding activities: walking up several stairs	General health	28510.00	0.38	0.10	0.00	0.56	0.13	-	-	-
Physical limitation to participate in moderately demanding activities: bending down or kneeling	General health	28478.00	0.15	0.10	0.15	0.83	0.02	-	-	-
Physical limitation to participate in moderately demanding activities: walking more than two km	General health	28521.00	0.13	0.10	0.18	0.00	0.36	-	-	-

Physical limitation that reduced the normal time spent at work or in other activities during the last four weeks	General health	28546.00	0.35	0.31	0.27	0.00	0.59	-	-	-
Physical limitation that made you do less than you wanted during the last four weeks	General health	28530.00	-0.05	0.25	0.84	0.00	0.36	-	-	-
Physical limitation that made you not being able to perform certain work tasks or other activities during the last four weeks	General health	28419.00	-0.11	0.28	0.69	0.00	0.60	-	-	-
Physical limitation that limited your ability to perform certain work tasks or other activities during the last four weeks	General health	28452.00	-0.14	0.28	0.63	0.00	0.92	-	-	-
Emotiol problems that made you do less than you wanted during the last four weeks	General health	28546.00	-1.31	0.29	0.00	0.00	0.95	0.19	0.66	36.00
Extent to what your physical and emotiol health disrupted your usual social life during the last four weeks	General health	28527.00	-0.26	0.10	0.01	0.00	0.68	-	-	-
Pain during the last four weeks	General health	28489.00	0.19	0.10	0.06	0.00	0.54	-	-	-
How much has the pain during the last four weeks disturbed your normal work?	General health	28427.00	0.15	0.10	0.14	0.00	0.95	-	-	-
For how much of the time during the last four weeks have you felt really alert and strong?	General health	28439.00	-0.09	0.10	0.36	0.00	0.45	-	-	-
For how much of the time during the last four weeks have you felt very nervous?	General health	28486.00	0.14	0.10	0.14	0.43	0.18	-	-	-
For how much of the time during the last four weeks have you felt so depressed that nothing could cheer you up?	General health	28516.00	-0.22	0.10	0.02	0.00	0.46	-	-	-
For how much of the time during the last four weeks have you felt calm and serene?	General health	28492.00	0.01	0.10	0.90	0.00	0.95	-	-	-
For how much of the time during the last four weeks have you felt full of energy?	General health	28456.00	0.01	0.10	0.88	0.00	0.51	-	-	-
For how much of the time during the last four weeks have you felt gloomy and sad?	General health	28464.00	-0.40	0.10	0.00	0.00	0.99	0.19	0.66	37.00
For how much of the time during the last four weeks have you felt worn out?	General health	28421.00	0.02	0.10	0.82	0.00	0.36	-	-	-
For how much of the time during the last four weeks have you felt happy?	General health	28483.00	0.15	0.10	0.13	0.47	0.17	-	-	-
For how much of the time during the last four weeks have you felt tired?	General health	28509.00	-0.17	0.10	0.10	0.00	0.41	-	-	-
Long-term sickness	General health	61554.00	-0.23	0.16	0.16	0.43	0.19	-	-	-
Work shifts/weekends	Psychosocial	63185.00	-0.14	0.14	0.32	0.80	0.02	-	-	-
Risk of sleeping while sitting and reading	Sleep	22130.00	-0.49	0.11		0.00	0.63	-	-	-

Risk of sleeping while watching TV	Sleep	22367.00	0.08	0.11		0.00	0.92	-	-	-
Risk of sleeping while sitting ictive in a public place	Sleep	22220.00	-0.56	0.11		0.00	0.39	-	-	-
Risk of sleeping as a passenger in a car for one hour without break	Sleep	22136.00	-0.78	0.11		0.55	0.14	-	-	-
Risk of sleeping while lying down resting in the afternoon	Sleep	22288.00	-0.37	0.11		0.62	0.10	-	-	-
Risk of sleeping while sitting still after having lunch	Sleep	22302.00	-0.43	0.11		0.00	0.37	-	-	-
Snore during sleep	Sleep	20880.00	0.60	0.12		0.00	0.92	-	-	-
Breath-holds during sleep	Sleep	15608.00	0.34	0.14		0.09	0.29	-	-	-
Number of cigarretes smoked per day (in groups)	Tobacco use	53212.00	-0.55	0.08	0.00	0.00	0.54	0.21	0.61	23.00
Years smoking	Tobacco use	57554.00	-0.86	0.08	0.00	0.00	0.39	0.21	0.61	8.00
Grams of tobacco smoked per week	Tobacco use	41546.00	-0.07	0.07	0.37	0.00	0.84	-	-	-
Number of cigarretes smoked per day	Tobacco use	42572.00	-0.35	0.08	0.00	0.00	0.94	0.21	0.61	7.00
Number of cigars smoked per day	Tobacco use	41510.00	-0.09	0.07	0.22	0.74	0.05	-	-	-
Smoking status: Smokers vs non-smokers	Tobacco use	42247.00	-2.75	0.21	0.00	0.00	0.96	0.21	0.61	21.00
Smoking status: Former smokers vs non-smokers	Tobacco use	46901.00	-1.42	0.19	0.00	0.19	0.27	0.21	0.62	4.00
Smoking status: Former occasiol smokers vs non-smokers	Tobacco use	39116.00	-1.44	0.22	0.00	0.00	0.53	0.21	0.62	11.00
Number of snuff boxes per week	Tobacco use	63715.00	-0.38	0.07	0.00	0.00	0.59	0.21	0.61	6.00
Snuff status: Snuff users vs non-snuff users	Tobacco use	56252.00	-1.27	0.20	0.00	0.00	0.80	0.21	0.61	12.00
Snuff status: Former snuff users vs non-snuff users	Tobacco use	52627.00	0.01	0.23	0.98	0.17	0.27	-	-	-
Years using snuff	Tobacco use	61980.00	-0.18	0.08	0.02	0.00	0.81	-	-	-
Participation in associations or voluntary organisations	Social	65130.00	-0.51	0.12	0.00	0.41	0.19	-	-	-
Participation in sports or physical exercise associations	Social	20984.00	-0.65	0.23	0.00	0.00	0.88	-	-	-
Participation in study circles	Social	20984.00	0.33	0.31	0.29	0.14	0.28	-	-	-
Participation in other association	Social	20984.00	0.22	0.22	0.33	0.00	0.59	-	-	-
People to ask for help apart from the ones at home	Social	65884.00	0.19	0.19	0.32	0.00	0.82	-	-	-
Number of friends that can come to your home at any time and feel at home	Social	65259.00	0.05	0.06	0.46	0.00	0.51	-	-	-
Number of social contacts with the same interests as you	Social	65969.00	-0.03	0.06	0.66	0.00	0.91	-	-	-
Would you say that the number of people that you meet in your everyday life is enough or would you like to meet more or fewer people?	Social	66079.00	-0.20	0.06	0.00	0.00	0.92	-	-	-
Close relationship with anyone	Social	65397.00	0.04	0.06	0.44	0.00	0.39	-	-	-
Frequency of engaging in clubs, associations or study circles	Social	41712.00	-0.22	0.07	0.00	0.00	0.83	-	-	-

Number of social interactions during a normal week	Social	65464.00	0.16	0.06	0.01	0.00	0.37	-	-	-
Support from others	Social	66129.00	0.07	0.06	0.21	0.00	0.67	-	-	-
Number of people with whom you can speak openly	Social	66043.00	0.12	0.06	0.04	0.00	0.77	-	-	-
Receive hugs to comfort and support you	Social	64980.00	-0.20	0.16	0.22	0.18	0.27	-	-	-
Stigmasterol intake (mg/day)	Nutrients	57971.00	0.18	0.07	0.01	0.00	0.71	-	-	-
Tiamin intake (mg/day)	Nutrients	57971.00	0.06	0.06	0.36	0.43	0.19	-	-	-
Vitamin E intake (mg/day)	Nutrients	57971.00	0.01	0.06	0.93	0.00	0.64	-	-	-
Trans fat intake (g/day)	Nutrients	57971.00	0.03	0.09	0.72	0.00	0.69	-	-	-
Sum of phytosterols intake (mg/day)	Nutrients	57971.00	0.18	0.06	0.00	0.00	0.93	-	-	-
Zinc intake (mg/day)	Nutrients	57971.00	-0.09	0.06	0.18	0.63	0.10	-	-	-

Supplementary Table 5. Linear mixed model association results for DBP

Description	Group	N	Effect estimate	S.E.	p-value	I^2	Q p-value	marginal R2	conditional R2	R2 rank
Alcohol intake (g/day)	Alcohol	57944.00	0.12	0.05	0.01	0.43	0.18	-	-	-
Permanent employment	Psychosocial	56996.00	0.15	0.09	0.10	0.61	0.11	-	-	-
Self-employed	Psychosocial	56351.00	-0.26	0.15	0.08	0.19	0.27	-	-	-
Distance to work in kilometers (one way)	Physical activity	56621.00	0.02	0.04	0.68	0.00	0.82	-	-	-
Last time a colleague visited you at home	Psychosocial	62165.00	0.11	0.04	0.01	0.00	0.42	-	-	-
Job demands to work very fast	Psychosocial	65195.00	-0.04	0.04	0.32	0.00	0.82	-	-	-
Frequency of social contacts with colleagues during leisure time	Psychosocial	61328.00	0.18	0.04	0.00	0.00	0.58	0.19	0.57	11.00
High physical demand from job	Physical activity	65343.00	-0.01	0.04	0.89	0.00	0.87	-	-	-
Enough time for job assignments	Psychosocial	64905.00	0.13	0.04	0.00	0.24	0.25	-	-	-
Control over planning and execution of the workday	Psychosocial	65205.00	-0.03	0.04	0.47	0.00	0.46	-	-	-
Ingenuity or creativity demand from job	Psychosocial	64901.00	-0.06	0.04	0.15	0.00	0.76	-	-	-
Frequent social contacts with colleagues during work	Psychosocial	62391.00	0.06	0.04	0.12	0.00	0.58	-	-	-
Contradictory demands in job	Psychosocial	64732.00	-0.10	0.04	0.01	0.36	0.21	-	-	-
Possibility to leave your work for a while to speak with a colleague	Psychosocial	62437.00	-0.01	0.04	0.86	0.55	0.14	-	-	-
Learn new things at job	Psychosocial	65062.00	-0.07	0.04	0.10	0.54	0.14	-	-	-
High mental demand from job	Psychosocial	64730.00	0.14	0.04	0.00	0.00	0.76	-	-	-
Repetitive job	Psychosocial	65080.00	0.16	0.04	0.00	0.75	0.04	-	-	-
Skill demand from job	Psychosocial	64987.00	-0.07	0.04	0.08	0.20	0.26	-	-	-
Possibility to speak with colleagues during breaks	Psychosocial	62835.00	0.01	0.04	0.78	0.00	0.58	-	-	-
Control over own work assignment	Psychosocial	65166.00	-0.03	0.04	0.43	0.00	0.84	-	-	-
Vitamin C intake (mg/day)	Nutrients	57944.00	0.05	0.04	0.21	0.56	0.13	-	-	-
Vitamin B12 intake (ug/day)	Nutrients	57944.00	-0.03	0.04	0.40	0.00	0.87	-	-	-
Vitamin B2 intake (ug/day)	Nutrients	57944.00	-0.13	0.04	0.00	0.39	0.20	-	-	-
Vitamin B6 intake (mg/day)	Nutrients	57944.00	0.07	0.04	0.13	0.00	0.38	-	-	-
Informed of having high blood pressure	General health	65810.00	10.85	0.09	0.00	0.00	0.37	0.35	0.55	1.00
Beta-sitostanol intake (mg/day)	Nutrients	57944.00	0.06	0.04	0.14	0.00	0.42	-	-	-
Beta-sitosterol intake (mg/day)	Nutrients	57944.00	-0.04	0.04	0.32	0.00	0.89	-	-	-
Marital status: Single vs Married/partner	Social	60693.00	-1.15	0.14	0.00	0.72	0.06	0.18	0.57	14.00
Marital status: Single vs Divorced/separated	Social	10945.00	-1.06	0.22	0.00	0.44	0.18	-	-	-
Marital status: Single vs Widow/widower	Social	7002.00	0.03	0.45	0.95	0.15	0.28	-	-	-
Campestanol intake (mg/day)	Nutrients	57944.00	0.03	0.04	0.41	0.00	0.55	-	-	-
Campesterol intake (mg/day)	Nutrients	57944.00	0.02	0.04	0.63	0.00	0.72	-	-	-
Parents or siblings have diabetes	General health	64435.00	0.36	0.11	0.00	0.00	0.44	-	-	-
Disaccharides intake (g/day)	Nutrients	57944.00	-0.07	0.04	0.08	0.56	0.13	-	-	-
Vitamin D intake (ug/day)	Nutrients	57944.00	-0.04	0.04	0.27	0.00	0.78	-	-	-
Total energy intake (kcal/day)	Nutrients	57944.00	-0.23	0.05	0.00	0.00	0.57	0.19	0.59	8.00

Formic acid intaje (g/day)	Nutrients	57944.00	-0.08	0.04	0.06	0.00	0.65	-	-	-
Pentadecanoic acid intake (g/day)	Nutrients	57944.00	-0.12	0.04	0.01	0.00	0.56	-	-	-
Palmitic acid intake (g/day)	Nutrients	57944.00	-0.11	0.04	0.01	0.00	0.70	-	-	-
Heptadecanoic acid intake (g/day)	Nutrients	57944.00	-0.12	0.04	0.01	0.00	0.56	-	-	-
Linoleic acid intake (g/day)	Nutrients	57944.00	-0.08	0.04	0.04	0.00	0.94	-	-	-
Linolenic acid intake (g/day)	Nutrients	57944.00	-0.05	0.04	0.24	0.00	0.98	-	-	-
Arachidonic acid (ARA) intake (g/day)	Nutrients	57942.00	-0.05	0.04	0.21	0.00	0.32	-	-	-
Eicosapentaenoic acid (EPA) intake (g/day)	Nutrients	57943.00	-0.06	0.04	0.15	0.00	0.55	-	-	-
Docosahexaenoic acid (DHA) intake (g/day)	Nutrients	57944.00	-0.07	0.04	0.08	0.00	0.50	-	-	-
Fat intake (g/day)	Nutrients	57944.00	-0.08	0.04	0.07	0.00	0.64	-	-	-
Fibre intake (g/day)	Nutrients	57944.00	0.04	0.05	0.44	0.00	0.68	-	-	-
Folic acid intake (ug/day)	Nutrients	57944.00	-0.10	0.05	0.04	0.00	0.91	-	-	-
Phosphate intake (mg/day)	Nutrients	57944.00	-0.17	0.04	0.00	0.00	0.56	-	-	-
Whole grain intake (g/day)	Food	57944.00	0.09	0.04	0.02	0.00	0.35	-	-	-
Travel to work: Walk to work vs passive travel to work	Physical activity	42198.00	0.23	0.15	0.11	0.25	0.25	-	-	-
Travel to work: Cycle to work vs passive travel to work	Physical activity	49375.00	-0.30	0.11	0.01	0.00	0.55	-	-	-
Travel to work: Irregular travel mode to work vs passive travel to work	Physical activity	42320.00	-0.02	0.14	0.86	0.09	0.29	-	-	-
Time spent in a week in moderately steneous activities	Physical activity	21827.00	-0.29	0.07		0.00	0.48	-	-	-
Sedentary or standing work	Physical activity	63433.00	0.01	0.09	0.89	0.00	0.88	-	-	-
Light but partly physically active work	Physical activity	63433.00	0.01	0.10	0.93	0.02	0.31	-	-	-
Light and physically active work	Physical activity	63433.00	0.11	0.09	0.21	0.00	0.75	-	-	-
Sometimes physically straining work	Physical activity	63433.00	-0.02	0.09	0.85	0.00	0.72	-	-	-
Frequency of walking during leisure time	Physical activity	63002.00	0.15	0.04	0.00	0.00	0.97	0.18	0.57	17.00
Frequency of cycling during leisure time	Physical activity	53427.00	-0.05	0.04	0.28	0.00	0.74	-	-	-
Frequency of dancing during leisure time	Physical activity	37660.00	-0.15	0.05	0.00	0.00	0.91	-	-	-
Frequency of shoveling snow during leisure time	Physical activity	40756.00	-0.03	0.05	0.59	0.03	0.31	-	-	-
Frequency of gardening during leisure time	Physical activity	40515.00	0.00	0.05	0.93	0.25	0.25	-	-	-
Frequency of hunting or fishing during leisure time	Physical activity	39467.00	0.26	0.06	0.00	0.00	0.96	0.16	0.57	29.00
Frequency of picking berries or mushrooms during leisure time	Physical activity	40325.00	0.18	0.05	0.00	0.00	0.42	-	-	-
Changed everyday exercise during the last year	Physical activity	42927.00	0.10	0.05	0.03	0.00	0.33	-	-	-
Everyday exercise satisfaction	Physical activity	42802.00	0.07	0.05	0.18	0.75	0.05	-	-	-
Exercise during the last three months	Physical activity	64680.00	-0.15	0.04	0.00	0.00	0.86	0.18	0.57	18.00
If you exercise, change in exercise habits during the last year	Physical activity	36908.00	0.07	0.05	0.18	0.54	0.14	-	-	-
Amount of exercise during the last 12 months	Physical activity	22523.00	-0.40	0.07		0.00	0.76	-	-	-
Bregott on bread	Food	57944.00	-0.04	0.04	0.37	0.00	0.37	-	-	-

Whole grain crisp bread	Food	57944.00	0.19	0.04	0.00	0.00	0.49	0.19	0.59	6.00
Whole grain soft bread	Food	57944.00	-0.02	0.04	0.64	0.03	0.31	-	-	-
White (soft) bread, thin crisp bread	Food	57944.00	0.01	0.04	0.83	0.21	0.26	-	-	-
Coffee rolls/buns, rusk	Food	57944.00	0.07	0.04	0.08	0.00	0.96	-	-	-
Cheese 28%	Food	57944.00	-0.08	0.04	0.04	0.00	0.88	-	-	-
Cheese 10-17%	Food	57944.00	0.07	0.04	0.08	0.58	0.12	-	-	-
Soft cheese	Food	13644.00	0.03	0.08		0.00	0.32	-	-	-
Soft whey cheese	Food	13644.00	-0.05	0.08		0.80	0.03	-	-	-
Sausage, liver pate on bread	Food	57944.00	0.10	0.04	0.01	0.00	0.34	-	-	-
Meat on bread	Food	57944.00	0.09	0.04	0.02	0.00	0.45	-	-	-
Butter on bread	Food	57944.00	0.05	0.04	0.22	0.00	0.53	-	-	-
Oatflake, whole wheat, rye or barley porridge	Food	57944.00	-0.13	0.04	0.00	0.55	0.13	-	-	-
Rosehip, sweet syrup soup	Food	57944.00	0.07	0.04	0.06	0.71	0.06	-	-	-
Sour milk, yoghurt (3% fat)	Food	57944.00	-0.09	0.04	0.03	0.00	0.64	-	-	-
Sour milk, yoghurt (low fat)	Food	57944.00	0.02	0.04	0.54	0.00	0.43	-	-	-
Fiber cereals	Food	57944.00	-0.13	0.04	0.00	0.00	0.40	-	-	-
Corn flakes	Food	57944.00	-0.09	0.04	0.03	0.00	0.63	-	-	-
Berries (fresh or frozen)	Food	57944.00	-0.07	0.04	0.08	0.00	0.94	-	-	-
Apple, pear, peach, orange, mandarin and grapefruit	Food	57944.00	-0.04	0.04	0.36	0.00	0.36	-	-	-
Ba	Food	57944.00	0.04	0.04	0.30	0.00	0.80	-	-	-
Root vegetables and carrot	Food	57944.00	-0.10	0.04	0.02	0.46	0.17	-	-	-
Low fat margarine on bread	Food	57944.00	0.09	0.04	0.02	0.00	0.66	-	-	-
Tomato and cucumber	Food	57944.00	-0.06	0.04	0.14	0.00	0.92	-	-	-
White cabbage, lettuce, lettuce cabbage, spich, borecole and broccoli	Food	57944.00	-0.13	0.04	0.00	0.00	0.63	-	-	-
Mixed frozen vegetables	Food	13644.00	0.10	0.08		0.01	0.31	-	-	-
Boiled or baked potato	Food	57944.00	0.16	0.04	0.00	0.40	0.20	-	-	-
Fried potatoes and pommes frites	Food	57944.00	0.01	0.04	0.73	0.00	0.70	-	-	-
Mashed potato	Food	13644.00	0.04	0.08		0.00	0.82	-	-	-
Potato salad	Food	10945.00	0.12	0.09		0.00	0.58	-	-	-
Rice	Food	57944.00	-0.10	0.04	0.02	0.78	0.03	-	-	-
Pasta	Food	57944.00	-0.12	0.04	0.01	0.00	0.54	-	-	-
Brown beans and pea soup	Food	57944.00	-0.04	0.04	0.35	0.00	0.56	-	-	-
Margarine on bread	Food	57944.00	0.01	0.04	0.76	0.00	0.80	-	-	-
Blota (broth + bread)	Food	13644.00	0.14	0.08		0.46	0.17	-	-	-
Pancake, waffle and Swedish dumpling	Food	57944.00	-0.09	0.04	0.03	0.00	0.49	-	-	-
Pizza	Food	57944.00	0.09	0.04	0.04	0.00	0.71	-	-	-
Minced meat dishes	Food	57944.00	-0.03	0.04	0.46	0.00	0.46	-	-	-
Meat stew	Food	57944.00	-0.02	0.04	0.59	0.38	0.21	-	-	-
Steak, chop, etc.	Food	57944.00	0.03	0.04	0.48	0.00	0.48	-	-	-
Bacon	Food	57944.00	-0.08	0.04	0.04	0.00	0.73	-	-	-
Sausage as main dish	Food	57944.00	-0.05	0.04	0.18	0.12	0.29	-	-	-
Hamburger	Food	57944.00	0.02	0.04	0.72	0.00	0.60	-	-	-
White meat (poultry)	Food	57944.00	-0.12	0.04	0.00	0.00	0.77	-	-	-
Butter for cooking	Food	57944.00	-0.05	0.04	0.23	0.64	0.10	-	-	-
Blood based food	Food	13644.00	-0.03	0.08		0.00	0.75	-	-	-
Liver and kidney	Food	13644.00	0.00	0.08		0.00	0.75	-	-	-
Lean fish (e.g. perch, bass, cod)	Food	57944.00	-0.04	0.04	0.38	0.74	0.05	-	-	-
Fatty fish (e.g. herring, whitefish, salmon)	Food	57944.00	-0.07	0.04	0.09	0.00	0.85	-	-	-
Shellfish (e.g. shrimps, scallops)	Food	13644.00	-0.02	0.08		0.00	0.57	-	-	-
Salty fish	Food	57944.00	0.02	0.04	0.63	0.00	0.66	-	-	-
Smoked fish/meat	Food	57944.00	-0.05	0.04	0.19	0.00	0.36	-	-	-
Ice cream	Food	57944.00	-0.10	0.04	0.02	0.45	0.18	-	-	-
Sweets	Food	57944.00	0.02	0.04	0.57	0.20	0.26	-	-	-
Sugar, honey, marmelade and jam	Food	57944.00	-0.12	0.04	0.00	0.00	0.82	-	-	-
Margarine for cooking	Food	57944.00	0.10	0.04	0.02	0.00	0.99	-	-	-
Cookies and pastry	Food	57944.00	-0.05	0.04	0.25	0.00	0.45	-	-	-

Chips, popcorn and salted nuts	Food	57944.00	-0.01	0.04	0.85	0.00	0.89	-	-	-
Low fat milk (0.5%)	Beverage	57944.00	0.05	0.04	0.24	0.00	0.47	-	-	-
Milk, sour milk (1.5%)	Beverage	57944.00	-0.11	0.04	0.01	0.47	0.17	-	-	-
Milk, sour milk (3%)	Beverage	57944.00	-0.02	0.04	0.70	0.30	0.23	-	-	-
Sodas, soft drinks and juice	Beverage	57944.00	0.12	0.04	0.00	0.00	0.96	-	-	-
Brewed (filtered) coffee	Beverage	57944.00	-0.26	0.04	0.00	0.08	0.30	0.19	0.59	4.00
Boiled coffee	Beverage	57944.00	0.05	0.04	0.22	0.00	0.75	-	-	-
Tea	Beverage	57944.00	-0.03	0.04	0.44	0.30	0.23	-	-	-
Light beer	Alcohol	57944.00	-0.03	0.04	0.49	0.51	0.15	-	-	-
Oil for cooking	Food	57944.00	-0.08	0.04	0.06	0.00	0.98	-	-	-
Medium beer	Alcohol	57944.00	-0.02	0.04	0.59	0.00	0.63	-	-	-
Strong beer	Alcohol	57944.00	0.18	0.04	0.00	0.00	0.41	-	-	-
Wine	Alcohol	57944.00	0.03	0.04	0.55	0.41	0.19	-	-	-
Liquor and spirits	Alcohol	57944.00	0.08	0.04	0.05	0.42	0.19	-	-	-
Salad dressing with oil	Food	57944.00	-0.12	0.04	0.00	0.00	0.80	-	-	-
Cream, creme fraiche, sour cream	Food	57944.00	-0.11	0.04	0.01	0.00	0.39	-	-	-
Average portion size of vegetables based on photographic illustration of four sizes (smallest to largest)	Food	57944.00	-0.31	0.04	0.00	0.00	0.92	0.18	0.58	27.00
Overall state of health during the last year	General health	65467.00	-0.41	0.04	0.00	0.00	0.91	0.19	0.57	13.00
Overall state of health compared to others your age	General health	32576.00	-0.49	0.06	0.00	0.74	0.05	0.16	0.54	30.00
Parents or siblings had a cerebral hemorrhage/thrombosis or cardiac infarction before the age of 60	General health	64385.00	1.24	0.11	0.00	0.00	0.98	0.19	0.57	7.00
Teetotaler	Alcohol	42207.00	0.43	0.18	0.01	0.00	0.35	-	-	-
Feel the need to reduce alcohol consumption	Alcohol	59448.00	0.60	0.12	0.00	0.00	0.85	0.19	0.57	10.00
Feel uneasy or guilty because of your way of drinking	Alcohol	38052.00	0.25	0.16	0.13	0.00	0.89	-	-	-
Frequency of alcohol consumption	Alcohol	22638.00	0.17	0.07		0.66	0.08	-	-	-
Amount of alcohol drunk in a day	Alcohol	21568.00	0.47	0.07		0.41	0.19	-	-	-
Frequency of drinking six or more glasses at the same occasion	Alcohol	21987.00	0.52	0.07		0.66	0.09	-	-	-
Times during last year that you felt guilty because of your drinking	Alcohol	21951.00	0.18	0.07		0.60	0.11	-	-	-
Iron intake (mg/day)	Nutrients	57944.00	0.05	0.04	0.21	0.00	0.40	-	-	-
Iodine intake (ug/day)	Nutrients	57944.00	-0.22	0.04	0.00	0.00	0.43	0.19	0.59	5.00
Calcium intake (mg/day)	Nutrients	57944.00	-0.09	0.04	0.03	0.09	0.29	-	-	-
Potassium intake (mg/day)	Nutrients	57944.00	-0.04	0.04	0.41	0.00	0.93	-	-	-
Beta-carotene intake (mg/day)	Nutrients	57944.00	-0.10	0.04	0.02	0.34	0.22	-	-	-
Cholesterol intake (g/day)	Nutrients	57944.00	-0.11	0.04	0.01	0.00	0.79	-	-	-
Carbohydrates intake (g/day)	Nutrients	57944.00	0.07	0.04	0.10	0.00	0.94	-	-	-
Average portion size of meat/fish based on photographic illustration of four sizes (smallest to largest)	Food	57944.00	-0.19	0.05	0.00	0.00	0.52	-	-	-
Breakfast habits: Only coffee/tea for breakfast vs not breakfast at all	Food	3877.00	-0.40	0.39	0.31	0.00	0.37	-	-	-
Breakfast habits: Coffee/tea and wheat buns or rusk for breakfast vs not breakfast at all	Food	3166.00	-0.29	0.40	0.46	0.00	0.99	-	-	-

Breakfast habits: Porridge w/o sandwich for breakfast vs not breakfast at all	Food	8469.00	-0.66	0.30	0.03	0.07	0.30	-	-	-
Breakfast habits: Gruel w/o sandwich for breakfast vs not breakfast at all	Food	2935.00	0.48	0.47	0.31	0.00	0.80	-	-	-
Eat breakfast from 2000	Food	33588.00	-0.67	0.20	0.00	0.04	0.31	-	-	-
Enterodiol intake (ug/day)	Nutrients	57944.00	0.03	0.04	0.46	0.00	0.90	-	-	-
Enterolactone intake (ug/day)	Nutrients	57944.00	-0.03	0.04	0.48	0.00	0.71	-	-	-
Equol intake (ug/day)	Nutrients	57944.00	0.05	0.04	0.21	0.00	0.90	-	-	-
Lariciresinol intake (ug/day)	Nutrients	57944.00	-0.04	0.04	0.38	0.00	0.87	-	-	-
Matairesinol intake (ug/day)	Nutrients	57944.00	0.06	0.04	0.17	0.00	0.44	-	-	-
Medioresinol intake (ug/day)	Nutrients	57944.00	0.11	0.04	0.01	0.03	0.31	-	-	-
Pinoresinol intake (ug/day)	Nutrients	57944.00	-0.01	0.04	0.75	0.00	0.71	-	-	-
Secoisolariciresinol intake (ug/day)	Nutrients	57944.00	-0.15	0.04	0.00	0.00	0.91	-	-	-
Sum of all ligns intake (ug/day)	Nutrients	57944.00	0.05	0.04	0.19	0.00	0.39	-	-	-
Syringaresinol intake (ug/day)	Nutrients	57944.00	0.08	0.04	0.05	0.11	0.29	-	-	-
Sum of Lariciresinol, Matairesinol, Pinoresinol, Secoisolariciresinol intake (ug/day)	Nutrients	57944.00	-0.04	0.04	0.38	0.00	0.77	-	-	-
Satisfaction with home and family situation	Psychosocial	48388.00	0.12	0.05	0.01	0.00	0.94	-	-	-
Appetite status	Psychosocial	48378.00	-0.02	0.05	0.64	0.00	0.38	-	-	-
Mood status	Psychosocial	48343.00	0.10	0.05	0.03	0.43	0.19	-	-	-
Energy status	Psychosocial	48312.00	-0.13	0.05	0.00	0.87	0.01	-	-	-
Patience status	Psychosocial	48351.00	-0.02	0.05	0.67	0.00	0.38	-	-	-
Confidence status	Psychosocial	48325.00	-0.04	0.05	0.38	0.30	0.23	-	-	-
Sleep status	Sleep	48421.00	-0.12	0.05	0.01	0.70	0.07	-	-	-
Do you feel important and appreciated outside your home?	Psychosocial	48414.00	0.10	0.05	0.04	0.00	0.53	-	-	-
Do you feel important and appreciated in your home?	Psychosocial	48056.00	0.15	0.05	0.00	0.00	0.56	-	-	-
Satisfaction with accomodation	Psychosocial	48430.00	0.19	0.05	0.00	0.00	0.34	-	-	-
Satisfaction with work situation	Psychosocial	47788.00	-0.02	0.05	0.63	0.40	0.20	-	-	-
Satisfaction with economy	Psychosocial	48378.00	0.26	0.05	0.00	0.00	0.32	0.18	0.60	22.00
Satisfaction with leisure time	Psychosocial	48316.00	0.13	0.05	0.01	0.00	0.44	-	-	-
Hearing status	General health	48413.00	0.09	0.05	0.08	0.00	0.89	-	-	-
Vision status	General health	48351.00	-0.11	0.05	0.02	0.56	0.13	-	-	-
Memory status	Psychosocial	48280.00	0.20	0.05	0.00	0.00	0.40	-	-	-
Fitness status	Physical activity	48347.00	-0.42	0.05	0.00	0.00	0.62	0.18	0.60	20.00
Magnesium intake (mg/day)	Nutrients	57944.00	-0.13	0.04	0.00	0.00	0.83	-	-	-
Saturated fat intake (g/day)	Nutrients	57944.00	-0.08	0.04	0.07	0.00	0.46	-	-	-
Monounsaturated fat intake (g/day)	Nutrients	57944.00	-0.13	0.04	0.00	0.00	0.82	-	-	-
Monosaccharides intake (g/day)	Nutrients	57944.00	-0.01	0.04	0.79	0.00	0.61	-	-	-
Sodium intake (mg/day)	Nutrients	57942.00	-0.13	0.04	0.00	0.45	0.18	-	-	-
Vitamin B3 intake (mg/day)	Nutrients	57944.00	-0.11	0.04	0.01	0.00	0.35	-	-	-
Cambridge physical activity index	Physical activity	62634.00	-0.16	0.04	0.00	0.49	0.16	-	-	-
Polyunsaturated fat intake (g/day)	Nutrients	57944.00	-0.07	0.04	0.10	0.00	0.95	-	-	-

Average portion size of potatoes/rice/pasta based on photographic illustration of four sizes (smallest to largest)	Food	57944.00	-0.34	0.05	0.00	0.03	0.31	0.18	0.58	26.00
Total protein intake (g/day)	Nutrients	57944.00	-0.09	0.04	0.02	0.00	0.87	-	-	-
Animal based protein intake (g/day)	Nutrients	57944.00	-0.09	0.04	0.02	0.00	0.89	-	-	-
Plant based protein intake (g/day)	Nutrients	57944.00	0.02	0.04	0.58	0.00	0.99	-	-	-
Vitamin A intake (mg/day)	Nutrients	57944.00	0.03	0.04	0.50	0.00	0.96	-	-	-
Sucrose intake (g/day)	Nutrients	57944.00	0.02	0.04	0.68	0.00	0.63	-	-	-
Cohabitation: Live alone vs Only one adult (spouse, partner)	Social	30057.00	-0.20	0.15	0.19	0.00	0.57	-	-	-
Cohabitation: Live alone vs Only children	Social	9769.00	-1.11	0.24	0.00	0.00	0.99	0.20	0.56	2.00
Cohabitation: Live alone vs Adult and children	Social	38134.00	-1.10	0.15	0.00	0.00	0.90	0.19	0.56	12.00
Cohabitation: Live alone vs Other/others	Social	7564.00	-0.41	0.39	0.29	0.00	0.49	-	-	-
Selenium intake (ug/day)	Nutrients	57944.00	-0.16	0.04	0.00	0.00	0.79	-	-	-
Self rate of overall health	General health	28571.00	-0.82	0.06	0.00	0.00	0.77	0.18	0.59	25.00
For how much of the time during the last four weeks has your physical health or your emotiol problems limited your ability to interact with others?	General health	28334.00	-0.03	0.06	0.61	0.00	0.44	-	-	-
Get sick more often than other people	General health	28458.00	0.24	0.06	0.00	0.71	0.06	-	-	-
As healthy as anyone	General health	28373.00	-0.42	0.06	0.00	0.80	0.02	0.18	0.59	28.00
Worsen in health in the future	General health	28365.00	0.23	0.06	0.00	0.00	0.52	-	-	-
Excellent health	General health	28460.00	-0.80	0.06	0.00	0.52	0.15	0.18	0.59	23.00
Self rate of overall health compared to a year ago	General health	28331.00	-0.18	0.06	0.00	0.00	0.45	-	-	-
Physical limitation to participate in steneous activities: running, lifting heavy objects, taking part in physically demanding sports	General health	28518.00	0.29	0.06	0.00	0.00	0.38	-	-	-
Physical limitation to participate in moderately demanding activities: moving a table, vacuuming, walking in the forest or gardening	General health	28508.00	0.13	0.06	0.04	0.70	0.07	-	-	-
Physical limitation to participate in moderately demanding activities: lifting or carrying grocery bags	General health	28487.00	0.09	0.06	0.15	0.00	0.64	-	-	-
Physical limitation to participate in moderately demanding activities: walking up several stairs	General health	28498.00	0.27	0.06	0.00	0.37	0.21	-	-	-
Physical limitation to participate in moderately demanding activities: bending down or kneeling	General health	28466.00	0.14	0.06	0.03	0.78	0.03	-	-	-
Physical limitation to participate in moderately demanding activities: walking more than two km	General health	28509.00	0.13	0.06	0.05	0.00	0.54	-	-	-

Physical limitation that reduced the normal time spent at work or in other activities during the last four weeks	General health	28534.00	0.48	0.19	0.01	0.66	0.09	-	-	-
Physical limitation that made you do less than you wanted during the last four weeks	General health	28518.00	0.34	0.15	0.03	0.57	0.13	-	-	-
Physical limitation that made you not being able to perform certain work tasks or other activities during the last four weeks	General health	28407.00	0.34	0.17	0.05	0.17	0.27	-	-	-
Physical limitation that limited your ability to perform certain work tasks or other activities during the last four weeks	General health	28440.00	0.28	0.18	0.11	0.00	0.53	-	-	-
Emotiol problems that made you do less than you wanted during the last four weeks	General health	28534.00	-0.40	0.18	0.03	0.63	0.10	-	-	-
Extent to what your physical and emotiol health disrupted your usual social life during the last four weeks	General health	28516.00	0.01	0.06	0.85	0.74	0.05	-	-	-
Pain during the last four weeks	General health	28478.00	0.21	0.06	0.00	0.81	0.02	-	-	-
How much has the pain during the last four weeks disturbed your normal work?	General health	28416.00	0.23	0.06	0.00	0.72	0.06	-	-	-
For how much of the time during the last four weeks have you felt really alert and strong?	General health	28428.00	-0.26	0.06	0.00	0.64	0.10	-	-	-
For how much of the time during the last four weeks have you felt very nervous?	General health	28475.00	0.07	0.06	0.26	0.00	0.37	-	-	-
For how much of the time during the last four weeks have you felt so depressed that nothing could cheer you up?	General health	28505.00	-0.10	0.06	0.09	0.06	0.30	-	-	-
For how much of the time during the last four weeks have you felt calm and serene?	General health	28481.00	-0.11	0.06	0.06	0.00	0.80	-	-	-
For how much of the time during the last four weeks have you felt full of energy?	General health	28445.00	-0.17	0.06	0.01	0.52	0.15	-	-	-
For how much of the time during the last four weeks have you felt gloomy and sad?	General health	28453.00	-0.18	0.06	0.00	0.00	0.47	-	-	-
For how much of the time during the last four weeks have you felt worn out?	General health	28410.00	0.09	0.06	0.15	0.00	0.91	-	-	-
For how much of the time during the last four weeks have you felt happy?	General health	28472.00	0.01	0.06	0.89	0.66	0.09	-	-	-
For how much of the time during the last four weeks have you felt tired?	General health	28498.00	0.12	0.06	0.06	0.39	0.20	-	-	-
Long-term sickness	General health	61524.00	-0.04	0.11	0.71	0.00	0.51	-	-	-
Work shifts/weekends	Psychosocial	63155.00	-0.08	0.09	0.37	0.00	0.75	-	-	-
Risk of sleeping while sitting and reading	Sleep	22121.00	-0.16	0.07		0.00	0.74	-	-	-

Risk of sleeping while watching TV	Sleep	22358.00	0.07	0.07		0.00	0.53	-	-	-
Risk of sleeping while sitting ictive in a public place	Sleep	22211.00	-0.26	0.07		0.00	0.51	-	-	-
Risk of sleeping as a passenger in a car for one hour without break	Sleep	22127.00	-0.38	0.07		0.00	0.91	-	-	-
Risk of sleeping while lying down resting in the afternoon	Sleep	22279.00	-0.06	0.07		0.49	0.16	-	-	-
Risk of sleeping while sitting still after having lunch	Sleep	22293.00	-0.25	0.07		0.00	0.73	-	-	-
Snore during sleep	Sleep	20871.00	0.47	0.07		0.00	0.50	-	-	-
Breath-holds during sleep	Sleep	15600.00	0.41	0.09		0.00	0.58	-	-	-
Number of cigarretes smoked per day (in groups)	Tobacco use	53191.00	-0.44	0.05	0.00	0.00	0.70	0.18	0.57	21.00
Years smoking	Tobacco use	57526.00	-0.57	0.05	0.00	0.15	0.28	0.18	0.57	16.00
Grams of tobacco smoked per week	Tobacco use	41534.00	-0.08	0.05	0.11	0.00	0.58	-	-	-
Number of cigarretes smoked per day	Tobacco use	42560.00	-0.22	0.05	0.00	0.00	0.87	0.19	0.57	3.00
Number of cigars smoked per day	Tobacco use	41498.00	-0.03	0.05	0.49	0.00	0.87	-	-	-
Smoking status: Smokers vs non-smokers	Tobacco use	42230.00	-1.68	0.14	0.00	0.00	1.00	0.18	0.57	19.00
Smoking status: Former smokers vs non-smokers	Tobacco use	46881.00	-0.76	0.12	0.00	0.50	0.16	0.19	0.58	9.00
Smoking status: Former occasiol smokers vs non-smokers	Tobacco use	39104.00	-0.48	0.15	0.00	0.00	0.82	-	-	-
Number of snuff boxes per week	Tobacco use	63687.00	-0.19	0.05	0.00	0.00	0.42	-	-	-
Snuff status: Snuff users vs non-snuff users	Tobacco use	56226.00	-0.63	0.13	0.00	0.00	0.47	0.18	0.57	24.00
Snuff status: Former snuff users vs non-snuff users	Tobacco use	52608.00	0.04	0.15	0.78	0.00	0.51	-	-	-
Years using snuff	Tobacco use	61954.00	0.07	0.05	0.20	0.00	0.86	-	-	-
Participation in associations or voluntary organisations	Social	65100.00	-0.36	0.08	0.00	0.00	0.95	0.18	0.57	15.00
Participation in sports or physical exercise associations	Social	20973.00	-0.50	0.14	0.00	0.00	0.62	-	-	-
Participation in study circles	Social	20973.00	-0.23	0.20	0.25	0.61	0.11	-	-	-
Participation in other association	Social	20973.00	0.10	0.14	0.51	0.05	0.30	-	-	-
People to ask for help apart from the ones at home	Social	65854.00	0.14	0.13	0.26	0.00	0.84	-	-	-
Number of friends that can come to your home at any time and feel at home	Social	65230.00	0.06	0.04	0.13	0.00	0.38	-	-	-
Number of social contacts with the same interests as you	Social	65939.00	0.02	0.04	0.57	0.00	0.51	-	-	-
Would you say that the number of people that you meet in your everyday life is enough or would you like to meet more or fewer people?	Social	66049.00	-0.14	0.04	0.00	0.00	0.75	-	-	-
Close relationship with anyone	Social	65367.00	0.00	0.04	0.93	0.19	0.27	-	-	-
Frequency of engaging in clubs, associations or study circles	Social	41695.00	-0.12	0.05	0.01	0.00	0.80	-	-	-

Number of social interactions during a normal week	Social	65434.00	0.13	0.04	0.00	0.00	0.98	-	-	-
Support from others	Social	66099.00	0.06	0.04	0.10	0.43	0.18	-	-	-
Number of people with whom you can speak openly	Social	66013.00	0.13	0.04	0.00	0.00	0.80	-	-	-
Receive hugs to comfort and support you	Social	64950.00	-0.21	0.11	0.05	0.00	0.54	-	-	-
Stigmasterol intake (mg/day)	Nutrients	57944.00	-0.02	0.04	0.61	0.00	0.43	-	-	-
Tiamin intake (mg/day)	Nutrients	57944.00	-0.10	0.04	0.02	0.00	0.96	-	-	-
Vitamin E intake (mg/day)	Nutrients	57944.00	-0.05	0.04	0.24	0.00	0.83	-	-	-
Trans fat intake (g/day)	Nutrients	57944.00	-0.05	0.06	0.37	0.00	0.77	-	-	-
Sum of phytosterols intake (mg/day)	Nutrients	57944.00	-0.02	0.04	0.65	0.00	0.90	-	-	-
Zinc intake (mg/day)	Nutrients	57944.00	-0.08	0.04	0.07	0.00	0.53	-	-	-

Supplementary Table 6. Linear mixed model association results for total cholesterol

Description	Group	N	Effect estimate	S.E.	p-value	I^2	Q p-value	marginal R2	conditional R2	R2 rank
Alcohol intake (g/day)	Alcohol	54570.00	0.04	0.00	0.00	0.16	0.28	0.10	0.59	15.00
Permanent employment	Psychosocial	50783.00	0.04	0.01	0.00	0.00	0.72	0.08	0.55	67.00
Self-employed	Psychosocial	50783.00	-0.05	0.02	0.00	0.00	0.93	-	-	-
Distance to work in kilometers (one way)	Physical activity	51557.00	0.01	0.00	0.01	0.62	0.10	-	-	-
Last time a colleague visited you at home	Psychosocial	58009.00	0.02	0.00	0.00	0.69	0.07	-	-	-
Job demands to work very fast	Psychosocial	58828.00	0.00	0.00	0.85	0.44	0.18	-	-	-
Frequency of social contacts with colleagues during leisure time	Psychosocial	57149.00	0.01	0.00	0.00	0.00	0.78	-	-	-
High physical demand from job	Physical activity	58970.00	0.01	0.00	0.03	0.80	0.02	-	-	-
Enough time for job assignments	Psychosocial	58558.00	0.01	0.00	0.02	0.00	0.62	-	-	-
Control over planning and execution of the workday	Psychosocial	58847.00	0.01	0.00	0.13	0.55	0.14	-	-	-
Ingenuity or creativity demand from job	Psychosocial	58548.00	0.00	0.00	0.46	0.54	0.14	-	-	-
Frequent social contacts with colleagues during work	Psychosocial	58194.00	-0.01	0.00	0.20	0.00	0.36	-	-	-
Contradictory demands in job	Psychosocial	58439.00	0.00	0.00	0.47	0.00	0.32	-	-	-
Possibility to leave your work for a while to speak with a colleague	Psychosocial	58239.00	0.01	0.00	0.14	0.00	0.74	-	-	-
Learn new things at job	Psychosocial	58693.00	0.01	0.00	0.02	0.00	0.58	-	-	-
High mental demand from job	Psychosocial	58368.00	0.01	0.00	0.00	0.00	0.59	-	-	-
Repetitive job	Psychosocial	58725.00	-0.01	0.00	0.14	0.68	0.08	-	-	-
Skill demand from job	Psychosocial	58655.00	0.00	0.00	0.40	0.60	0.11	-	-	-
Possibility to speak with colleagues during breaks	Psychosocial	58601.00	0.00	0.00	0.25	0.33	0.22	-	-	-
Control over own work assignment	Psychosocial	58825.00	0.00	0.00	0.71	0.00	0.75	-	-	-
Vitamin C intake (mg/day)	Nutrients	54570.00	-0.05	0.00	0.00	0.14	0.28	0.10	0.59	8.00
Vitamin B12 intake (ug/day)	Nutrients	54570.00	0.01	0.00	0.14	0.61	0.11	-	-	-
Vitamin B2 intake (ug/day)	Nutrients	54570.00	-0.02	0.00	0.00	0.00	0.57	0.10	0.59	39.00
Vitamin B6 intake (mg/day)	Nutrients	54570.00	-0.01	0.00	0.11	0.00	0.81	-	-	-
Informed of having high blood pressure	General health	59708.00	0.03	0.01	0.01	0.00	0.96	-	-	-
Beta-sitostanol intake (mg/day)	Nutrients	54570.00	0.02	0.00	0.00	0.00	0.54	-	-	-
Beta-sitosterol intake (mg/day)	Nutrients	54570.00	0.00	0.00	0.89	0.00	0.46	-	-	-
Marital status: Single vs Married/partner	Social	54575.00	-0.02	0.02	0.19	0.00	0.51	-	-	-
Marital status: Single vs Divorced/separated	Social	9930.00	-0.07	0.02	0.00	0.49	0.16	-	-	-
Marital status: Single vs Widow/widower	Social	6232.00	0.01	0.05	0.87	0.00	0.86	-	-	-
Campestanol intake (mg/day)	Nutrients	54570.00	0.01	0.00	0.08	0.00	0.59	-	-	-
Campesterol intake (mg/day)	Nutrients	54570.00	0.02	0.00	0.00	0.00	0.90	0.10	0.59	35.00
Parents or siblings have diabetes	General health	58671.00	-0.02	0.01	0.18	0.88	0.00	-	-	-
Disaccharides intake (g/day)	Nutrients	54570.00	0.00	0.00	0.57	0.28	0.24	-	-	-
Vitamin D intake (ug/day)	Nutrients	54570.00	0.03	0.00	0.00	0.00	0.46	0.10	0.59	20.00
Total energy intake (kcal/day)	Nutrients	54570.00	-0.03	0.01	0.00	0.00	0.40	0.10	0.59	60.00
Formic acid intaje (g/day)	Nutrients	54570.00	0.05	0.00	0.00	0.00	0.62	0.10	0.59	7.00
Pentadecanoic acid intake (g/day)	Nutrients	54570.00	0.04	0.00	0.00	0.00	0.44	0.10	0.59	16.00
Palmitic acid intake (g/day)	Nutrients	54570.00	0.06	0.00	0.00	0.00	0.91	0.10	0.59	2.00

Heptadecanoic acid intake (g/day)	Nutrients	54570.00	0.04	0.00	0.00	0.00	0.44	0.10	0.59	17.00
Linoleic acid intake (g/day)	Nutrients	54570.00	0.01	0.00	0.04	0.00	0.69	-	-	-
Linolenic acid intake (g/day)	Nutrients	54570.00	0.02	0.00	0.00	0.00	1.00	0.10	0.59	45.00
Arachidonic acid (ARA) intake (g/day)	Nutrients	54568.00	0.02	0.00	0.00	0.73	0.05	-	-	-
Eicosapentaenoic acid (EPA) intake (g/day)	Nutrients	54569.00	0.02	0.00	0.00	0.00	0.32	0.10	0.59	37.00
Docosahexaenoic acid (DHA) intake (g/day)	Nutrients	54570.00	0.03	0.00	0.00	0.00	0.32	0.10	0.59	31.00
Fat intake (g/day)	Nutrients	54570.00	0.04	0.00	0.00	0.00	0.67	0.10	0.59	12.00
Fibre intake (g/day)	Nutrients	54570.00	-0.05	0.00	0.00	0.00	0.44	0.10	0.59	9.00
Folic acid intake (ug/day)	Nutrients	54570.00	-0.02	0.00	0.00	0.00	0.78	0.10	0.59	54.00
Phosphate intake (mg/day)	Nutrients	54570.00	0.00	0.00	0.95	0.00	0.60	-	-	-
Whole grain intake (g/day)	Food	54570.00	-0.01	0.00	0.16	0.56	0.13	-	-	-
Travel to work: Walk to work vs passive travel to work	Physical activity	38591.00	0.02	0.02	0.19	0.00	0.58	-	-	-
Travel to work: Cycle to work vs passive travel to work	Physical activity	44950.00	-0.04	0.01	0.00	0.00	0.88	-	-	-
Travel to work: Irregular travel mode to work vs passive travel to work	Physical activity	38578.00	0.00	0.02	0.91	0.34	0.22	-	-	-
Time spent in a week in moderately steneous activities	Physical activity	21614.00	-0.01	0.01		0.00	0.69	-	-	-
Sedentary or standing work	Physical activity	57924.00	-0.02	0.01	0.09	0.00	0.97	-	-	-
Light but partly physically active work	Physical activity	57924.00	-0.01	0.01	0.49	0.00	0.92	-	-	-
Light and physically active work	Physical activity	57924.00	0.00	0.01	0.86	0.13	0.28	-	-	-
Sometimes physically straining work	Physical activity	57924.00	0.02	0.01	0.02	0.50	0.16	-	-	-
Frequency of walking during leisure time	Physical activity	57551.00	0.00	0.00	0.30	0.52	0.15	-	-	-
Frequency of cycling during leisure time	Physical activity	48823.00	-0.02	0.00	0.00	0.00	0.66	-	-	-
Frequency of dancing during leisure time	Physical activity	33347.00	-0.02	0.01	0.00	0.24	0.25	-	-	-
Frequency of shoveling snow during leisure time	Physical activity	35738.00	0.01	0.01	0.02	0.00	0.65	-	-	-
Frequency of gardening during leisure time	Physical activity	35525.00	0.02	0.01	0.00	0.15	0.28	-	-	-
Frequency of hunting or fishing during leisure time	Physical activity	34803.00	0.06	0.01	0.00	0.59	0.12	0.10	0.57	64.00
Frequency of picking berries or mushrooms during leisure time	Physical activity	35365.00	0.05	0.01	0.00	0.00	0.86	0.10	0.57	65.00
Changed everyday exercise during the last year	Physical activity	37400.00	-0.02	0.01	0.00	0.00	0.42	-	-	-
Everyday exercise satisfaction	Physical activity	37290.00	0.00	0.01	0.53	0.00	0.82	-	-	-
Exercise during the last three months	Physical activity	59067.00	-0.03	0.00	0.00	0.00	0.99	0.07	0.55	72.00
If you exercise, change in exercise habits during the last year	Physical activity	32971.00	-0.03	0.01	0.00	0.00	0.89	0.10	0.57	61.00
Amount of exercise during the last 12 months	Physical activity	22298.00	-0.03	0.01		0.00	0.51	-	-	-
Bregott on bread	Food	54570.00	0.04	0.00	0.00	0.00	0.44	0.10	0.59	19.00
Whole grain crisp bread	Food	54570.00	0.02	0.00	0.00	0.00	0.67	0.10	0.59	46.00
Whole grain soft bread	Food	54570.00	0.00	0.00	0.73	0.00	0.44	-	-	-
White (soft) bread, thin crisp bread	Food	54570.00	0.01	0.00	0.00	0.00	0.93	-	-	-
Coffee rolls/buns, rusk	Food	54570.00	0.00	0.00	0.71	0.50	0.16	-	-	-
Cheese 28%	Food	54570.00	0.00	0.00	0.45	0.00	0.90	-	-	-
Cheese 10-17%	Food	54570.00	-0.01	0.00	0.01	0.00	0.83	-	-	-
Soft cheese	Food	10687.00	-0.01	0.01		0.93	0.00	-	-	-
Soft whey cheese	Food	10687.00	-0.03	0.01		0.00	0.65	-	-	-
Sausage, liver pate on bread	Food	54570.00	0.00	0.00	0.69	0.00	0.60	-	-	-

Meat on bread	Food	54570.00	0.00	0.00	0.65	0.04	0.31	-	-	-
Butter on bread	Food	54570.00	0.01	0.00	0.04	0.00	0.84	-	-	-
Oatflake, whole wheat, rye or barley porridge	Food	54570.00	-0.03	0.00	0.00	0.00	0.80	0.10	0.59	25.00
Rosehip, sweet syrup soup	Food	54570.00	-0.02	0.00	0.00	0.67	0.08	-	-	-
Sour milk, yoghurt (3% fat)	Food	54570.00	-0.01	0.00	0.02	0.04	0.31	-	-	-
Sour milk, yoghurt (low fat)	Food	54570.00	-0.02	0.00	0.00	0.00	0.96	0.10	0.59	44.00
Fiber cereals	Food	54570.00	-0.03	0.00	0.00	0.00	0.50	0.10	0.59	18.00
Corn flakes	Food	54570.00	-0.01	0.00	0.01	0.00	0.93	-	-	-
Berries (fresh or frozen)	Food	54570.00	-0.01	0.00	0.10	0.00	0.90	-	-	-
Apple, pear, peach, orange, mandarin and grapefruit	Food	54570.00	-0.01	0.00	0.00	0.06	0.30	-	-	-
Ba	Food	54570.00	-0.04	0.00	0.00	0.00	0.38	0.10	0.59	11.00
Root vegetables and carrot	Food	54570.00	-0.02	0.00	0.00	0.00	0.69	0.10	0.59	50.00
Low fat margarine on bread	Food	54570.00	-0.03	0.00	0.00	0.00	0.75	0.10	0.59	41.00
Tomato and cucumber	Food	54570.00	-0.02	0.00	0.00	0.00	0.90	0.10	0.59	53.00
White cabbage, lettuce, lettuce cabbage, spich, borecole and broccoli	Food	54570.00	-0.01	0.00	0.16	0.00	0.61	-	-	-
Mixed frozen vegetables	Food	10687.00	0.00	0.01		0.61	0.11	-	-	-
Boiled or baked potato	Food	54570.00	0.02	0.00	0.00	0.00	0.54	-	-	-
Fried potatoes and pommes frites	Food	54570.00	0.02	0.00	0.00	0.00	0.83	0.10	0.59	38.00
Mashed potato	Food	10687.00	0.00	0.01		0.42	0.19	-	-	-
Potato salad	Food	10687.00	0.01	0.01		0.00	0.99	-	-	-
Rice	Food	54570.00	-0.02	0.00	0.00	0.00	0.79	0.10	0.59	55.00
Pasta	Food	54570.00	-0.01	0.00	0.01	0.00	0.88	-	-	-
Brown beans and pea soup	Food	54570.00	-0.01	0.00	0.19	0.00	0.51	-	-	-
Margarine on bread	Food	54570.00	-0.01	0.00	0.01	0.00	0.89	-	-	-
Blota (broth + bread)	Food	10687.00	0.03	0.01		0.00	0.47	-	-	-
Pancake, waffle and Swedish dumpling	Food	54570.00	0.01	0.00	0.04	0.00	0.59	-	-	-
Pizza	Food	54570.00	-0.01	0.00	0.14	0.41	0.19	-	-	-
Minced meat dishes	Food	54570.00	0.01	0.00	0.00	0.00	0.69	-	-	-
Meat stew	Food	54570.00	0.03	0.00	0.00	0.00	0.45	0.10	0.59	28.00
Steak, chop, etc.	Food	54570.00	0.02	0.00	0.00	0.83	0.02	-	-	-
Bacon	Food	54570.00	0.03	0.00	0.00	0.00	0.51	0.10	0.59	22.00
Sausage as main dish	Food	54570.00	0.01	0.00	0.01	0.18	0.27	-	-	-
Hamburger	Food	54570.00	0.00	0.00	0.93	0.00	0.92	-	-	-
White meat (poultry)	Food	54570.00	0.00	0.00	0.33	0.00	0.64	-	-	-
Butter for cooking	Food	54570.00	0.03	0.00	0.00	0.00	0.58	0.10	0.59	30.00
Blood based food	Food	10687.00	-0.02	0.01		0.36	0.21	-	-	-
Liver and kidney	Food	10687.00	-0.02	0.01		0.66	0.09	-	-	-
Lean fish (e.g. perch, bass, cod)	Food	54570.00	0.01	0.00	0.11	0.77	0.04	-	-	-
Fatty fish (e.g. herring, whitefish, salmon)	Food	54570.00	0.03	0.00	0.00	0.00	0.38	0.10	0.59	36.00
Shellfish (e.g. shrimps, scallops)	Food	10687.00	0.00	0.01		0.00	0.67	-	-	-
Salty fish	Food	54570.00	0.01	0.00	0.00	0.00	0.95	0.10	0.59	59.00
Smoked fish/meat	Food	54570.00	0.00	0.00	0.70	0.00	0.78	-	-	-
Ice cream	Food	54570.00	0.00	0.00	0.84	0.57	0.13	-	-	-
Sweets	Food	54570.00	0.01	0.00	0.00	0.00	0.97	-	-	-
Sugar, honey, marmelade and jam	Food	54570.00	0.02	0.00	0.00	0.00	0.66	0.10	0.59	58.00
Margarine for cooking	Food	54570.00	0.03	0.00	0.00	0.67	0.08	0.10	0.59	23.00
Cookies and pastry	Food	54570.00	0.00	0.00	0.49	0.00	0.52	-	-	-
Chips, popcorn and salted nuts	Food	54570.00	0.01	0.00	0.01	0.00	0.64	-	-	-
Low fat milk (0.5%)	Beverage	54570.00	-0.02	0.00	0.00	0.00	0.83	0.10	0.59	52.00
Milk, sour milk (1.5%)	Beverage	54570.00	0.01	0.00	0.07	0.00	0.50	-	-	-
Milk, sour milk (3%)	Beverage	54570.00	0.02	0.00	0.00	0.00	0.52	0.10	0.59	56.00
Sodas, soft drinks and juice	Beverage	54570.00	0.01	0.00	0.19	0.20	0.26	-	-	-
Brewed (filtered) coffee	Beverage	54570.00	-0.01	0.00	0.06	0.00	0.55	-	-	-
Boiled coffee	Beverage	54570.00	0.08	0.00	0.00	0.00	0.97	0.11	0.59	1.00
Tea	Beverage	54570.00	-0.04	0.00	0.00	0.00	1.00	0.10	0.59	10.00
Light beer	Alcohol	54570.00	0.01	0.00	0.02	0.00	0.73	-	-	-
Oil for cooking	Food	54570.00	0.00	0.00	0.83	0.00	0.67	-	-	-
Medium beer	Alcohol	54570.00	0.02	0.00	0.00	0.59	0.12	0.10	0.59	42.00
Strong beer	Alcohol	54570.00	0.02	0.00	0.00	0.00	0.63	0.10	0.59	33.00
Wine	Alcohol	54570.00	0.03	0.00	0.00	0.00	0.56	0.10	0.59	34.00

Liquor and spirits	Alcohol	54570.00	0.02	0.00	0.00	0.00	0.88	0.10	0.59	51.00
Salad dressing with oil	Food	54570.00	-0.01	0.00	0.00	0.00	0.82	-	-	-
Cream, creme fraiche, sour cream	Food	54570.00	0.03	0.00	0.00	0.00	0.33	0.10	0.59	27.00
Average portion size of vegetables based on photographic illustration of four sizes (smallest to largest)	Food	54570.00	-0.02	0.00	0.00	0.25	0.25	0.07	0.55	80.00
Overall state of health during the last year	General health	59691.00	-0.01	0.00	0.03	0.00	0.82	-	-	-
Overall state of health compared to others your age	General health	31463.00	-0.03	0.01	0.00	0.00	0.89	0.10	0.53	63.00
Parents or siblings had a cerebral hemorrhage/thrombosis or cardiac infarction before the age of 60	General health	58618.00	0.04	0.01	0.00	0.00	0.86	0.07	0.54	75.00
Teetotaler	Alcohol	37375.00	-0.05	0.02	0.02	0.00	0.49	-	-	-
Feel the need to reduce alcohol consumption	Alcohol	54979.00	0.05	0.01	0.00	0.69	0.07	-	-	-
Feel uneasy or guilty because of your way of drinking	Alcohol	33805.00	-0.01	0.02	0.68	0.68	0.08	-	-	-
Frequency of alcohol consumption	Alcohol	22414.00	0.05	0.01		0.66	0.09	-	-	-
Amount of alcohol drunk in a day	Alcohol	21355.00	0.05	0.01		0.09	0.29	-	-	-
Frequency of drinking six or more glasses at the same occasion	Alcohol	21769.00	0.06	0.01		0.00	0.38	-	-	-
Times during last year that you felt guilty because of your drinking	Alcohol	21734.00	0.02	0.01		0.00	0.37	-	-	-
Iron intake (mg/day)	Nutrients	54570.00	-0.03	0.00	0.00	0.00	0.42	0.10	0.59	24.00
Iodine intake (ug/day)	Nutrients	54570.00	0.01	0.00	0.18	0.00	0.61	-	-	-
Calcium intake (mg/day)	Nutrients	54570.00	-0.03	0.00	0.00	0.33	0.22	0.10	0.59	21.00
Potassium intake (mg/day)	Nutrients	54570.00	0.00	0.00	0.45	0.00	0.67	-	-	-
Beta-carotene intake (mg/day)	Nutrients	54570.00	-0.02	0.00	0.00	0.00	0.65	0.10	0.59	47.00
Cholesterol intake (g/day)	Nutrients	54570.00	0.04	0.00	0.00	0.00	0.43	0.10	0.59	14.00
Carbohydrates intake (g/day)	Nutrients	54570.00	-0.05	0.00	0.00	0.00	0.34	0.10	0.59	5.00
Average portion size of meat/fish based on photographic illustration of four sizes (smallest to largest)	Food	54570.00	0.03	0.00	0.00	0.00	0.46	0.07	0.55	77.00
Breakfast habits: Only coffee/tea for breakfast vs not breakfast at all	Food	3831.00	-0.17	0.04	0.00	0.00	0.46	-	-	-
Breakfast habits: Coffee/tea and wheat buns or rusk for breakfast vs not breakfast at all	Food	3117.00	-0.07	0.04	0.08	0.00	0.48	-	-	-
Breakfast habits: Porridge w/o sandwich for breakfast vs not breakfast at all	Food	8347.00	-0.28	0.03	0.00	0.00	0.40	0.08	0.60	66.00
Breakfast habits: Gruel w/o sandwich for breakfast vs not breakfast at all	Food	2902.00	-0.15	0.05	0.00	0.00	0.96	-	-	-
Eat breakfast from 2000	Food	33269.00	-0.16	0.02	0.00	0.24	0.25	0.10	0.56	62.00
Enterodiol intake (ug/day)	Nutrients	54570.00	-0.01	0.00	0.11	0.00	0.83	-	-	-
Enterolactone intake (ug/day)	Nutrients	54570.00	-0.01	0.00	0.00	0.00	0.66	-	-	-
Equol intake (ug/day)	Nutrients	54570.00	-0.03	0.00	0.00	0.00	0.86	0.10	0.59	29.00
Lariciresinol intake (ug/day)	Nutrients	54570.00	0.00	0.00	0.63	0.00	0.55	-	-	-
Matairesinol intake (ug/day)	Nutrients	54570.00	0.00	0.00	0.68	0.00	0.77	-	-	-
Medioresinol intake (ug/day)	Nutrients	54570.00	0.01	0.00	0.00	0.00	0.81	0.10	0.59	57.00

Pinoresinol intake (ug/day)	Nutrients	54570.00	-0.01	0.00	0.10	0.00	0.84	-	-	-
Secoisolariciresinol intake (ug/day)	Nutrients	54570.00	0.02	0.00	0.00	0.00	0.73	0.10	0.59	32.00
Sum of all ligns intake (ug/day)	Nutrients	54570.00	0.01	0.00	0.09	0.00	0.68	-	-	-
Syringaresinol intake (ug/day)	Nutrients	54570.00	0.01	0.00	0.05	0.00	0.66	-	-	-
Sum of Lariciresinol, Matairesinol, Pinoresinol, Secoisolariciresinol intake (ug/day)	Nutrients	54570.00	0.00	0.00	0.77	0.00	0.68	-	-	-
Satisfaction with home and family situation	Psychosocial	47930.00	0.02	0.00	0.00	0.00	0.39	-	-	-
Appetite status	Psychosocial	47921.00	0.01	0.00	0.06	0.53	0.14	-	-	-
Mood status	Psychosocial	47886.00	0.01	0.00	0.21	0.00	0.42	-	-	-
Energy status	Psychosocial	47854.00	-0.01	0.00	0.01	0.00	0.97	-	-	-
Patience status	Psychosocial	47893.00	0.00	0.00	0.85	0.00	0.45	-	-	-
Confidence status	Psychosocial	47867.00	0.00	0.00	0.93	0.43	0.19	-	-	-
Sleep status	Sleep	47964.00	-0.01	0.00	0.01	0.29	0.24	-	-	-
Do you feel important and appreciated outside your home?	Psychosocial	47956.00	0.00	0.00	0.68	0.00	0.80	-	-	-
Do you feel important and appreciated in your home?	Psychosocial	47602.00	0.01	0.00	0.06	0.00	0.87	-	-	-
Satisfaction with accomodation	Psychosocial	47971.00	0.01	0.00	0.19	0.23	0.25	-	-	-
Satisfaction with work situation	Psychosocial	47331.00	-0.01	0.00	0.00	0.00	0.89	-	-	-
Satisfaction with economy	Psychosocial	47918.00	0.00	0.00	0.80	0.00	0.62	-	-	-
Satisfaction with leisure time	Psychosocial	47857.00	0.00	0.00	0.57	0.00	0.89	-	-	-
Hearing status	General health	47954.00	0.00	0.00	0.55	0.00	0.86	-	-	-
Vision status	General health	47893.00	-0.01	0.00	0.20	0.00	0.99	-	-	-
Memory status	Psychosocial	47822.00	0.01	0.00	0.03	0.00	0.84	-	-	-
Fitness status	Physical activity	47890.00	-0.04	0.00	0.00	0.00	0.93	0.07	0.57	78.00
Magnesium intake (mg/day)	Nutrients	54570.00	0.00	0.00	0.30	0.00	0.51	-	-	-
Saturated fat intake (g/day)	Nutrients	54570.00	0.04	0.00	0.00	0.00	0.85	0.10	0.59	13.00
Monounsaturated fat intake (g/day)	Nutrients	54570.00	0.06	0.00	0.00	0.00	0.92	0.10	0.59	3.00
Monosaccharides intake (g/day)	Nutrients	54570.00	-0.02	0.00	0.00	0.66	0.09	0.10	0.59	49.00
Sodium intake (mg/day)	Nutrients	54568.00	0.02	0.00	0.00	0.00	0.81	0.10	0.59	48.00
Vitamin B3 intake (mg/day)	Nutrients	54570.00	0.03	0.00	0.00	0.00	0.53	0.10	0.59	26.00
Cambridge physical activity index	Physical activity	57199.00	-0.02	0.00	0.00	0.00	0.97	0.07	0.55	71.00
Polyunsaturated fat intake (g/day)	Nutrients	54570.00	0.02	0.00	0.00	0.00	0.93	0.10	0.59	40.00
Average portion size of potatoes/rice/pasta based on photographic illustration of four sizes (smallest to largest)	Food	54570.00	-0.04	0.00	0.00	0.00	0.95	0.07	0.55	73.00
Total protein intake (g/day)	Nutrients	54570.00	-0.02	0.00	0.00	0.00	0.37	-	-	-
Animal based protein intake (g/day)	Nutrients	54570.00	0.00	0.00	0.28	0.04	0.31	-	-	-
Plant based protein intake (g/day)	Nutrients	54570.00	-0.05	0.00	0.00	0.00	0.56	0.10	0.59	6.00
Vitamin A intake (mg/day)	Nutrients	54570.00	0.01	0.00	0.23	0.00	0.47	-	-	-
Sucrose intake (g/day)	Nutrients	54570.00	-0.02	0.00	0.00	0.00	0.58	0.10	0.59	43.00
Cohabitation: Live alone vs Only one adult (spouse, partner)	Social	27798.00	0.03	0.02	0.05	0.00	0.42	-	-	-
Cohabitation: Live alone vs Only children	Social	8998.00	-0.11	0.03	0.00	0.00	0.90	0.07	0.52	81.00
Cohabitation: Live alone vs Adult and children	Social	34522.00	-0.03	0.02	0.11	0.00	0.56	-	-	-
Cohabitation: Live alone vs Other/others	Social	6859.00	0.12	0.05	0.01	0.00	0.42	-	-	-
Selenium intake (ug/day)	Nutrients	54570.00	0.01	0.00	0.12	0.48	0.17	-	-	-
Self rate of overall health	General health	28267.00	-0.03	0.01	0.00	0.00	0.32	0.07	0.58	84.00

For how much of the time during the last four weeks has your physical health or your emotiol problems limited your ability to interact with others?	General health	28035.00	0.01	0.01	0.31	0.00	0.81	-	-	-
Get sick more often than other people	General health	28155.00	0.00	0.01	0.45	0.00	0.51	-	-	-
As healthy as anyone	General health	28072.00	-0.01	0.01	0.07	0.16	0.28	-	-	-
Worsen in health in the future	General health	28063.00	0.01	0.01	0.19	0.00	0.66	-	-	-
Excellent health	General health	28159.00	-0.02	0.01	0.00	0.00	0.97	0.07	0.58	82.00
Self rate of overall health compared to a year ago	General health	28032.00	-0.01	0.01	0.02	0.00	0.38	-	-	-
Physical limitation to participate in steneous activities: running, lifting heavy objects, taking part in physically demanding sports	General health	28215.00	0.02	0.01	0.00	0.00	0.74	0.07	0.59	83.00
Physical limitation to participate in moderately demanding activities: moving a table, vacuuming, walking in the forest or gardening	General health	28205.00	0.02	0.01	0.01	0.00	0.73	-	-	-
Physical limitation to participate in moderately demanding activities: lifting or carrying grocery bags	General health	28184.00	0.02	0.01	0.01	0.00	0.93	-	-	-
Physical limitation to participate in moderately demanding activities: walking up several stairs	General health	28196.00	0.01	0.01	0.03	0.00	0.89	-	-	-
Physical limitation to participate in moderately demanding activities: bending down or kneeling	General health	28164.00	0.00	0.01	0.74	0.00	0.85	-	-	-
Physical limitation to participate in moderately demanding activities: walking more than two km	General health	28205.00	0.01	0.01	0.29	0.00	0.39	-	-	-
Physical limitation that reduced the normal time spent at work or in other activities during the last four weeks	General health	28231.00	0.04	0.02	0.03	0.31	0.23	-	-	-
Physical limitation that made you do less than you wanted during the last four weeks	General health	28214.00	0.02	0.01	0.13	0.00	0.80	-	-	-
Physical limitation that made you not being able to perform certain work tasks or other activities during the last four weeks	General health	28103.00	0.02	0.02	0.36	0.60	0.11	-	-	-
Physical limitation that limited your ability to perform certain work tasks or other activities during the last four weeks	General health	28140.00	0.02	0.02	0.27	0.00	0.87	-	-	-
Emotiol problems that made you do less than you wanted during the last four weeks	General health	28230.00	0.00	0.02	0.78	0.63	0.10	-	-	-
Extent to what your physical and emotiol health disrupted your usual social life during the last four weeks	General health	28214.00	0.00	0.01	0.50	0.00	0.38	-	-	-
Pain during the last four weeks	General health	28177.00	0.01	0.01	0.04	0.00	0.36	-	-	-

How much has the pain during the last four weeks disturbed your normal work?	General health	28115.00	0.01	0.01	0.05	0.00	0.33	-	-	-
For how much of the time during the last four weeks have you felt really alert and strong?	General health	28127.00	-0.01	0.01	0.04	0.00	0.65	-	-	-
For how much of the time during the last four weeks have you felt very nervous?	General health	28173.00	0.01	0.01	0.20	0.04	0.31	-	-	-
For how much of the time during the last four weeks have you felt so depressed that nothing could cheer you up?	General health	28203.00	0.01	0.01	0.14	0.00	0.62	-	-	-
For how much of the time during the last four weeks have you felt calm and serene?	General health	28179.00	-0.01	0.01	0.08	0.00	0.59	-	-	-
For how much of the time during the last four weeks have you felt full of energy?	General health	28142.00	-0.01	0.01	0.01	0.00	0.61	-	-	-
For how much of the time during the last four weeks have you felt gloomy and sad?	General health	28150.00	0.01	0.01	0.26	0.68	0.08	-	-	-
For how much of the time during the last four weeks have you felt worn out?	General health	28107.00	0.02	0.01	0.01	0.00	0.74	-	-	-
For how much of the time during the last four weeks have you felt happy?	General health	28169.00	-0.01	0.01	0.14	0.00	0.98	-	-	-
For how much of the time during the last four weeks have you felt tired?	General health	28195.00	0.00	0.01	0.44	0.00	0.54	-	-	-
Long-term sickness	General health	58044.00	-0.01	0.01	0.39	0.16	0.28	-	-	-
Work shifts/weekends	Psychosocial	58150.00	-0.01	0.01	0.21	0.00	0.41	-	-	-
Risk of sleeping while sitting and reading	Sleep	21904.00	-0.02	0.01		0.00	0.45	-	-	-
Risk of sleeping while watching TV	Sleep	22137.00	0.00	0.01		0.00	0.43	-	-	-
Risk of sleeping while sitting ictive in a public place	Sleep	21993.00	-0.03	0.01		0.00	0.35	-	-	-
Risk of sleeping as a passenger in a car for one hour without break	Sleep	21908.00	-0.02	0.01		0.00	0.94	-	-	-
Risk of sleeping while lying down resting in the afternoon	Sleep	22065.00	-0.01	0.01		0.00	0.66	-	-	-
Risk of sleeping while sitting still after having lunch	Sleep	22076.00	-0.01	0.01		0.00	0.61	-	-	-
Snore during sleep	Sleep	20666.00	0.03	0.01		0.00	0.84	-	-	-
Breath-holds during sleep	Sleep	15446.00	0.02	0.01		0.00	0.77	-	-	-
Number of cigarretes smoked per day (in groups)	Tobacco use	49255.00	0.03	0.01	0.00	0.00	0.42	0.07	0.55	69.00
Years smoking	Tobacco use	52070.00	0.03	0.01	0.00	0.00	0.32	0.07	0.54	74.00
Smoking status: Smokers vs non-smokers	Tobacco use	38085.00	0.10	0.02	0.00	0.00	0.64	0.08	0.54	68.00
Smoking status: Former smokers vs non-smokers	Tobacco use	42720.00	0.04	0.01	0.00	0.00	0.37	-	-	-
Smoking status: Former occasiol smokers vs non-smokers	Tobacco use	35637.00	0.00	0.02	0.84	0.00	0.91	-	-	-
Number of snuff boxes per week	Tobacco use	57762.00	0.00	0.00	0.41	0.69	0.07	-	-	-
Snuff status: Snuff users vs non-snuff users	Tobacco use	50978.00	0.03	0.01	0.02	0.62	0.10	-	-	-

Snuff status: Former snuff users vs non-snuff users	Tobacco use	47600.00	0.07	0.02	0.00	0.00	0.67	0.07	0.55	70.00
Years using snuff	Tobacco use	56250.00	0.02	0.01	0.00	0.37	0.21	-	-	-
Participation in associations or voluntary organisations	Social	59347.00	-0.02	0.01	0.08	0.00	0.56	-	-	-
Participation in sports or physical exercise associations	Social	20771.00	0.01	0.01	0.60	0.00	0.84	-	-	-
Participation in study circles	Social	20771.00	-0.02	0.02	0.38	0.00	0.74	-	-	-
Participation in other association	Social	20771.00	-0.02	0.01	0.26	0.66	0.08	-	-	-
People to ask for help apart from the ones at home	Social	59507.00	0.02	0.01	0.14	0.00	0.84	-	-	-
Number of friends that can come to your home at any time and feel at home	Social	59481.00	0.03	0.00	0.00	0.00	0.40	0.07	0.55	76.00
Number of social contacts with the same interests as you	Social	59590.00	0.02	0.00	0.00	0.00	0.81	0.07	0.55	79.00
Would you say that the number of people that you meet in your everyday life is enough or would you like to meet more or fewer people?	Social	59656.00	-0.01	0.00	0.00	0.82	0.02	-	-	-
Close relationship with anyone	Social	59615.00	0.01	0.00	0.19	0.27	0.24	-	-	-
Frequency of engaging in clubs, associations or study circles	Social	37401.00	-0.01	0.01	0.03	0.00	0.71	-	-	-
Number of social interactions during a normal week	Social	59681.00	0.00	0.00	0.31	0.00	0.66	-	-	-
Support from others	Social	59704.00	0.01	0.00	0.19	0.64	0.10	-	-	-
Number of people with whom you can speak openly	Social	59633.00	0.02	0.00	0.00	0.00	0.67	-	-	-
Receive hugs to comfort and support you	Social	59226.00	0.01	0.01	0.42	0.00	0.76	-	-	-
Stigmasterol intake (mg/day)	Nutrients	54570.00	-0.01	0.00	0.01	0.00	0.50	-	-	-
Tiamin intake (mg/day)	Nutrients	54570.00	0.00	0.00	0.54	0.00	0.83	-	-	-
Vitamin E intake (mg/day)	Nutrients	54570.00	0.01	0.00	0.02	0.00	0.40	-	-	-
Trans fat intake (g/day)	Nutrients	54570.00	0.07	0.01	0.00	0.00	0.65	0.10	0.59	4.00
Sum of phytosterols intake (mg/day)	Nutrients	54570.00	0.01	0.00	0.11	0.00	0.54	-	-	-
Zinc intake (mg/day)	Nutrients	54570.00	-0.01	0.00	0.10	0.00	0.86	-	-	-

Supplementary Table 7. Linear mixed model association results for triglycerides

Description	Group	N	Effect estimate	S.E.	p-value	I^2	Q p-value	marginal R2	conditional R2	R2 rank
Alcohol intake (g/day)	Alcohol	49319.00	0.00	0.00	0.39	0.68	0.08	-	-	-
Permanent employment	Psychosocial	45477.00	-0.03	0.01	0.00	0.00	0.73	0.12	0.50	48.00
Self-employed	Psychosocial	45477.00	-0.06	0.01	0.00	0.00	0.75	0.12	0.50	45.00
Distance to work in kilometers (one way)	Physical activity	46421.00	0.01	0.00	0.08	0.00	0.85	-	-	-
Last time a colleague visited you at home	Psychosocial	52428.00	0.00	0.00	0.71	0.00	0.67	-	-	-
Job demands to work very fast	Psychosocial	53199.00	-0.02	0.00	0.00	0.00	0.65	0.11	0.50	64.00
Frequency of social contacts with colleagues during leisure time	Psychosocial	51651.00	0.00	0.00	0.21	0.00	0.56	-	-	-
High physical demand from job	Physical activity	53330.00	-0.01	0.00	0.01	0.11	0.29	-	-	-
Enough time for job assignments	Psychosocial	52951.00	0.02	0.00	0.00	0.00	0.39	0.11	0.51	61.00
Control over planning and execution of the workday	Psychosocial	53214.00	0.00	0.00	0.55	0.45	0.18	-	-	-
Ingenuity or creativity demand from job	Psychosocial	52934.00	-0.01	0.00	0.00	0.00	0.85	-	-	-
Frequent social contacts with colleagues during work	Psychosocial	52597.00	-0.02	0.00	0.00	0.00	0.40	0.11	0.50	66.00
Contradictory demands in job	Psychosocial	52842.00	-0.01	0.00	0.00	0.00	0.60	-	-	-
Possibility to leave your work for a while to speak with a colleague	Psychosocial	52644.00	0.00	0.00	0.63	0.00	0.59	-	-	-
Learn new things at job	Psychosocial	53082.00	-0.01	0.00	0.00	0.00	0.35	-	-	-
High mental demand from job	Psychosocial	52769.00	0.01	0.00	0.00	0.00	0.40	-	-	-
Repetitive job	Psychosocial	53113.00	0.00	0.00	0.54	0.70	0.07	-	-	-
Skill demand from job	Psychosocial	53042.00	-0.02	0.00	0.00	0.00	0.56	0.11	0.51	62.00
Possibility to speak with colleagues during breaks	Psychosocial	52985.00	-0.01	0.00	0.00	0.69	0.07	-	-	-
Control over own work assignment	Psychosocial	53200.00	0.01	0.00	0.02	0.80	0.03	-	-	-
Vitamin C intake (mg/day)	Nutrients	49319.00	-0.02	0.00	0.00	0.00	0.55	0.12	0.51	8.00
Vitamin B12 intake (ug/day)	Nutrients	49319.00	0.00	0.00	0.51	0.00	0.33	-	-	-
Vitamin B2 intake (ug/day)	Nutrients	49319.00	0.00	0.00	0.16	0.91	0.00	-	-	-
Vitamin B6 intake (mg/day)	Nutrients	49319.00	0.00	0.00	0.43	0.62	0.10	-	-	-
Informed of having high blood pressure	General health	54025.00	0.10	0.01	0.00	0.70	0.07	0.12	0.49	52.00
Beta-sitostanol intake (mg/day)	Nutrients	49319.00	0.01	0.00	0.10	0.70	0.07	-	-	-
Beta-sitosterol intake (mg/day)	Nutrients	49319.00	-0.01	0.00	0.12	0.00	0.50	-	-	-
Marital status: Single vs Married/partner	Social	49350.00	-0.06	0.01	0.00	0.91	0.00	-	-	-
Marital status: Single vs Divorced/separated	Social	8918.00	0.00	0.02	0.88	0.88	0.00	-	-	-
Marital status: Single vs Widow/widower	Social	5568.00	0.02	0.04	0.69	0.00	0.50	-	-	-
Campestanol intake (mg/day)	Nutrients	49319.00	0.01	0.00	0.12	0.80	0.03	-	-	-
Campesterol intake (mg/day)	Nutrients	49319.00	0.01	0.00	0.00	0.00	0.34	-	-	-
Parents or siblings have diabetes	General health	53046.00	0.04	0.01	0.00	0.09	0.29	0.11	0.49	67.00
Disaccharides intake (g/day)	Nutrients	49319.00	0.01	0.00	0.02	0.23	0.25	-	-	-

Vitamin D intake (ug/day)	Nutrients	49319.00	0.01	0.00	0.00	0.00	0.59	-	-	-
Total energy intake (kcal/day)	Nutrients	49319.00	-0.02	0.00	0.00	0.00	0.82	0.12	0.51	30.00
Formic acid intaje (g/day)	Nutrients	49319.00	0.01	0.00	0.01	0.61	0.11	-	-	-
Pentadecanoic acid intake (g/day)	Nutrients	49319.00	0.00	0.00	0.55	0.52	0.15	-	-	-
Palmitic acid intake (g/day)	Nutrients	49319.00	0.02	0.00	0.00	0.75	0.05	-	-	-
Heptadecanoic acid intake (g/day)	Nutrients	49319.00	0.00	0.00	0.55	0.52	0.15	-	-	-
Linoleic acid intake (g/day)	Nutrients	49319.00	0.01	0.00	0.02	0.00	0.44	-	-	-
Linolenic acid intake (g/day)	Nutrients	49319.00	0.01	0.00	0.00	0.59	0.12	-	-	-
Arachidonic acid (ARA) intake (g/day)	Nutrients	49317.00	0.00	0.00	0.25	0.00	0.91	-	-	-
Eicosapentaenoic acid (EPA) intake (g/day)	Nutrients	49318.00	-0.01	0.00	0.00	0.00	0.86	-	-	-
Docosahexaenoic acid (DHA) intake (g/day)	Nutrients	49319.00	-0.01	0.00	0.00	0.00	0.67	-	-	-
Fat intake (g/day)	Nutrients	49319.00	0.01	0.00	0.00	0.77	0.04	-	-	-
Fibre intake (g/day)	Nutrients	49319.00	-0.03	0.00	0.00	0.84	0.01	0.12	0.51	7.00
Folic acid intake (ug/day)	Nutrients	49319.00	-0.02	0.00	0.00	0.63	0.10	0.12	0.51	15.00
Phosphate intake (mg/day)	Nutrients	49319.00	0.00	0.00	0.17	0.92	0.00	-	-	-
Whole grain intake (g/day)	Food	49319.00	-0.01	0.00	0.00	0.50	0.16	-	-	-
Travel to work: Walk to work vs passive travel to work	Physical activity	35059.00	-0.01	0.01	0.42	0.15	0.28	-	-	-
Travel to work: Cycle to work vs passive travel to work	Physical activity	40495.00	-0.05	0.01	0.00	0.51	0.15	0.12	0.51	51.00
Travel to work: Irregular travel mode to work vs passive travel to work	Physical activity	34963.00	0.00	0.01	0.97	0.00	0.98	-	-	-
Time spent in a week in moderately steneous activities	Physical activity	20008.00	-0.04	0.01		0.47	0.17	-	-	-
Sedentary or standing work	Physical activity	52354.00	0.01	0.01	0.12	0.39	0.20	-	-	-
Light but partly physically active work	Physical activity	52354.00	0.01	0.01	0.23	0.00	0.48	-	-	-
Light and physically active work	Physical activity	52354.00	0.00	0.01	0.81	0.00	0.70	-	-	-
Sometimes physically straining work	Physical activity	52354.00	-0.01	0.01	0.08	0.00	0.66	-	-	-
Frequency of walking during leisure time	Physical activity	52082.00	-0.01	0.00	0.00	0.00	0.52	-	-	-
Frequency of cycling during leisure time	Physical activity	43908.00	-0.02	0.00	0.00	0.46	0.18	0.12	0.49	59.00
Frequency of dancing during leisure time	Physical activity	29683.00	0.00	0.00	0.42	0.00	0.97	-	-	-
Frequency of shoveling snow during leisure time	Physical activity	31822.00	-0.02	0.00	0.00	0.00	0.91	0.11	0.46	77.00
Frequency of gardening during leisure time	Physical activity	31648.00	-0.01	0.00	0.05	0.00	0.65	-	-	-
Frequency of hunting or fishing during leisure time	Physical activity	30977.00	0.01	0.00	0.01	0.00	0.94	-	-	-
Frequency of picking berries or mushrooms during leisure time	Physical activity	31486.00	-0.01	0.00	0.07	0.00	0.80	-	-	-
Changed everyday exercise during the last year	Physical activity	33339.00	-0.01	0.00	0.02	0.00	0.82	-	-	-
Everyday exercise satisfaction	Physical activity	33233.00	-0.02	0.00	0.00	0.15	0.28	0.11	0.48	76.00
Exercise during the last three months	Physical activity	53420.00	-0.04	0.00	0.00	0.74	0.05	0.12	0.49	57.00

If you exercise, change in exercise habits during the last year	Physical activity	29383.00	-0.01	0.00	0.00	0.00	0.61	-	-	-
Amount of exercise during the last 12 months	Physical activity	20671.00	-0.06	0.01		0.00	0.75	-	-	-
Bregott on bread	Food	49319.00	0.00	0.00	0.14	0.91	0.00	-	-	-
Whole grain crisp bread	Food	49319.00	0.01	0.00	0.01	0.00	0.99	-	-	-
Whole grain soft bread	Food	49319.00	0.00	0.00	0.62	0.71	0.06	-	-	-
White (soft) bread, thin crisp bread	Food	49319.00	0.01	0.00	0.01	0.00	0.55	-	-	-
Coffee rolls/buns, rusk	Food	49319.00	-0.01	0.00	0.08	0.00	0.49	-	-	-
Cheese 28%	Food	49319.00	0.00	0.00	0.88	0.00	0.89	-	-	-
Cheese 10-17%	Food	49319.00	0.00	0.00	0.15	0.00	0.38	-	-	-
Soft cheese	Food	8421.00	-0.01	0.01		0.70	0.07	-	-	-
Soft whey cheese	Food	8421.00	-0.01	0.01		0.00	0.81	-	-	-
Sausage, liver pate on bread	Food	49319.00	0.01	0.00	0.00	0.00	0.79	0.12	0.51	27.00
Meat on bread	Food	49319.00	0.00	0.00	0.46	0.00	0.39	-	-	-
Butter on bread	Food	49319.00	0.01	0.00	0.09	0.00	0.71	-	-	-
Oatflake, whole wheat, rye or barley porridge	Food	49319.00	-0.02	0.00	0.00	0.00	0.62	0.12	0.51	21.00
Rosehip, sweet syrup soup	Food	49319.00	0.00	0.00	0.32	0.00	0.32	-	-	-
Sour milk, yoghurt (3% fat)	Food	49319.00	-0.02	0.00	0.00	0.00	0.54	0.12	0.51	14.00
Sour milk, yoghurt (low fat)	Food	49319.00	-0.01	0.00	0.01	0.66	0.09	-	-	-
Fiber cereals	Food	49319.00	-0.02	0.00	0.00	0.67	0.08	0.12	0.51	3.00
Corn flakes	Food	49319.00	0.00	0.00	0.16	0.65	0.09	-	-	-
Berries (fresh or frozen)	Food	49319.00	-0.01	0.00	0.01	0.02	0.31	-	-	-
Apple, pear, peach, orange, mandarin and grapefruit	Food	49319.00	-0.02	0.00	0.00	0.00	0.49	0.12	0.51	13.00
Ba	Food	49319.00	-0.01	0.00	0.00	0.00	0.83	-	-	-
Root vegetables and carrot	Food	49319.00	-0.02	0.00	0.00	0.58	0.12	0.12	0.51	22.00
Low fat margarine on bread	Food	49319.00	0.00	0.00	0.23	0.00	0.69	-	-	-
Tomato and cucumber	Food	49319.00	-0.02	0.00	0.00	0.00	0.68	0.12	0.51	19.00
White cabbage, lettuce, lettuce cabbage, spich, borecole and broccoli	Food	49319.00	-0.02	0.00	0.00	0.00	0.34	0.12	0.51	26.00
Mixed frozen vegetables	Food	8421.00	0.00	0.01		0.00	0.77	-	-	-
Boiled or baked potato	Food	49319.00	0.01	0.00	0.11	0.00	0.87	-	-	-
Fried potatoes and pommes frites	Food	49319.00	0.02	0.00	0.00	0.00	0.51	0.12	0.51	6.00
Mashed potato	Food	8421.00	0.02	0.01		0.68	0.08	-	-	-
Potato salad	Food	8421.00	0.01	0.01		0.27	0.24	-	-	-
Rice	Food	49319.00	0.01	0.00	0.07	0.89	0.00	-	-	-
Pasta	Food	49319.00	0.01	0.00	0.01	0.74	0.05	-	-	-
Brown beans and pea soup	Food	49319.00	-0.01	0.00	0.08	0.00	0.96	-	-	-
Margarine on bread	Food	49319.00	0.00	0.00	0.39	0.00	0.34	-	-	-
Blota (broth + bread)	Food	8421.00	0.02	0.01		0.00	0.94	-	-	-
Pancake, waffle and Swedish dumpling	Food	49319.00	0.01	0.00	0.10	0.00	0.58	-	-	-
Pizza	Food	49319.00	0.02	0.00	0.00	0.00	0.88	0.12	0.51	11.00
Minced meat dishes	Food	49319.00	0.00	0.00	0.33	0.00	0.74	-	-	-
Meat stew	Food	49319.00	0.01	0.00	0.05	0.00	0.35	-	-	-
Steak, chop, etc.	Food	49319.00	0.00	0.00	0.24	0.68	0.08	-	-	-
Bacon	Food	49319.00	0.01	0.00	0.03	0.00	0.61	-	-	-
Sausage as main dish	Food	49319.00	0.01	0.00	0.00	0.00	0.34	-	-	-
Hamburger	Food	49319.00	0.01	0.00	0.00	0.77	0.04	-	-	-
White meat (poultry)	Food	49319.00	0.00	0.00	0.15	0.00	0.32	-	-	-
Butter for cooking	Food	49319.00	0.00	0.00	0.47	0.39	0.20	-	-	-
Blood based food	Food	8421.00	-0.01	0.01		0.21	0.26	-	-	-
Liver and kidney	Food	8421.00	-0.02	0.01		0.84	0.01	-	-	-

Lean fish (e.g. perch, bass, cod)	Food	49319.00	-0.01	0.00	0.11	0.76	0.04	-	-	-
Fatty fish (e.g. herring, whitefish, salmon)	Food	49319.00	-0.01	0.00	0.00	0.00	0.90	0.12	0.51	28.00
Shellfish (e.g. shrimps, scallops)	Food	8421.00	-0.01	0.01		0.48	0.17	-	-	-
Salty fish	Food	49319.00	0.00	0.00	0.46	0.00	0.83	-	-	-
Smoked fish/meat	Food	49319.00	0.00	0.00	0.69	0.00	0.56	-	-	-
Ice cream	Food	49319.00	-0.01	0.00	0.00	0.69	0.07	-	-	-
Sweets	Food	49319.00	0.01	0.00	0.03	0.00	0.84	-	-	-
Sugar, honey, marmelade and jam	Food	49319.00	0.01	0.00	0.00	0.00	0.33	-	-	-
Margarine for cooking	Food	49319.00	0.02	0.00	0.00	0.00	0.62	0.12	0.51	12.00
Cookies and pastry	Food	49319.00	-0.01	0.00	0.00	0.61	0.11	-	-	-
Chips, popcorn and salted nuts	Food	49319.00	0.01	0.00	0.02	0.00	0.61	-	-	-
Low fat milk (0.5%)	Beverage	49319.00	0.01	0.00	0.04	0.00	0.83	-	-	-
Milk, sour milk (1.5%)	Beverage	49319.00	0.01	0.00	0.00	0.59	0.12	-	-	-
Milk, sour milk (3%)	Beverage	49319.00	0.00	0.00	0.76	0.00	0.79	-	-	-
Sodas, soft drinks and juice	Beverage	49319.00	0.02	0.00	0.00	0.93	0.00	-	-	-
Brewed (filtered) coffee	Beverage	49319.00	-0.03	0.00	0.00	0.00	0.77	0.12	0.51	5.00
Boiled coffee	Beverage	49319.00	0.02	0.00	0.00	0.00	0.78	0.12	0.51	4.00
Tea	Beverage	49319.00	-0.01	0.00	0.00	0.70	0.07	-	-	-
Light beer	Alcohol	49319.00	-0.01	0.00	0.00	0.17	0.27	-	-	-
Oil for cooking	Food	49319.00	0.00	0.00	0.45	0.00	0.50	-	-	-
Medium beer	Alcohol	49319.00	0.00	0.00	0.71	0.58	0.12	-	-	-
Strong beer	Alcohol	49319.00	0.02	0.00	0.00	0.73	0.05	0.12	0.51	9.00
Wine	Alcohol	49319.00	-0.01	0.00	0.09	0.00	0.35	-	-	-
Liquor and spirits	Alcohol	49319.00	0.01	0.00	0.00	0.43	0.19	-	-	-
Salad dressing with oil	Food	49319.00	-0.01	0.00	0.04	0.00	0.67	-	-	-
Cream, creme fraiche, sour cream	Food	49319.00	0.00	0.00	0.56	0.00	0.53	-	-	-
Average portion size of vegetables based on photographic illustration of four sizes (smallest to largest)	Food	49319.00	-0.03	0.00	0.00	0.74	0.05	0.12	0.50	49.00
Overall state of health during the last year	General health	54012.00	-0.04	0.00	0.00	0.00	0.64	0.12	0.49	55.00
Overall state of health compared to others your age	General health	27504.00	-0.04	0.00	0.00	0.00	0.55	0.11	0.48	71.00
Parents or siblings had a cerebral hemorrhage/thrombosis or cardiac infarction before the age of 60	General health	53002.00	0.04	0.01	0.00	0.72	0.06	-	-	-
Teetotaler	Alcohol	33310.00	-0.02	0.01	0.07	0.00	0.54	-	-	-
Feel the need to reduce alcohol consumption	Alcohol	49717.00	0.08	0.01	0.00	0.00	0.63	0.12	0.48	58.00
Feel uneasy or guilty because of your way of drinking	Alcohol	30090.00	0.03	0.01	0.04	0.00	0.44	-	-	-
Frequency of alcohol consumption	Alcohol	20776.00	0.01	0.01		0.12	0.29	-	-	-
Amount of alcohol drunk in a day	Alcohol	19801.00	0.05	0.01		0.22	0.26	-	-	-
Frequency of drinking six or more glasses at the same occasion	Alcohol	20184.00	0.05	0.01		0.00	0.72	-	-	-
Times during last year that you felt guilty because of your drinking	Alcohol	20153.00	0.01	0.01		0.00	0.56	-	-	-
Iron intake (mg/day)	Nutrients	49319.00	-0.01	0.00	0.08	0.62	0.10	-	-	-
Iodine intake (ug/day)	Nutrients	49319.00	0.00	0.00	0.19	0.90	0.00	-	-	-
Calcium intake (mg/day)	Nutrients	49319.00	-0.01	0.00	0.01	0.81	0.02	-	-	-
Potassium intake (mg/day)	Nutrients	49319.00	-0.01	0.00	0.00	0.79	0.03	-	-	-

Beta-carotene intake (mg/day)	Nutrients	49319.00	-0.02	0.00	0.00	0.56	0.13	0.12	0.51	20.00
Cholesterol intake (g/day)	Nutrients	49319.00	0.00	0.00	0.41	0.21	0.26	-	-	-
Carbohydrates intake (g/day)	Nutrients	49319.00	-0.01	0.00	0.00	0.61	0.11	-	-	-
Average portion size of meat/fish based on photographic illustration of four sizes (smallest to largest)	Food	49319.00	0.00	0.00	0.59	0.00	0.94	-	-	-
Breakfast habits: Only coffee/tea for breakfast vs not breakfast at all	Food	3488.00	0.02	0.04	0.65	0.00	0.63	-	-	-
Breakfast habits: Coffee/tea and wheat buns or rusk for breakfast vs not breakfast at all	Food	2730.00	-0.01	0.04	0.79	0.00	0.44	-	-	-
Breakfast habits: Porridge w/o sandwich for breakfast vs not breakfast at all	Food	7449.00	-0.18	0.02	0.00	0.38	0.20	0.10	0.47	96.00
Breakfast habits: Gruel w/o sandwich for breakfast vs not breakfast at all	Food	2531.00	-0.03	0.05	0.60	0.00	0.63	-	-	-
Eat breakfast from 2000	Food	31473.00	-0.14	0.01	0.00	0.00	0.45	0.11	0.46	68.00
Enterodiol intake (ug/day)	Nutrients	49319.00	0.01	0.00	0.12	0.00	0.99	-	-	-
Enterolactone intake (ug/day)	Nutrients	49319.00	-0.01	0.00	0.10	0.00	0.68	-	-	-
Equol intake (ug/day)	Nutrients	49319.00	0.00	0.00	0.95	0.25	0.25	-	-	-
Lariciresinol intake (ug/day)	Nutrients	49319.00	-0.02	0.00	0.00	0.75	0.05	0.12	0.51	24.00
Matairesinol intake (ug/day)	Nutrients	49319.00	-0.01	0.00	0.03	0.65	0.09	-	-	-
Medioresinol intake (ug/day)	Nutrients	49319.00	0.01	0.00	0.09	0.47	0.17	-	-	-
Pinoresinol intake (ug/day)	Nutrients	49319.00	-0.01	0.00	0.00	0.77	0.04	-	-	-
Secoisolariciresinol intake (ug/day)	Nutrients	49319.00	-0.02	0.00	0.00	0.00	0.44	0.12	0.51	16.00
Sum of all ligns intake (ug/day)	Nutrients	49319.00	0.00	0.00	0.24	0.71	0.06	-	-	-
Syringaresinol intake (ug/day)	Nutrients	49319.00	0.00	0.00	0.74	0.68	0.08	-	-	-
Sum of Lariciresinol, Matairesinol, Pinoresinol, Secoisolariciresinol intake (ug/day)	Nutrients	49319.00	-0.02	0.00	0.00	0.75	0.04	0.12	0.51	23.00
Satisfaction with home and family situation	Psychosocial	44731.00	-0.01	0.00	0.00	0.00	0.71	0.12	0.50	42.00
Appetite status	Psychosocial	44724.00	-0.03	0.00	0.00	0.68	0.08	0.12	0.50	36.00
Mood status	Psychosocial	44692.00	-0.02	0.00	0.00	0.00	0.86	0.12	0.50	39.00
Energy status	Psychosocial	44665.00	-0.04	0.00	0.00	0.00	0.94	0.12	0.50	32.00
Patience status	Psychosocial	44704.00	-0.02	0.00	0.00	0.00	0.49	0.12	0.50	40.00
Confidence status	Psychosocial	44678.00	-0.01	0.00	0.00	0.00	0.90	0.12	0.51	44.00
Sleep status	Sleep	44766.00	-0.04	0.00	0.00	0.36	0.21	0.12	0.50	34.00
Do you feel important and appreciated outside your home?	Psychosocial	44756.00	-0.01	0.00	0.00	0.00	0.41	-	-	-
Do you feel important and appreciated in your home?	Psychosocial	44431.00	-0.02	0.00	0.00	0.00	0.94	0.12	0.50	41.00
Satisfaction with accomodation	Psychosocial	44771.00	-0.01	0.00	0.00	0.02	0.31	-	-	-
Satisfaction with work situation	Psychosocial	44158.00	-0.02	0.00	0.00	0.00	0.43	0.12	0.51	33.00
Satisfaction with economy	Psychosocial	44719.00	-0.02	0.00	0.00	0.15	0.28	0.12	0.51	38.00

Satisfaction with leisure time	Psychosocial	44665.00	-0.02	0.00	0.00	0.39	0.20	-	-	-
Hearing status	General health	44753.00	-0.01	0.00	0.08	0.00	0.43	-	-	-
Vision status	General health	44706.00	-0.02	0.00	0.00	0.00	0.78	0.12	0.50	43.00
Memory status	Psychosocial	44636.00	-0.01	0.00	0.01	0.42	0.19	-	-	-
Fitness status	Physical activity	44693.00	-0.07	0.00	0.00	0.77	0.04	0.13	0.51	2.00
Magnesium intake (mg/day)	Nutrients	49319.00	-0.02	0.00	0.00	0.88	0.00	-	-	-
Saturated fat intake (g/day)	Nutrients	49319.00	0.01	0.00	0.01	0.66	0.09	-	-	-
Monounsaturated fat intake (g/day)	Nutrients	49319.00	0.02	0.00	0.00	0.83	0.02	0.12	0.51	10.00
Monosaccharides intake (g/day)	Nutrients	49319.00	-0.01	0.00	0.00	0.00	0.67	0.12	0.51	29.00
Sodium intake (mg/day)	Nutrients	49317.00	0.02	0.00	0.00	0.79	0.03	-	-	-
Vitamin B3 intake (mg/day)	Nutrients	49319.00	0.00	0.00	0.82	0.80	0.02	-	-	-
Cambridge physical activity index	Physical activity	51679.00	-0.04	0.00	0.00	0.86	0.01	0.12	0.49	53.00
Polyunsaturated fat intake (g/day)	Nutrients	49319.00	0.01	0.00	0.00	0.00	0.46	-	-	-
Average portion size of potatoes/rice/pasta based on photographic illustration of four sizes (smallest to largest)	Food	49319.00	0.00	0.00	0.26	0.34	0.22	-	-	-
Total protein intake (g/day)	Nutrients	49319.00	-0.01	0.00	0.04	0.86	0.01	-	-	-
Animal based protein intake (g/day)	Nutrients	49319.00	0.00	0.00	0.64	0.34	0.22	-	-	-
Plant based protein intake (g/day)	Nutrients	49319.00	-0.02	0.00	0.00	0.89	0.00	0.12	0.51	18.00
Vitamin A intake (mg/day)	Nutrients	49319.00	0.02	0.00	0.00	0.00	0.89	0.12	0.51	25.00
Sucrose intake (g/day)	Nutrients	49319.00	0.00	0.00	0.90	0.89	0.00	-	-	-
Cohabitation: Live alone vs Only one adult (spouse, partner)	Social	25579.00	-0.05	0.01	0.00	0.67	0.08	-	-	-
Cohabitation: Live alone vs Only children	Social	8080.00	-0.06	0.02	0.01	0.00	0.68	-	-	-
Cohabitation: Live alone vs Adult and children	Social	30769.00	-0.09	0.01	0.00	0.52	0.15	0.13	0.50	1.00
Cohabitation: Live alone vs Other/others	Social	6174.00	-0.12	0.04	0.00	0.00	0.84	-	-	-
Selenium intake (ug/day)	Nutrients	49319.00	-0.01	0.00	0.00	0.87	0.01	-	-	-
Self rate of overall health	General health	26526.00	-0.08	0.00	0.00	0.53	0.15	0.12	0.55	60.00
For how much of the time during the last four weeks has your physical health or your emotiol problems limited your ability to interact with others?	General health	26301.00	0.03	0.00	0.00	0.82	0.02	0.11	0.55	83.00
Get sick more often than other people	General health	26413.00	0.03	0.00	0.00	0.00	0.60	0.11	0.56	88.00
As healthy as anyone	General health	26334.00	-0.04	0.00	0.00	0.00	0.76	0.11	0.56	80.00
Worsen in health in the future	General health	26327.00	0.03	0.00	0.00	0.63	0.10	0.11	0.56	89.00
Excellent health	General health	26413.00	-0.06	0.00	0.00	0.00	0.54	0.11	0.56	69.00
Self rate of overall health compared to a year ago	General health	26309.00	-0.02	0.00	0.00	0.32	0.23	-	-	-
Physical limitation to participate in steneous activities: running, lifting heavy objects, taking part in physically demanding sports	General health	26474.00	0.05	0.00	0.00	0.60	0.11	0.11	0.55	72.00

Physical limitation to participate in moderately demanding activities: moving a table, vacuuming, walking in the forest or gardening	General health	26461.00	0.04	0.00	0.00	0.00	0.65	0.11	0.55	81.00
Physical limitation to participate in moderately demanding activities: lifting or carrying grocery bags	General health	26452.00	0.05	0.00	0.00	0.24	0.25	0.11	0.56	75.00
Physical limitation to participate in moderately demanding activities: walking up several stairs	General health	26455.00	0.05	0.00	0.00	0.00	0.78	0.11	0.55	74.00
Physical limitation to participate in moderately demanding activities: bending down or kneeling	General health	26422.00	0.03	0.00	0.00	0.00	0.47	0.11	0.55	93.00
Physical limitation to participate in moderately demanding activities: walking more than two km	General health	26464.00	0.04	0.00	0.00	0.00	0.68	0.11	0.55	90.00
Physical limitation that reduced the normal time spent at work or in other activities during the last four weeks	General health	26488.00	0.13	0.01	0.00	0.00	0.47	0.11	0.55	82.00
Physical limitation that made you do less than you wanted during the last four weeks	General health	26468.00	0.08	0.01	0.00	0.19	0.27	0.11	0.55	86.00
Physical limitation that made you not being able to perform certain work tasks or other activities during the last four weeks	General health	26363.00	0.10	0.01	0.00	0.00	0.59	0.11	0.55	91.00
Physical limitation that limited your ability to perform certain work tasks or other activities during the last four weeks	General health	26397.00	0.10	0.01	0.00	0.00	0.70	0.11	0.56	92.00
Emotiol problems that made you do less than you wanted during the last four weeks	General health	26485.00	0.06	0.01	0.00	0.51	0.15	-	-	-
Extent to what your physical and emotiol health disrupted your usual social life during the last four weeks	General health	26473.00	0.03	0.00	0.00	0.63	0.10	0.11	0.56	95.00
Pain during the last four weeks	General health	26436.00	0.04	0.00	0.00	0.50	0.16	0.11	0.55	84.00
How much has the pain during the last four weeks disturbed your normal work?	General health	26376.00	0.04	0.00	0.00	0.00	0.44	0.11	0.55	85.00
For how much of the time during the last four weeks have you felt really alert and strong?	General health	26389.00	-0.05	0.00	0.00	0.00	0.36	0.11	0.56	70.00

For how much of the time during the last four weeks have you felt very nervous?	General health	26430.00	0.01	0.00	0.00	0.00	0.97	-	-	-
For how much of the time during the last four weeks have you felt so depressed that nothing could cheer you up?	General health	26458.00	0.01	0.00	0.01	0.00	0.41	-	-	-
For how much of the time during the last four weeks have you felt calm and serene?	General health	26440.00	-0.03	0.00	0.00	0.71	0.06	0.11	0.56	94.00
For how much of the time during the last four weeks have you felt full of energy?	General health	26403.00	-0.05	0.00	0.00	0.18	0.27	0.11	0.55	73.00
For how much of the time during the last four weeks have you felt gloomy and sad?	General health	26410.00	0.01	0.00	0.02	0.00	0.52	-	-	-
For how much of the time during the last four weeks have you felt worn out?	General health	26366.00	0.03	0.00	0.00	0.78	0.03	0.11	0.56	79.00
For how much of the time during the last four weeks have you felt happy?	General health	26431.00	-0.03	0.00	0.00	0.00	0.54	0.11	0.58	87.00
For how much of the time during the last four weeks have you felt tired?	General health	26455.00	0.04	0.00	0.00	0.36	0.21	0.11	0.56	78.00
Long-term sickness	General health	52534.00	0.09	0.01	0.00	0.00	0.50	0.12	0.50	56.00
Work shifts/weekends	Psychosocial	52590.00	0.01	0.01	0.19	0.00	0.35	-	-	-
Risk of sleeping while sitting and reading	Sleep	20291.00	-0.01	0.01		0.00	0.37	-	-	-
Risk of sleeping while watching TV	Sleep	20515.00	-0.01	0.01		0.64	0.10	-	-	-
Risk of sleeping while sitting ictive in a public place	Sleep	20377.00	-0.01	0.01		0.00	0.37	-	-	-
Risk of sleeping as a passenger in a car for one hour without break	Sleep	20301.00	-0.01	0.01		0.00	0.48	-	-	-
Risk of sleeping while lying down resting in the afternoon	Sleep	20446.00	0.01	0.01		0.00	0.84	-	-	-
Risk of sleeping while sitting still after having lunch	Sleep	20457.00	0.00	0.01		0.00	0.73	-	-	-
Snore during sleep	Sleep	19173.00	0.03	0.01		0.43	0.19	-	-	-
Breath-holds during sleep	Sleep	14261.00	0.03	0.01		0.50	0.16	-	-	-
Number of cigarretes smoked per day (in groups)	Tobacco use	44454.00	0.06	0.00	0.00	0.52	0.15	0.12	0.49	35.00
Years smoking	Tobacco use	47115.00	0.07	0.00	0.00	0.00	0.45	0.12	0.50	37.00
Smoking status: Smokers vs non-smokers	Tobacco use	34348.00	0.20	0.01	0.00	0.00	0.92	0.12	0.50	31.00
Smoking status: Former smokers vs non-smokers	Tobacco use	38547.00	0.06	0.01	0.00	0.00	0.34	0.12	0.49	47.00
Smoking status: Former occasiol smokers vs non-smokers	Tobacco use	31997.00	0.02	0.01	0.10	0.30	0.23	-	-	-
Number of snuff boxes per week	Tobacco use	52255.00	0.02	0.00	0.00	0.45	0.18	0.12	0.50	54.00
Snuff status: Snuff users vs non-snuff users	Tobacco use	46107.00	0.07	0.01	0.00	0.00	0.50	0.12	0.51	46.00
Snuff status: Former snuff users vs non-snuff users	Tobacco use	43135.00	0.03	0.01	0.00	0.61	0.11	-	-	-

Years using snuff	Tobacco use	50881.00	0.02	0.00	0.00	0.00	0.77	0.12	0.51	50.00
Participation in associations or voluntary organisations	Social	53680.00	-0.04	0.01	0.00	0.13	0.28	0.11	0.49	65.00
Participation in sports or physical exercise associations	Social	19597.00	-0.02	0.01	0.02	0.44	0.18	-	-	-
Participation in study circles	Social	19597.00	-0.01	0.01	0.39	0.00	0.99	-	-	-
Participation in other association	Social	19597.00	0.01	0.01	0.50	0.00	0.97	-	-	-
People to ask for help apart from the ones at home	Social	53833.00	0.00	0.01	0.85	0.00	0.71	-	-	-
Number of friends that can come to your home at any time and feel at home	Social	53808.00	0.00	0.00	0.29	0.00	0.52	-	-	-
Number of social contacts with the same interests as you	Social	53911.00	-0.01	0.00	0.00	0.00	0.67	-	-	-
Would you say that the number of people that you meet in your everyday life is enough or would you like to meet more or fewer people?	Social	53974.00	0.01	0.00	0.05	0.00	0.42	-	-	-
Close relationship with anyone	Social	53935.00	0.00	0.00	0.20	0.82	0.02	-	-	-
Frequency of engaging in clubs, associations or study circles	Social	33592.00	0.00	0.00	0.42	0.00	0.49	-	-	-
Number of social interactions during a normal week	Social	53992.00	-0.01	0.00	0.00	0.77	0.04	-	-	-
Support from others	Social	54010.00	-0.01	0.00	0.06	0.00	0.32	-	-	-
Number of people with whom you can speak openly	Social	53947.00	-0.01	0.00	0.05	0.00	0.64	-	-	-
Receive hugs to comfort and support you	Social	53573.00	-0.03	0.01	0.00	0.00	0.85	0.11	0.49	63.00
Stigmasterol intake (mg/day)	Nutrients	49319.00	-0.01	0.00	0.01	0.33	0.22	-	-	-
Tiamin intake (mg/day)	Nutrients	49319.00	-0.01	0.00	0.00	0.87	0.01	-	-	-
Vitamin E intake (mg/day)	Nutrients	49319.00	0.00	0.00	0.79	0.00	0.98	-	-	-
Trans fat intake (g/day)	Nutrients	49319.00	0.02	0.01	0.00	0.00	0.35	0.12	0.51	17.00
Sum of phytosterols intake (mg/day)	Nutrients	49319.00	0.00	0.00	1.00	0.00	0.67	-	-	-
Zinc intake (mg/day)	Nutrients	49319.00	0.00	0.00	0.27	0.88	0.00	-	-	-

Supplementary Table 8. Linear mixed model association results for HDL cholesterol

Description	Group	N	Effect estimate	S.E.	p-value	I^2	Q p-value	marginal R2	conditional R2	R2 rank
Alcohol intake (g/day)	Alcohol	23462.00	0.04	0.00	0.00	0.00	0.86	0.17	0.36	4.00
Permanent employment	Psychosocial	22901.00	0.01	0.01	0.07	0.68	0.08	-	-	-
Self-employed	Psychosocial	22901.00	0.01	0.01	0.25	0.00	0.82	-	-	-
Distance to work in kilometers (one way)	Physical activity	21957.00	0.00	0.00	0.39	0.25	0.25	-	-	-
Last time a colleague visited you at home	Psychosocial	25263.00	0.00	0.00	0.09	0.00	0.61	-	-	-
Job demands to work very fast	Psychosocial	25591.00	0.01	0.00	0.00	0.00	0.74	-	-	-
Frequency of social contacts with colleagues during leisure time	Psychosocial	24816.00	0.01	0.00	0.03	0.00	0.63	-	-	-
High physical demand from job	Physical activity	25660.00	0.01	0.00	0.02	0.00	0.81	-	-	-
Enough time for job assignments	Psychosocial	25464.00	0.00	0.00	0.31	0.00	0.94	-	-	-
Control over planning and execution of the workday	Psychosocial	25594.00	0.00	0.00	0.30	0.00	0.80	-	-	-
Ingenuity or creativity demand from job	Psychosocial	25448.00	0.01	0.00	0.07	0.91	0.00	-	-	-
Frequent social contacts with colleagues during work	Psychosocial	25320.00	0.01	0.00	0.00	0.33	0.22	-	-	-
Contradictory demands in job	Psychosocial	25434.00	0.00	0.00	0.46	0.53	0.15	-	-	-
Possibility to leave your work for a while to speak with a colleague	Psychosocial	25335.00	0.00	0.00	0.60	0.00	0.49	-	-	-
Learn new things at job	Psychosocial	25514.00	0.01	0.00	0.02	0.22	0.26	-	-	-
High mental demand from job	Psychosocial	25371.00	0.00	0.00	0.65	0.00	0.67	-	-	-
Repetitive job	Psychosocial	25537.00	0.00	0.00	0.97	0.00	0.52	-	-	-
Skill demand from job	Psychosocial	25505.00	0.01	0.00	0.00	0.27	0.24	-	-	-
Possibility to speak with colleagues during breaks	Psychosocial	25499.00	0.00	0.00	0.15	0.18	0.27	-	-	-
Control over own work assignment	Psychosocial	25582.00	-0.01	0.00	0.05	0.00	0.51	-	-	-
Vitamin C intake (mg/day)	Nutrients	23462.00	-0.01	0.00	0.01	0.00	0.62	-	-	-
Vitamin B12 intake (ug/day)	Nutrients	23462.00	0.01	0.00	0.00	0.00	0.89	-	-	-
Vitamin B2 intake (ug/day)	Nutrients	23462.00	0.00	0.00	0.13	0.00	0.80	-	-	-
Vitamin B6 intake (mg/day)	Nutrients	23462.00	0.00	0.00	0.59	0.00	0.68	-	-	-
Informed of having high blood pressure	General health	25984.00	-0.04	0.01	0.00	0.00	0.80	0.16	0.36	41.00
Beta-sitostanol intake (mg/day)	Nutrients	23462.00	0.00	0.00	0.72	0.00	0.36	-	-	-
Beta-sitosterol intake (mg/day)	Nutrients	23462.00	0.00	0.00	0.47	0.01	0.32	-	-	-
Marital status: Single vs Married/partner	Social	23590.00	-0.02	0.01	0.09	0.00	0.73	-	-	-
Marital status: Single vs Divorced/separated	Social	4596.00	0.00	0.01	0.78	0.00	0.53	-	-	-
Marital status: Single vs Widow/widower	Social	2946.00	0.03	0.03	0.24	0.68	0.08	-	-	-
Campestanol intake (mg/day)	Nutrients	23462.00	0.00	0.00	0.64	0.00	0.37	-	-	-
Campesterol intake (mg/day)	Nutrients	23462.00	-0.01	0.00	0.05	0.00	0.79	-	-	-
Parents or siblings have diabetes	General health	25426.00	-0.03	0.01	0.00	0.00	0.96	0.16	0.36	42.00
Disaccharides intake (g/day)	Nutrients	23462.00	-0.02	0.00	0.00	0.12	0.29	0.17	0.36	6.00
Vitamin D intake (ug/day)	Nutrients	23462.00	0.00	0.00	0.40	0.00	0.88	-	-	-
Total energy intake (kcal/day)	Nutrients	23462.00	-0.01	0.00	0.00	0.20	0.26	-	-	-
Formic acid intaje (g/day)	Nutrients	23462.00	0.01	0.00	0.00	0.00	0.73	0.17	0.36	21.00
Pentadecanoic acid intake (g/day)	Nutrients	23462.00	0.01	0.00	0.00	0.00	0.48	-	-	-
Palmitic acid intake (g/day)	Nutrients	23462.00	0.02	0.00	0.00	0.00	0.87	0.17	0.36	18.00
Heptadecanoic acid intake (g/day)	Nutrients	23462.00	0.01	0.00	0.00	0.00	0.48	-	-	-

Linoleic acid intake (g/day)	Nutrients	23462.00	0.00	0.00	0.35	0.00	0.74	-	-	-
Linolenic acid intake (g/day)	Nutrients	23462.00	0.00	0.00	0.27	0.00	0.87	-	-	-
Arachidonic acid (ARA) intake (g/day)	Nutrients	23461.00	0.01	0.00	0.00	0.00	0.41	0.17	0.36	26.00
Eicosapentaenoic acid (EPA) intake (g/day)	Nutrients	23461.00	0.01	0.00	0.00	0.00	0.40	0.17	0.36	24.00
Docosahexaenoic acid (DHA) intake (g/day)	Nutrients	23462.00	0.01	0.00	0.00	0.00	0.41	0.17	0.36	25.00
Fat intake (g/day)	Nutrients	23462.00	0.01	0.00	0.00	0.00	0.57	0.17	0.36	15.00
Fibre intake (g/day)	Nutrients	23462.00	0.00	0.00	0.51	0.00	0.45	-	-	-
Folic acid intake (ug/day)	Nutrients	23462.00	0.01	0.00	0.00	0.00	0.34	-	-	-
Phosphate intake (mg/day)	Nutrients	23462.00	0.01	0.00	0.03	0.00	0.47	-	-	-
Whole grain intake (g/day)	Food	23462.00	0.00	0.00	0.25	0.30	0.23	-	-	-
Travel to work: Walk to work vs passive travel to work	Physical activity	17211.00	0.03	0.01	0.00	0.00	0.88	-	-	-
Travel to work: Cycle to work vs passive travel to work	Physical activity	19093.00	0.04	0.01	0.00	0.00	0.50	0.16	0.34	35.00
Travel to work: Irregular travel mode to work vs passive travel to work	Physical activity	16920.00	0.01	0.01	0.33	0.22	0.26	-	-	-
Time spent in a week in moderately steneous activities	Physical activity	16406.00	0.02	0.00		0.00	0.45	-	-	-
Sedentary or standing work	Physical activity	25005.00	-0.02	0.01	0.02	0.00	0.87	-	-	-
Light but partly physically active work	Physical activity	25005.00	0.00	0.01	0.54	0.00	0.97	-	-	-
Light and physically active work	Physical activity	25005.00	-0.01	0.01	0.12	0.49	0.16	-	-	-
Sometimes physically straining work	Physical activity	25005.00	0.01	0.01	0.12	0.00	0.37	-	-	-
Frequency of walking during leisure time	Physical activity	24730.00	0.01	0.00	0.00	0.00	0.62	-	-	-
Frequency of cycling during leisure time	Physical activity	20187.00	0.01	0.00	0.00	0.00	0.40	0.16	0.38	44.00
Frequency of dancing during leisure time	Physical activity	7793.00	-0.01	0.01	0.38	0.00	0.99	-	-	-
Frequency of shoveling snow during leisure time	Physical activity	8542.00	0.02	0.01	0.02	0.29	0.24	-	-	-
Frequency of gardening during leisure time	Physical activity	8419.00	0.00	0.01	0.70	0.50	0.16	-	-	-
Frequency of hunting or fishing during leisure time	Physical activity	8341.00	0.00	0.01	0.46	0.00	0.47	-	-	-
Frequency of picking berries or mushrooms during leisure time	Physical activity	8445.00	0.01	0.01	0.18	0.00	0.85	-	-	-
Changed everyday exercise during the last year	Physical activity	9031.00	0.01	0.01	0.33	0.00	0.54	-	-	-
Everyday exercise satisfaction	Physical activity	8996.00	0.01	0.01	0.01	0.00	0.98	-	-	-
Exercise during the last three months	Physical activity	25474.00	0.02	0.00	0.00	0.00	0.82	0.16	0.36	40.00
If you exercise, change in exercise habits during the last year	Physical activity	7950.00	0.01	0.01	0.31	0.39	0.20	-	-	-
Amount of exercise during the last 12 months	Physical activity	16940.00	0.03	0.00		0.16	0.28	-	-	-
Bregott on bread	Food	23462.00	0.00	0.00	0.65	0.35	0.22	-	-	-
Whole grain crisp bread	Food	23462.00	0.00	0.00	0.21	0.00	0.79	-	-	-
Whole grain soft bread	Food	23462.00	0.00	0.00	0.75	0.00	0.33	-	-	-
White (soft) bread, thin crisp bread	Food	23462.00	-0.01	0.00	0.12	0.00	0.94	-	-	-
Coffee rolls/buns, rusk	Food	23462.00	-0.02	0.00	0.00	0.00	0.51	0.17	0.36	14.00
Cheese 28%	Food	23462.00	0.01	0.00	0.00	0.00	0.37	-	-	-
Cheese 10-17%	Food	23462.00	0.00	0.00	0.14	0.53	0.14	-	-	-
Soft cheese	Food	2566.00	0.00	0.01		0.00	0.32	-	-	-
Soft whey cheese	Food	2566.00	0.00	0.01		0.00	0.75	-	-	-
Sausage, liver pate on bread	Food	23462.00	0.00	0.00	0.25	0.76	0.04	-	-	-
Meat on bread	Food	23462.00	0.00	0.00	0.16	0.00	0.55	-	-	-
Butter on bread	Food	23462.00	0.00	0.00	0.08	0.63	0.10	-	-	-

Oatflake, whole wheat, rye or barley porridge	Food	23462.00	0.00	0.00	0.60	0.00	0.84	-	-	-
Rosehip, sweet syrup soup	Food	23462.00	-0.01	0.00	0.00	0.61	0.11	-	-	-
Sour milk, yoghurt (3% fat)	Food	23462.00	0.01	0.00	0.04	0.63	0.10	-	-	-
Sour milk, yoghurt (low fat)	Food	23462.00	0.00	0.00	0.09	0.52	0.15	-	-	-
Fiber cereals	Food	23462.00	0.00	0.00	0.30	0.44	0.18	-	-	-
Corn flakes	Food	23462.00	-0.01	0.00	0.00	0.00	0.90	-	-	-
Berries (fresh or frozen)	Food	23462.00	0.00	0.00	0.36	0.00	0.72	-	-	-
Apple, pear, peach, orange, mandarin and grapefruit	Food	23462.00	0.00	0.00	0.87	0.28	0.24	-	-	-
Ba	Food	23462.00	-0.01	0.00	0.05	0.00	0.62	-	-	-
Root vegetables and carrot	Food	23462.00	0.00	0.00	0.14	0.00	0.56	-	-	-
Low fat margarine on bread	Food	23462.00	-0.01	0.00	0.00	0.00	0.34	-	-	-
Tomato and cucumber	Food	23462.00	0.01	0.00	0.00	0.00	0.72	-	-	-
White cabbage, lettuce, lettuce cabbage, spich, borecole and broccoli	Food	23462.00	0.01	0.00	0.00	0.00	0.65	0.17	0.36	28.00
Mixed frozen vegetables	Food	2566.00	0.00	0.01		0.00	0.42	-	-	-
Boiled or baked potato	Food	23462.00	-0.01	0.00	0.02	0.00	0.86	-	-	-
Fried potatoes and pommes frites	Food	23462.00	0.00	0.00	0.39	0.00	0.87	-	-	-
Mashed potato	Food	2566.00	0.01	0.01		0.00	0.84	-	-	-
Potato salad	Food	2566.00	0.00	0.01		0.38	0.20	-	-	-
Rice	Food	23462.00	0.00	0.00	0.17	0.00	0.37	-	-	-
Pasta	Food	23462.00	0.00	0.00	0.17	0.58	0.12	-	-	-
Brown beans and pea soup	Food	23462.00	0.01	0.00	0.01	0.00	0.36	-	-	-
Margarine on bread	Food	23462.00	0.00	0.00	0.27	0.73	0.05	-	-	-
Blota (broth + bread)	Food	2566.00	0.01	0.01		0.00	0.49	-	-	-
Pancake, waffle and Swedish dumpling	Food	23462.00	0.00	0.00	0.25	0.00	0.41	-	-	-
Pizza	Food	23462.00	0.00	0.00	0.50	0.28	0.24	-	-	-
Minced meat dishes	Food	23462.00	0.01	0.00	0.00	0.65	0.09	-	-	-
Meat stew	Food	23462.00	0.01	0.00	0.00	0.00	0.95	0.17	0.36	23.00
Steak, chop, etc.	Food	23462.00	0.01	0.00	0.00	0.00	0.83	-	-	-
Bacon	Food	23462.00	0.01	0.00	0.00	0.00	0.33	0.17	0.36	19.00
Sausage as main dish	Food	23462.00	0.00	0.00	0.15	0.00	0.86	-	-	-
Hamburger	Food	23462.00	0.00	0.00	0.96	0.00	0.53	-	-	-
White meat (poultry)	Food	23462.00	0.01	0.00	0.00	0.63	0.10	-	-	-
Butter for cooking	Food	23462.00	0.01	0.00	0.00	0.00	0.86	0.17	0.36	22.00
Blood based food	Food	2566.00	0.01	0.01		0.00	0.92	-	-	-
Liver and kidney	Food	2566.00	0.00	0.01		0.47	0.17	-	-	-
Lean fish (e.g. perch, bass, cod)	Food	23462.00	0.00	0.00	0.08	0.00	0.81	-	-	-
Fatty fish (e.g. herring, whitefish, salmon)	Food	23462.00	0.01	0.00	0.00	0.50	0.16	-	-	-
Shellfish (e.g. shrimps, scallops)	Food	2566.00	0.00	0.01		0.36	0.21	-	-	-
Salty fish	Food	23462.00	0.01	0.00	0.00	0.00	0.91	-	-	-
Smoked fish/meat	Food	23462.00	0.01	0.00	0.00	0.75	0.05	-	-	-
Ice cream	Food	23462.00	0.00	0.00	0.35	0.52	0.15	-	-	-
Sweets	Food	23462.00	-0.01	0.00	0.02	0.00	0.79	-	-	-
Sugar, honey, marmelade and jam	Food	23462.00	-0.01	0.00	0.00	0.00	0.72	0.17	0.36	13.00
Margarine for cooking	Food	23462.00	0.00	0.00	0.11	0.19	0.27	-	-	-
Cookies and pastry	Food	23462.00	-0.01	0.00	0.00	0.00	0.44	0.17	0.36	17.00
Chips, popcorn and salted nuts	Food	23462.00	0.00	0.00	0.50	0.56	0.13	-	-	-
Low fat milk (0.5%)	Beverage	23462.00	-0.01	0.00	0.01	0.00	0.77	-	-	-
Milk, sour milk (1.5%)	Beverage	23462.00	-0.01	0.00	0.01	0.00	1.00	-	-	-
Milk, sour milk (3%)	Beverage	23462.00	0.00	0.00	0.79	0.00	0.55	-	-	-
Sodas, soft drinks and juice	Beverage	23462.00	-0.01	0.00	0.00	0.00	0.61	-	-	-
Brewed (filtered) coffee	Beverage	23462.00	0.01	0.00	0.00	0.00	0.36	-	-	-
Boiled coffee	Beverage	23462.00	0.00	0.00	0.51	0.00	0.91	-	-	-
Tea	Beverage	23462.00	0.00	0.00	0.11	0.00	0.60	-	-	-
Light beer	Alcohol	23462.00	0.02	0.00	0.00	0.00	0.98	0.17	0.36	31.00
Oil for cooking	Food	23462.00	0.00	0.00	0.44	0.00	0.49	-	-	-
Medium beer	Alcohol	23462.00	0.02	0.00	0.00	0.00	0.66	0.17	0.35	20.00
Strong beer	Alcohol	23462.00	0.02	0.00	0.00	0.00	0.69	0.17	0.36	8.00
Wine	Alcohol	23462.00	0.04	0.00	0.00	0.00	0.73	0.17	0.36	5.00
Liquor and spirits	Alcohol	23462.00	0.02	0.00	0.00	0.74	0.05	0.17	0.36	12.00
Salad dressing with oil	Food	23462.00	0.01	0.00	0.02	0.00	0.72	-	-	-

Cream, creme fraiche, sour cream	Food	23462.00	0.01	0.00	0.00	0.55	0.14	-	-	-
Average portion size of vegetables based on photographic illustration of four sizes (smallest to largest)	Food	23462.00	0.01	0.00	0.00	0.00	0.97	0.16	0.35	38.00
Overall state of health during the last year	General health	25991.00	0.03	0.00	0.00	0.00	0.57	0.16	0.36	37.00
Overall state of health compared to others your age	General health	7362.00	0.02	0.01	0.00	0.10	0.29	-	-	-
Parents or siblings had a cerebral hemorrhage/thrombosis or cardiac infarction before the age of 60	General health	25430.00	-0.02	0.01	0.01	0.00	0.35	-	-	-
Teetotaler	Alcohol	9016.00	-0.04	0.02	0.02	0.00	0.79	-	-	-
Feel the need to reduce alcohol consumption	Alcohol	23969.00	0.07	0.01	0.00	0.00	0.91	0.16	0.35	36.00
Feel uneasy or guilty because of your way of drinking	Alcohol	8046.00	0.04	0.02	0.02	0.00	0.58	-	-	-
Frequency of alcohol consumption	Alcohol	17039.00	0.06	0.00		0.77	0.04	-	-	-
Amount of alcohol drunk in a day	Alcohol	16240.00	0.02	0.00		0.00	0.71	-	-	-
Frequency of drinking six or more glasses at the same occasion	Alcohol	16536.00	0.03	0.00		0.51	0.15	-	-	-
Times during last year that you felt guilty because of your drinking	Alcohol	16513.00	0.03	0.00		0.14	0.28	-	-	-
Iron intake (mg/day)	Nutrients	23462.00	0.00	0.00	0.86	0.00	0.68	-	-	-
Iodine intake (ug/day)	Nutrients	23462.00	0.01	0.00	0.07	0.00	0.79	-	-	-
Calcium intake (mg/day)	Nutrients	23462.00	-0.01	0.00	0.01	0.00	0.73	-	-	-
Potassium intake (mg/day)	Nutrients	23462.00	0.00	0.00	0.51	0.00	0.41	-	-	-
Beta-carotene intake (mg/day)	Nutrients	23462.00	0.01	0.00	0.07	0.00	0.57	-	-	-
Cholesterol intake (g/day)	Nutrients	23462.00	0.02	0.00	0.00	0.00	0.97	0.17	0.36	11.00
Carbohydrates intake (g/day)	Nutrients	23462.00	-0.03	0.00	0.00	0.00	0.33	0.17	0.36	9.00
Average portion size of meat/fish based on photographic illustration of four sizes (smallest to largest)	Food	23462.00	0.01	0.00	0.00	0.68	0.08	-	-	-
Breakfast habits: Only coffee/tea for breakfast vs not breakfast at all	Food	1793.00	0.07	0.02	0.00	0.00	0.40	-	-	-
Breakfast habits: Coffee/tea and wheat buns or rusk for breakfast vs not breakfast at all	Food	1211.00	0.02	0.03	0.42	0.00	0.38	-	-	-
Breakfast habits: Porridge w/o sandwich for breakfast vs not breakfast at all	Food	3656.00	0.05	0.02	0.02	0.00	0.48	-	-	-
Breakfast habits: Gruel w/o sandwich for breakfast vs not breakfast at all	Food	1055.00	0.00	0.03	0.97	0.00	0.82	-	-	-
Eat breakfast from 2000	Food	19082.00	-0.01	0.01	0.49	0.00	0.53	-	-	-
Enterodiol intake (ug/day)	Nutrients	23462.00	-0.01	0.00	0.05	0.00	0.91	-	-	-
Enterolactone intake (ug/day)	Nutrients	23462.00	0.00	0.00	0.72	0.15	0.28	-	-	-
Equol intake (ug/day)	Nutrients	23462.00	-0.01	0.00	0.00	0.14	0.28	-	-	-
Lariciresinol intake (ug/day)	Nutrients	23462.00	0.00	0.00	0.21	0.10	0.29	-	-	-
Matairesinol intake (ug/day)	Nutrients	23462.00	0.01	0.00	0.01	0.16	0.27	-	-	-
Medioresinol intake (ug/day)	Nutrients	23462.00	0.00	0.00	0.84	0.00	0.42	-	-	-
Pinoresinol intake (ug/day)	Nutrients	23462.00	0.01	0.00	0.03	0.00	0.33	-	-	-
Secoisolariciresinol intake (ug/day)	Nutrients	23462.00	0.03	0.00	0.00	0.00	0.48	0.17	0.35	10.00

Sum of all ligns intake (ug/day)	Nutrients	23462.00	0.00	0.00	0.28	0.00	0.32	-	-	-
Syringaresinol intake (ug/day)	Nutrients	23462.00	0.00	0.00	0.90	0.00	0.32	-	-	-
Sum of Lariciresinol, Matairesinol, Pinoresinol, Secoisolariciresinol intake (ug/day)	Nutrients	23462.00	0.01	0.00	0.00	0.00	0.38	-	-	-
Satisfaction with home and family situation	Psychosocial	23096.00	0.00	0.00	0.43	0.00	0.36	-	-	-
Appetite status	Psychosocial	23101.00	0.01	0.00	0.00	0.23	0.25	-	-	-
Mood status	Psychosocial	23070.00	0.01	0.00	0.07	0.59	0.12	-	-	-
Energy status	Psychosocial	23063.00	0.02	0.00	0.00	0.00	0.83	0.18	0.34	3.00
Patience status	Psychosocial	23075.00	0.01	0.00	0.03	0.09	0.30	-	-	-
Confidence status	Psychosocial	23049.00	0.00	0.00	0.88	0.00	0.93	-	-	-
Sleep status	Sleep	23104.00	0.00	0.00	0.60	0.43	0.19	-	-	-
Do you feel important and appreciated outside your home?	Psychosocial	23101.00	0.01	0.00	0.00	0.00	0.54	-	-	-
Do you feel important and appreciated in your home?	Psychosocial	22935.00	0.01	0.00	0.00	0.51	0.15	-	-	-
Satisfaction with accomodation	Psychosocial	23117.00	0.00	0.00	0.56	0.84	0.01	-	-	-
Satisfaction with work situation	Psychosocial	22809.00	0.00	0.00	0.09	0.00	0.53	-	-	-
Satisfaction with economy	Psychosocial	23088.00	0.01	0.00	0.01	0.00	0.89	-	-	-
Satisfaction with leisure time	Psychosocial	23058.00	0.01	0.00	0.01	0.00	0.94	-	-	-
Hearing status	General health	23109.00	0.01	0.00	0.01	0.00	0.96	-	-	-
Vision status	General health	23083.00	0.01	0.00	0.07	0.00	0.95	-	-	-
Memory status	Psychosocial	23036.00	0.01	0.00	0.01	0.00	0.71	-	-	-
Fitness status	Physical activity	23076.00	0.03	0.00	0.00	0.00	0.34	0.18	0.34	2.00
Magnesium intake (mg/day)	Nutrients	23462.00	0.01	0.00	0.01	0.22	0.26	-	-	-
Saturated fat intake (g/day)	Nutrients	23462.00	0.01	0.00	0.00	0.00	0.98	0.17	0.36	16.00
Monounsaturated fat intake (g/day)	Nutrients	23462.00	0.01	0.00	0.00	0.35	0.21	0.17	0.36	32.00
Monosaccharides intake (g/day)	Nutrients	23462.00	-0.01	0.00	0.00	0.56	0.13	-	-	-
Sodium intake (mg/day)	Nutrients	23460.00	0.01	0.00	0.00	0.00	0.74	0.17	0.36	27.00
Vitamin B3 intake (mg/day)	Nutrients	23462.00	0.01	0.00	0.00	0.00	0.77	0.17	0.36	29.00
Cambridge physical activity index	Physical activity	24456.00	0.02	0.00	0.00	0.00	0.37	0.16	0.35	39.00
Polyunsaturated fat intake (g/day)	Nutrients	23462.00	0.00	0.00	0.13	0.00	1.00	-	-	-
Average portion size of potatoes/rice/pasta based on photographic illustration of four sizes (smallest to largest)	Food	23462.00	-0.01	0.00	0.00	0.00	0.55	-	-	-
Total protein intake (g/day)	Nutrients	23462.00	0.01	0.00	0.00	0.00	0.49	-	-	-
Animal based protein intake (g/day)	Nutrients	23462.00	0.01	0.00	0.00	0.00	0.62	-	-	-
Plant based protein intake (g/day)	Nutrients	23462.00	-0.01	0.00	0.01	0.00	0.73	-	-	-
Vitamin A intake (mg/day)	Nutrients	23462.00	-0.01	0.00	0.06	0.00	0.78	-	-	-
Sucrose intake (g/day)	Nutrients	23462.00	-0.02	0.00	0.00	0.00	0.50	0.17	0.36	7.00
Cohabitation: Live alone vs Only one adult (spouse, partner)	Social	14649.00	-0.02	0.01	0.03	0.00	0.39	-	-	-
Cohabitation: Live alone vs Only children	Social	4197.00	-0.02	0.02	0.29	0.00	0.48	-	-	-
Cohabitation: Live alone vs Adult and children	Social	12958.00	-0.02	0.01	0.06	0.60	0.11	-	-	-
Cohabitation: Live alone vs Other/others	Social	3380.00	-0.01	0.03	0.66	0.19	0.27	-	-	-
Selenium intake (ug/day)	Nutrients	23462.00	0.01	0.00	0.00	0.00	0.68	0.17	0.36	30.00
Self rate of overall health	General health	18649.00	0.02	0.00	0.00	0.00	0.88	-	-	-

For how much of the time during the last four weeks has your physical health or your emotiol problems limited your ability to interact with others?	General health	18513.00	-0.01	0.00	0.00	0.00	0.73	-	-	-
Get sick more often than other people	General health	18598.00	-0.01	0.00	0.00	0.00	0.71	-	-	-
As healthy as anyone	General health	18545.00	0.01	0.00	0.00	0.00	0.35	-	-	-
Worsen in health in the future	General health	18541.00	-0.01	0.00	0.00	0.00	0.59	-	-	-
Excellent health	General health	18596.00	0.02	0.00	0.00	0.00	0.88	-	-	-
Self rate of overall health compared to a year ago	General health	18471.00	0.00	0.00	0.82	0.00	0.44	-	-	-
Physical limitation to participate in steneous activities: running, lifting heavy objects, taking part in physically demanding sports	General health	18607.00	-0.02	0.00	0.00	0.00	0.75	-	-	-
Physical limitation to participate in moderately demanding activities: moving a table, vacuuming, walking in the forest or gardening	General health	18592.00	-0.01	0.00	0.00	0.00	0.44	-	-	-
Physical limitation to participate in moderately demanding activities: lifting or carrying grocery bags	General health	18576.00	-0.01	0.00	0.00	0.00	0.87	-	-	-
Physical limitation to participate in moderately demanding activities: walking up several stairs	General health	18587.00	-0.01	0.00	0.00	0.00	0.81	-	-	-
Physical limitation to participate in moderately demanding activities: bending down or kneeling	General health	18566.00	-0.01	0.00	0.03	0.00	0.64	-	-	-
Physical limitation to participate in moderately demanding activities: walking more than two km	General health	18597.00	-0.01	0.00	0.00	0.00	0.51	-	-	-
Physical limitation that reduced the normal time spent at work or in other activities during the last four weeks	General health	18621.00	-0.04	0.01	0.00	0.00	0.96	-	-	-
Physical limitation that made you do less than you wanted during the last four weeks	General health	18613.00	-0.03	0.01	0.00	0.00	0.52	-	-	-
Physical limitation that made you not being able to perform certain work tasks or other activities during the last four weeks	General health	18539.00	-0.03	0.01	0.00	0.00	0.95	-	-	-
Physical limitation that limited your ability to perform certain work tasks or other activities during the last four weeks	General health	18561.00	-0.03	0.01	0.00	0.00	0.63	-	-	-
Emotiol problems that made you do less than you wanted during the last four weeks	General health	18631.00	-0.01	0.01	0.49	0.00	0.61	-	-	-
Extent to what your physical and emotiol health disrupted your usual social life during the last four weeks	General health	18625.00	-0.01	0.00	0.00	0.00	0.65	-	-	-
Pain during the last four weeks	General health	18610.00	-0.01	0.00	0.00	0.00	0.77	-	-	-
How much has the pain during the last four weeks disturbed your normal work?	General health	18574.00	-0.01	0.00	0.00	0.00	0.80	-	-	-

For how much of the time during the last four weeks have you felt really alert and strong?	General health	18575.00	0.02	0.00	0.00	0.00	0.65	-	-	-
For how much of the time during the last four weeks have you felt very nervous?	General health	18603.00	0.00	0.00	0.90	0.80	0.03	-	-	-
For how much of the time during the last four weeks have you felt so depressed that nothing could cheer you up?	General health	18623.00	0.00	0.00	0.53	0.08	0.30	-	-	-
For how much of the time during the last four weeks have you felt calm and serene?	General health	18614.00	0.00	0.00	0.19	0.20	0.26	-	-	-
For how much of the time during the last four weeks have you felt full of energy?	General health	18591.00	0.02	0.00	0.00	0.00	0.48	-	-	-
For how much of the time during the last four weeks have you felt gloomy and sad?	General health	18596.00	0.00	0.00	0.32	0.00	0.79	-	-	-
For how much of the time during the last four weeks have you felt worn out?	General health	18568.00	-0.01	0.00	0.00	0.00	0.93	-	-	-
For how much of the time during the last four weeks have you felt happy?	General health	18605.00	0.01	0.00	0.01	0.85	0.01	-	-	-
For how much of the time during the last four weeks have you felt tired?	General health	18624.00	-0.02	0.00	0.00	0.00	0.85	0.22	0.22	1.00
Long-term sickness	General health	25348.00	-0.02	0.01	0.00	0.00	0.88	-	-	-
Work shifts/weekends	Psychosocial	25367.00	-0.01	0.01	0.20	0.00	0.71	-	-	-
Risk of sleeping while sitting and reading	Sleep	16674.00	0.00	0.00		0.00	0.85	-	-	-
Risk of sleeping while watching TV	Sleep	16855.00	0.00	0.00		0.00	0.83	-	-	-
Risk of sleeping while sitting ictive in a public place	Sleep	16752.00	0.00	0.00		0.00	0.86	-	-	-
Risk of sleeping as a passenger in a car for one hour without break	Sleep	16676.00	0.00	0.00		0.00	0.35	-	-	-
Risk of sleeping while lying down resting in the afternoon	Sleep	16800.00	-0.01	0.00		0.46	0.18	-	-	-
Risk of sleeping while sitting still after having lunch	Sleep	16802.00	0.00	0.00		0.00	0.55	-	-	-
Snore during sleep	Sleep	15687.00	-0.01	0.00		0.75	0.05	-	-	-
Breath-holds during sleep	Sleep	11709.00	-0.01	0.00		0.00	0.50	-	-	-
Number of cigarretes smoked per day (in groups)	Tobacco use	21109.00	-0.02	0.00	0.00	0.62	0.10	0.16	0.35	43.00
Years smoking	Tobacco use	22395.00	-0.02	0.00	0.00	0.00	0.41	0.15	0.36	46.00
Smoking status: Smokers vs non-smokers	Tobacco use	16291.00	-0.07	0.01	0.00	0.77	0.04	0.15	0.34	45.00
Smoking status: Former smokers vs non-smokers	Tobacco use	18813.00	0.01	0.01	0.11	0.00	0.54	-	-	-
Smoking status: Former occasiol smokers vs non-smokers	Tobacco use	15562.00	0.02	0.01	0.05	0.00	0.71	-	-	-
Number of snuff boxes per week	Tobacco use	25344.00	0.01	0.00	0.00	0.16	0.27	-	-	-
Snuff status: Snuff users vs non-snuff users	Tobacco use	22021.00	0.03	0.01	0.00	0.00	0.57	0.16	0.39	33.00
Snuff status: Former snuff users vs non-snuff users	Tobacco use	20723.00	0.02	0.01	0.01	0.00	0.53	-	-	-
Years using snuff	Tobacco use	24637.00	0.01	0.00	0.00	0.06	0.30	0.16	0.39	34.00
Participation in associations or voluntary organisations	Social	25755.00	0.01	0.01	0.27	0.00	0.56	-	-	-
Participation in sports or physical exercise associations	Social	11563.00	0.03	0.01	0.00	0.52	0.15	-	-	-

Participation in study circles	Social	11563.00	0.01	0.01	0.26	0.00	0.84	-	-	-
Participation in other association	Social	11563.00	-0.02	0.01	0.00	0.49	0.16	-	-	-
People to ask for help apart from the ones at home	Social	25898.00	0.01	0.01	0.23	0.66	0.09	-	-	-
Number of friends that can come to your home at any time and feel at home	Social	25899.00	0.00	0.00	0.08	0.00	0.79	-	-	-
Number of social contacts with the same interests as you	Social	25971.00	0.01	0.00	0.06	0.42	0.19	-	-	-
Would you say that the number of people that you meet in your everyday life is enough or would you like to meet more or fewer people?	Social	25985.00	0.00	0.00	0.37	0.00	0.96	-	-	-
Close relationship with anyone	Social	25925.00	0.00	0.00	0.31	0.00	0.88	-	-	-
Frequency of engaging in clubs, associations or study circles	Social	15586.00	0.00	0.00	0.22	0.00	0.82	-	-	-
Number of social interactions during a normal week	Social	25994.00	0.00	0.00	0.17	0.00	0.64	-	-	-
Support from others	Social	26007.00	0.00	0.00	0.31	0.00	0.70	-	-	-
Number of people with whom you can speak openly	Social	25975.00	0.00	0.00	1.00	0.26	0.25	-	-	-
Receive hugs to comfort and support you	Social	25735.00	-0.01	0.01	0.11	0.00	0.48	-	-	-
Stigmasterol intake (mg/day)	Nutrients	23462.00	0.00	0.00	0.34	0.00	0.45	-	-	-
Tiamin intake (mg/day)	Nutrients	23462.00	0.00	0.00	0.11	0.00	0.47	-	-	-
Vitamin E intake (mg/day)	Nutrients	23462.00	0.00	0.00	0.53	0.00	0.64	-	-	-
Trans fat intake (g/day)	Nutrients	23462.00	0.02	0.01	0.00	0.00	0.66	-	-	-
Sum of phytosterols intake (mg/day)	Nutrients	23462.00	0.00	0.00	0.31	0.00	0.38	-	-	-
Zinc intake (mg/day)	Nutrients	23462.00	0.00	0.00	0.32	0.00	0.79	-	-	-

Supplementary Table 9. Linear mixed model association results for LDL cholesterol

Description	Group	N	Effect estimate	S.E.	p-value	I^2	Q p-value	marginal R2	conditional R2	R2 rank
Alcohol intake (g/day)	Alcohol	23348.00	0.01	0.01	0.29	0.00	0.59	-	-	-
Permanent employment	Psychosocial	22774.00	0.02	0.01	0.26	0.00	0.67	-	-	-
Self-employed	Psychosocial	22774.00	-0.01	0.02	0.76	0.00	0.85	-	-	-
Distance to work in kilometers (one way)	Physical activity	21844.00	0.00	0.01	0.54	0.00	0.37	-	-	-
Last time a colleague visited you at home	Psychosocial	25138.00	0.02	0.01	0.01	0.00	0.63	-	-	-
Job demands to work very fast	Psychosocial	25461.00	0.00	0.01	0.72	0.00	0.88	-	-	-
Frequency of social contacts with colleagues during leisure time	Psychosocial	24693.00	0.00	0.01	0.60	0.00	0.34	-	-	-
High physical demand from job	Physical activity	25531.00	0.01	0.01	0.18	0.00	0.34	-	-	-
Enough time for job assignments	Psychosocial	25335.00	0.00	0.01	0.44	0.37	0.21	-	-	-
Control over planning and execution of the workday	Psychosocial	25463.00	0.01	0.01	0.02	0.00	0.74	-	-	-
Ingenuity or creativity demand from job	Psychosocial	25317.00	0.01	0.01	0.30	0.00	0.70	-	-	-
Frequent social contacts with colleagues during work	Psychosocial	25194.00	0.00	0.01	0.47	0.00	0.60	-	-	-
Contradictory demands in job	Psychosocial	25306.00	0.00	0.01	0.46	0.00	0.42	-	-	-
Possibility to leave your work for a while to speak with a colleague	Psychosocial	25207.00	0.01	0.01	0.03	0.00	0.75	-	-	-
Learn new things at job	Psychosocial	25384.00	0.01	0.01	0.05	0.76	0.04	-	-	-
High mental demand from job	Psychosocial	25241.00	0.00	0.01	0.56	0.00	0.95	-	-	-
Repetitive job	Psychosocial	25405.00	-0.01	0.01	0.18	0.50	0.16	-	-	-
Skill demand from job	Psychosocial	25375.00	0.00	0.01	0.99	0.00	0.77	-	-	-
Possibility to speak with colleagues during breaks	Psychosocial	25371.00	-0.01	0.01	0.13	0.00	0.46	-	-	-
Control over own work assignment	Psychosocial	25451.00	0.01	0.01	0.28	0.04	0.31	-	-	-
Vitamin C intake (mg/day)	Nutrients	23348.00	-0.03	0.01	0.00	0.00	0.44	-	-	-
Vitamin B12 intake (ug/day)	Nutrients	23348.00	0.01	0.01	0.10	0.00	0.37	-	-	-
Vitamin B2 intake (ug/day)	Nutrients	23348.00	-0.02	0.01	0.01	0.00	0.49	-	-	-
Vitamin B6 intake (mg/day)	Nutrients	23348.00	0.00	0.01	0.83	0.00	0.37	-	-	-
Informed of having high blood pressure	General health	25851.00	-0.02	0.01	0.12	0.00	0.43	-	-	-
Beta-sitostanol intake (mg/day)	Nutrients	23348.00	0.01	0.01	0.08	0.00	0.89	-	-	-
Beta-sitosterol intake (mg/day)	Nutrients	23348.00	0.00	0.01	0.58	0.00	0.81	-	-	-
Marital status: Single vs Married/partner	Social	23470.00	0.03	0.02	0.23	0.00	0.62	-	-	-
Marital status: Single vs Divorced/separated	Social	4570.00	-0.01	0.03	0.79	0.00	0.36	-	-	-
Marital status: Single vs Widow/widower	Social	2934.00	-0.05	0.06	0.46	0.00	0.42	-	-	-
Campestanol intake (mg/day)	Nutrients	23348.00	0.00	0.01	0.54	0.00	0.72	-	-	-
Campesterol intake (mg/day)	Nutrients	23348.00	0.02	0.01	0.00	0.00	0.62	-	-	-
Parents or siblings have diabetes	General health	25294.00	-0.02	0.02	0.27	0.08	0.30	-	-	-
Disaccharides intake (g/day)	Nutrients	23348.00	0.00	0.01	0.56	0.52	0.15	-	-	-
Vitamin D intake (ug/day)	Nutrients	23348.00	0.03	0.01	0.00	0.00	0.80	0.08	0.55	7.00
Total energy intake (kcal/day)	Nutrients	23348.00	-0.03	0.01	0.00	0.00	0.67	-	-	-
Formic acid intaje (g/day)	Nutrients	23348.00	0.04	0.01	0.00	0.00	0.37	0.08	0.55	5.00
Pentadecanoic acid intake (g/day)	Nutrients	23348.00	0.03	0.01	0.00	0.00	0.39	0.08	0.55	9.00
Palmitic acid intake (g/day)	Nutrients	23348.00	0.05	0.01	0.00	0.20	0.26	0.08	0.55	3.00

Heptadecanoic acid intake (g/day)	Nutrients	23348.00	0.03	0.01	0.00	0.00	0.39	0.08	0.55	10.00
Linoleic acid intake (g/day)	Nutrients	23348.00	0.01	0.01	0.26	0.00	1.00	-	-	-
Linolenic acid intake (g/day)	Nutrients	23348.00	0.02	0.01	0.01	0.00	0.75	-	-	-
Arachidonic acid (ARA) intake (g/day)	Nutrients	23347.00	0.02	0.01	0.00	0.00	0.45	-	-	-
Eicosapentaenoic acid (EPA) intake (g/day)	Nutrients	23347.00	0.02	0.01	0.00	0.43	0.18	-	-	-
Docosahexaenoic acid (DHA) intake (g/day)	Nutrients	23348.00	0.03	0.01	0.00	0.46	0.17	-	-	-
Fat intake (g/day)	Nutrients	23348.00	0.04	0.01	0.00	0.00	0.46	0.08	0.55	8.00
Fibre intake (g/day)	Nutrients	23348.00	-0.03	0.01	0.00	0.00	0.46	-	-	-
Folic acid intake (ug/day)	Nutrients	23348.00	-0.02	0.01	0.01	0.00	0.43	-	-	-
Phosphate intake (mg/day)	Nutrients	23348.00	0.00	0.01	0.97	0.00	0.60	-	-	-
Whole grain intake (g/day)	Food	23348.00	0.00	0.01	0.73	0.00	0.72	-	-	-
Travel to work: Walk to work vs passive travel to work	Physical activity	17127.00	0.02	0.02	0.37	0.00	0.61	-	-	-
Travel to work: Cycle to work vs passive travel to work	Physical activity	19005.00	-0.04	0.02	0.03	0.00	0.77	-	-	-
Travel to work: Irregular travel mode to work vs passive travel to work	Physical activity	16838.00	0.01	0.02	0.54	0.76	0.04	-	-	-
Time spent in a week in moderately steneous activities	Physical activity	16400.00	-0.01	0.01		0.00	0.76	-	-	-
Sedentary or standing work	Physical activity	24880.00	-0.02	0.01	0.14	0.00	0.76	-	-	-
Light but partly physically active work	Physical activity	24880.00	0.02	0.02	0.33	0.00	0.92	-	-	-
Light and physically active work	Physical activity	24880.00	0.00	0.01	0.78	0.00	0.40	-	-	-
Sometimes physically straining work	Physical activity	24880.00	0.01	0.01	0.66	0.00	0.97	-	-	-
Frequency of walking during leisure time	Physical activity	24604.00	0.00	0.01	0.57	0.57	0.13	-	-	-
Frequency of cycling during leisure time	Physical activity	20085.00	-0.02	0.01	0.01	0.00	0.69	-	-	-
Frequency of dancing during leisure time	Physical activity	7692.00	-0.02	0.01	0.07	0.25	0.25	-	-	-
Frequency of shoveling snow during leisure time	Physical activity	8421.00	0.00	0.01	0.93	0.00	0.43	-	-	-
Frequency of gardening during leisure time	Physical activity	8305.00	0.02	0.01	0.14	0.00	0.93	-	-	-
Frequency of hunting or fishing during leisure time	Physical activity	8229.00	0.01	0.01	0.50	0.61	0.11	-	-	-
Frequency of picking berries or mushrooms during leisure time	Physical activity	8330.00	0.02	0.01	0.08	0.00	0.58	-	-	-
Changed everyday exercise during the last year	Physical activity	8905.00	-0.04	0.01	0.00	0.00	0.33	-	-	-
Everyday exercise satisfaction	Physical activity	8871.00	-0.02	0.01	0.05	0.00	0.35	-	-	-
Exercise during the last three months	Physical activity	25345.00	-0.04	0.01	0.00	0.19	0.27	0.05	0.51	19.00
If you exercise, change in exercise habits during the last year	Physical activity	7836.00	-0.05	0.01	0.00	0.00	0.68	-	-	-
Amount of exercise during the last 12 months	Physical activity	16932.00	-0.02	0.01		0.38	0.20	-	-	-
Bregott on bread	Food	23348.00	0.03	0.01	0.00	0.54	0.14	-	-	-
Whole grain crisp bread	Food	23348.00	0.02	0.01	0.01	0.00	0.42	-	-	-
Whole grain soft bread	Food	23348.00	0.00	0.01	0.82	0.00	0.80	-	-	-
White (soft) bread, thin crisp bread	Food	23348.00	0.00	0.01	0.89	0.03	0.31	-	-	-
Coffee rolls/buns, rusk	Food	23348.00	0.00	0.01	0.66	0.00	0.94	-	-	-
Cheese 28%	Food	23348.00	0.00	0.01	0.65	0.00	0.56	-	-	-
Cheese 10-17%	Food	23348.00	-0.02	0.01	0.00	0.21	0.26	-	-	-
Soft cheese	Food	2539.00	-0.01	0.02		0.83	0.01	-	-	-
Soft whey cheese	Food	2539.00	-0.04	0.02		0.00	0.91	-	-	-
Sausage, liver pate on bread	Food	23348.00	-0.01	0.01	0.03	0.00	0.35	-	-	-

Meat on bread	Food	23348.00	0.00	0.01	0.92	0.00	0.46	-	-	-
Butter on bread	Food	23348.00	0.00	0.01	0.91	0.00	0.87	-	-	-
Oatflake, whole wheat, rye or barley porridge	Food	23348.00	-0.01	0.01	0.09	0.00	0.43	-	-	-
Rosehip, sweet syrup soup	Food	23348.00	-0.01	0.01	0.03	0.00	0.51	-	-	-
Sour milk, yoghurt (3% fat)	Food	23348.00	-0.01	0.01	0.20	0.53	0.15	-	-	-
Sour milk, yoghurt (low fat)	Food	23348.00	-0.02	0.01	0.02	0.00	0.68	-	-	-
Fiber cereals	Food	23348.00	-0.02	0.01	0.01	0.00	0.64	-	-	-
Corn flakes	Food	23348.00	-0.01	0.01	0.23	0.60	0.11	-	-	-
Berries (fresh or frozen)	Food	23348.00	-0.01	0.01	0.02	0.00	0.36	-	-	-
Apple, pear, peach, orange, mandarin and grapefruit	Food	23348.00	0.00	0.01	0.96	0.00	0.61	-	-	-
Ba	Food	23348.00	-0.03	0.01	0.00	0.00	0.83	0.08	0.55	15.00
Root vegetables and carrot	Food	23348.00	-0.02	0.01	0.03	0.00	0.62	-	-	-
Low fat margarine on bread	Food	23348.00	-0.02	0.01	0.00	0.00	0.49	-	-	-
Tomato and cucumber	Food	23348.00	-0.02	0.01	0.02	0.00	0.35	-	-	-
White cabbage, lettuce, lettuce cabbage, spich, borecole and broccoli	Food	23348.00	0.00	0.01	0.77	0.00	0.78	-	-	-
Mixed frozen vegetables	Food	2539.00	0.01	0.02		0.46	0.17	-	-	-
Boiled or baked potato	Food	23348.00	0.02	0.01	0.03	0.00	0.33	-	-	-
Fried potatoes and pommes frites	Food	23348.00	0.01	0.01	0.06	0.00	0.60	-	-	-
Mashed potato	Food	2539.00	-0.03	0.02		0.00	0.45	-	-	-
Potato salad	Food	2539.00	0.03	0.02		0.00	0.89	-	-	-
Rice	Food	23348.00	-0.02	0.01	0.01	0.90	0.00	-	-	-
Pasta	Food	23348.00	-0.01	0.01	0.18	0.79	0.03	-	-	-
Brown beans and pea soup	Food	23348.00	-0.02	0.01	0.00	0.00	0.69	-	-	-
Margarine on bread	Food	23348.00	-0.01	0.01	0.07	0.62	0.10	-	-	-
Blota (broth + bread)	Food	2539.00	0.03	0.03		0.00	0.47	-	-	-
Pancake, waffle and Swedish dumpling	Food	23348.00	0.00	0.01	0.96	0.00	0.32	-	-	-
Pizza	Food	23348.00	-0.02	0.01	0.00	0.00	0.40	-	-	-
Minced meat dishes	Food	23348.00	0.02	0.01	0.01	0.00	0.85	-	-	-
Meat stew	Food	23348.00	0.02	0.01	0.00	0.00	0.98	-	-	-
Steak, chop, etc.	Food	23348.00	0.02	0.01	0.00	0.00	0.87	-	-	-
Bacon	Food	23348.00	0.02	0.01	0.00	0.00	0.69	-	-	-
Sausage as main dish	Food	23348.00	0.01	0.01	0.12	0.76	0.04	-	-	-
Hamburger	Food	23348.00	0.00	0.01	0.81	0.61	0.11	-	-	-
White meat (poultry)	Food	23348.00	0.01	0.01	0.30	0.00	0.77	-	-	-
Butter for cooking	Food	23348.00	0.03	0.01	0.00	0.00	0.45	-	-	-
Blood based food	Food	2539.00	-0.07	0.02		0.66	0.08	-	-	-
Liver and kidney	Food	2539.00	0.00	0.02		0.00	0.61	-	-	-
Lean fish (e.g. perch, bass, cod)	Food	23348.00	0.01	0.01	0.27	0.64	0.10	-	-	-
Fatty fish (e.g. herring, whitefish, salmon)	Food	23348.00	0.03	0.01	0.00	0.49	0.16	-	-	-
Shellfish (e.g. shrimps, scallops)	Food	2539.00	0.01	0.02		0.00	1.00	-	-	-
Salty fish	Food	23348.00	0.01	0.01	0.05	0.00	0.67	-	-	-
Smoked fish/meat	Food	23348.00	0.00	0.01	0.94	0.31	0.23	-	-	-
Ice cream	Food	23348.00	0.01	0.01	0.04	0.81	0.02	-	-	-
Sweets	Food	23348.00	0.01	0.01	0.17	0.00	0.92	-	-	-
Sugar, honey, marmelade and jam	Food	23348.00	0.01	0.01	0.15	0.00	0.32	-	-	-
Margarine for cooking	Food	23348.00	0.03	0.01	0.00	0.00	0.36	-	-	-
Cookies and pastry	Food	23348.00	0.01	0.01	0.08	0.00	0.37	-	-	-
Chips, popcorn and salted nuts	Food	23348.00	0.02	0.01	0.01	0.00	0.91	-	-	-
Low fat milk (0.5%)	Beverage	23348.00	-0.03	0.01	0.00	0.00	0.90	0.08	0.55	17.00
Milk, sour milk (1.5%)	Beverage	23348.00	0.01	0.01	0.19	0.71	0.06	-	-	-
Milk, sour milk (3%)	Beverage	23348.00	0.02	0.01	0.00	0.49	0.16	-	-	-
Sodas, soft drinks and juice	Beverage	23348.00	0.00	0.01	0.51	0.74	0.05	-	-	-
Brewed (filtered) coffee	Beverage	23348.00	0.01	0.01	0.14	0.23	0.25	-	-	-
Boiled coffee	Beverage	23348.00	0.06	0.01	0.00	0.00	0.60	0.08	0.55	1.00
Tea	Beverage	23348.00	-0.03	0.01	0.00	0.00	0.60	-	-	-
Light beer	Alcohol	23348.00	0.03	0.01	0.00	0.58	0.12	-	-	-
Oil for cooking	Food	23348.00	0.00	0.01	0.85	0.00	0.59	-	-	-
Medium beer	Alcohol	23348.00	0.02	0.01	0.04	0.00	0.86	-	-	-
Strong beer	Alcohol	23348.00	0.01	0.01	0.09	0.28	0.24	-	-	-
Wine	Alcohol	23348.00	0.00	0.01	0.55	0.00	0.94	-	-	-

Liquor and spirits	Alcohol	23348.00	0.00	0.01	0.71	0.00	0.92	-	-	-
Salad dressing with oil	Food	23348.00	-0.02	0.01	0.01	0.00	0.49	-	-	-
Cream, creme fraiche, sour cream	Food	23348.00	0.03	0.01	0.00	0.00	0.86	0.08	0.55	13.00
Average portion size of vegetables based on photographic illustration of four sizes (smallest to largest)	Food	23348.00	-0.02	0.01	0.00	0.00	0.35	-	-	-
Overall state of health during the last year	General health	25859.00	-0.01	0.01	0.12	0.00	0.35	-	-	-
Overall state of health compared to others your age	General health	7246.00	-0.02	0.01	0.08	0.00	0.92	-	-	-
Parents or siblings had a cerebral hemorrhage/thrombosis or cardiac infarction before the age of 60	General health	25300.00	0.06	0.02	0.00	0.00	0.66	-	-	-
Teetotaler	Alcohol	8891.00	0.00	0.04	0.92	0.00	0.81	-	-	-
Feel the need to reduce alcohol consumption	Alcohol	23845.00	-0.03	0.02	0.16	0.44	0.18	-	-	-
Feel uneasy or guilty because of your way of drinking	Alcohol	7928.00	-0.03	0.04	0.40	0.00	0.68	-	-	-
Frequency of alcohol consumption	Alcohol	17032.00	-0.01	0.01		0.00	0.39	-	-	-
Amount of alcohol drunk in a day	Alcohol	16233.00	0.00	0.01		0.00	0.75	-	-	-
Frequency of drinking six or more glasses at the same occasion	Alcohol	16529.00	0.00	0.01		0.00	0.56	-	-	-
Times during last year that you felt guilty because of your drinking	Alcohol	16506.00	-0.01	0.01		0.00	0.69	-	-	-
Iron intake (mg/day)	Nutrients	23348.00	-0.04	0.01	0.00	0.69	0.07	-	-	-
Iodine intake (ug/day)	Nutrients	23348.00	0.01	0.01	0.10	0.02	0.31	-	-	-
Calcium intake (mg/day)	Nutrients	23348.00	-0.03	0.01	0.00	0.00	0.93	0.08	0.55	18.00
Potassium intake (mg/day)	Nutrients	23348.00	0.01	0.01	0.35	0.00	0.50	-	-	-
Beta-carotene intake (mg/day)	Nutrients	23348.00	-0.02	0.01	0.02	0.00	0.57	-	-	-
Cholesterol intake (g/day)	Nutrients	23348.00	0.04	0.01	0.00	0.00	0.99	0.08	0.55	14.00
Carbohydrates intake (g/day)	Nutrients	23348.00	-0.04	0.01	0.00	0.00	0.64	0.08	0.55	11.00
Average portion size of meat/fish based on photographic illustration of four sizes (smallest to largest)	Food	23348.00	0.03	0.01	0.00	0.00	0.83	0.05	0.51	20.00
Breakfast habits: Only coffee/tea for breakfast vs not breakfast at all	Food	1784.00	-0.08	0.06	0.18	0.52	0.15	-	-	-
Breakfast habits: Coffee/tea and wheat buns or rusk for breakfast vs not breakfast at all	Food	1201.00	-0.06	0.07	0.34	0.00	0.76	-	-	-
Breakfast habits: Porridge w/o sandwich for breakfast vs not breakfast at all	Food	3630.00	-0.15	0.05	0.00	0.00	0.60	-	-	-
Breakfast habits: Gruel w/o sandwich for breakfast vs not breakfast at all	Food	1049.00	-0.11	0.08	0.16	0.00	0.89	-	-	-
Eat breakfast from 2000	Food	19033.00	-0.09	0.02	0.00	0.00	0.78	-	-	-
Enterodiol intake (ug/day)	Nutrients	23348.00	-0.01	0.01	0.16	0.00	0.43	-	-	-
Enterolactone intake (ug/day)	Nutrients	23348.00	-0.01	0.01	0.05	0.60	0.11	-	-	-
Equol intake (ug/day)	Nutrients	23348.00	-0.03	0.01	0.00	0.00	0.88	0.08	0.55	16.00
Lariciresinol intake (ug/day)	Nutrients	23348.00	0.00	0.01	0.97	0.00	0.69	-	-	-
Matairesinol intake (ug/day)	Nutrients	23348.00	0.00	0.01	0.50	0.00	0.66	-	-	-
Medioresinol intake (ug/day)	Nutrients	23348.00	0.01	0.01	0.06	0.00	0.65	-	-	-

Pinoresinol intake (ug/day)	Nutrients	23348.00	-0.01	0.01	0.25	0.00	0.77	-	-	-
Secoisolariciresinol intake (ug/day)	Nutrients	23348.00	0.01	0.01	0.13	0.00	0.83	-	-	-
Sum of all ligns intake (ug/day)	Nutrients	23348.00	0.01	0.01	0.35	0.00	0.87	-	-	-
Syringaresinol intake (ug/day)	Nutrients	23348.00	0.01	0.01	0.22	0.00	0.75	-	-	-
Sum of Lariciresinol, Matairesinol, Pinoresinol, Secoisolariciresinol intake (ug/day)	Nutrients	23348.00	0.00	0.01	0.76	0.00	0.78	-	-	-
Satisfaction with home and family situation	Psychosocial	23000.00	0.02	0.01	0.01	0.00	0.46	-	-	-
Appetite status	Psychosocial	23004.00	0.02	0.01	0.02	0.00	0.94	-	-	-
Mood status	Psychosocial	22975.00	0.01	0.01	0.42	0.00	0.33	-	-	-
Energy status	Psychosocial	22966.00	-0.01	0.01	0.34	0.00	0.87	-	-	-
Patience status	Psychosocial	22978.00	0.00	0.01	0.54	0.00	0.37	-	-	-
Confidence status	Psychosocial	22953.00	0.01	0.01	0.23	0.53	0.15	-	-	-
Sleep status	Sleep	23007.00	0.00	0.01	0.53	0.00	0.48	-	-	-
Do you feel important and appreciated outside your home?	Psychosocial	23003.00	-0.01	0.01	0.29	0.00	0.59	-	-	-
Do you feel important and appreciated in your home?	Psychosocial	22839.00	0.01	0.01	0.20	0.18	0.27	-	-	-
Satisfaction with accomodation	Psychosocial	23021.00	0.01	0.01	0.20	0.00	0.70	-	-	-
Satisfaction with work situation	Psychosocial	22714.00	-0.01	0.01	0.27	0.00	0.72	-	-	-
Satisfaction with economy	Psychosocial	22993.00	0.00	0.01	0.72	0.00	0.49	-	-	-
Satisfaction with leisure time	Psychosocial	22963.00	0.00	0.01	0.84	0.00	0.99	-	-	-
Hearing status	General health	23012.00	0.00	0.01	0.84	0.00	0.74	-	-	-
Vision status	General health	22986.00	0.00	0.01	0.86	0.00	0.34	-	-	-
Memory status	Psychosocial	22938.00	0.00	0.01	0.62	0.00	0.72	-	-	-
Fitness status	Physical activity	22979.00	-0.03	0.01	0.00	0.62	0.10	-	-	-
Magnesium intake (mg/day)	Nutrients	23348.00	0.00	0.01	0.50	0.00	0.98	-	-	-
Saturated fat intake (g/day)	Nutrients	23348.00	0.04	0.01	0.00	0.00	0.58	0.08	0.55	12.00
Monounsaturated fat intake (g/day)	Nutrients	23348.00	0.06	0.01	0.00	0.51	0.15	0.08	0.55	2.00
Monosaccharides intake (g/day)	Nutrients	23348.00	-0.01	0.01	0.09	0.00	0.59	-	-	-
Sodium intake (mg/day)	Nutrients	23346.00	0.01	0.01	0.13	0.00	0.60	-	-	-
Vitamin B3 intake (mg/day)	Nutrients	23348.00	0.02	0.01	0.00	0.47	0.17	-	-	-
Cambridge physical activity index	Physical activity	24334.00	-0.02	0.01	0.00	0.00	0.78	-	-	-
Polyunsaturated fat intake (g/day)	Nutrients	23348.00	0.02	0.01	0.00	0.00	0.68	-	-	-
Average portion size of potatoes/rice/pasta based on photographic illustration of four sizes (smallest to largest)	Food	23348.00	-0.01	0.01	0.10	0.00	0.70	-	-	-
Total protein intake (g/day)	Nutrients	23348.00	-0.01	0.01	0.12	0.40	0.20	-	-	-
Animal based protein intake (g/day)	Nutrients	23348.00	0.01	0.01	0.35	0.00	0.57	-	-	-
Plant based protein intake (g/day)	Nutrients	23348.00	-0.04	0.01	0.00	0.56	0.13	0.08	0.55	6.00
Vitamin A intake (mg/day)	Nutrients	23348.00	0.02	0.01	0.04	0.00	0.63	-	-	-
Sucrose intake (g/day)	Nutrients	23348.00	-0.02	0.01	0.02	0.57	0.13	-	-	-
Cohabitation: Live alone vs Only one adult (spouse, partner)	Social	14595.00	0.02	0.02	0.33	0.00	0.33	-	-	-
Cohabitation: Live alone vs Only children	Social	4176.00	-0.08	0.04	0.03	0.00	0.87	-	-	-
Cohabitation: Live alone vs Adult and children	Social	12875.00	-0.02	0.02	0.31	0.00	0.70	-	-	-
Cohabitation: Live alone vs Other/others	Social	3368.00	0.08	0.06	0.19	0.00	0.84	-	-	-
Selenium intake (ug/day)	Nutrients	23348.00	0.01	0.01	0.05	0.73	0.06	-	-	-
Self rate of overall health	General health	18631.00	-0.01	0.01	0.12	0.00	0.96	-	-	-

For how much of the time during the last four weeks has your physical health or your emotiol problems limited your ability to interact with others?	General health	18495.00	-0.01	0.01	0.34	0.00	0.32	-	-	-
Get sick more often than other people	General health	18581.00	-0.01	0.01	0.13	0.00	0.32	-	-	-
As healthy as anyone	General health	18529.00	0.00	0.01	0.75	0.00	1.00	-	-	-
Worsen in health in the future	General health	18524.00	0.00	0.01	0.63	0.00	0.75	-	-	-
Excellent health	General health	18580.00	-0.01	0.01	0.36	0.00	0.39	-	-	-
Self rate of overall health compared to a year ago	General health	18453.00	-0.01	0.01	0.34	0.63	0.10	-	-	-
Physical limitation to participate in steneous activities: running, lifting heavy objects, taking part in physically demanding sports	General health	18590.00	0.01	0.01	0.38	0.00	0.54	-	-	-
Physical limitation to participate in moderately demanding activities: moving a table, vacuuming, walking in the forest or gardening	General health	18575.00	0.00	0.01	0.86	0.58	0.12	-	-	-
Physical limitation to participate in moderately demanding activities: lifting or carrying grocery bags	General health	18558.00	0.01	0.01	0.16	0.41	0.19	-	-	-
Physical limitation to participate in moderately demanding activities: walking up several stairs	General health	18570.00	0.00	0.01	0.50	0.00	0.45	-	-	-
Physical limitation to participate in moderately demanding activities: bending down or kneeling	General health	18549.00	-0.02	0.01	0.03	0.12	0.29	-	-	-
Physical limitation to participate in moderately demanding activities: walking more than two km	General health	18580.00	-0.01	0.01	0.10	0.00	0.36	-	-	-
Physical limitation that reduced the normal time spent at work or in other activities during the last four weeks	General health	18604.00	0.00	0.02	0.94	0.00	0.61	-	-	-
Physical limitation that made you do less than you wanted during the last four weeks	General health	18596.00	0.01	0.02	0.66	0.77	0.04	-	-	-
Physical limitation that made you not being able to perform certain work tasks or other activities during the last four weeks	General health	18523.00	-0.02	0.02	0.40	0.00	0.72	-	-	-
Physical limitation that limited your ability to perform certain work tasks or other activities during the last four weeks	General health	18545.00	-0.02	0.02	0.38	0.78	0.04	-	-	-
Emotiol problems that made you do less than you wanted during the last four weeks	General health	18614.00	-0.03	0.02	0.22	0.87	0.01	-	-	-
Extent to what your physical and emotiol health disrupted your usual social life during the last four weeks	General health	18609.00	-0.01	0.01	0.18	0.00	0.65	-	-	-
Pain during the last four weeks	General health	18594.00	0.00	0.01	0.87	0.00	0.59	-	-	-

How much has the pain during the last four weeks disturbed your normal work?	General health	18559.00	0.00	0.01	0.81	0.00	0.97	-	-	-
For how much of the time during the last four weeks have you felt really alert and strong?	General health	18558.00	0.00	0.01	0.89	0.00	0.55	-	-	-
For how much of the time during the last four weeks have you felt very nervous?	General health	18587.00	-0.01	0.01	0.42	0.00	0.74	-	-	-
For how much of the time during the last four weeks have you felt so depressed that nothing could cheer you up?	General health	18607.00	0.00	0.01	0.68	0.00	0.71	-	-	-
For how much of the time during the last four weeks have you felt calm and serene?	General health	18598.00	0.01	0.01	0.33	0.00	0.53	-	-	-
For how much of the time during the last four weeks have you felt full of energy?	General health	18575.00	0.00	0.01	0.66	0.00	0.80	-	-	-
For how much of the time during the last four weeks have you felt gloomy and sad?	General health	18580.00	0.00	0.01	0.85	0.00	0.55	-	-	-
For how much of the time during the last four weeks have you felt worn out?	General health	18552.00	0.01	0.01	0.21	0.00	0.94	-	-	-
For how much of the time during the last four weeks have you felt happy?	General health	18589.00	0.00	0.01	0.78	0.00	0.51	-	-	-
For how much of the time during the last four weeks have you felt tired?	General health	18608.00	-0.01	0.01	0.14	0.00	0.37	-	-	-
Long-term sickness	General health	25218.00	-0.01	0.02	0.53	0.00	0.86	-	-	-
Work shifts/weekends	Psychosocial	25239.00	-0.02	0.01	0.21	0.00	0.37	-	-	-
Risk of sleeping while sitting and reading	Sleep	16666.00	-0.01	0.01		0.00	0.35	-	-	-
Risk of sleeping while watching TV	Sleep	16848.00	0.00	0.01		0.00	0.35	-	-	-
Risk of sleeping while sitting ictive in a public place	Sleep	16745.00	-0.03	0.01		0.00	0.71	-	-	-
Risk of sleeping as a passenger in a car for one hour without break	Sleep	16669.00	-0.02	0.01		0.00	0.49	-	-	-
Risk of sleeping while lying down resting in the afternoon	Sleep	16793.00	0.00	0.01		0.00	0.84	-	-	-
Risk of sleeping while sitting still after having lunch	Sleep	16795.00	-0.01	0.01		0.00	0.66	-	-	-
Snore during sleep	Sleep	15681.00	0.03	0.01		0.00	0.38	-	-	-
Breath-holds during sleep	Sleep	11702.00	0.00	0.01		0.33	0.22	-	-	-
Number of cigarretes smoked per day (in groups)	Tobacco use	20994.00	0.01	0.01	0.05	0.00	0.60	-	-	-
Years smoking	Tobacco use	22278.00	0.00	0.01	0.71	0.00	0.46	-	-	-
Smoking status: Smokers vs non-smokers	Tobacco use	16199.00	0.05	0.02	0.01	0.00	0.99	-	-	-
Smoking status: Former smokers vs non-smokers	Tobacco use	18724.00	-0.01	0.02	0.56	0.48	0.16	-	-	-
Smoking status: Former occasiol smokers vs non-smokers	Tobacco use	15481.00	-0.01	0.02	0.60	0.28	0.24	-	-	-
Number of snuff boxes per week	Tobacco use	25221.00	-0.02	0.01	0.00	0.00	0.64	-	-	-
Snuff status: Snuff users vs non-snuff users	Tobacco use	21901.00	-0.02	0.02	0.22	0.00	0.62	-	-	-
Snuff status: Former snuff users vs non-snuff users	Tobacco use	20629.00	0.04	0.02	0.06	0.00	0.65	-	-	-
Years using snuff	Tobacco use	24516.00	0.00	0.01	0.65	0.00	0.47	-	-	-

Participation in associations or voluntary organisations	Social	25623.00	-0.01	0.01	0.52	0.70	0.07	-	-	-
Participation in sports or physical exercise associations	Social	11533.00	-0.01	0.02	0.56	0.47	0.17	-	-	-
Participation in study circles	Social	11533.00	0.00	0.03	0.88	0.00	0.57	-	-	-
Participation in other association	Social	11533.00	-0.01	0.02	0.59	0.70	0.07	-	-	-
People to ask for help apart from the ones at home	Social	25765.00	0.00	0.02	1.00	0.00	0.85	-	-	-
Number of friends that can come to your home at any time and feel at home	Social	25767.00	0.02	0.01	0.00	0.00	0.34	-	-	-
Number of social contacts with the same interests as you	Social	25839.00	0.02	0.01	0.00	0.38	0.21	-	-	-
Would you say that the number of people that you meet in your everyday life is enough or would you like to meet more or fewer people?	Social	25853.00	-0.02	0.01	0.00	0.40	0.20	-	-	-
Close relationship with anyone	Social	25792.00	0.00	0.01	0.72	0.00	0.60	-	-	-
Frequency of engaging in clubs, associations or study circles	Social	15487.00	-0.01	0.01	0.11	0.00	0.45	-	-	-
Number of social interactions during a normal week	Social	25861.00	0.00	0.01	0.93	0.00	0.91	-	-	-
Support from others	Social	25874.00	0.00	0.01	0.75	0.78	0.03	-	-	-
Number of people with whom you can speak openly	Social	25842.00	0.02	0.01	0.01	0.00	0.69	-	-	-
Receive hugs to comfort and support you	Social	25601.00	0.01	0.02	0.60	0.00	0.48	-	-	-
Stigmasterol intake (mg/day)	Nutrients	23348.00	-0.01	0.01	0.17	0.00	0.72	-	-	-
Tiamin intake (mg/day)	Nutrients	23348.00	0.00	0.01	0.61	0.22	0.26	-	-	-
Vitamin E intake (mg/day)	Nutrients	23348.00	0.02	0.01	0.00	0.00	0.77	-	-	-
Trans fat intake (g/day)	Nutrients	23348.00	0.08	0.01	0.00	0.71	0.06	0.08	0.55	4.00
Sum of phytosterols intake (mg/day)	Nutrients	23348.00	0.01	0.01	0.18	0.00	0.95	-	-	-
Zinc intake (mg/day)	Nutrients	23348.00	0.00	0.01	0.71	0.00	0.34	-	-	-

Supplementary Table 10. Linear mixed model association results for fasting glucose

Description	Group	N	Effect estimate	S.E.	p-value	I^2	Q p-value	marginal R2	conditional R2	R2 rank
Alcohol intake (g/day)	Alcohol	54578.00	0.02	0.00	0.00	0.00	0.50	0.08	0.29	15.00
Permanent employment	Psychosocial	50802.00	-0.03	0.01	0.00	0.73	0.05	-	-	-
Self-employed	Psychosocial	50802.00	0.00	0.01	0.78	0.00	0.67	-	-	-
Distance to work in kilometers (one way)	Physical activity	51564.00	-0.01	0.00	0.01	0.42	0.19	-	-	-
Last time a colleague visited you at home	Psychosocial	58018.00	-0.01	0.00	0.00	0.13	0.28	-	-	-
Job demands to work very fast	Psychosocial	58840.00	0.01	0.00	0.10	0.51	0.15	-	-	-
Frequency of social contacts with colleagues during leisure time	Psychosocial	57160.00	-0.01	0.00	0.00	0.00	0.62	-	-	-
High physical demand from job	Physical activity	58982.00	0.01	0.00	0.06	0.42	0.19	-	-	-
Enough time for job assignments	Psychosocial	58574.00	-0.01	0.00	0.03	0.00	0.81	-	-	-
Control over planning and execution of the workday	Psychosocial	58861.00	0.00	0.00	0.69	0.67	0.08	-	-	-
Ingenuity or creativity demand from job	Psychosocial	58556.00	0.00	0.00	0.80	0.00	0.33	-	-	-
Frequent social contacts with colleagues during work	Psychosocial	58203.00	-0.01	0.00	0.01	0.00	0.73	-	-	-
Contradictory demands in job	Psychosocial	58452.00	0.00	0.00	0.84	0.00	0.85	-	-	-
Possibility to leave your work for a while to speak with a colleague	Psychosocial	58250.00	0.00	0.00	0.48	0.54	0.14	-	-	-
Learn new things at job	Psychosocial	58704.00	0.00	0.00	0.11	0.55	0.13	-	-	-
High mental demand from job	Psychosocial	58384.00	-0.01	0.00	0.04	0.80	0.03	-	-	-
Repetitive job	Psychosocial	58739.00	0.00	0.00	0.35	0.00	0.89	-	-	-
Skill demand from job	Psychosocial	58667.00	0.00	0.00	0.75	0.61	0.11	-	-	-
Possibility to speak with colleagues during breaks	Psychosocial	58614.00	0.00	0.00	0.14	0.62	0.10	-	-	-
Control over own work assignment	Psychosocial	58839.00	0.01	0.00	0.10	0.22	0.26	-	-	-
Vitamin C intake (mg/day)	Nutrients	54578.00	0.00	0.00	0.15	0.00	0.80	-	-	-
Vitamin B12 intake (ug/day)	Nutrients	54578.00	0.01	0.00	0.02	0.00	0.51	-	-	-
Vitamin B2 intake (ug/day)	Nutrients	54578.00	0.00	0.00	0.12	0.00	0.92	-	-	-
Vitamin B6 intake (mg/day)	Nutrients	54578.00	0.00	0.00	0.35	0.69	0.07	-	-	-
Informed of having high blood pressure	General health	59721.00	0.05	0.01	0.00	0.32	0.23	0.06	0.25	35.00
Beta-sitostanol intake (mg/day)	Nutrients	54578.00	-0.01	0.00	0.00	0.00	0.57	0.08	0.29	23.00
Beta-sitosterol intake (mg/day)	Nutrients	54578.00	-0.02	0.00	0.00	0.54	0.14	0.09	0.29	8.00
Marital status: Single vs Married/partner	Social	54593.00	-0.01	0.01	0.31	0.68	0.08	-	-	-
Marital status: Single vs Divorced/separated	Social	9933.00	-0.01	0.02	0.45	0.00	0.38	-	-	-
Marital status: Single vs Widow/widower	Social	6238.00	0.01	0.03	0.77	0.00	0.53	-	-	-
Campestanol intake (mg/day)	Nutrients	54578.00	-0.01	0.00	0.00	0.00	0.91	0.08	0.29	22.00
Campesterol intake (mg/day)	Nutrients	54578.00	-0.01	0.00	0.01	0.78	0.03	-	-	-
Parents or siblings have diabetes	General health	58686.00	0.09	0.01	0.00	0.56	0.13	0.06	0.25	32.00
Disaccharides intake (g/day)	Nutrients	54578.00	0.00	0.00	0.53	0.00	0.35	-	-	-
Vitamin D intake (ug/day)	Nutrients	54578.00	0.01	0.00	0.00	0.00	0.94	-	-	-
Total energy intake (kcal/day)	Nutrients	54578.00	0.00	0.00	0.64	0.00	0.54	-	-	-
Formic acid intaje (g/day)	Nutrients	54578.00	0.01	0.00	0.04	0.00	0.73	-	-	-
Pentadecanoic acid intake (g/day)	Nutrients	54578.00	0.00	0.00	0.19	0.00	0.97	-	-	-
Palmitic acid intake (g/day)	Nutrients	54578.00	0.01	0.00	0.01	0.00	0.96	-	-	-

Heptadecanoic acid intake (g/day)	Nutrients	54578.00	0.00	0.00	0.19	0.00	0.97	-	-	-
Linoleic acid intake (g/day)	Nutrients	54578.00	0.00	0.00	0.21	0.26	0.24	-	-	-
Linolenic acid intake (g/day)	Nutrients	54578.00	0.00	0.00	0.82	0.74	0.05	-	-	-
Arachidonic acid (ARA) intake (g/day)	Nutrients	54576.00	0.01	0.00	0.05	0.53	0.14	-	-	-
Eicosapentaenoic acid (EPA) intake (g/day)	Nutrients	54577.00	0.00	0.00	0.18	0.89	0.00	-	-	-
Docosahexaenoic acid (DHA) intake (g/day)	Nutrients	54578.00	0.01	0.00	0.08	0.87	0.00	-	-	-
Fat intake (g/day)	Nutrients	54578.00	0.01	0.00	0.00	0.00	0.90	-	-	-
Fibre intake (g/day)	Nutrients	54578.00	-0.02	0.00	0.00	0.00	0.35	0.09	0.29	4.00
Folic acid intake (ug/day)	Nutrients	54578.00	-0.02	0.00	0.00	0.00	0.70	0.08	0.29	24.00
Phosphate intake (mg/day)	Nutrients	54578.00	-0.01	0.00	0.06	0.00	0.92	-	-	-
Whole grain intake (g/day)	Food	54578.00	-0.02	0.00	0.00	0.00	0.42	0.09	0.29	1.00
Travel to work: Walk to work vs passive travel to work	Physical activity	38609.00	-0.02	0.01	0.04	0.54	0.14	-	-	-
Travel to work: Cycle to work vs passive travel to work	Physical activity	44946.00	-0.01	0.01	0.34	0.00	0.57	-	-	-
Travel to work: Irregular travel mode to work vs passive travel to work	Physical activity	38587.00	0.01	0.01	0.49	0.00	0.33	-	-	-
Time spent in a week in moderately steneous activities	Physical activity	21640.00	-0.02	0.01		0.00	0.95	-	-	-
Sedentary or standing work	Physical activity	57934.00	0.01	0.01	0.26	0.00	0.51	-	-	-
Light but partly physically active work	Physical activity	57934.00	-0.02	0.01	0.01	0.00	0.40	-	-	-
Light and physically active work	Physical activity	57934.00	0.01	0.01	0.33	0.18	0.27	-	-	-
Sometimes physically straining work	Physical activity	57934.00	-0.01	0.01	0.39	0.00	0.36	-	-	-
Frequency of walking during leisure time	Physical activity	57561.00	-0.01	0.00	0.02	0.00	0.86	-	-	-
Frequency of cycling during leisure time	Physical activity	48825.00	0.00	0.00	0.18	0.00	0.94	-	-	-
Frequency of dancing during leisure time	Physical activity	33329.00	0.00	0.00	0.31	0.00	0.77	-	-	-
Frequency of shoveling snow during leisure time	Physical activity	35723.00	-0.01	0.00	0.06	0.00	0.89	-	-	-
Frequency of gardening during leisure time	Physical activity	35509.00	0.00	0.00	0.33	0.00	1.00	-	-	-
Frequency of hunting or fishing during leisure time	Physical activity	34791.00	-0.02	0.00	0.00	0.00	0.91	0.08	0.24	28.00
Frequency of picking berries or mushrooms during leisure time	Physical activity	35348.00	-0.02	0.00	0.00	0.00	0.60	0.09	0.24	7.00
Changed everyday exercise during the last year	Physical activity	37385.00	0.00	0.00	0.20	0.00	0.54	-	-	-
Everyday exercise satisfaction	Physical activity	37275.00	0.00	0.00	0.92	0.71	0.07	-	-	-
Exercise during the last three months	Physical activity	59079.00	-0.03	0.00	0.00	0.00	0.75	0.06	0.25	36.00
If you exercise, change in exercise habits during the last year	Physical activity	32968.00	-0.01	0.00	0.01	0.00	0.70	-	-	-
Amount of exercise during the last 12 months	Physical activity	22326.00	-0.03	0.01		0.00	0.49	-	-	-
Bregott on bread	Food	54578.00	0.01	0.00	0.07	0.00	0.36	-	-	-
Whole grain crisp bread	Food	54578.00	0.00	0.00	0.65	0.00	0.75	-	-	-
Whole grain soft bread	Food	54578.00	-0.02	0.00	0.00	0.00	0.75	0.08	0.29	16.00
White (soft) bread, thin crisp bread	Food	54578.00	0.01	0.00	0.01	0.00	0.69	-	-	-
Coffee rolls/buns, rusk	Food	54578.00	-0.01	0.00	0.00	0.00	0.79	0.08	0.29	27.00
Cheese 28%	Food	54578.00	0.00	0.00	0.89	0.00	0.64	-	-	-
Cheese 10-17%	Food	54578.00	-0.01	0.00	0.00	0.86	0.01	-	-	-
Soft cheese	Food	10679.00	0.00	0.01		0.75	0.05	-	-	-
Soft whey cheese	Food	10679.00	0.00	0.01		0.66	0.09	-	-	-

Sausage, liver pate on bread	Food	54578.00	0.01	0.00	0.06	0.00	0.80	-	-	-
Meat on bread	Food	54578.00	0.00	0.00	0.31	0.74	0.05	-	-	-
Butter on bread	Food	54578.00	0.00	0.00	0.17	0.64	0.09	-	-	-
Oatflake, whole wheat, rye or barley porridge	Food	54578.00	-0.02	0.00	0.00	0.45	0.18	0.08	0.29	14.00
Rosehip, sweet syrup soup	Food	54578.00	0.01	0.00	0.00	0.00	0.48	-	-	-
Sour milk, yoghurt (3% fat)	Food	54578.00	-0.01	0.00	0.09	0.00	0.81	-	-	-
Sour milk, yoghurt (low fat)	Food	54578.00	0.00	0.00	0.12	0.00	1.00	-	-	-
Fiber cereals	Food	54578.00	-0.02	0.00	0.00	0.00	0.99	0.08	0.29	9.00
Corn flakes	Food	54578.00	-0.01	0.00	0.08	0.00	0.35	-	-	-
Berries (fresh or frozen)	Food	54578.00	-0.01	0.00	0.07	0.00	0.39	-	-	-
Apple, pear, peach, orange, mandarin and grapefruit	Food	54578.00	-0.02	0.00	0.00	0.00	0.86	0.08	0.29	18.00
Ba	Food	54578.00	-0.01	0.00	0.00	0.88	0.00	-	-	-
Root vegetables and carrot	Food	54578.00	-0.01	0.00	0.00	0.00	0.79	-	-	-
Low fat margarine on bread	Food	54578.00	0.00	0.00	0.71	0.00	0.36	-	-	-
Tomato and cucumber	Food	54578.00	0.00	0.00	0.14	0.00	0.96	-	-	-
White cabbage, lettuce, lettuce cabbage, spich, borecole and broccoli	Food	54578.00	-0.01	0.00	0.00	0.00	0.37	-	-	-
Mixed frozen vegetables	Food	10679.00	0.00	0.01		0.13	0.28	-	-	-
Boiled or baked potato	Food	54578.00	0.00	0.00	0.18	0.00	0.34	-	-	-
Fried potatoes and pommes frites	Food	54578.00	0.01	0.00	0.00	0.00	0.73	-	-	-
Mashed potato	Food	10679.00	0.01	0.01		0.00	0.90	-	-	-
Potato salad	Food	10679.00	0.01	0.01		0.00	0.70	-	-	-
Rice	Food	54578.00	0.00	0.00	0.92	0.00	0.35	-	-	-
Pasta	Food	54578.00	0.00	0.00	0.54	0.00	0.42	-	-	-
Brown beans and pea soup	Food	54578.00	0.00	0.00	0.19	0.00	0.47	-	-	-
Margarine on bread	Food	54578.00	0.01	0.00	0.02	0.00	0.64	-	-	-
Blota (broth + bread)	Food	10679.00	0.01	0.01		0.56	0.13	-	-	-
Pancake, waffle and Swedish dumpling	Food	54578.00	0.00	0.00	0.55	0.87	0.00	-	-	-
Pizza	Food	54578.00	0.00	0.00	0.15	0.73	0.05	-	-	-
Minced meat dishes	Food	54578.00	0.01	0.00	0.03	0.00	0.64	-	-	-
Meat stew	Food	54578.00	0.00	0.00	0.61	0.00	0.60	-	-	-
Steak, chop, etc.	Food	54578.00	0.01	0.00	0.01	0.00	0.36	-	-	-
Bacon	Food	54578.00	0.00	0.00	0.23	0.88	0.00	-	-	-
Sausage as main dish	Food	54578.00	0.00	0.00	0.44	0.73	0.06	-	-	-
Hamburger	Food	54578.00	0.01	0.00	0.00	0.00	0.48	-	-	-
White meat (poultry)	Food	54578.00	0.00	0.00	0.14	0.00	0.41	-	-	-
Butter for cooking	Food	54578.00	0.00	0.00	0.48	0.00	0.75	-	-	-
Blood based food	Food	10679.00	0.01	0.01		0.77	0.04	-	-	-
Liver and kidney	Food	10679.00	0.01	0.01		0.00	0.81	-	-	-
Lean fish (e.g. perch, bass, cod)	Food	54578.00	0.00	0.00	0.18	0.00	0.57	-	-	-
Fatty fish (e.g. herring, whitefish, salmon)	Food	54578.00	0.01	0.00	0.11	0.82	0.02	-	-	-
Shellfish (e.g. shrimps, scallops)	Food	10679.00	0.00	0.01		0.00	0.58	-	-	-
Salty fish	Food	54578.00	0.00	0.00	0.33	0.82	0.02	-	-	-
Smoked fish/meat	Food	54578.00	0.00	0.00	0.38	0.00	0.81	-	-	-
Ice cream	Food	54578.00	-0.01	0.00	0.05	0.53	0.14	-	-	-
Sweets	Food	54578.00	0.00	0.00	0.16	0.00	0.57	-	-	-
Sugar, honey, marmelade and jam	Food	54578.00	0.00	0.00	0.17	0.83	0.02	-	-	-
Margarine for cooking	Food	54578.00	0.00	0.00	0.72	0.00	0.40	-	-	-
Cookies and pastry	Food	54578.00	-0.01	0.00	0.02	0.00	0.93	-	-	-
Chips, popcorn and salted nuts	Food	54578.00	0.01	0.00	0.01	0.00	0.33	-	-	-
Low fat milk (0.5%)	Beverage	54578.00	0.01	0.00	0.04	0.00	0.61	-	-	-
Milk, sour milk (1.5%)	Beverage	54578.00	0.00	0.00	0.85	0.00	0.99	-	-	-
Milk, sour milk (3%)	Beverage	54578.00	0.01	0.00	0.03	0.00	0.86	-	-	-
Sodas, soft drinks and juice	Beverage	54578.00	0.01	0.00	0.00	0.08	0.30	-	-	-
Brewed (filtered) coffee	Beverage	54578.00	0.00	0.00	0.99	0.00	0.35	-	-	-
Boiled coffee	Beverage	54578.00	-0.01	0.00	0.11	0.00	0.48	-	-	-
Tea	Beverage	54578.00	-0.01	0.00	0.00	0.49	0.16	-	-	-
Light beer	Alcohol	54578.00	0.00	0.00	0.13	0.00	0.88	-	-	-
Oil for cooking	Food	54578.00	0.00	0.00	0.34	0.25	0.25	-	-	-
Medium beer	Alcohol	54578.00	0.00	0.00	0.57	0.00	0.66	-	-	-

Strong beer	Alcohol	54578.00	0.01	0.00	0.06	0.00	0.50	-	-	-
Wine	Alcohol	54578.00	0.01	0.00	0.00	0.00	0.95	0.08	0.29	25.00
Liquor and spirits	Alcohol	54578.00	0.02	0.00	0.00	0.39	0.20	-	-	-
Salad dressing with oil	Food	54578.00	0.00	0.00	0.17	0.00	0.48	-	-	-
Cream, creme fraiche, sour cream	Food	54578.00	0.00	0.00	0.49	0.00	0.57	-	-	-
Average portion size of vegetables based on photographic illustration of four sizes (smallest to largest)	Food	54578.00	-0.02	0.00	0.00	0.27	0.24	0.06	0.25	42.00
Overall state of health during the last year	General health	59704.00	-0.02	0.00	0.00	0.19	0.27	0.06	0.25	38.00
Overall state of health compared to others your age	General health	15480.00						-	-	-
Parents or siblings had a cerebral hemorrhage/thrombosis or cardiac infarction before the age of 60	General health	58630.00	0.00	0.01	1.00	0.00	0.71	-	-	-
Teetotaler	Alcohol	37361.00	-0.06	0.01	0.00	0.00	0.81	0.08	0.25	21.00
Feel the need to reduce alcohol consumption	Alcohol	55002.00	0.01	0.01	0.33	0.00	0.42	-	-	-
Feel uneasy or guilty because of your way of drinking	Alcohol	33801.00	-0.03	0.01	0.00	0.00	0.96	-	-	-
Frequency of alcohol consumption	Alcohol	22442.00	0.02	0.01		0.00	0.84	-	-	-
Amount of alcohol drunk in a day	Alcohol	21382.00	0.03	0.01		0.00	0.42	-	-	-
Frequency of drinking six or more glasses at the same occasion	Alcohol	21795.00	0.03	0.01		0.00	0.67	-	-	-
Times during last year that you felt guilty because of your drinking	Alcohol	21760.00	0.01	0.01		0.00	0.59	-	-	-
Iron intake (mg/day)	Nutrients	54578.00	-0.01	0.00	0.11	0.59	0.12	-	-	-
Iodine intake (ug/day)	Nutrients	54578.00	0.00	0.00	0.77	0.00	0.55	-	-	-
Calcium intake (mg/day)	Nutrients	54578.00	0.00	0.00	0.32	0.02	0.31	-	-	-
Potassium intake (mg/day)	Nutrients	54578.00	-0.01	0.00	0.02	0.00	0.70	-	-	-
Beta-carotene intake (mg/day)	Nutrients	54578.00	-0.01	0.00	0.00	0.00	0.84	-	-	-
Cholesterol intake (g/day)	Nutrients	54578.00	0.01	0.00	0.00	0.06	0.30	-	-	-
Carbohydrates intake (g/day)	Nutrients	54578.00	-0.01	0.00	0.00	0.00	0.61	0.08	0.29	19.00
Average portion size of meat/fish based on photographic illustration of four sizes (smallest to largest)	Food	54578.00	0.00	0.00	0.45	0.00	0.58	-	-	-
Breakfast habits: Only coffee/tea for breakfast vs not breakfast at all	Food	3836.00	0.10	0.03	0.00	0.00	0.98	-	-	-
Breakfast habits: Coffee/tea and wheat buns or rusk for breakfast vs not breakfast at all	Food	3121.00	-0.11	0.03	0.00	0.00	0.94	-	-	-
Breakfast habits: Porridge w/o sandwich for breakfast vs not breakfast at all	Food	8358.00	-0.10	0.02	0.00	0.00	0.52	0.07	0.31	29.00
Breakfast habits: Gruel w/o sandwich for breakfast vs not breakfast at all	Food	2904.00	-0.13	0.03	0.00	0.00	0.55	-	-	-
Eat breakfast from 2000	Food	33295.00	-0.10	0.02	0.00	0.00	0.67	0.06	0.32	39.00
Enterodiol intake (ug/day)	Nutrients	54578.00	0.01	0.00	0.00	0.00	0.58	-	-	-
Enterolactone intake (ug/day)	Nutrients	54578.00	0.00	0.00	0.12	0.00	0.54	-	-	-
Equol intake (ug/day)	Nutrients	54578.00	0.00	0.00	0.47	0.00	0.70	-	-	-
Lariciresinol intake (ug/day)	Nutrients	54578.00	-0.02	0.00	0.00	0.00	0.94	0.09	0.29	2.00
Matairesinol intake (ug/day)	Nutrients	54578.00	-0.02	0.00	0.00	0.00	0.91	0.08	0.29	13.00

Medioresinol intake (ug/day)	Nutrients	54578.00	-0.01	0.00	0.00	0.00	0.99	-	-	-
Pinoresinol intake (ug/day)	Nutrients	54578.00	-0.02	0.00	0.00	0.00	0.87	0.09	0.29	5.00
Secoisolariciresinol intake (ug/day)	Nutrients	54578.00	-0.01	0.00	0.00	0.00	0.38	-	-	-
Sum of all ligns intake (ug/day)	Nutrients	54578.00	-0.02	0.00	0.00	0.00	0.97	0.08	0.29	11.00
Syringaresinol intake (ug/day)	Nutrients	54578.00	-0.01	0.00	0.00	0.00	0.98	0.08	0.29	20.00
Sum of Lariciresinol, Matairesinol, Pinoresinol, Secoisolariciresinol intake (ug/day)	Nutrients	54578.00	-0.02	0.00	0.00	0.00	0.94	0.09	0.29	3.00
Satisfaction with home and family situation	Psychosocial	47948.00	0.00	0.00	0.78	0.00	0.63	-	-	-
Appetite status	Psychosocial	47939.00	-0.02	0.00	0.00	0.00	0.82	0.05	0.27	44.00
Mood status	Psychosocial	47904.00	-0.01	0.00	0.01	0.43	0.19	-	-	-
Energy status	Psychosocial	47872.00	-0.01	0.00	0.00	0.00	0.94	-	-	-
Patience status	Psychosocial	47911.00	-0.01	0.00	0.00	0.17	0.27	-	-	-
Confidence status	Psychosocial	47885.00	0.00	0.00	0.70	0.00	0.97	-	-	-
Sleep status	Sleep	47981.00	-0.01	0.00	0.01	0.00	0.53	-	-	-
Do you feel important and appreciated outside your home?	Psychosocial	47973.00	0.00	0.00	0.41	0.00	0.86	-	-	-
Do you feel important and appreciated in your home?	Psychosocial	47617.00	0.00	0.00	0.18	0.00	0.59	-	-	-
Satisfaction with accomodation	Psychosocial	47990.00	0.00	0.00	0.85	0.00	0.93	-	-	-
Satisfaction with work situation	Psychosocial	47352.00	0.00	0.00	0.55	0.38	0.21	-	-	-
Satisfaction with economy	Psychosocial	47936.00	0.00	0.00	0.28	0.00	0.64	-	-	-
Satisfaction with leisure time	Psychosocial	47875.00	0.00	0.00	0.51	0.00	0.65	-	-	-
Hearing status	General health	47971.00	-0.01	0.00	0.15	0.20	0.26	-	-	-
Vision status	General health	47911.00	0.00	0.00	0.85	0.00	0.61	-	-	-
Memory status	Psychosocial	47841.00	0.00	0.00	0.70	0.00	0.83	-	-	-
Fitness status	Physical activity	47908.00	-0.03	0.00	0.00	0.00	0.89	0.05	0.27	43.00
Magnesium intake (mg/day)	Nutrients	54578.00	-0.02	0.00	0.00	0.00	0.44	0.08	0.30	10.00
Saturated fat intake (g/day)	Nutrients	54578.00	0.01	0.00	0.00	0.00	0.82	-	-	-
Monounsaturated fat intake (g/day)	Nutrients	54578.00	0.01	0.00	0.05	0.00	0.88	-	-	-
Monosaccharides intake (g/day)	Nutrients	54578.00	-0.02	0.00	0.00	0.00	0.53	0.08	0.29	17.00
Sodium intake (mg/day)	Nutrients	54576.00	0.00	0.00	0.97	0.81	0.02	-	-	-
Vitamin B3 intake (mg/day)	Nutrients	54578.00	0.00	0.00	0.54	0.80	0.03	-	-	-
Cambridge physical activity index	Physical activity	57209.00	-0.02	0.00	0.00	0.00	0.89	0.06	0.25	40.00
Polyunsaturated fat intake (g/day)	Nutrients	54578.00	0.00	0.00	0.47	0.15	0.28	-	-	-
Average portion size of potatoes/rice/pasta based on photographic illustration of four sizes (smallest to largest)	Food	54578.00	0.02	0.00	0.00	0.00	0.74	0.06	0.25	41.00
Total protein intake (g/day)	Nutrients	54578.00	0.00	0.00	0.50	0.00	0.36	-	-	-
Animal based protein intake (g/day)	Nutrients	54578.00	0.01	0.00	0.00	0.00	0.52	-	-	-
Plant based protein intake (g/day)	Nutrients	54578.00	-0.02	0.00	0.00	0.00	0.61	0.09	0.29	6.00
Vitamin A intake (mg/day)	Nutrients	54578.00	0.01	0.00	0.00	0.76	0.04	-	-	-
Sucrose intake (g/day)	Nutrients	54578.00	0.01	0.00	0.11	0.00	0.51	-	-	-
Cohabitation: Live alone vs Only one adult (spouse, partner)	Social	27805.00	-0.03	0.01	0.02	0.00	0.44	-	-	-
Cohabitation: Live alone vs Only children	Social	8998.00	0.00	0.02	0.99	0.00	0.65	-	-	-
Cohabitation: Live alone vs Adult and children	Social	34525.00	-0.03	0.01	0.00	0.18	0.27	-	-	-
Cohabitation: Live alone vs Other/others	Social	6858.00	-0.05	0.03	0.16	0.00	0.69	-	-	-

Selenium intake (ug/day)	Nutrients	54578.00	0.00	0.00	0.23	0.59	0.12	-	-	-
Self rate of overall health	General health	28291.00	-0.02	0.01	0.00	0.00	0.84	-	-	-
For how much of the time during the last four weeks has your physical health or your emotiol problems limited your ability to interact with others?	General health	28059.00	0.01	0.00	0.01	0.00	0.34	-	-	-
Get sick more often than other people	General health	28179.00	0.01	0.00	0.06	0.04	0.31	-	-	-
As healthy as anyone	General health	28095.00	-0.01	0.00	0.05	0.00	0.80	-	-	-
Worsen in health in the future	General health	28087.00	0.01	0.00	0.01	0.14	0.28	-	-	-
Excellent health	General health	28183.00	-0.02	0.00	0.00	0.00	0.72	-	-	-
Self rate of overall health compared to a year ago	General health	28056.00	-0.01	0.00	0.20	0.00	0.94	-	-	-
Physical limitation to participate in steneous activities: running, lifting heavy objects, taking part in physically demanding sports	General health	28238.00	0.01	0.01	0.04	0.00	0.57	-	-	-
Physical limitation to participate in moderately demanding activities: moving a table, vacuuming, walking in the forest or gardening	General health	28230.00	0.00	0.00	0.88	0.00	0.64	-	-	-
Physical limitation to participate in moderately demanding activities: lifting or carrying grocery bags	General health	28209.00	-0.01	0.01	0.21	0.00	0.90	-	-	-
Physical limitation to participate in moderately demanding activities: walking up several stairs	General health	28220.00	0.01	0.01	0.01	0.00	0.42	-	-	-
Physical limitation to participate in moderately demanding activities: bending down or kneeling	General health	28188.00	0.01	0.01	0.06	0.00	0.63	-	-	-
Physical limitation to participate in moderately demanding activities: walking more than two km	General health	28231.00	0.01	0.01	0.05	0.00	0.98	-	-	-
Physical limitation that reduced the normal time spent at work or in other activities during the last four weeks	General health	28255.00	-0.01	0.02	0.58	0.00	0.32	-	-	-
Physical limitation that made you do less than you wanted during the last four weeks	General health	28239.00	0.00	0.01	0.90	0.00	0.40	-	-	-
Physical limitation that made you not being able to perform certain work tasks or other activities during the last four weeks	General health	28128.00	-0.01	0.01	0.68	0.00	0.79	-	-	-
Physical limitation that limited your ability to perform certain work tasks or other activities during the last four weeks	General health	28164.00	0.01	0.01	0.45	0.00	0.86	-	-	-
Emotiol problems that made you do less than you wanted during the last four weeks	General health	28255.00	-0.01	0.01	0.68	0.70	0.07	-	-	-
Extent to what your physical and emotiol health disrupted your usual social life during the last four weeks	General health	28237.00	0.00	0.00	0.64	0.26	0.25	-	-	-

Pain during the last four weeks	General health	28201.00	0.00	0.00	0.92	0.20	0.26	-	-	-
How much has the pain during the last four weeks disturbed your normal work?	General health	28138.00	0.00	0.00	0.95	0.29	0.24	-	-	-
For how much of the time during the last four weeks have you felt really alert and strong?	General health	28151.00	-0.01	0.00	0.07	0.00	0.99	-	-	-
For how much of the time during the last four weeks have you felt very nervous?	General health	28197.00	0.02	0.00	0.00	0.74	0.05	-	-	-
For how much of the time during the last four weeks have you felt so depressed that nothing could cheer you up?	General health	28226.00	0.01	0.00	0.02	0.00	0.47	-	-	-
For how much of the time during the last four weeks have you felt calm and serene?	General health	28203.00	-0.01	0.00	0.04	0.11	0.29	-	-	-
For how much of the time during the last four weeks have you felt full of energy?	General health	28166.00	-0.01	0.00	0.06	0.24	0.25	-	-	-
For how much of the time during the last four weeks have you felt gloomy and sad?	General health	28174.00	0.00	0.00	0.37	0.00	0.43	-	-	-
For how much of the time during the last four weeks have you felt worn out?	General health	28131.00	0.01	0.00	0.01	0.00	0.98	-	-	-
For how much of the time during the last four weeks have you felt happy?	General health	28193.00	0.00	0.00	0.57	0.00	0.72	-	-	-
For how much of the time during the last four weeks have you felt tired?	General health	28219.00	0.01	0.00	0.01	0.00	0.79	-	-	-
Long-term sickness	General health	58058.00	0.02	0.01	0.05	0.71	0.06	-	-	-
Work shifts/weekends	Psychosocial	58163.00	-0.01	0.01	0.14	0.61	0.11	-	-	-
Risk of sleeping while sitting and reading	Sleep	21931.00	-0.01	0.01		0.00	0.74	-	-	-
Risk of sleeping while watching TV	Sleep	22165.00	0.00	0.01		0.00	0.67	-	-	-
Risk of sleeping while sitting ictive in a public place	Sleep	22020.00	0.00	0.01		0.00	0.81	-	-	-
Risk of sleeping as a passenger in a car for one hour without break	Sleep	21935.00	0.00	0.01		0.41	0.19	-	-	-
Risk of sleeping while lying down resting in the afternoon	Sleep	22090.00	0.00	0.01		0.00	0.34	-	-	-
Risk of sleeping while sitting still after having lunch	Sleep	22103.00	0.00	0.01		0.54	0.14	-	-	-
Snore during sleep	Sleep	20693.00	0.02	0.01		0.00	0.94	-	-	-
Breath-holds during sleep	Sleep	15459.00	0.03	0.01		0.00	0.68	-	-	-
Number of cigarretes smoked per day (in groups)	Tobacco use	49257.00	0.04	0.00	0.00	0.00	0.46	0.06	0.25	31.00
Years smoking	Tobacco use	52081.00	0.04	0.00	0.00	0.62	0.10	0.07	0.25	30.00
Smoking status: Smokers vs non-smokers	Tobacco use	38098.00	0.10	0.01	0.00	0.00	0.43	0.06	0.26	34.00
Smoking status: Former smokers vs non-smokers	Tobacco use	42728.00	0.04	0.01	0.00	0.00	0.84	0.06	0.26	33.00
Smoking status: Former occasiol smokers vs non-smokers	Tobacco use	35631.00	0.01	0.01	0.27	0.00	0.72	-	-	-
Number of snuff boxes per week	Tobacco use	57772.00	0.00	0.00	0.14	0.80	0.03	-	-	-

Snuff status: Snuff users vs non-snuff users	Tobacco use	50984.00	0.00	0.01	0.96	0.73	0.06	-	-	-
Snuff status: Former snuff users vs non-snuff users	Tobacco use	47611.00	-0.02	0.01	0.13	0.47	0.17	-	-	-
Years using snuff	Tobacco use	56260.00	-0.01	0.00	0.02	0.85	0.01	-	-	-
Participation in associations or voluntary organisations	Social	59362.00	-0.05	0.01	0.00	0.00	0.79	0.06	0.25	37.00
Participation in sports or physical exercise associations	Social	20787.00	-0.02	0.01	0.07	0.00	0.81	-	-	-
Participation in study circles	Social	20787.00	0.00	0.02	0.77	0.05	0.30	-	-	-
Participation in other association	Social	20787.00	0.03	0.01	0.00	0.00	0.65	-	-	-
People to ask for help apart from the ones at home	Social	59521.00	-0.02	0.01	0.07	0.35	0.22	-	-	-
Number of friends that can come to your home at any time and feel at home	Social	59497.00	0.00	0.00	0.95	0.00	0.37	-	-	-
Number of social contacts with the same interests as you	Social	59609.00	-0.01	0.00	0.01	0.00	0.90	-	-	-
Would you say that the number of people that you meet in your everyday life is enough or would you like to meet more or fewer people?	Social	59672.00	-0.01	0.00	0.02	0.22	0.26	-	-	-
Close relationship with anyone	Social	59630.00	-0.01	0.00	0.07	0.00	0.83	-	-	-
Frequency of engaging in clubs, associations or study circles	Social	37406.00	-0.01	0.00	0.02	0.46	0.17	-	-	-
Number of social interactions during a normal week	Social	59697.00	0.00	0.00	0.72	0.46	0.17	-	-	-
Support from others	Social	59720.00	-0.01	0.00	0.04	0.00	0.63	-	-	-
Number of people with whom you can speak openly	Social	59648.00	0.00	0.00	0.67	0.00	0.58	-	-	-
Receive hugs to comfort and support you	Social	59239.00	-0.02	0.01	0.03	0.00	0.92	-	-	-
Stigmasterol intake (mg/day)	Nutrients	54578.00	-0.01	0.00	0.00	0.00	0.66	0.08	0.29	26.00
Tiamin intake (mg/day)	Nutrients	54578.00	-0.01	0.00	0.00	0.60	0.12	-	-	-
Vitamin E intake (mg/day)	Nutrients	54578.00	-0.01	0.00	0.11	0.00	0.58	-	-	-
Trans fat intake (g/day)	Nutrients	54578.00	0.00	0.00	0.35	0.05	0.30	-	-	-
Sum of phytosterols intake (mg/day)	Nutrients	54578.00	-0.02	0.00	0.00	0.63	0.10	0.08	0.29	12.00
Zinc intake (mg/day)	Nutrients	54578.00	-0.01	0.00	0.00	0.00	0.95	-	-	-

Supplementary Table 11. Linear mixed model association results for 2h glucose

Description	Group	N	Effect estimate	S.E.	p-value	I^2	Q p-value	marginal R2	conditional R2	R2 rank
Alcohol intake (g/day)	Alcohol	52818.00	-0.04	0.01	0.00	0.00	0.39	0.09	0.42	13.00
Permanent employment	Psychosocial	49014.00	-0.04	0.01	0.00	0.00	0.98	-	-	-
Self-employed	Psychosocial	49014.00	-0.03	0.02	0.23	0.73	0.05	-	-	-
Distance to work in kilometers (one way)	Physical activity	49893.00	0.00	0.01	0.71	0.00	0.92	-	-	-
Last time a colleague visited you at home	Psychosocial	56077.00	-0.01	0.01	0.21	0.00	0.37	-	-	-
Job demands to work very fast	Psychosocial	56856.00	-0.02	0.01	0.00	0.00	0.51	-	-	-
Frequency of social contacts with colleagues during leisure time	Psychosocial	55250.00	-0.01	0.01	0.26	0.00	0.42	-	-	-
High physical demand from job	Physical activity	56988.00	-0.05	0.01	0.00	0.72	0.06	0.08	0.40	33.00
Enough time for job assignments	Psychosocial	56601.00	0.01	0.01	0.18	0.56	0.13	-	-	-
Control over planning and execution of the workday	Psychosocial	56880.00	0.01	0.01	0.25	0.00	0.36	-	-	-
Ingenuity or creativity demand from job	Psychosocial	56582.00	-0.03	0.01	0.00	0.00	0.43	0.08	0.40	34.00
Frequent social contacts with colleagues during work	Psychosocial	56253.00	0.00	0.01	0.80	0.20	0.26	-	-	-
Contradictory demands in job	Psychosocial	56495.00	-0.01	0.01	0.03	0.00	0.55	-	-	-
Possibility to leave your work for a while to speak with a colleague	Psychosocial	56302.00	0.01	0.01	0.02	0.48	0.17	-	-	-
Learn new things at job	Psychosocial	56733.00	-0.02	0.01	0.00	0.78	0.03	-	-	-
High mental demand from job	Psychosocial	56418.00	0.02	0.01	0.00	0.00	0.69	-	-	-
Repetitive job	Psychosocial	56762.00	0.01	0.01	0.05	0.39	0.20	-	-	-
Skill demand from job	Psychosocial	56691.00	-0.03	0.01	0.00	0.00	0.38	-	-	-
Possibility to speak with colleagues during breaks	Psychosocial	56643.00	0.00	0.01	0.58	0.30	0.23	-	-	-
Control over own work assignment	Psychosocial	56860.00	0.01	0.01	0.23	0.00	0.37	-	-	-
Vitamin C intake (mg/day)	Nutrients	52818.00	0.01	0.01	0.42	0.00	0.39	-	-	-
Vitamin B12 intake (ug/day)	Nutrients	52818.00	0.01	0.01	0.06	0.70	0.07	-	-	-
Vitamin B2 intake (ug/day)	Nutrients	52818.00	0.00	0.01	0.75	0.31	0.23	-	-	-
Vitamin B6 intake (mg/day)	Nutrients	52818.00	0.00	0.01	0.88	0.73	0.05	-	-	-
Informed of having high blood pressure	General health	57688.00	0.26	0.02	0.00	0.00	0.64	0.09	0.40	21.00
Beta-sitostanol intake (mg/day)	Nutrients	52818.00	0.00	0.01	0.65	0.00	0.84	-	-	-
Beta-sitosterol intake (mg/day)	Nutrients	52818.00	-0.01	0.01	0.05	0.00	0.51	-	-	-
Marital status: Single vs Married/partner	Social	52723.00	-0.03	0.02	0.19	0.00	0.42	-	-	-
Marital status: Single vs Divorced/separated	Social	9551.00	-0.09	0.03	0.01	0.00	0.82	-	-	-
Marital status: Single vs Widow/widower	Social	5956.00	-0.15	0.07	0.04	0.00	0.91	-	-	-
Campestanol intake (mg/day)	Nutrients	52818.00	0.01	0.01	0.41	0.00	0.89	-	-	-
Campesterol intake (mg/day)	Nutrients	52818.00	0.01	0.01	0.34	0.51	0.15	-	-	-
Parents or siblings have diabetes	General health	56713.00	0.21	0.02	0.00	0.00	0.62	0.08	0.40	24.00
Disaccharides intake (g/day)	Nutrients	52818.00	-0.02	0.01	0.02	0.00	0.99	-	-	-
Vitamin D intake (ug/day)	Nutrients	52818.00	0.01	0.01	0.23	0.00	0.43	-	-	-
Total energy intake (kcal/day)	Nutrients	52818.00	-0.04	0.01	0.00	0.00	0.38	0.09	0.42	19.00
Formic acid intaje (g/day)	Nutrients	52818.00	-0.01	0.01	0.07	0.00	0.48	-	-	-
Pentadecanoic acid intake (g/day)	Nutrients	52818.00	-0.02	0.01	0.01	0.00	0.52	-	-	-
Palmitic acid intake (g/day)	Nutrients	52818.00	-0.01	0.01	0.03	0.00	0.78	-	-	-

Heptadecanoic acid intake (g/day)	Nutrients	52818.00	-0.02	0.01	0.01	0.00	0.52	-	-	-
Linoleic acid intake (g/day)	Nutrients	52818.00	0.00	0.01	0.75	0.00	0.76	-	-	-
Linolenic acid intake (g/day)	Nutrients	52818.00	0.00	0.01	0.73	0.00	0.44	-	-	-
Arachidonic acid (ARA) intake (g/day)	Nutrients	52816.00	0.01	0.01	0.29	0.35	0.22	-	-	-
Eicosapentaenoic acid (EPA) intake (g/day)	Nutrients	52817.00	0.00	0.01	0.64	0.86	0.01	-	-	-
Docosahexaenoic acid (DHA) intake (g/day)	Nutrients	52818.00	0.00	0.01	0.71	0.85	0.01	-	-	-
Fat intake (g/day)	Nutrients	52818.00	0.00	0.01	0.92	0.00	0.78	-	-	-
Fibre intake (g/day)	Nutrients	52818.00	0.00	0.01	0.61	0.00	0.76	-	-	-
Folic acid intake (ug/day)	Nutrients	52818.00	-0.03	0.01	0.00	0.00	0.66	-	-	-
Phosphate intake (mg/day)	Nutrients	52818.00	-0.02	0.01	0.00	0.29	0.23	-	-	-
Whole grain intake (g/day)	Food	52818.00	0.00	0.01	0.65	0.00	0.58	-	-	-
Travel to work: Walk to work vs passive travel to work	Physical activity	37344.00	-0.03	0.02	0.13	0.00	0.73	-	-	-
Travel to work: Cycle to work vs passive travel to work	Physical activity	43486.00	-0.13	0.02	0.00	0.12	0.29	0.08	0.40	39.00
Travel to work: Irregular travel mode to work vs passive travel to work	Physical activity	37313.00	-0.05	0.02	0.04	0.22	0.26	-	-	-
Time spent in a week in moderately steneous activities	Physical activity	20906.00	-0.08	0.01		0.00	0.59	-	-	-
Sedentary or standing work	Physical activity	55985.00	0.07	0.01	0.00	0.00	0.36	0.08	0.40	35.00
Light but partly physically active work	Physical activity	55985.00	0.02	0.02	0.13	0.00	0.65	-	-	-
Light and physically active work	Physical activity	55985.00	-0.01	0.01	0.67	0.00	0.52	-	-	-
Sometimes physically straining work	Physical activity	55985.00	-0.07	0.01	0.00	0.07	0.30	0.08	0.40	36.00
Frequency of walking during leisure time	Physical activity	55622.00	-0.01	0.01	0.23	0.82	0.02	-	-	-
Frequency of cycling during leisure time	Physical activity	47191.00	-0.04	0.01	0.00	0.00	0.95	0.08	0.40	37.00
Frequency of dancing during leisure time	Physical activity	32211.00	-0.02	0.01	0.01	0.69	0.07	-	-	-
Frequency of shoveling snow during leisure time	Physical activity	34529.00	-0.04	0.01	0.00	0.00	0.73	0.10	0.39	5.00
Frequency of gardening during leisure time	Physical activity	34311.00	0.00	0.01	0.96	0.00	0.33	-	-	-
Frequency of hunting or fishing during leisure time	Physical activity	33626.00	-0.06	0.01	0.00	0.44	0.18	0.10	0.40	3.00
Frequency of picking berries or mushrooms during leisure time	Physical activity	34168.00	-0.03	0.01	0.00	0.00	0.70	0.10	0.40	4.00
Changed everyday exercise during the last year	Physical activity	36121.00	0.00	0.01	0.91	0.00	0.85	-	-	-
Everyday exercise satisfaction	Physical activity	36015.00	-0.02	0.01	0.00	0.51	0.15	-	-	-
Exercise during the last three months	Physical activity	57080.00	-0.07	0.01	0.00	0.00	0.87	0.08	0.40	27.00
If you exercise, change in exercise habits during the last year	Physical activity	31863.00	0.00	0.01	0.70	0.00	0.62	-	-	-
Amount of exercise during the last 12 months	Physical activity	21567.00	-0.10	0.01		0.00	0.44	-	-	-
Bregott on bread	Food	52818.00	-0.01	0.01	0.15	0.00	0.49	-	-	-
Whole grain crisp bread	Food	52818.00	0.00	0.01	0.87	0.00	0.45	-	-	-
Whole grain soft bread	Food	52818.00	0.00	0.01	0.96	0.00	0.68	-	-	-
White (soft) bread, thin crisp bread	Food	52818.00	0.02	0.01	0.01	0.00	0.44	-	-	-
Coffee rolls/buns, rusk	Food	52818.00	0.01	0.01	0.03	0.00	0.84	-	-	-
Cheese 28%	Food	52818.00	-0.02	0.01	0.01	0.00	0.86	-	-	-
Cheese 10-17%	Food	52818.00	0.00	0.01	0.96	0.00	0.89	-	-	-
Soft cheese	Food	10160.00	0.00	0.01		0.00	0.36	-	-	-
Soft whey cheese	Food	10160.00	-0.02	0.01		0.45	0.18	-	-	-

Sausage, liver pate on bread	Food	52818.00	0.00	0.01	0.95	0.20	0.26	-	-	-
Meat on bread	Food	52818.00	0.02	0.01	0.00	0.00	0.42	-	-	-
Butter on bread	Food	52818.00	0.00	0.01	0.97	0.00	0.38	-	-	-
Oatflake, whole wheat, rye or barley porridge	Food	52818.00	0.00	0.01	0.85	0.54	0.14	-	-	-
Rosehip, sweet syrup soup	Food	52818.00	0.03	0.01	0.00	0.00	0.77	0.09	0.42	16.00
Sour milk, yoghurt (3% fat)	Food	52818.00	-0.01	0.01	0.21	0.00	0.96	-	-	-
Sour milk, yoghurt (low fat)	Food	52818.00	0.00	0.01	0.77	0.00	0.51	-	-	-
Fiber cereals	Food	52818.00	-0.01	0.01	0.20	0.00	0.63	-	-	-
Corn flakes	Food	52818.00	0.01	0.01	0.12	0.00	0.47	-	-	-
Berries (fresh or frozen)	Food	52818.00	-0.01	0.01	0.02	0.00	0.86	-	-	-
Apple, pear, peach, orange, mandarin and grapefruit	Food	52818.00	-0.03	0.01	0.00	0.00	0.84	0.09	0.41	11.00
Ba	Food	52818.00	0.00	0.01	0.59	0.74	0.05	-	-	-
Root vegetables and carrot	Food	52818.00	-0.02	0.01	0.00	0.02	0.31	-	-	-
Low fat margarine on bread	Food	52818.00	0.01	0.01	0.41	0.00	0.94	-	-	-
Tomato and cucumber	Food	52818.00	-0.01	0.01	0.12	0.00	0.88	-	-	-
White cabbage, lettuce, lettuce cabbage, spich, borecole and broccoli	Food	52818.00	-0.02	0.01	0.00	0.00	0.91	-	-	-
Mixed frozen vegetables	Food	10160.00	0.00	0.01		0.21	0.26	-	-	-
Boiled or baked potato	Food	52818.00	0.01	0.01	0.02	0.00	0.76	-	-	-
Fried potatoes and pommes frites	Food	52818.00	0.00	0.01	0.62	0.00	0.49	-	-	-
Mashed potato	Food	10160.00	0.01	0.01		0.20	0.26	-	-	-
Potato salad	Food	10160.00	0.03	0.01		0.00	0.67	-	-	-
Rice	Food	52818.00	0.01	0.01	0.13	0.00	0.84	-	-	-
Pasta	Food	52818.00	-0.01	0.01	0.19	0.00	0.71	-	-	-
Brown beans and pea soup	Food	52818.00	-0.01	0.01	0.16	0.55	0.14	-	-	-
Margarine on bread	Food	52818.00	0.01	0.01	0.16	0.00	0.67	-	-	-
Blota (broth + bread)	Food	10160.00	0.00	0.01		0.57	0.13	-	-	-
Pancake, waffle and Swedish dumpling	Food	52818.00	-0.01	0.01	0.12	0.00	0.93	-	-	-
Pizza	Food	52818.00	0.02	0.01	0.00	0.00	0.99	-	-	-
Minced meat dishes	Food	52818.00	0.00	0.01	0.63	0.00	0.47	-	-	-
Meat stew	Food	52818.00	-0.01	0.01	0.02	0.59	0.12	-	-	-
Steak, chop, etc.	Food	52818.00	0.00	0.01	0.60	0.00	0.83	-	-	-
Bacon	Food	52818.00	-0.02	0.01	0.00	0.00	0.54	-	-	-
Sausage as main dish	Food	52818.00	0.00	0.01	0.80	0.68	0.08	-	-	-
Hamburger	Food	52818.00	0.03	0.01	0.00	0.00	0.38	-	-	-
White meat (poultry)	Food	52818.00	0.00	0.01	0.62	0.54	0.14	-	-	-
Butter for cooking	Food	52818.00	0.00	0.01	0.85	0.00	0.79	-	-	-
Blood based food	Food	10160.00	0.01	0.01		0.00	0.36	-	-	-
Liver and kidney	Food	10160.00	0.02	0.01		0.00	0.76	-	-	-
Lean fish (e.g. perch, bass, cod)	Food	52818.00	0.01	0.01	0.04	0.53	0.14	-	-	-
Fatty fish (e.g. herring, whitefish, salmon)	Food	52818.00	-0.01	0.01	0.20	0.75	0.05	-	-	-
Shellfish (e.g. shrimps, scallops)	Food	10160.00	-0.02	0.01		0.00	0.34	-	-	-
Salty fish	Food	52818.00	0.01	0.01	0.38	0.73	0.05	-	-	-
Smoked fish/meat	Food	52818.00	0.00	0.01	0.93	0.00	0.79	-	-	-
Ice cream	Food	52818.00	-0.01	0.01	0.09	0.00	0.75	-	-	-
Sweets	Food	52818.00	-0.01	0.01	0.30	0.00	0.94	-	-	-
Sugar, honey, marmelade and jam	Food	52818.00	-0.01	0.01	0.13	0.62	0.10	-	-	-
Margarine for cooking	Food	52818.00	0.02	0.01	0.01	0.06	0.30	-	-	-
Cookies and pastry	Food	52818.00	0.00	0.01	0.54	0.00	0.45	-	-	-
Chips, popcorn and salted nuts	Food	52818.00	-0.02	0.01	0.02	0.55	0.14	-	-	-
Low fat milk (0.5%)	Beverage	52818.00	0.01	0.01	0.05	0.00	0.45	-	-	-
Milk, sour milk (1.5%)	Beverage	52818.00	0.00	0.01	0.99	0.00	0.88	-	-	-
Milk, sour milk (3%)	Beverage	52818.00	-0.01	0.01	0.03	0.00	0.95	-	-	-
Sodas, soft drinks and juice	Beverage	52818.00	0.01	0.01	0.39	0.00	0.43	-	-	-
Brewed (filtered) coffee	Beverage	52818.00	-0.07	0.01	0.00	0.00	0.54	0.09	0.42	7.00
Boiled coffee	Beverage	52818.00	-0.04	0.01	0.00	0.00	0.90	0.09	0.42	9.00
Tea	Beverage	52818.00	0.03	0.01	0.00	0.00	0.91	0.09	0.42	17.00
Light beer	Alcohol	52818.00	0.00	0.01	0.78	0.88	0.00	-	-	-
Oil for cooking	Food	52818.00	0.00	0.01	0.84	0.00	0.76	-	-	-
Medium beer	Alcohol	52818.00	-0.02	0.01	0.00	0.38	0.20	-	-	-

Strong beer	Alcohol	52818.00	-0.04	0.01	0.00	0.00	0.94	0.09	0.42	10.00
Wine	Alcohol	52818.00	-0.02	0.01	0.00	0.00	0.50	-	-	-
Liquor and spirits	Alcohol	52818.00	-0.02	0.01	0.00	0.27	0.24	-	-	-
Salad dressing with oil	Food	52818.00	-0.01	0.01	0.03	0.00	0.91	-	-	-
Cream, creme fraiche, sour cream	Food	52818.00	-0.01	0.01	0.05	0.00	0.97	-	-	-
Average portion size of vegetables based on photographic illustration of four sizes (smallest to largest)	Food	52818.00	-0.05	0.01	0.00	0.00	0.92	0.08	0.40	32.00
Overall state of health during the last year	General health	57671.00	-0.04	0.01	0.00	0.00	0.56	0.08	0.40	30.00
Overall state of health compared to others your age	General health	30329.00	-0.06	0.01	0.00	0.40	0.20	0.11	0.43	1.00
Parents or siblings had a cerebral hemorrhage/thrombosis or cardiac infarction before the age of 60	General health	56644.00	0.05	0.02	0.00	0.00	0.86	-	-	-
Teetotaler	Alcohol	36096.00	0.13	0.02	0.00	0.00	0.81	0.10	0.39	2.00
Feel the need to reduce alcohol consumption	Alcohol	53153.00	-0.07	0.02	0.00	0.00	0.48	-	-	-
Feel uneasy or guilty because of your way of drinking	Alcohol	32682.00	-0.06	0.02	0.01	0.00	0.71	-	-	-
Frequency of alcohol consumption	Alcohol	21675.00	-0.05	0.01		0.03	0.31	-	-	-
Amount of alcohol drunk in a day	Alcohol	20668.00	-0.03	0.01		0.00	0.89	-	-	-
Frequency of drinking six or more glasses at the same occasion	Alcohol	21052.00	-0.02	0.01		0.58	0.12	-	-	-
Times during last year that you felt guilty because of your drinking	Alcohol	21021.00	-0.02	0.01		0.00	0.52	-	-	-
Iron intake (mg/day)	Nutrients	52818.00	0.03	0.01	0.00	0.00	0.37	-	-	-
Iodine intake (ug/day)	Nutrients	52818.00	-0.01	0.01	0.30	0.62	0.11	-	-	-
Calcium intake (mg/day)	Nutrients	52818.00	-0.01	0.01	0.33	0.00	0.47	-	-	-
Potassium intake (mg/day)	Nutrients	52818.00	-0.03	0.01	0.00	0.60	0.11	-	-	-
Beta-carotene intake (mg/day)	Nutrients	52818.00	-0.02	0.01	0.00	0.00	0.37	-	-	-
Cholesterol intake (g/day)	Nutrients	52818.00	-0.01	0.01	0.33	0.00	0.39	-	-	-
Carbohydrates intake (g/day)	Nutrients	52818.00	0.01	0.01	0.23	0.00	0.84	-	-	-
Average portion size of meat/fish based on photographic illustration of four sizes (smallest to largest)	Food	52818.00	-0.06	0.01	0.00	0.00	0.38	0.08	0.40	31.00
Breakfast habits: Only coffee/tea for breakfast vs not breakfast at all	Food	3673.00	-0.09	0.06	0.17	0.40	0.20	-	-	-
Breakfast habits: Coffee/tea and wheat buns or rusk for breakfast vs not breakfast at all	Food	3003.00	0.08	0.06	0.16	0.00	0.69	-	-	-
Breakfast habits: Porridge w/o sandwich for breakfast vs not breakfast at all	Food	8040.00	0.12	0.04	0.01	0.87	0.01	-	-	-
Breakfast habits: Gruel w/o sandwich for breakfast vs not breakfast at all	Food	2781.00	0.15	0.07	0.04	0.00	0.46	-	-	-
Eat breakfast from 2000	Food	32249.00	0.16	0.03	0.00	0.00	0.61	0.07	0.48	43.00
Enterodiol intake (ug/day)	Nutrients	52818.00	0.00	0.01	0.74	0.00	0.55	-	-	-
Enterolactone intake (ug/day)	Nutrients	52818.00	0.00	0.01	0.61	0.00	0.63	-	-	-
Equol intake (ug/day)	Nutrients	52818.00	0.01	0.01	0.18	0.00	0.32	-	-	-
Lariciresinol intake (ug/day)	Nutrients	52818.00	-0.03	0.01	0.00	0.00	0.99	0.09	0.41	12.00
Matairesinol intake (ug/day)	Nutrients	52818.00	0.00	0.01	0.71	0.00	0.94	-	-	-

Medioresinol intake (ug/day)	Nutrients	52818.00	0.00	0.01	0.51	0.00	0.80	-	-	-
Pinoresinol intake (ug/day)	Nutrients	52818.00	-0.01	0.01	0.18	0.00	0.82	-	-	-
Secoisolariciresinol intake (ug/day)	Nutrients	52818.00	-0.08	0.01	0.00	0.00	0.72	0.09	0.42	6.00
Sum of all ligns intake (ug/day)	Nutrients	52818.00	-0.01	0.01	0.35	0.00	0.87	-	-	-
Syringaresinol intake (ug/day)	Nutrients	52818.00	0.00	0.01	0.61	0.00	0.89	-	-	-
Sum of Lariciresinol, Matairesinol, Pinoresinol, Secoisolariciresinol intake (ug/day)	Nutrients	52818.00	-0.03	0.01	0.00	0.00	0.89	0.09	0.41	14.00
Satisfaction with home and family situation	Psychosocial	46496.00	0.02	0.01	0.02	0.00	0.57	-	-	-
Appetite status	Psychosocial	46487.00	-0.02	0.01	0.02	0.00	0.81	-	-	-
Mood status	Psychosocial	46449.00	-0.01	0.01	0.44	0.00	0.32	-	-	-
Energy status	Psychosocial	46417.00	-0.03	0.01	0.00	0.00	0.96	0.07	0.42	41.00
Patience status	Psychosocial	46459.00	0.00	0.01	0.79	0.00	0.47	-	-	-
Confidence status	Psychosocial	46436.00	-0.01	0.01	0.25	0.00	0.54	-	-	-
Sleep status	Sleep	46527.00	-0.01	0.01	0.11	0.00	0.70	-	-	-
Do you feel important and appreciated outside your home?	Psychosocial	46524.00	0.01	0.01	0.32	0.00	0.63	-	-	-
Do you feel important and appreciated in your home?	Psychosocial	46180.00	0.01	0.01	0.04	0.00	0.86	-	-	-
Satisfaction with accomodation	Psychosocial	46533.00	0.02	0.01	0.01	0.00	0.41	-	-	-
Satisfaction with work situation	Psychosocial	45929.00	0.01	0.01	0.04	0.00	0.93	-	-	-
Satisfaction with economy	Psychosocial	46487.00	0.03	0.01	0.00	0.37	0.21	-	-	-
Satisfaction with leisure time	Psychosocial	46426.00	0.00	0.01	0.53	0.00	0.79	-	-	-
Hearing status	General health	46517.00	0.02	0.01	0.03	0.00	0.45	-	-	-
Vision status	General health	46458.00	0.03	0.01	0.00	0.00	0.41	-	-	-
Memory status	Psychosocial	46389.00	0.01	0.01	0.04	0.47	0.17	-	-	-
Fitness status	Physical activity	46456.00	-0.08	0.01	0.00	0.00	0.38	0.07	0.41	40.00
Magnesium intake (mg/day)	Nutrients	52818.00	-0.04	0.01	0.00	0.42	0.19	0.09	0.41	8.00
Saturated fat intake (g/day)	Nutrients	52818.00	-0.01	0.01	0.26	0.00	0.72	-	-	-
Monounsaturated fat intake (g/day)	Nutrients	52818.00	-0.01	0.01	0.18	0.00	0.81	-	-	-
Monosaccharides intake (g/day)	Nutrients	52818.00	-0.03	0.01	0.00	0.00	0.69	0.09	0.41	15.00
Sodium intake (mg/day)	Nutrients	52816.00	0.00	0.01	0.61	0.81	0.02	-	-	-
Vitamin B3 intake (mg/day)	Nutrients	52818.00	-0.01	0.01	0.06	0.00	0.60	-	-	-
Cambridge physical activity index	Physical activity	55293.00	-0.08	0.01	0.00	0.00	0.82	0.08	0.40	26.00
Polyunsaturated fat intake (g/day)	Nutrients	52818.00	0.01	0.01	0.38	0.00	0.74	-	-	-
Average portion size of potatoes/rice/pasta based on photographic illustration of four sizes (smallest to largest)	Food	52818.00	-0.02	0.01	0.02	0.00	0.49	-	-	-
Total protein intake (g/day)	Nutrients	52818.00	0.00	0.01	0.60	0.71	0.06	-	-	-
Animal based protein intake (g/day)	Nutrients	52818.00	0.00	0.01	0.70	0.50	0.16	-	-	-
Plant based protein intake (g/day)	Nutrients	52818.00	0.01	0.01	0.03	0.00	0.53	-	-	-
Vitamin A intake (mg/day)	Nutrients	52818.00	0.01	0.01	0.10	0.00	0.44	-	-	-
Sucrose intake (g/day)	Nutrients	52818.00	0.00	0.01	0.88	0.00	0.48	-	-	-
Cohabitation: Live alone vs Only one adult (spouse, partner)	Social	26740.00	0.01	0.02	0.70	0.00	0.77	-	-	-
Cohabitation: Live alone vs Only children	Social	8659.00	0.00	0.04	1.00	0.00	0.36	-	-	-
Cohabitation: Live alone vs Adult and children	Social	33396.00	-0.04	0.02	0.09	0.00	0.41	-	-	-
Cohabitation: Live alone vs Other/others	Social	6564.00	0.01	0.07	0.85	0.00	0.32	-	-	-

Selenium intake (ug/day)	Nutrients	52818.00	0.00	0.01	0.53	0.72	0.06	-	-	-
Self rate of overall health	General health	27385.00	-0.07	0.01	0.00	0.00	0.83	-	-	-
For how much of the time during the last four weeks has your physical health or your emotiol problems limited your ability to interact with others?	General health	27171.00	0.02	0.01	0.09	0.20	0.26	-	-	-
Get sick more often than other people	General health	27283.00	0.06	0.01	0.00	0.00	0.75	-	-	-
As healthy as anyone	General health	27202.00	-0.05	0.01	0.00	0.00	0.66	-	-	-
Worsen in health in the future	General health	27192.00	0.03	0.01	0.01	0.00	0.90	-	-	-
Excellent health	General health	27285.00	-0.07	0.01	0.00	0.00	0.71	-	-	-
Self rate of overall health compared to a year ago	General health	27160.00	-0.03	0.01	0.01	0.00	0.50	-	-	-
Physical limitation to participate in steneous activities: running, lifting heavy objects, taking part in physically demanding sports	General health	27330.00	0.06	0.01	0.00	0.00	0.64	-	-	-
Physical limitation to participate in moderately demanding activities: moving a table, vacuuming, walking in the forest or gardening	General health	27322.00	0.05	0.01	0.00	0.74	0.05	-	-	-
Physical limitation to participate in moderately demanding activities: lifting or carrying grocery bags	General health	27302.00	0.05	0.01	0.00	0.00	0.81	-	-	-
Physical limitation to participate in moderately demanding activities: walking up several stairs	General health	27313.00	0.04	0.01	0.00	0.62	0.11	-	-	-
Physical limitation to participate in moderately demanding activities: bending down or kneeling	General health	27283.00	0.04	0.01	0.00	0.00	0.44	-	-	-
Physical limitation to participate in moderately demanding activities: walking more than two km	General health	27329.00	0.03	0.01	0.00	0.00	0.95	-	-	-
Physical limitation that reduced the normal time spent at work or in other activities during the last four weeks	General health	27349.00	0.06	0.03	0.06	0.00	0.96	-	-	-
Physical limitation that made you do less than you wanted during the last four weeks	General health	27332.00	0.04	0.02	0.07	0.00	0.68	-	-	-
Physical limitation that made you not being able to perform certain work tasks or other activities during the last four weeks	General health	27227.00	0.07	0.03	0.01	0.00	0.88	-	-	-
Physical limitation that limited your ability to perform certain work tasks or other activities during the last four weeks	General health	27262.00	0.08	0.03	0.01	0.00	0.40	-	-	-
Emotiol problems that made you do less than you wanted during the last four weeks	General health	27350.00	-0.02	0.03	0.45	0.82	0.02	-	-	-
Extent to what your physical and emotiol health disrupted your usual social life during the last four weeks	General health	27337.00	0.01	0.01	0.29	0.04	0.31	-	-	-

Pain during the last four weeks	General health	27301.00	0.02	0.01	0.08	0.00	0.41	-	-	-
How much has the pain during the last four weeks disturbed your normal work?	General health	27241.00	0.02	0.01	0.08	0.00	0.75	-	-	-
For how much of the time during the last four weeks have you felt really alert and strong?	General health	27257.00	-0.03	0.01	0.00	0.00	0.40	-	-	-
For how much of the time during the last four weeks have you felt very nervous?	General health	27301.00	0.00	0.01	0.93	0.00	0.41	-	-	-
For how much of the time during the last four weeks have you felt so depressed that nothing could cheer you up?	General health	27327.00	0.00	0.01	0.83	0.00	0.51	-	-	-
For how much of the time during the last four weeks have you felt calm and serene?	General health	27304.00	-0.01	0.01	0.29	0.00	0.96	-	-	-
For how much of the time during the last four weeks have you felt full of energy?	General health	27269.00	-0.03	0.01	0.01	0.00	0.70	-	-	-
For how much of the time during the last four weeks have you felt gloomy and sad?	General health	27276.00	-0.01	0.01	0.50	0.00	0.84	-	-	-
For how much of the time during the last four weeks have you felt worn out?	General health	27236.00	0.00	0.01	0.81	0.00	0.56	-	-	-
For how much of the time during the last four weeks have you felt happy?	General health	27296.00	0.00	0.01	0.95	0.00	0.38	-	-	-
For how much of the time during the last four weeks have you felt tired?	General health	27319.00	0.01	0.01	0.19	0.00	0.53	-	-	-
Long-term sickness	General health	56100.00	0.05	0.02	0.01	0.00	0.36	-	-	-
Work shifts/weekends	Psychosocial	56190.00	-0.06	0.01	0.00	0.00	0.68	-	-	-
Risk of sleeping while sitting and reading	Sleep	21187.00	-0.02	0.01		0.22	0.26	-	-	-
Risk of sleeping while watching TV	Sleep	21413.00	-0.02	0.01		0.00	0.92	-	-	-
Risk of sleeping while sitting ictive in a public place	Sleep	21276.00	-0.03	0.01		0.18	0.27	-	-	-
Risk of sleeping as a passenger in a car for one hour without break	Sleep	21194.00	-0.02	0.01		0.00	0.63	-	-	-
Risk of sleeping while lying down resting in the afternoon	Sleep	21347.00	0.01	0.01		0.00	0.79	-	-	-
Risk of sleeping while sitting still after having lunch	Sleep	21359.00	0.00	0.01		0.00	0.79	-	-	-
Snore during sleep	Sleep	19996.00	0.03	0.01		0.67	0.08	-	-	-
Breath-holds during sleep	Sleep	14947.00	0.02	0.01		0.00	0.72	-	-	-
Number of cigarretes smoked per day (in groups)	Tobacco use	47588.00	-0.08	0.01	0.00	0.00	0.50	0.09	0.40	20.00
Years smoking	Tobacco use	50309.00	-0.10	0.01	0.00	0.00	0.39	0.09	0.40	23.00
Smoking status: Smokers vs non-smokers	Tobacco use	36797.00	-0.32	0.02	0.00	0.00	0.92	0.09	0.41	18.00
Smoking status: Former smokers vs non-smokers	Tobacco use	41331.00	-0.12	0.02	0.00	0.00	0.45	0.09	0.41	22.00
Smoking status: Former occasiol smokers vs non-smokers	Tobacco use	34514.00	-0.07	0.02	0.00	0.81	0.02	-	-	-
Number of snuff boxes per week	Tobacco use	55796.00	-0.05	0.01	0.00	0.00	0.79	0.08	0.40	29.00

Snuff status: Snuff users vs non-snuff users	Tobacco use	49204.00	-0.18	0.02	0.00	0.00	0.50	0.08	0.40	25.00
Snuff status: Former snuff users vs non-snuff users	Tobacco use	45991.00	-0.12	0.02	0.00	0.22	0.26	0.08	0.40	38.00
Years using snuff	Tobacco use	54339.00	-0.06	0.01	0.00	0.00	0.52	0.08	0.40	28.00
Participation in associations or voluntary organisations	Social	57354.00	-0.07	0.01	0.00	0.70	0.07	-	-	-
Participation in sports or physical exercise associations	Social	20173.00	-0.11	0.02	0.00	0.10	0.29	0.07	0.48	42.00
Participation in study circles	Social	20173.00	0.09	0.03	0.00	0.00	0.81	-	-	-
Participation in other association	Social	20173.00	0.05	0.02	0.01	0.30	0.23	-	-	-
People to ask for help apart from the ones at home	Social	57506.00	-0.03	0.02	0.13	0.00	0.73	-	-	-
Number of friends that can come to your home at any time and feel at home	Social	57473.00	-0.01	0.01	0.24	0.79	0.03	-	-	-
Number of social contacts with the same interests as you	Social	57588.00	-0.03	0.01	0.00	0.00	0.40	-	-	-
Would you say that the number of people that you meet in your everyday life is enough or would you like to meet more or fewer people?	Social	57647.00	-0.01	0.01	0.19	0.72	0.06	-	-	-
Close relationship with anyone	Social	57614.00	0.00	0.01	0.48	0.54	0.14	-	-	-
Frequency of engaging in clubs, associations or study circles	Social	36188.00	-0.02	0.01	0.02	0.14	0.28	-	-	-
Number of social interactions during a normal week	Social	57671.00	-0.02	0.01	0.00	0.00	0.61	-	-	-
Support from others	Social	57690.00	0.00	0.01	0.92	0.00	0.84	-	-	-
Number of people with whom you can speak openly	Social	57620.00	0.00	0.01	0.93	0.52	0.15	-	-	-
Receive hugs to comfort and support you	Social	57245.00	0.00	0.02	0.97	0.07	0.30	-	-	-
Stigmasterol intake (mg/day)	Nutrients	52818.00	-0.02	0.01	0.01	0.00	0.91	-	-	-
Tiamin intake (mg/day)	Nutrients	52818.00	-0.02	0.01	0.00	0.00	0.50	-	-	-
Vitamin E intake (mg/day)	Nutrients	52818.00	0.00	0.01	0.90	0.00	0.94	-	-	-
Trans fat intake (g/day)	Nutrients	52818.00	-0.01	0.01	0.14	0.11	0.29	-	-	-
Sum of phytosterols intake (mg/day)	Nutrients	52818.00	-0.01	0.01	0.27	0.00	0.39	-	-	-
Zinc intake (mg/day)	Nutrients	52818.00	0.00	0.01	0.87	0.65	0.09	-	-	-

Supplementary Table 12. Longitudinal association results for BMI

Description	Group	N	Effect estimate	S.E.	p-value	I^2	Q p-value	adjusted R2	R2 rank
Alcohol intake (g/day)	Alcohol	25801.00	-0.03	0.01	0.08	0.00	0.69	-	-
Permanent employment	Psychosocial	28753.00	-0.03	0.03	0.37	0.66	0.09	-	-
Self-employed	Psychosocial	28495.00	0.00	0.05	0.97	0.00	0.73	-	-
Distance to work in kilometers (one way)	Physical activity	25665.00	0.01	0.01	0.62	0.03	0.31	-	-
Last time a colleague visited you at home	Psychosocial	27744.00	0.00	0.01	0.89	0.00	0.75	-	-
Job demands to work very fast	Psychosocial	28956.00	0.02	0.01	0.22	0.00	0.73	-	-
Frequency of social contacts with colleagues during leisure time	Psychosocial	27475.00	0.01	0.01	0.32	0.00	0.52	-	-
High physical demand from job	Physical activity	29023.00	0.06	0.01	0.00	0.00	0.39	0.70	31.00
Enough time for job assignments	Psychosocial	28837.00	0.01	0.01	0.54	0.00	0.65	-	-
Control over planning and execution of the workday	Psychosocial	28977.00	-0.04	0.01	0.00	0.00	0.53	-	-
Ingenuity or creativity demand from job	Psychosocial	28861.00	-0.04	0.01	0.00	0.00	0.90	-	-
Frequent social contacts with colleagues during work	Psychosocial	27872.00	-0.02	0.01	0.06	0.01	0.31	-	-
Contradictory demands in job	Psychosocial	28736.00	-0.01	0.01	0.41	0.00	0.92	-	-
Possibility to leave your work for a while to speak with a colleague	Psychosocial	27901.00	0.00	0.01	0.73	0.00	0.48	-	-
Learn new things at job	Psychosocial	28922.00	-0.03	0.01	0.02	0.15	0.28	-	-
High mental demand from job	Psychosocial	28780.00	0.00	0.01	0.97	0.00	0.93	-	-
Repetitive job	Psychosocial	28941.00	0.07	0.01	0.00	0.18	0.27	0.70	22.00
Skill demand from job	Psychosocial	28884.00	-0.02	0.01	0.19	0.00	0.72	-	-
Possibility to speak with colleagues during breaks	Psychosocial	28063.00	-0.02	0.01	0.18	0.00	0.66	-	-
Control over own work assignment	Psychosocial	28949.00	-0.03	0.01	0.02	0.00	0.86	-	-
Vitamin C intake (mg/day)	Nutrients	25801.00	0.05	0.01	0.00	0.11	0.29	-	-
Vitamin B12 intake (ug/day)	Nutrients	25801.00	0.05	0.01	0.00	0.00	0.86	-	-
Vitamin B2 intake (ug/day)	Nutrients	25801.00	0.01	0.01	0.54	0.00	0.60	-	-
Vitamin B6 intake (mg/day)	Nutrients	25801.00	0.04	0.01	0.02	0.00	0.72	-	-
Informed of having high blood pressure	General health	29167.00	0.17	0.04	0.00	0.00	0.98	0.70	30.00
Beta-sitostanol intake (mg/day)	Nutrients	25801.00	-0.03	0.01	0.06	0.00	0.65	-	-
Beta-sitosterol intake (mg/day)	Nutrients	25801.00	0.01	0.01	0.31	0.00	0.67	-	-
Marital status: Single vs Married/partner	Social	27269.00	-0.20	0.04	0.00	0.00	0.57	0.70	17.00
Marital status: Single vs Divorced/separated	Social	4680.00	0.21	0.08	0.01	0.00	0.35	-	-
Marital status: Single vs Widow/widower	Social	3074.00	0.28	0.21	0.18	0.00	0.97	-	-
Campestanol intake (mg/day)	Nutrients	25801.00	-0.03	0.01	0.03	0.00	0.84	-	-
Campesterol intake (mg/day)	Nutrients	25801.00	-0.01	0.01	0.66	0.00	0.44	-	-
Parents or siblings have diabetes	General health	28865.00	0.10	0.04	0.00	0.00	0.73	-	-
Disaccharides intake (g/day)	Nutrients	25801.00	0.03	0.01	0.03	0.00	0.77	-	-
Vitamin D intake (ug/day)	Nutrients	25801.00	0.03	0.01	0.02	0.00	0.77	-	-
Total energy intake (kcal/day)	Nutrients	25801.00	-0.05	0.02	0.00	0.27	0.24	-	-
Formic acid intaje (g/day)	Nutrients	25801.00	-0.04	0.01	0.00	0.00	0.87	-	-
Pentadecanoic acid intake (g/day)	Nutrients	25801.00	-0.05	0.01	0.00	0.00	0.99	-	-
Palmitic acid intake (g/day)	Nutrients	25801.00	0.00	0.01	0.94	0.00	0.89	-	-
Heptadecanoic acid intake (g/day)	Nutrients	25801.00	-0.05	0.01	0.00	0.00	0.99	-	-
Linoleic acid intake (g/day)	Nutrients	25801.00	0.03	0.01	0.04	0.54	0.14	-	-
Linolenic acid intake (g/day)	Nutrients	25801.00	0.02	0.01	0.12	0.00	0.36	-	-
Arachidonic acid (ARA) intake (g/day)	Nutrients	25801.00	0.06	0.01	0.00	0.00	0.86	0.70	16.00
Eicosapentaenoic acid (EPA) intake (g/day)	Nutrients	25801.00	0.02	0.01	0.17	0.00	0.92	-	-
Docosahexaenoic acid (DHA) intake (g/day)	Nutrients	25801.00	0.02	0.01	0.14	0.00	0.97	-	-
Fat intake (g/day)	Nutrients	25801.00	0.00	0.01	0.82	0.00	0.70	-	-
Fibre intake (g/day)	Nutrients	25801.00	-0.01	0.01	0.32	0.00	0.97	-	-
Folic acid intake (ug/day)	Nutrients	25801.00	0.03	0.01	0.02	0.00	0.99	-	-
Phosphate intake (mg/day)	Nutrients	25801.00	0.01	0.01	0.52	0.00	0.66	-	-
Whole grain intake (g/day)	Food	25801.00	-0.05	0.01	0.00	0.65	0.09	-	-
Travel to work: Walk to work vs passive travel to work	Physical activity	18640.00	0.04	0.05	0.46	0.00	0.78	-	-
Travel to work: Cycle to work vs passive travel to work	Physical activity	22566.00	-0.14	0.03	0.00	0.00	0.62	0.70	18.00
Travel to work: Irregular travel mode to work vs passive travel to work	Physical activity	19103.00	-0.06	0.05	0.21	0.00	0.38	-	-
Sedentary or standing work	Physical activity	28488.00	0.03	0.03	0.29	0.00	0.83	-	-
Light but partly physically active work	Physical activity	28488.00	-0.02	0.03	0.49	0.00	0.80	-	-
Light and physically active work	Physical activity	28488.00	-0.05	0.03	0.14	0.00	0.90	-	-
Sometimes physically straining work	Physical activity	28488.00	0.03	0.03	0.26	0.00	0.76	-	-
Frequency of walking during leisure time	Physical activity	28200.00	-0.03	0.01	0.06	0.00	0.49	-	-
Frequency of cycling during leisure time	Physical activity	24719.00	-0.04	0.01	0.00	0.00	0.73	-	-
Frequency of dancing during leisure time	Physical activity	25367.00	0.02	0.01	0.13	0.80	0.03	-	-
Frequency of shoveling snow during leisure time	Physical activity	27592.00	-0.03	0.01	0.03	0.00	0.57	-	-
Frequency of gardening during leisure time	Physical activity	27380.00	-0.02	0.01	0.08	0.00	0.38	-	-
Frequency of hunting or fishing during leisure time	Physical activity	26664.00	0.02	0.01	0.16	0.00	0.49	-	-
Frequency of picking berries or mushrooms during leisure time	Physical activity	27240.00	0.00	0.01	0.94	0.16	0.28	-	-
Changed everyday exercise during the last year	Physical activity	29073.00	0.07	0.01	0.00	0.00	0.83	0.70	28.00
Everyday exercise satisfaction	Physical activity	28988.00	0.03	0.01	0.01	0.25	0.25	-	-
Exercise during the last three months	Physical activity	28968.00	-0.06	0.01	0.00	0.40	0.20	-	-
If you exercise, change in exercise habits during the last year	Physical activity	24774.00	0.08	0.01	0.00	0.00	0.94	0.70	35.00
Bregott on bread	Food	25801.00	-0.06	0.01	0.00	0.00	0.36	-	-
Whole grain crisp bread	Food	25801.00	0.00	0.01	0.80	0.00	0.55	-	-
Whole grain soft bread	Food	25801.00	-0.05	0.01	0.00	0.00	0.72	-	-
White (soft) bread, thin crisp bread	Food	25801.00	0.00	0.01	0.94	0.00	0.96	-	-
Coffee rolls/buns, rusk	Food	25801.00	-0.01	0.01	0.29	0.87	0.01	-	-
Cheese 28%	Food	25801.00	-0.07	0.01	0.00	0.49	0.16	-	-
Cheese 10-17%	Food	25801.00	0.03	0.01	0.06	0.01	0.32	-	-
Soft cheese	Food	12978.00	0.04	0.02	0.06	0.00	0.97	-	-
Soft whey cheese	Food	12978.00	-0.03	0.02	0.12	0.00	0.65	-	-
Sausage, liver pate on bread	Food	25801.00	0.00	0.01	0.79	0.00	0.73	-	-
Meat on bread	Food	25801.00	0.02	0.01	0.14	0.00	0.68	-	-

Butter on bread	Food	25801.00	0.02	0.01	0.11	0.25	0.25	-	-
Oatflake, whole wheat, rye or barley porridge	Food	25801.00	-0.01	0.01	0.55	0.00	0.85	-	-
Rosehip, sweet syrup soup	Food	25801.00	0.01	0.01	0.29	0.00	0.93	-	-
Sour milk, yoghurt (3% fat)	Food	25801.00	-0.09	0.01	0.00	0.25	0.25	0.70	8.00
Sour milk, yoghurt (low fat)	Food	25801.00	0.05	0.01	0.00	0.07	0.30	-	-
Fiber cereals	Food	25801.00	-0.09	0.01	0.00	0.00	0.86	0.70	7.00
Corn flakes	Food	25801.00	-0.02	0.01	0.17	0.59	0.12	-	-
Berries (fresh or frozen)	Food	25801.00	-0.02	0.01	0.13	0.00	0.46	-	-
Apple, pear, peach, orange, mandarin and grapefruit	Food	25801.00	0.01	0.02	0.67	0.00	0.52	-	-
Ba	Food	25801.00	-0.01	0.01	0.59	0.00	0.62	-	-
Root vegetables and carrot	Food	25801.00	0.05	0.01	0.00	0.00	0.64	-	-
Low fat margarine on bread	Food	25801.00	0.04	0.01	0.01	0.00	0.34	-	-
Tomato and cucumber	Food	25801.00	0.07	0.01	0.00	0.00	0.52	0.70	14.00
White cabbage, lettuce, lettuce cabbage, spich, borecole and broccoli	Food	25801.00	0.04	0.01	0.01	0.00	0.76	-	-
Mixed frozen vegetables	Food	12978.00	0.05	0.02	0.01	0.00	0.69	-	-
Boiled or baked potato	Food	25801.00	-0.01	0.01	0.34	0.00	0.70	-	-
Fried potatoes and pommes frites	Food	25801.00	0.06	0.01	0.00	0.00	0.93	0.70	15.00
Mashed potato	Food	12978.00	0.07	0.02	0.00	0.00	0.61	-	-
Potato salad	Food	10590.00	0.06	0.02	0.01	0.00	0.66	-	-
Rice	Food	25801.00	0.02	0.01	0.15	0.00	0.34	-	-
Pasta	Food	25801.00	0.02	0.01	0.20	0.00	0.37	-	-
Brown beans and pea soup	Food	25801.00	0.02	0.01	0.09	0.00	0.85	-	-
Margarine on bread	Food	25801.00	0.00	0.01	0.95	0.31	0.23	-	-
Blota (broth + bread)	Food	12978.00	0.02	0.02	0.19	0.58	0.12	-	-
Pancake, waffle and Swedish dumpling	Food	25801.00	0.00	0.01	0.95	0.00	0.74	-	-
Pizza	Food	25801.00	0.01	0.01	0.72	0.00	0.77	-	-
Minced meat dishes	Food	25801.00	0.04	0.01	0.00	0.00	0.35	-	-
Meat stew	Food	25801.00	0.02	0.01	0.11	0.00	0.97	-	-
Steak, chop, etc.	Food	25801.00	0.03	0.01	0.05	0.00	0.83	-	-
Bacon	Food	25801.00	0.05	0.01	0.00	0.00	0.88	-	-
Sausage as main dish	Food	25801.00	0.04	0.01	0.00	0.00	0.97	-	-
Hamburger	Food	25801.00	0.05	0.01	0.00	0.00	0.43	-	-
White meat (poultry)	Food	25801.00	0.03	0.01	0.05	0.00	0.46	-	-
Butter for cooking	Food	25801.00	0.04	0.01	0.01	0.77	0.04	-	-
Blood based food	Food	12978.00	0.04	0.02	0.05	0.79	0.03	-	-
Liver and kidney	Food	12978.00	0.04	0.02	0.03	0.18	0.27	-	-
Lean fish (e.g. perch, bass, cod)	Food	25801.00	0.02	0.01	0.15	0.00	0.51	-	-
Fatty fish (e.g. herring, whitefish, salmon)	Food	25801.00	0.01	0.01	0.60	0.00	0.86	-	-
Shellfish (e.g. shrimps, scallops)	Food	12978.00	0.05	0.02	0.01	0.72	0.06	-	-
Salty fish	Food	25801.00	0.02	0.01	0.27	0.00	0.60	-	-
Smoked fish/meat	Food	25801.00	0.04	0.01	0.01	0.00	0.69	-	-
Ice cream	Food	25801.00	0.01	0.01	0.64	0.43	0.19	-	-
Sweets (chocolate and candy)	Food	25801.00	0.03	0.01	0.07	0.00	0.66	-	-
Sugar, honey, marmelade and jam	Food	25801.00	-0.01	0.01	0.38	0.00	0.37	-	-
Margarine for cooking	Food	25801.00	0.00	0.01	0.80	0.00	0.62	-	-
Cookies and pastry	Food	25801.00	-0.05	0.01	0.00	0.00	0.74	-	-
Chips, popcorn and salted nuts	Food	25801.00	0.06	0.01	0.00	0.00	0.49	-	-
Low fat milk (0.5%)	Beverage	25801.00	0.05	0.01	0.00	0.00	0.76	-	-
Milk, sour milk (1.5%)	Beverage	25801.00	-0.03	0.01	0.06	0.00	0.75	-	-
Milk, sour milk (3%)	Beverage	25801.00	0.04	0.01	0.01	0.00	0.54	-	-
Sodas, soft drinks and juice	Beverage	25801.00	0.07	0.01	0.00	0.67	0.08	-	-
Brewed (filtered) coffee	Beverage	25801.00	0.01	0.01	0.32	0.00	0.95	-	-
Boiled coffee	Beverage	25801.00	0.02	0.01	0.18	0.00	0.71	-	-
Tea	Beverage	25801.00	-0.04	0.01	0.00	0.00	0.97	-	-
Light beer	Alcohol	25801.00	-0.05	0.01	0.00	0.00	0.78	-	-
Oil for cooking	Food	25801.00	0.03	0.01	0.02	0.52	0.15	-	-
Medium beer	Alcohol	25801.00	0.00	0.01	0.79	0.00	0.68	-	-
Strong beer	Alcohol	25801.00	0.02	0.01	0.21	0.00	0.54	-	-
Wine	Alcohol	25801.00	-0.05	0.01	0.00	0.00	0.51	-	-
Liquor and spirits	Alcohol	25801.00	0.03	0.01	0.04	0.00	0.51	-	-
Salad dressing with oil	Food	25801.00	-0.01	0.01	0.57	0.00	0.76	-	-
Cream, creme fraiche, sour cream	Food	25801.00	-0.04	0.01	0.01	0.26	0.24	-	-
Average portion size of vegetables based on photographic illustration of four sizes (smallest to largest)	Food	25801.00	-0.03	0.01	0.03	0.00	0.48	-	-
Overall state of health during the last year	General health	29035.00	-0.10	0.01	0.00	0.00	0.81	0.70	33.00
Overall state of health compared to others your age	General health	25276.00	-0.03	0.01	0.03	0.63	0.10	-	-
Parents or siblings had a cerebral hemorrhage/thrombosis or cardiac infarction before the age of 60	General health	28814.00	0.12	0.03	0.00	0.40	0.20	-	-
Teetotaler	Alcohol	28677.00	0.03	0.04	0.45	0.00	0.32	-	-
Feel the need to reduce alcohol consumption	Alcohol	25924.00	0.13	0.05	0.00	0.00	0.44	-	-
Feel uneasy or guilty because of your way of drinking	Alcohol	25904.00	0.13	0.04	0.00	0.00	0.99	-	-
Iron intake (mg/day)	Nutrients	25801.00	0.01	0.02	0.63	0.14	0.28	-	-
Iodine intake (ug/day)	Nutrients	25801.00	0.04	0.01	0.00	0.00	0.48	-	-
Calcium intake (mg/day)	Nutrients	25801.00	0.00	0.01	0.80	0.00	0.86	-	-
Potassium intake (mg/day)	Nutrients	25801.00	0.04	0.02	0.01	0.00	0.53	-	-
Beta-carotene intake (mg/day)	Nutrients	25801.00	0.06	0.01	0.00	0.00	0.62	-	-
Cholesterol intake (g/day)	Nutrients	25801.00	0.00	0.01	0.98	0.00	0.78	-	-
Carbohydrates intake (g/day)	Nutrients	25801.00	-0.01	0.01	0.31	0.00	0.88	-	-
Average portion size of meat/fish based on photographic illustration of four sizes (smallest to largest)	Food	25801.00	0.05	0.02	0.00	0.00	0.74	-	-
Breakfast habits: Only coffee/tea for breakfast vs not breakfast at all	Food	1590.00	-0.11	0.20	0.58	0.00	0.62	-	-
Breakfast habits: Coffee/tea and wheat buns or rusk for breakfast vs not breakfast at all	Food	1987.00	-0.18	0.12	0.16	0.00	0.49	-	-

Breakfast habits: Porridge w/o sandwich for breakfast vs not breakfast at all	Food	3444.00	-0.38	0.09	0.00	0.31	0.23	-	-
Breakfast habits: Gruel w/o sandwich for breakfast vs not breakfast at all	Food	1874.00	-0.48	0.14	0.00	0.00	0.85	-	-
Eat breakfast from 2000	Food	2297.00	-0.37	0.15	0.02	0.00	0.84	-	-
Enterodiol intake (ug/day)	Nutrients	25801.00	0.07	0.01	0.00	0.00	0.88	0.70	11.00
Enterolactone intake (ug/day)	Nutrients	25801.00	0.01	0.01	0.52	0.00	0.67	-	-
Equol intake (ug/day)	Nutrients	25801.00	0.07	0.01	0.00	0.00	0.79	0.70	10.00
Lariciresinol intake (ug/day)	Nutrients	25801.00	-0.05	0.01	0.00	0.00	0.63	-	-
Matairesinol intake (ug/day)	Nutrients	25801.00	-0.07	0.01	0.00	0.00	0.87	0.70	12.00
Medioresinol intake (ug/day)	Nutrients	25801.00	-0.03	0.01	0.02	0.00	0.99	-	-
Pinoresinol intake (ug/day)	Nutrients	25801.00	-0.05	0.01	0.00	0.00	0.67	-	-
Secoisolariciresinol intake (ug/day)	Nutrients	25801.00	-0.03	0.01	0.06	0.00	0.35	-	-
Sum of all ligns intake (ug/day)	Nutrients	25801.00	-0.05	0.01	0.00	0.00	0.83	-	-
Syringaresinol intake (ug/day)	Nutrients	25801.00	-0.05	0.01	0.00	0.00	0.92	-	-
Sum of Lariciresinol, Matairesinol, Pinoresinol, Secoisolariciresinol intake (ug/day)	Nutrients	25801.00	-0.05	0.01	0.00	0.00	0.61	-	-
Satisfaction with home and family situation	Psychosocial	13713.00	-0.08	0.02	0.00	0.00	0.77	0.70	23.00
Appetite status	Psychosocial	13703.00	-0.09	0.02	0.00	0.00	0.71	0.70	26.00
Mood status	Psychosocial	13702.00	-0.05	0.02	0.01	0.00	0.83	-	-
Energy status	Psychosocial	13690.00	-0.05	0.02	0.01	0.00	0.85	-	-
Patience status	Psychosocial	13705.00	-0.05	0.02	0.01	0.00	0.72	-	-
Confidence status	Psychosocial	13699.00	-0.03	0.02	0.15	0.00	0.80	-	-
Sleep status	Sleep	13728.00	-0.08	0.02	0.00	0.00	0.91	0.70	27.00
Do you feel important and appreciated outside your home?	Psychosocial	13718.00	-0.03	0.02	0.08	0.00	0.92	-	-
Do you feel important and appreciated in your home?	Psychosocial	13613.00	-0.06	0.02	0.00	0.00	0.74	-	-
Satisfaction with accomodation	Psychosocial	13721.00	-0.08	0.02	0.00	0.00	0.53	0.70	24.00
Satisfaction with work situation	Psychosocial	13579.00	-0.07	0.02	0.00	0.34	0.22	-	-
Satisfaction with economy	Psychosocial	13704.00	-0.11	0.02	0.00	0.00	0.51	0.70	20.00
Satisfaction with leisure time	Psychosocial	13692.00	-0.02	0.02	0.34	0.36	0.21	-	-
Hearing status	General health	13718.00	-0.05	0.02	0.01	0.00	0.45	-	-
Vision status	General health	13704.00	-0.01	0.02	0.64	0.00	0.34	-	-
Memory status	Psychosocial	13681.00	-0.04	0.02	0.05	0.00	0.51	-	-
Fitness status	Physical activity	13696.00	-0.10	0.02	0.00	0.00	0.52	0.70	29.00
Magnesium intake (mg/day)	Nutrients	25801.00	0.00	0.01	0.84	0.00	0.73	-	-
Saturated fat intake (g/day)	Nutrients	25801.00	-0.02	0.01	0.15	0.00	0.89	-	-
Monounsaturated fat intake (g/day)	Nutrients	25801.00	0.04	0.02	0.02	0.00	0.81	-	-
Monosaccharides intake (g/day)	Nutrients	25801.00	0.03	0.01	0.04	0.00	0.77	-	-
Sodium intake (mg/day)	Nutrients	25801.00	0.06	0.01	0.00	0.26	0.25	-	-
Vitamin B3 intake (mg/day)	Nutrients	25801.00	0.05	0.01	0.00	0.00	0.40	-	-
Cambridge physical activity index	Physical activity	28399.00	-0.03	0.01	0.02	0.00	0.36	-	-
Polyunsaturated fat intake (g/day)	Nutrients	25801.00	0.03	0.01	0.03	0.43	0.18	-	-
Average portion size of potatoes/rice/pasta based on photographic illustration of four sizes (smallest to largest)	Food	25801.00	-0.01	0.02	0.34	0.00	0.32	-	-
Total protein intake (g/day)	Nutrients	25801.00	0.04	0.01	0.01	0.00	0.63	-	-
Animal based protein intake (g/day)	Nutrients	25801.00	0.04	0.01	0.00	0.00	0.67	-	-
Plant based protein intake (g/day)	Nutrients	25801.00	-0.03	0.01	0.07	0.00	0.96	-	-
Vitamin A intake (mg/day)	Nutrients	25801.00	0.00	0.02	0.94	0.00	0.86	-	-
Sucrose intake (g/day)	Nutrients	25801.00	0.03	0.01	0.08	0.00	0.65	-	-
Cohabitation: Live alone vs Only one adult (spouse, partner)	Social	9277.00	-0.36	0.06	0.00	0.11	0.29	0.70	34.00
Cohabitation: Live alone vs Only children	Social	3874.00	-0.18	0.09	0.06	0.00	0.67	-	-
Cohabitation: Live alone vs Adult and children	Social	20262.00	-0.39	0.05	0.00	0.00	0.90	0.70	21.00
Cohabitation: Live alone vs Other/others	Social	2986.00	-0.33	0.14	0.02	0.00	0.40	-	-
Selenium intake (ug/day)	Nutrients	25801.00	0.05	0.01	0.00	0.00	0.84	-	-
Self rate of overall health	General health	352.00	-0.38	0.14	0.01	0.63	0.10	-	-
For how much of the time during the last four weeks has your physical health or your emotiol problems limited your ability to interact with others?	General health	350.00	0.22	0.14	0.13	0.00	0.70	-	-
Get sick more often than other people	General health	351.00	0.11	0.14	0.45	0.00	0.66	-	-
As healthy as anyone	General health	352.00	-0.32	0.14	0.02	0.79	0.03	-	-
Worsen in health in the future	General health	348.00	0.37	0.14	0.01	0.00	0.75	-	-
Excellent health	General health	351.00	-0.44	0.14	0.00	0.00	0.33	-	-
Self rate of overall health compared to a year ago	General health	351.00	-0.12	0.14	0.39	0.00	0.79	-	-
Physical limitation to participate in steneous activities: running, lifting heavy objects, taking part in physically demanding sports	General health	352.00	0.29	0.15	0.05	0.00	0.43	-	-
Physical limitation to participate in moderately demanding activities: moving a table, vacuuming, walking in the forest or gardening	General health	352.00	0.14	0.14	0.33	0.57	0.13	-	-
Physical limitation to participate in moderately demanding activities: lifting or carrying grocery bags	General health	351.00	0.00	0.14	0.98	0.14	0.28	-	-
Physical limitation to participate in moderately demanding activities: walking up several stairs	General health	350.00	0.05	0.15	0.75	0.58	0.12	-	-
Physical limitation to participate in moderately demanding activities: bending down or kneeling	General health	351.00	0.17	0.14	0.23	0.45	0.18	-	-
Physical limitation to participate in moderately demanding activities: walking more than two km	General health	351.00	0.10	0.15	0.49	0.00	0.32	-	-
Physical limitation that reduced the normal time spent at work or in other activities during the last four weeks	General health	351.00	0.42	0.43	0.33	0.76	0.04	-	-
Physical limitation that made you do less than you wanted during the last four weeks	General health	350.00	0.53	0.35	0.13	0.00	0.78	-	-
Physical limitation that made you not being able to perform certain work tasks or other activities during the last four weeks	General health	348.00	0.45	0.36	0.20	0.78	0.03	-	-

Physical limitation that limited your ability to perform certain work tasks or other activities during the last four weeks	General health	349.00	0.69	0.41	0.09	0.68	0.08	-	-
Emotiol problems that made you do less than you wanted during the last four weeks	General health	351.00	0.78	0.41	0.06	0.00	0.72	-	-
Extent to what your physical and emotiol health disrupted your usual social life during the last four weeks	General health	354.00	0.34	0.14	0.01	0.70	0.07	-	-
Pain during the last four weeks	General health	353.00	0.33	0.14	0.02	0.45	0.18	-	-
How much has the pain during the last four weeks disturbed your normal work?	General health	352.00	0.31	0.14	0.02	0.55	0.14	-	-
For how much of the time during the last four weeks have you felt really alert and strong?	General health	354.00	-0.42	0.14	0.00	0.00	0.32	-	-
For how much of the time during the last four weeks have you felt very nervous?	General health	354.00	0.25	0.14	0.08	0.00	0.82	-	-
For how much of the time during the last four weeks have you felt so depressed that nothing could cheer you up?	General health	353.00	0.24	0.14	0.08	0.38	0.21	-	-
For how much of the time during the last four weeks have you felt calm and serene?	General health	353.00	-0.30	0.14	0.03	0.00	0.63	-	-
For how much of the time during the last four weeks have you felt full of energy?	General health	352.00	-0.33	0.14	0.02	0.00	0.37	-	-
For how much of the time during the last four weeks have you felt gloomy and sad?	General health	351.00	0.02	0.14	0.89	0.60	0.11	-	-
For how much of the time during the last four weeks have you felt worn out?	General health	350.00	0.08	0.14	0.57	0.57	0.13	-	-
For how much of the time during the last four weeks have you felt happy?	General health	354.00	-0.38	0.14	0.01	0.00	0.76	-	-
For how much of the time during the last four weeks have you felt tired?	General health	352.00	0.25	0.14	0.07	0.00	0.71	-	-
Long-term sickness	General health	26481.00	0.13	0.04	0.00	0.00	0.66	-	-
Work shifts/weekends	Psychosocial	27407.00	0.14	0.03	0.00	0.00	0.82	0.70	19.00
Number of cigarretes smoked per day (in groups)	Tobacco use	23608.00	0.10	0.01	0.00	0.77	0.04	0.71	3.00
Years smoking	Tobacco use	25782.00	0.12	0.01	0.00	0.00	0.46	0.71	6.00
Grams of tobacco smoked per week	Tobacco use	18064.00	0.03	0.02	0.10	0.00	0.34	-	-
Number of cigarretes smoked per day	Tobacco use	18738.00	0.12	0.02	0.00	0.62	0.11	0.71	1.00
Number of cigars smoked per day	Tobacco use	18023.00	0.04	0.02	0.01	0.00	0.51	-	-
Smoking status: Smokers vs non-smokers	Tobacco use	19283.00	0.38	0.04	0.00	0.13	0.28	0.71	5.00
Smoking status: Former smokers vs non-smokers	Tobacco use	20039.00	0.02	0.03	0.54	0.00	0.93	-	-
Smoking status: Former occasiol smokers vs non-smokers	Tobacco use	16751.00	0.07	0.05	0.14	0.00	0.77	-	-
Number of snuff boxes per week	Tobacco use	27713.00	0.11	0.01	0.00	0.00	0.58	0.70	9.00
Snuff status: Snuff users vs non-snuff users	Tobacco use	24717.00	0.28	0.04	0.00	0.00	0.87	0.71	2.00
Snuff status: Former snuff users vs non-snuff users	Tobacco use	22819.00	-0.07	0.05	0.14	0.00	0.79	-	-
Years using snuff	Tobacco use	26942.00	0.06	0.01	0.00	0.00	0.86	0.71	4.00
Participation in associations or voluntary organisations	Social	28958.00	-0.02	0.03	0.45	0.00	0.99	-	-
Participation in sports or physical exercise associations	Social	1467.00	-0.22	0.12	0.07	0.54	0.14	-	-
Participation in study circles	Social	1467.00	0.41	0.17	0.01	0.61	0.11	-	-
Participation in other association	Social	1467.00	0.15	0.12	0.21	0.85	0.01	-	-
People to ask for help apart from the ones at home	Social	29187.00	-0.14	0.04	0.00	0.47	0.17	-	-
Number of friends that can come to your home at any time and feel at home	Social	28937.00	-0.03	0.01	0.02	0.00	0.97	-	-
Number of social contacts with the same interests as you	Social	29170.00	0.01	0.01	0.58	0.00	0.70	-	-
Would you say that the number of people that you meet in your everyday life is enough or would you like to meet more or fewer people?	Social	29243.00	0.02	0.01	0.07	0.00	0.32	-	-
Close relationship with anyone	Social	29002.00	-0.06	0.01	0.00	0.00	0.93	0.70	32.00
Frequency of engaging in clubs, associations or study circles	Social	19549.00	0.00	0.02	0.76	0.73	0.05	-	-
Number of social interactions during a normal week	Social	28994.00	-0.01	0.01	0.70	0.00	0.60	-	-
Support from others	Social	29271.00	-0.05	0.01	0.00	0.00	0.74	-	-
Number of people with whom you can speak openly	Social	29242.00	-0.05	0.01	0.00	0.23	0.26	-	-
Receive hugs to comfort and support you	Social	28860.00	-0.14	0.04	0.00	0.70	0.07	-	-
Stigmasterol intake (mg/day)	Nutrients	25801.00	0.06	0.01	0.00	0.00	0.44	-	-
Tiamin intake (mg/day)	Nutrients	25801.00	0.07	0.01	0.00	0.00	0.71	0.70	13.00
Vitamin E intake (mg/day)	Nutrients	25801.00	0.03	0.01	0.06	0.00	0.61	-	-
Trans fat intake (g/day)	Nutrients	25801.00	0.02	0.02	0.27	0.00	0.87	-	-
Sum of phytosterols intake (mg/day)	Nutrients	25801.00	0.01	0.01	0.56	0.00	0.56	-	-
Educatiol level	Psychosocial	29228.00	-0.11	0.01	0.00	0.00	0.76	0.70	25.00
Zinc intake (mg/day)	Nutrients	25801.00	0.00	0.01	0.84	0.00	0.64	-	-

Supplementary Table 13. Longitudinal association results for SBP

Description	Group	N	Effect estimate	S.E.	p-value	I^2	Q p-value	adjusted R2	R2 rank
Alcohol intake (g/day)	Alcohol	25506.00	0.24	0.09	0.01	0.00	0.49	-	-
Permanent employment	Psychosocial	28426.00	0.31	0.20	0.11	0.74	0.05	-	-
Self-employed	Psychosocial	28171.00	0.08	0.30	0.79	0.00	0.55	-	-
Distance to work in kilometers (one way)	Physical activity	25373.00	0.02	0.09	0.84	0.00	0.47	-	-
Last time a colleague visited you at home	Psychosocial	27423.00	0.08	0.09	0.36	0.00	0.57	-	-
Job demands to work very fast	Psychosocial	28626.00	0.26	0.09	0.00	0.00	0.62	-	-
Frequency of social contacts with colleagues during leisure time	Psychosocial	27163.00	-0.12	0.09	0.19	0.12	0.29	-	-
High physical demand from job	Physical activity	28692.00	0.28	0.09	0.00	0.00	0.55	-	-
Enough time for job assignments	Psychosocial	28509.00	0.16	0.09	0.07	0.12	0.29	-	-
Control over planning and execution of the workday	Psychosocial	28646.00	-0.17	0.09	0.05	0.00	0.88	-	-
Ingenuity or creativity demand from job	Psychosocial	28532.00	-0.26	0.09	0.00	0.00	0.92	-	-
Frequent social contacts with colleagues during work	Psychosocial	27550.00	-0.02	0.09	0.86	0.00	0.68	-	-
Contradictory demands in job	Psychosocial	28409.00	-0.34	0.09	0.00	0.00	0.86	-	-
Possibility to leave your work for a while to speak with a colleague	Psychosocial	27576.00	0.01	0.09	0.94	0.15	0.28	-	-
Learn new things at job	Psychosocial	28592.00	-0.24	0.09	0.01	0.00	0.52	-	-
High mental demand from job	Psychosocial	28452.00	0.25	0.09	0.00	0.00	0.42	-	-
Repetitive job	Psychosocial	28610.00	0.39	0.09	0.00	0.00	0.36	-	-
Skill demand from job	Psychosocial	28553.00	-0.17	0.09	0.05	0.00	0.48	-	-
Possibility to speak with colleagues during breaks	Psychosocial	27739.00	0.10	0.09	0.26	0.00	0.64	-	-
Control over own work assignment	Psychosocial	28617.00	-0.06	0.09	0.50	0.00	0.39	-	-
Vitamin C intake (mg/day)	Nutrients	25506.00	0.11	0.09	0.23	0.41	0.19	-	-
Vitamin B12 intake (ug/day)	Nutrients	25506.00	0.02	0.09	0.85	0.00	0.51	-	-
Vitamin B2 intake (ug/day)	Nutrients	25506.00	-0.10	0.09	0.25	0.00	0.70	-	-
Vitamin B6 intake (mg/day)	Nutrients	25506.00	0.05	0.09	0.62	0.00	0.69	-	-
Informed of having high blood pressure	General health	28835.00	4.83	0.27	0.00	0.00	0.52	0.42	1.00
Beta-sitostanol intake (mg/day)	Nutrients	25506.00	0.01	0.09	0.87	0.11	0.29	-	-
Beta-sitosterol intake (mg/day)	Nutrients	25506.00	-0.16	0.09	0.07	0.00	0.57	-	-
Marital status: Single vs Married/partner	Social	26962.00	0.08	0.29	0.77	0.00	0.81	-	-
Marital status: Single vs Divorced/separated	Social	4630.00	-0.44	0.48	0.36	0.00	0.50	-	-
Marital status: Single vs Widow/widower	Social	3044.00	0.43	1.20	0.72	0.00	0.35	-	-
Campestanol intake (mg/day)	Nutrients	25506.00	-0.01	0.09	0.94	0.00	0.44	-	-
Campesterol intake (mg/day)	Nutrients	25506.00	-0.15	0.09	0.11	0.00	0.35	-	-
Parents or siblings have diabetes	General health	28534.00	0.34	0.23	0.14	0.00	0.63	-	-
Disaccharides intake (g/day)	Nutrients	25506.00	-0.12	0.09	0.18	0.56	0.13	-	-
Vitamin D intake (ug/day)	Nutrients	25506.00	-0.15	0.09	0.11	0.32	0.23	-	-

Total energy intake (kcal/day)	Nutrients	25506.00	-0.47	0.10	0.00	0.00	0.69	-	-
Formic acid intaje (g/day)	Nutrients	25506.00	-0.03	0.09	0.71	0.36	0.21	-	-
Pentadecanoic acid intake (g/day)	Nutrients	25506.00	-0.03	0.09	0.72	0.00	0.39	-	-
Palmitic acid intake (g/day)	Nutrients	25506.00	-0.03	0.09	0.78	0.00	0.67	-	-
Heptadecanoic acid intake (g/day)	Nutrients	25506.00	-0.03	0.09	0.72	0.00	0.39	-	-
Linoleic acid intake (g/day)	Nutrients	25506.00	-0.12	0.09	0.20	0.00	0.56	-	-
Linolenic acid intake (g/day)	Nutrients	25506.00	-0.13	0.09	0.17	0.00	0.55	-	-
Arachidonic acid (ARA) intake (g/day)	Nutrients	25506.00	0.07	0.09	0.48	0.13	0.28	-	-
Eicosapentaenoic acid (EPA) intake (g/day)	Nutrients	25506.00	-0.10	0.09	0.25	0.00	0.92	-	-
Docosahexaenoic acid (DHA) intake (g/day)	Nutrients	25506.00	-0.06	0.09	0.50	0.00	0.90	-	-
Fat intake (g/day)	Nutrients	25506.00	-0.04	0.09	0.70	0.00	0.72	-	-
Fibre intake (g/day)	Nutrients	25506.00	0.06	0.10	0.56	0.00	0.71	-	-
Folic acid intake (ug/day)	Nutrients	25506.00	-0.03	0.10	0.73	0.00	0.66	-	-
Phosphate intake (mg/day)	Nutrients	25506.00	-0.26	0.09	0.00	0.00	0.86	-	-
Whole grain intake (g/day)	Food	25506.00	-0.21	0.09	0.02	0.00	0.33	-	-
Travel to work: Walk to work vs passive travel to work	Physical activity	18442.00	-0.52	0.33	0.12	0.00	0.68	-	-
Travel to work: Cycle to work vs passive travel to work	Physical activity	22304.00	-0.62	0.22	0.00	0.00	0.79	-	-
Travel to work: Irregular travel mode to work vs passive travel to work	Physical activity	18898.00	-0.19	0.31	0.54	0.28	0.24	-	-
Sedentary or standing work	Physical activity	28161.00	0.17	0.19	0.39	0.00	0.44	-	-
Light but partly physically active work	Physical activity	28161.00	-0.49	0.22	0.03	0.31	0.23	-	-
Light and physically active work	Physical activity	28161.00	0.00	0.20	0.98	0.00	0.38	-	-
Sometimes physically straining work	Physical activity	28161.00	0.28	0.19	0.14	0.00	0.57	-	-
Frequency of walking during leisure time	Physical activity	27879.00	0.17	0.09	0.05	0.71	0.06	-	-
Frequency of cycling during leisure time	Physical activity	24426.00	0.07	0.09	0.47	0.00	0.75	-	-
Frequency of dancing during leisure time	Physical activity	25073.00	-0.04	0.09	0.62	0.22	0.26	-	-
Frequency of shoveling snow during leisure time	Physical activity	27278.00	0.14	0.09	0.11	0.00	0.98	-	-
Frequency of gardening during leisure time	Physical activity	27063.00	0.30	0.09	0.00	0.45	0.18	-	-
Frequency of hunting or fishing during leisure time	Physical activity	26357.00	0.21	0.10	0.03	0.13	0.28	-	-
Frequency of picking berries or mushrooms during leisure time	Physical activity	26927.00	0.17	0.09	0.06	0.26	0.24	-	-
Changed everyday exercise during the last year	Physical activity	28742.00	0.31	0.08	0.00	0.00	0.90	-	-
Everyday exercise satisfaction	Physical activity	28659.00	0.34	0.09	0.00	0.00	0.32	-	-
Exercise during the last three months	Physical activity	28637.00	-0.25	0.09	0.00	0.00	0.40	-	-
If you exercise, change in exercise habits during the last year	Physical activity	24490.00	0.33	0.09	0.00	0.00	0.81	-	-
Bregott on bread	Food	25506.00	-0.06	0.09	0.54	0.00	0.40	-	-
Whole grain crisp bread	Food	25506.00	0.12	0.09	0.19	0.43	0.19	-	-
Whole grain soft bread	Food	25506.00	-0.02	0.09	0.86	0.00	0.44	-	-

White (soft) bread, thin crisp bread	Food	25506.00	0.12	0.09	0.21	0.72	0.06	-	-
Coffee rolls/buns, rusk	Food	25506.00	0.05	0.09	0.59	0.00	0.72	-	-
Cheese 28%	Food	25506.00	-0.23	0.09	0.01	0.00	0.34	-	-
Cheese 10-17%	Food	25506.00	0.10	0.09	0.28	0.85	0.01	-	-
Soft cheese	Food	12810.00	0.09	0.13	0.47	0.00	0.64	-	-
Soft whey cheese	Food	12810.00	-0.16	0.13	0.22	0.00	0.58	-	-
Sausage, liver pate on bread	Food	25506.00	0.01	0.09	0.94	0.00	0.92	-	-
Meat on bread	Food	25506.00	0.25	0.09	0.00	0.00	0.48	-	-
Butter on bread	Food	25506.00	0.15	0.09	0.08	0.00	0.53	-	-
Oatflake, whole wheat, rye or barley porridge	Food	25506.00	-0.32	0.09	0.00	0.00	0.36	-	-
Rosehip, sweet syrup soup	Food	25506.00	0.05	0.09	0.60	0.00	0.66	-	-
Sour milk, yoghurt (3% fat)	Food	25506.00	-0.07	0.09	0.45	0.82	0.02	-	-
Sour milk, yoghurt (low fat)	Food	25506.00	-0.06	0.09	0.48	0.78	0.03	-	-
Fiber cereals	Food	25506.00	-0.12	0.09	0.18	0.07	0.30	-	-
Corn flakes	Food	25506.00	-0.05	0.09	0.59	0.00	0.36	-	-
Berries (fresh or frozen)	Food	25506.00	0.04	0.09	0.65	0.00	0.91	-	-
Apple, pear, peach, orange, mandarin and grapefruit	Food	25506.00	-0.12	0.10	0.23	0.00	0.64	-	-
Ba	Food	25506.00	0.00	0.09	0.96	0.00	0.38	-	-
Root vegetables and carrot	Food	25506.00	0.01	0.10	0.91	0.00	0.81	-	-
Low fat margarine on bread	Food	25506.00	-0.03	0.09	0.74	0.00	0.48	-	-
Tomato and cucumber	Food	25506.00	-0.04	0.09	0.70	0.00	0.73	-	-
White cabbage, lettuce, lettuce cabbage, spich, borecole and broccoli	Food	25506.00	-0.07	0.10	0.48	0.00	0.98	-	-
Mixed frozen vegetables	Food	12810.00	0.12	0.13	0.37	0.09	0.29	-	-
Boiled or baked potato	Food	25506.00	0.05	0.09	0.59	0.00	0.66	-	-
Fried potatoes and pommes frites	Food	25506.00	0.21	0.09	0.02	0.00	0.67	-	-
Mashed potato	Food	12810.00	0.02	0.13	0.87	0.00	0.59	-	-
Potato salad	Food	10439.00	0.06	0.14	0.68	0.00	0.50	-	-
Rice	Food	25506.00	-0.25	0.09	0.01	0.00	0.37	-	-
Pasta	Food	25506.00	-0.26	0.09	0.01	0.00	0.71	-	-
Brown beans and pea soup	Food	25506.00	0.19	0.09	0.03	0.00	0.81	-	-
Margarine on bread	Food	25506.00	0.10	0.09	0.27	0.00	0.95	-	-
Blota (broth + bread)	Food	12810.00	0.22	0.13	0.09	0.00	0.43	-	-
Pancake, waffle and Swedish dumpling	Food	25506.00	-0.02	0.09	0.84	0.00	0.77	-	-
Pizza	Food	25506.00	-0.07	0.10	0.50	0.00	0.45	-	-
Minced meat dishes	Food	25506.00	0.00	0.09	0.97	0.53	0.14	-	-
Meat stew	Food	25506.00	-0.08	0.09	0.35	0.00	0.33	-	-
Steak, chop, etc.	Food	25506.00	0.10	0.09	0.26	0.00	0.74	-	-
Bacon	Food	25506.00	0.10	0.09	0.25	0.00	0.36	-	-
Sausage as main dish	Food	25506.00	-0.04	0.09	0.66	0.00	0.81	-	-
Hamburger	Food	25506.00	0.23	0.09	0.01	0.00	0.55	-	-
White meat (poultry)	Food	25506.00	-0.09	0.09	0.35	0.82	0.02	-	-
Butter for cooking	Food	25506.00	0.09	0.09	0.31	0.00	0.81	-	-
Blood based food	Food	12810.00	0.00	0.13	0.97	0.00	0.98	-	-
Liver and kidney	Food	12810.00	-0.06	0.13	0.66	0.00	0.43	-	-
Lean fish (e.g. perch, bass, cod)	Food	25506.00	0.12	0.09	0.19	0.57	0.13	-	-
Fatty fish (e.g. herring, whitefish, salmon)	Food	25506.00	-0.05	0.09	0.61	0.00	0.99	-	-
Shellfish (e.g. shrimps, scallops)	Food	12810.00	0.01	0.13	0.95	0.00	0.45	-	-
Salty fish	Food	25506.00	-0.05	0.09	0.55	0.00	0.55	-	-
Smoked fish/meat	Food	25506.00	0.10	0.09	0.29	0.61	0.11	-	-
Ice cream	Food	25506.00	-0.30	0.09	0.00	0.31	0.23	-	-
Sweets (chocolate and candy)	Food	25506.00	0.03	0.09	0.79	0.00	0.58	-	-

Sugar, honey, marmelade and jam	Food	25506.00	-0.05	0.09	0.58	0.00	0.35	-	-
Margarine for cooking	Food	25506.00	-0.06	0.09	0.50	0.10	0.29	-	-
Cookies and pastry	Food	25506.00	-0.25	0.09	0.00	0.00	0.48	-	-
Chips, popcorn and salted nuts	Food	25506.00	0.19	0.09	0.04	0.64	0.10	-	-
Low fat milk (0.5%)	Beverage	25506.00	-0.04	0.09	0.67	0.00	0.99	-	-
Milk, sour milk (1.5%)	Beverage	25506.00	-0.02	0.09	0.79	0.69	0.07	-	-
Milk, sour milk (3%)	Beverage	25506.00	0.02	0.09	0.84	0.00	0.71	-	-
Sodas, soft drinks and juice	Beverage	25506.00	0.07	0.09	0.46	0.00	0.86	-	-
Brewed (filtered) coffee	Beverage	25506.00	-0.03	0.09	0.71	0.85	0.01	-	-
Boiled coffee	Beverage	25506.00	0.01	0.09	0.90	0.00	0.67	-	-
Tea	Beverage	25506.00	-0.23	0.09	0.01	0.48	0.17	-	-
Light beer	Alcohol	25506.00	-0.15	0.09	0.11	0.00	0.56	-	-
Oil for cooking	Food	25506.00	-0.07	0.09	0.45	0.00	0.68	-	-
Medium beer	Alcohol	25506.00	0.24	0.09	0.01	0.54	0.14	-	-
Strong beer	Alcohol	25506.00	0.09	0.10	0.35	0.00	0.74	-	-
Wine	Alcohol	25506.00	0.10	0.09	0.26	0.00	0.82	-	-
Liquor and spirits	Alcohol	25506.00	0.27	0.09	0.00	0.00	0.45	-	-
Salad dressing with oil	Food	25506.00	-0.01	0.09	0.87	0.00	0.67	-	-
Cream, creme fraiche, sour cream	Food	25506.00	0.01	0.09	0.94	0.57	0.13	-	-
Average portion size of vegetables based on photographic illustration of four sizes (smallest to largest)	Food	25506.00	-0.38	0.09	0.00	0.00	0.92	-	-
Overall state of health during the last year	General health	28706.00	0.12	0.09	0.18	0.00	0.89	-	-
Overall state of health compared to others your age	General health	24967.00	-0.39	0.09	0.00	0.00	0.46	-	-
Parents or siblings had a cerebral hemorrhage/thrombosis or cardiac infarction before the age of 60	General health	28487.00	1.16	0.22	0.00	0.00	0.39	0.41	3.00
Teetotaler	Alcohol	28347.00	-0.03	0.29	0.92	0.00	0.78	-	-
Feel the need to reduce alcohol consumption	Alcohol	25641.00	0.35	0.29	0.23	0.00	0.98	-	-
Feel uneasy or guilty because of your way of drinking	Alcohol	25621.00	-0.24	0.28	0.39	0.80	0.03	-	-
Iron intake (mg/day)	Nutrients	25506.00	0.05	0.10	0.64	0.24	0.25	-	-
Iodine intake (ug/day)	Nutrients	25506.00	-0.22	0.09	0.02	0.81	0.02	-	-
Calcium intake (mg/day)	Nutrients	25506.00	-0.11	0.09	0.22	0.37	0.21	-	-
Potassium intake (mg/day)	Nutrients	25506.00	-0.04	0.10	0.71	0.00	0.96	-	-
Beta-carotene intake (mg/day)	Nutrients	25506.00	0.01	0.10	0.88	0.00	0.79	-	-
Cholesterol intake (g/day)	Nutrients	25506.00	0.01	0.10	0.95	0.00	0.89	-	-
Carbohydrates intake (g/day)	Nutrients	25506.00	-0.02	0.09	0.81	0.00	0.59	-	-
Average portion size of meat/fish based on photographic illustration of four sizes (smallest to largest)	Food	25506.00	-0.10	0.10	0.33	0.00	0.99	-	-
Breakfast habits: Only coffee/tea for breakfast vs not breakfast at all	Food	1577.00	-1.51	1.22	0.21	0.00	0.70	-	-
Breakfast habits: Coffee/tea and wheat buns or rusk for breakfast vs not breakfast at all	Food	1964.00	-1.68	0.75	0.02	0.80	0.03	-	-
Breakfast habits: Porridge w/o sandwich for breakfast vs not breakfast at all	Food	3412.00	-2.59	0.54	0.00	0.40	0.20	-	-

Breakfast habits: Gruel w/o sandwich for breakfast vs not breakfast at all	Food	1853.00	-1.75	0.87	0.04	0.00	0.82	-	-
Eat breakfast from 2000	Food	2287.00	-2.08	0.93	0.02	0.00	0.88	-	-
Enterodiol intake (ug/day)	Nutrients	25506.00	-0.02	0.09	0.82	0.00	0.80	-	-
Enterolactone intake (ug/day)	Nutrients	25506.00	-0.06	0.09	0.54	0.22	0.26	-	-
Equol intake (ug/day)	Nutrients	25506.00	-0.06	0.09	0.48	0.12	0.29	-	-
Lariciresinol intake (ug/day)	Nutrients	25506.00	-0.08	0.09	0.37	0.00	0.33	-	-
Matairesinol intake (ug/day)	Nutrients	25506.00	-0.02	0.09	0.82	0.00	0.68	-	-
Medioresinol intake (ug/day)	Nutrients	25506.00	0.08	0.09	0.38	0.00	0.38	-	-
Pinoresinol intake (ug/day)	Nutrients	25506.00	-0.06	0.09	0.49	0.00	0.58	-	-
Secoisolariciresinol intake (ug/day)	Nutrients	25506.00	0.03	0.09	0.72	0.77	0.04	-	-
Sum of all ligns intake (ug/day)	Nutrients	25506.00	0.02	0.09	0.81	0.00	0.38	-	-
Syringaresinol intake (ug/day)	Nutrients	25506.00	0.05	0.09	0.59	0.00	0.45	-	-
Sum of Lariciresinol, Matairesinol, Pinoresinol, Secoisolariciresinol intake (ug/day)	Nutrients	25506.00	-0.06	0.09	0.49	0.00	0.35	-	-
Satisfaction with home and family situation	Psychosocial	13577.00	0.32	0.12	0.01	0.00	0.62	-	-
Appetite status	Psychosocial	13567.00	-0.01	0.12	0.93	0.00	0.68	-	-
Mood status	Psychosocial	13565.00	0.21	0.12	0.09	0.00	0.75	-	-
Energy status	Psychosocial	13553.00	0.39	0.12	0.00	0.00	0.93	-	-
Patience status	Psychosocial	13569.00	0.24	0.12	0.05	0.00	0.82	-	-
Confidence status	Psychosocial	13562.00	0.26	0.12	0.03	0.00	0.59	-	-
Sleep status	Sleep	13591.00	0.10	0.12	0.41	0.00	0.67	-	-
Do you feel important and appreciated outside your home?	Psychosocial	13581.00	0.10	0.12	0.39	0.00	0.58	-	-
Do you feel important and appreciated in your home?	Psychosocial	13476.00	0.18	0.12	0.14	0.00	0.54	-	-
Satisfaction with accomodation	Psychosocial	13585.00	0.12	0.12	0.32	0.00	0.71	-	-
Satisfaction with work situation	Psychosocial	13444.00	0.14	0.12	0.25	0.00	0.95	-	-
Satisfaction with economy	Psychosocial	13567.00	0.03	0.12	0.79	0.00	0.41	-	-
Satisfaction with leisure time	Psychosocial	13557.00	0.31	0.12	0.01	0.00	0.84	-	-
Hearing status	General health	13581.00	0.07	0.12	0.57	0.75	0.05	-	-
Vision status	General health	13567.00	-0.15	0.13	0.24	0.00	0.42	-	-
Memory status	Psychosocial	13544.00	0.50	0.12	0.00	0.00	0.63	-	-
Fitness status	Physical activity	13559.00	0.06	0.13	0.64	0.00	0.33	-	-
Magnesium intake (mg/day)	Nutrients	25506.00	-0.24	0.10	0.01	0.00	0.57	-	-
Saturated fat intake (g/day)	Nutrients	25506.00	-0.01	0.09	0.91	0.00	0.40	-	-
Monounsaturated fat intake (g/day)	Nutrients	25506.00	0.01	0.10	0.89	0.00	0.49	-	-
Monosaccharides intake (g/day)	Nutrients	25506.00	-0.08	0.10	0.39	0.00	0.36	-	-
Sodium intake (mg/day)	Nutrients	25506.00	-0.13	0.09	0.16	0.56	0.13	-	-
Vitamin B3 intake (mg/day)	Nutrients	25506.00	0.06	0.09	0.53	0.80	0.03	-	-
Cambridge physical activity index	Physical activity	28072.00	-0.11	0.09	0.22	0.18	0.27	-	-
Polyunsaturated fat intake (g/day)	Nutrients	25506.00	-0.13	0.09	0.16	0.00	0.64	-	-

Average portion size of potatoes/rice/pasta based on photographic illustration of four sizes (smallest to largest)	Food	25506.00	-0.48	0.10	0.00	0.00	0.37	-	-
Total protein intake (g/day)	Nutrients	25506.00	0.01	0.09	0.94	0.00	0.90	-	-
Animal based protein intake (g/day)	Nutrients	25506.00	-0.01	0.09	0.91	0.00	0.95	-	-
Plant based protein intake (g/day)	Nutrients	25506.00	0.04	0.09	0.66	0.00	0.66	-	-
Vitamin A intake (mg/day)	Nutrients	25506.00	0.04	0.10	0.71	0.00	0.43	-	-
Sucrose intake (g/day)	Nutrients	25506.00	0.03	0.09	0.75	0.54	0.14	-	-
Cohabitation: Live alone vs Only one adult (spouse, partner)	Social	9158.00	0.73	0.37	0.05	0.00	0.67	-	-
Cohabitation: Live alone vs Only children	Social	3832.00	0.11	0.56	0.85	0.00	0.33	-	-
Cohabitation: Live alone vs Adult and children	Social	20046.00	0.04	0.31	0.89	0.07	0.30	-	-
Cohabitation: Live alone vs Other/others	Social	2953.00	-0.11	0.76	0.88	0.00	0.78	-	-
Selenium intake (ug/day)	Nutrients	25506.00	-0.01	0.09	0.89	0.00	0.78	-	-
Self rate of overall health	General health	352.00	0.20	0.74	0.78	0.00	0.95	-	-
For how much of the time during the last four weeks has your physical health or your emotiol problems limited your ability to interact with others?	General health	350.00	-0.47	0.73	0.52	0.00	0.95	-	-
Get sick more often than other people	General health	351.00	-0.50	0.72	0.49	0.00	0.67	-	-
As healthy as anyone	General health	352.00	-0.04	0.72	0.95	0.79	0.03	-	-
Worsen in health in the future	General health	348.00	0.69	0.73	0.35	0.00	0.94	-	-
Excellent health	General health	351.00	-0.29	0.74	0.69	0.80	0.03	-	-
Self rate of overall health compared to a year ago	General health	351.00	-0.51	0.72	0.48	0.54	0.14	-	-
Physical limitation to participate in steneous activities: running, lifting heavy objects, taking part in physically demanding sports	General health	352.00	0.71	0.76	0.35	0.00	0.44	-	-
Physical limitation to participate in moderately demanding activities: moving a table, vacuuming, walking in the forest or gardening	General health	352.00	0.35	0.73	0.63	0.00	0.71	-	-
Physical limitation to participate in moderately demanding activities: lifting or carrying grocery bags	General health	351.00	1.29	0.74	0.08	0.00	0.68	-	-
Physical limitation to participate in moderately demanding activities: walking up several stairs	General health	350.00	-0.11	0.76	0.89	0.00	0.58	-	-
Physical limitation to participate in moderately demanding activities: bending down or kneeling	General health	351.00	0.01	0.74	0.99	0.00	0.42	-	-
Physical limitation to participate in moderately demanding activities: walking more than two km	General health	351.00	0.43	0.76	0.57	0.00	0.95	-	-

Physical limitation that reduced the normal time spent at work or in other activities during the last four weeks	General health	351.00	3.83	2.21	0.08	0.00	0.62	-	-
Physical limitation that made you do less than you wanted during the last four weeks	General health	350.00	3.11	1.80	0.08	0.00	0.92	-	-
Physical limitation that made you not being able to perform certain work tasks or other activities during the last four weeks	General health	348.00	4.08	1.87	0.03	0.00	0.84	-	-
Physical limitation that limited your ability to perform certain work tasks or other activities during the last four weeks	General health	349.00	3.49	2.10	0.10	0.00	0.56	-	-
Emotiol problems that made you do less than you wanted during the last four weeks	General health	351.00	-0.33	2.12	0.88	0.70	0.07	-	-
Extent to what your physical and emotiol health disrupted your usual social life during the last four weeks	General health	354.00	-0.18	0.72	0.80	0.00	0.47	-	-
Pain during the last four weeks	General health	353.00	1.00	0.72	0.17	0.00	0.89	-	-
How much has the pain during the last four weeks disturbed your normal work?	General health	352.00	0.75	0.72	0.30	0.00	0.96	-	-
For how much of the time during the last four weeks have you felt really alert and strong?	General health	354.00	-0.15	0.74	0.84	0.00	0.84	-	-
For how much of the time during the last four weeks have you felt very nervous?	General health	354.00	0.41	0.72	0.57	0.00	0.41	-	-
For how much of the time during the last four weeks have you felt so depressed that nothing could cheer you up?	General health	353.00	-0.36	0.72	0.62	0.38	0.20	-	-
For how much of the time during the last four weeks have you felt calm and serene?	General health	353.00	-0.39	0.72	0.59	0.00	0.61	-	-
For how much of the time during the last four weeks have you felt full of energy?	General health	352.00	-0.45	0.74	0.54	0.00	0.58	-	-
For how much of the time during the last four weeks have you felt gloomy and sad?	General health	351.00	-0.73	0.74	0.32	0.00	0.99	-	-
For how much of the time during the last four weeks have you felt worn out?	General health	350.00	-0.09	0.74	0.90	0.00	0.43	-	-
For how much of the time during the last four weeks have you felt happy?	General health	354.00	-0.33	0.73	0.65	0.00	0.41	-	-
For how much of the time during the last four weeks have you felt tired?	General health	352.00	-0.48	0.74	0.52	0.00	0.68	-	-
Long-term sickness	General health	26174.00	0.17	0.27	0.53	0.00	0.48	-	-
Work shifts/weekends	Psychosocial	27091.00	0.24	0.20	0.22	0.00	0.54	-	-

Number of cigarretes smoked per day (in groups)	Tobacco use	23330.00	0.13	0.09	0.19	0.74	0.05	-	-
Years smoking	Tobacco use	25492.00	0.16	0.09	0.09	0.66	0.09	-	-
Grams of tobacco smoked per week	Tobacco use	17855.00	0.03	0.11	0.80	0.51	0.15	-	-
Number of cigarretes smoked per day	Tobacco use	18525.00	0.40	0.10	0.00	0.00	0.92	-	-
Number of cigars smoked per day	Tobacco use	17814.00	0.07	0.11	0.49	0.00	0.57	-	-
Smoking status: Smokers vs non-smokers	Tobacco use	19050.00	0.56	0.24	0.02	0.61	0.11	-	-
Smoking status: Former smokers vs non-smokers	Tobacco use	19812.00	0.05	0.23	0.81	0.00	0.52	-	-
Smoking status: Former occasiol smokers vs non-smokers	Tobacco use	16553.00	-0.04	0.31	0.91	0.00	0.89	-	-
Number of snuff boxes per week	Tobacco use	27393.00	0.33	0.09	0.00	0.00	0.32	-	-
Snuff status: Snuff users vs non-snuff users	Tobacco use	24420.00	0.69	0.25	0.01	0.00	0.43	-	-
Snuff status: Former snuff users vs non-snuff users	Tobacco use	22540.00	-0.40	0.30	0.17	0.00	0.50	-	-
Years using snuff	Tobacco use	26631.00	0.16	0.10	0.09	0.20	0.26	-	-
Participation in associations or voluntary organisations	Social	28628.00	-0.58	0.18	0.00	0.00	0.37	-	-
Participation in sports or physical exercise associations	Social	1461.00	0.21	0.71	0.77	0.58	0.12	-	-
Participation in study circles	Social	1461.00	-0.85	1.00	0.39	0.00	0.43	-	-
Participation in other association	Social	1461.00	-1.62	0.70	0.02	0.74	0.05	-	-
People to ask for help apart from the ones at home	Social	28853.00	-0.06	0.28	0.84	0.00	0.43	-	-
Number of friends that can come to your home at any time and feel at home	Social	28609.00	-0.03	0.09	0.71	0.00	0.75	-	-
Number of social contacts with the same interests as you	Social	28839.00	-0.16	0.09	0.06	0.00	0.32	-	-
Would you say that the number of people that you meet in your everyday life is enough or would you like to meet more or fewer people?	Social	28911.00	-0.35	0.08	0.00	0.00	0.81	-	-
Close relationship with anyone	Social	28672.00	-0.12	0.09	0.17	0.26	0.25	-	-
Frequency of engaging in clubs, associations or study circles	Social	19315.00	-0.35	0.10	0.00	0.00	0.83	-	-
Number of social interactions during a normal week	Social	28665.00	-0.02	0.09	0.77	0.00	0.89	-	-
Support from others	Social	28938.00	-0.19	0.09	0.03	0.06	0.30	-	-
Number of people with whom you can speak openly	Social	28910.00	-0.11	0.08	0.20	0.00	0.77	-	-
Receive hugs to comfort and support you	Social	28532.00	-0.51	0.23	0.03	0.00	0.93	-	-
Stigmasterol intake (mg/day)	Nutrients	25506.00	0.00	0.09	0.99	0.00	0.54	-	-
Tiamin intake (mg/day)	Nutrients	25506.00	0.00	0.09	0.98	0.00	0.32	-	-
Vitamin E intake (mg/day)	Nutrients	25506.00	0.01	0.09	0.89	0.00	0.62	-	-
Trans fat intake (g/day)	Nutrients	25506.00	-0.17	0.11	0.12	0.00	0.68	-	-
Sum of phytosterols intake (mg/day)	Nutrients	25506.00	-0.15	0.09	0.10	0.00	0.44	-	-

Educatiol level	Psychosocial	28895.00	-0.75	0.09	0.00	0.00	0.61	0.41	2.00
Zinc intake (mg/day)	Nutrients	25506.00	0.04	0.10	0.68	0.00	0.96	-	-

Supplementary Table 14. Longitudinal association results for DBP

Description	Group	N	Effect estimate	S.E.	p-value	I^2	Q p-value	adjusted R2	R2 rank
Alcohol intake (g/day)	Alcohol	25477.00	0.15	0.06	0.01	0.61	0.11	-	-
Permanent employment	Psychosocial	28395.00	0.31	0.13	0.02	0.00	0.93	-	-
Self-employed	Psychosocial	28140.00	-0.15	0.19	0.42	0.00	0.34	-	-
Distance to work in kilometers (one way)	Physical activity	25346.00	-0.04	0.06	0.50	0.60	0.11	-	-
Last time a colleague visited you at home	Psychosocial	27392.00	0.02	0.06	0.68	0.59	0.12	-	-
Job demands to work very fast	Psychosocial	28596.00	0.15	0.05	0.00	0.00	0.71	-	-
Frequency of social contacts with colleagues during leisure time	Psychosocial	27134.00	-0.12	0.06	0.03	0.31	0.23	-	-
High physical demand from job	Physical activity	28661.00	0.16	0.06	0.00	0.00	0.91	-	-
Enough time for job assignments	Psychosocial	28478.00	-0.01	0.06	0.88	0.00	0.85	-	-
Control over planning and execution of the workday	Psychosocial	28615.00	-0.22	0.06	0.00	0.00	0.76	-	-
Ingenuity or creativity demand from job	Psychosocial	28501.00	-0.07	0.05	0.21	0.00	0.81	-	-
Frequent social contacts with colleagues during work	Psychosocial	27519.00	0.05	0.06	0.35	0.00	0.73	-	-
Contradictory demands in job	Psychosocial	28380.00	-0.09	0.06	0.11	0.00	0.49	-	-
Possibility to leave your work for a while to speak with a colleague	Psychosocial	27545.00	-0.03	0.06	0.55	0.00	0.69	-	-
Learn new things at job	Psychosocial	28561.00	-0.14	0.05	0.01	0.00	0.83	-	-
High mental demand from job	Psychosocial	28421.00	0.07	0.06	0.18	0.61	0.11	-	-
Repetitive job	Psychosocial	28579.00	0.14	0.06	0.01	0.00	0.43	-	-
Skill demand from job	Psychosocial	28522.00	-0.07	0.06	0.22	0.00	0.51	-	-
Possibility to speak with colleagues during breaks	Psychosocial	27708.00	0.11	0.06	0.05	0.00	0.78	-	-
Control over own work assignment	Psychosocial	28586.00	-0.17	0.06	0.00	0.59	0.12	-	-
Vitamin C intake (mg/day)	Nutrients	25477.00	0.05	0.06	0.37	0.00	0.71	-	-
Vitamin B12 intake (ug/day)	Nutrients	25477.00	0.03	0.06	0.62	0.00	0.99	-	-
Vitamin B2 intake (ug/day)	Nutrients	25477.00	-0.07	0.06	0.22	0.00	0.90	-	-
Vitamin B6 intake (mg/day)	Nutrients	25477.00	0.02	0.06	0.77	0.06	0.30	-	-
Informed of having high blood pressure	General health	28803.00	4.10	0.17	0.00	0.41	0.19	0.38	1.00
Beta-sitostanol intake (mg/day)	Nutrients	25477.00	-0.04	0.06	0.53	0.00	0.75	-	-
Beta-sitosterol intake (mg/day)	Nutrients	25477.00	-0.07	0.06	0.26	0.00	0.47	-	-
Marital status: Single vs Married/partner	Social	26931.00	0.21	0.19	0.26	0.00	0.67	-	-
Marital status: Single vs Divorced/separated	Social	4624.00	0.20	0.31	0.51	0.74	0.05	-	-
Marital status: Single vs Widow/widower	Social	3039.00	0.57	0.78	0.46	0.00	0.63	-	-
Campestanol intake (mg/day)	Nutrients	25477.00	-0.05	0.06	0.35	0.00	0.42	-	-
Campesterol intake (mg/day)	Nutrients	25477.00	0.01	0.06	0.82	0.00	0.49	-	-
Parents or siblings have diabetes	General health	28502.00	0.17	0.15	0.26	0.56	0.13	-	-

Disaccharides intake (g/day)	Nutrients	25477.00	-0.06	0.06	0.35	0.00	0.33	-	-
Vitamin D intake (ug/day)	Nutrients	25477.00	0.05	0.06	0.43	0.00	0.36	-	-
Total energy intake (kcal/day)	Nutrients	25477.00	-0.31	0.07	0.00	0.00	0.38	-	-
Formic acid intaje (g/day)	Nutrients	25477.00	0.02	0.06	0.71	0.00	0.40	-	-
Pentadecanoic acid intake (g/day)	Nutrients	25477.00	-0.03	0.06	0.59	0.00	0.48	-	-
Palmitic acid intake (g/day)	Nutrients	25477.00	0.02	0.06	0.73	0.00	0.71	-	-
Heptadecanoic acid intake (g/day)	Nutrients	25477.00	-0.03	0.06	0.59	0.00	0.48	-	-
Linoleic acid intake (g/day)	Nutrients	25477.00	-0.09	0.06	0.15	0.00	0.71	-	-
Linolenic acid intake (g/day)	Nutrients	25477.00	-0.02	0.06	0.79	0.00	0.59	-	-
Arachidonic acid (ARA) intake (g/day)	Nutrients	25477.00	0.03	0.06	0.64	0.00	0.81	-	-
Eicosapentaenoic acid (EPA) intake (g/day)	Nutrients	25477.00	-0.08	0.06	0.15	0.00	0.83	-	-
Docosahexaenoic acid (DHA) intake (g/day)	Nutrients	25477.00	-0.06	0.06	0.32	0.00	0.79	-	-
Fat intake (g/day)	Nutrients	25477.00	0.04	0.06	0.52	0.00	0.72	-	-
Fibre intake (g/day)	Nutrients	25477.00	-0.06	0.06	0.32	0.00	0.85	-	-
Folic acid intake (ug/day)	Nutrients	25477.00	-0.06	0.06	0.32	0.00	0.58	-	-
Phosphate intake (mg/day)	Nutrients	25477.00	-0.14	0.06	0.02	0.00	0.47	-	-
Whole grain intake (g/day)	Food	25477.00	-0.19	0.06	0.00	0.00	0.98	-	-
Travel to work: Walk to work vs passive travel to work	Physical activity	18420.00	-0.30	0.21	0.15	0.00	0.67	-	-
Travel to work: Cycle to work vs passive travel to work	Physical activity	22281.00	-0.50	0.14	0.00	0.00	0.84	-	-
Travel to work: Irregular travel mode to work vs passive travel to work	Physical activity	18872.00	-0.13	0.20	0.52	0.36	0.21	-	-
Sedentary or standing work	Physical activity	28130.00	0.03	0.13	0.82	0.00	0.51	-	-
Light but partly physically active work	Physical activity	28130.00	-0.43	0.14	0.00	0.18	0.27	-	-
Light and physically active work	Physical activity	28130.00	0.05	0.13	0.70	0.00	0.76	-	-
Sometimes physically straining work	Physical activity	28130.00	0.12	0.12	0.30	0.00	0.72	-	-
Frequency of walking during leisure time	Physical activity	27850.00	0.19	0.06	0.00	0.00	0.32	-	-
Frequency of cycling during leisure time	Physical activity	24400.00	0.03	0.06	0.65	0.00	0.97	-	-
Frequency of dancing during leisure time	Physical activity	25043.00	-0.02	0.06	0.73	0.15	0.28	-	-
Frequency of shoveling snow during leisure time	Physical activity	27247.00	-0.08	0.06	0.20	0.00	0.39	-	-
Frequency of gardening during leisure time	Physical activity	27032.00	0.01	0.06	0.82	0.63	0.10	-	-
Frequency of hunting or fishing during leisure time	Physical activity	26329.00	0.06	0.06	0.37	0.00	0.54	-	-
Frequency of picking berries or mushrooms during leisure time	Physical activity	26898.00	-0.26	0.06	0.00	0.61	0.11	-	-
Changed everyday exercise during the last year	Physical activity	28710.00	0.18	0.05	0.00	0.74	0.05	-	-

Everyday exercise satisfaction	Physical activity	28627.00	0.23	0.06	0.00	0.02	0.31	-	-
Exercise during the last three months	Physical activity	28605.00	-0.19	0.06	0.00	0.00	0.68	-	-
If you exercise, change in exercise habits during the last year	Physical activity	24465.00	0.18	0.06	0.00	0.37	0.21	-	-
Bregott on bread	Food	25477.00	0.00	0.06	0.97	0.00	0.38	-	-
Whole grain crisp bread	Food	25477.00	0.15	0.06	0.01	0.00	0.68	-	-
Whole grain soft bread	Food	25477.00	-0.16	0.06	0.01	0.38	0.20	-	-
White (soft) bread, thin crisp bread	Food	25477.00	0.21	0.06	0.00	0.71	0.06	-	-
Coffee rolls/buns, rusk	Food	25477.00	0.02	0.06	0.78	0.00	0.77	-	-
Cheese 28%	Food	25477.00	-0.12	0.06	0.03	0.00	0.64	-	-
Cheese 10-17%	Food	25477.00	0.11	0.06	0.06	0.92	0.00	-	-
Soft cheese	Food	12795.00	0.06	0.08	0.43	0.00	0.37	-	-
Soft whey cheese	Food	12795.00	-0.19	0.08	0.03	0.00	0.53	-	-
Sausage, liver pate on bread	Food	25477.00	0.00	0.06	0.97	0.00	0.92	-	-
Meat on bread	Food	25477.00	0.23	0.06	0.00	0.00	0.58	-	-
Butter on bread	Food	25477.00	0.03	0.06	0.65	0.00	0.41	-	-
Oatflake, whole wheat, rye or barley porridge	Food	25477.00	-0.31	0.06	0.00	0.00	0.40	0.37	2.00
Rosehip, sweet syrup soup	Food	25477.00	0.03	0.06	0.57	0.00	0.74	-	-
Sour milk, yoghurt (3% fat)	Food	25477.00	-0.06	0.06	0.31	0.03	0.31	-	-
Sour milk, yoghurt (low fat)	Food	25477.00	-0.08	0.06	0.17	0.18	0.27	-	-
Fiber cereals	Food	25477.00	-0.19	0.06	0.00	0.00	0.37	-	-
Corn flakes	Food	25477.00	-0.03	0.06	0.61	0.00	0.58	-	-
Berries (fresh or frozen)	Food	25477.00	-0.03	0.06	0.57	0.00	0.63	-	-
Apple, pear, peach, orange, mandarin and grapefruit	Food	25477.00	-0.07	0.06	0.28	0.00	0.94	-	-
Ba	Food	25477.00	0.01	0.06	0.91	0.00	0.65	-	-
Root vegetables and carrot	Food	25477.00	-0.07	0.06	0.28	0.00	0.87	-	-
Low fat margarine on bread	Food	25477.00	0.07	0.06	0.21	0.00	0.78	-	-
Tomato and cucumber	Food	25477.00	-0.06	0.06	0.37	0.00	0.78	-	-
White cabbage, lettuce, lettuce cabbage, spich, borecole and broccoli	Food	25477.00	-0.06	0.06	0.31	0.00	0.39	-	-
Mixed frozen vegetables	Food	12795.00	-0.02	0.09	0.79	0.70	0.07	-	-
Boiled or baked potato	Food	25477.00	-0.02	0.06	0.72	0.00	0.90	-	-
Fried potatoes and pommes frites	Food	25477.00	0.11	0.06	0.06	0.00	0.94	-	-
Mashed potato	Food	12795.00	-0.01	0.09	0.93	0.00	0.72	-	-
Potato salad	Food	10427.00	0.11	0.09	0.22	0.00	0.62	-	-
Rice	Food	25477.00	-0.17	0.06	0.00	0.00	0.90	-	-
Pasta	Food	25477.00	-0.17	0.06	0.01	0.00	0.35	-	-
Brown beans and pea soup	Food	25477.00	0.02	0.06	0.74	0.39	0.20	-	-
Margarine on bread	Food	25477.00	0.06	0.06	0.26	0.00	0.88	-	-
Blota (broth + bread)	Food	12795.00	0.21	0.08	0.01	0.00	0.72	-	-
Pancake, waffle and Swedish dumpling	Food	25477.00	0.01	0.06	0.81	0.00	0.36	-	-
Pizza	Food	25477.00	-0.01	0.06	0.90	0.00	0.63	-	-
Minced meat dishes	Food	25477.00	0.02	0.06	0.76	0.55	0.13	-	-
Meat stew	Food	25477.00	-0.07	0.06	0.24	0.00	0.68	-	-
Steak, chop, etc.	Food	25477.00	0.08	0.06	0.20	0.00	0.63	-	-
Bacon	Food	25477.00	0.03	0.06	0.65	0.00	0.72	-	-
Sausage as main dish	Food	25477.00	-0.03	0.06	0.58	0.00	0.92	-	-
Hamburger	Food	25477.00	0.14	0.06	0.02	0.00	0.85	-	-
White meat (poultry)	Food	25477.00	-0.06	0.06	0.33	0.58	0.12	-	-
Butter for cooking	Food	25477.00	0.05	0.06	0.41	0.00	0.94	-	-
Blood based food	Food	12795.00	-0.08	0.08	0.36	0.00	0.49	-	-

Liver and kidney	Food	12795.00	-0.05	0.08	0.58	0.00	0.55	-	-
Lean fish (e.g. perch, bass, cod)	Food	25477.00	-0.05	0.06	0.41	0.00	0.69	-	-
Fatty fish (e.g. herring, whitefish, salmon)	Food	25477.00	-0.03	0.06	0.57	0.00	0.44	-	-
Shellfish (e.g. shrimps, scallops)	Food	12795.00	0.07	0.09	0.43	0.00	0.82	-	-
Salty fish	Food	25477.00	-0.03	0.06	0.64	0.00	0.87	-	-
Smoked fish/meat	Food	25477.00	0.11	0.06	0.05	0.00	0.40	-	-
Ice cream	Food	25477.00	-0.24	0.06	0.00	0.00	0.71	-	-
Sweets (chocolate and candy)	Food	25477.00	-0.02	0.06	0.69	0.00	0.47	-	-
Sugar, honey, marmelade and jam	Food	25477.00	-0.01	0.06	0.83	0.00	0.33	-	-
Margarine for cooking	Food	25477.00	0.06	0.06	0.31	0.00	0.80	-	-
Cookies and pastry	Food	25477.00	-0.12	0.06	0.03	0.00	0.69	-	-
Chips, popcorn and salted nuts	Food	25477.00	0.13	0.06	0.03	0.00	0.66	-	-
Low fat milk (0.5%)	Beverage	25477.00	-0.01	0.06	0.82	0.00	0.96	-	-
Milk, sour milk (1.5%)	Beverage	25477.00	0.07	0.06	0.24	0.60	0.12	-	-
Milk, sour milk (3%)	Beverage	25477.00	-0.06	0.06	0.27	0.00	0.72	-	-
Sodas, soft drinks and juice	Beverage	25477.00	0.08	0.06	0.16	0.00	0.66	-	-
Brewed (filtered) coffee	Beverage	25477.00	0.01	0.06	0.83	0.84	0.01	-	-
Boiled coffee	Beverage	25477.00	0.03	0.06	0.64	0.00	0.71	-	-
Tea	Beverage	25477.00	-0.06	0.06	0.28	0.47	0.17	-	-
Light beer	Alcohol	25477.00	-0.11	0.06	0.06	0.00	0.43	-	-
Oil for cooking	Food	25477.00	-0.09	0.06	0.12	0.00	0.80	-	-
Medium beer	Alcohol	25477.00	0.13	0.06	0.03	0.82	0.02	-	-
Strong beer	Alcohol	25477.00	0.09	0.06	0.14	0.33	0.22	-	-
Wine	Alcohol	25477.00	0.08	0.06	0.18	0.00	0.60	-	-
Liquor and spirits	Alcohol	25477.00	0.16	0.06	0.01	0.07	0.30	-	-
Salad dressing with oil	Food	25477.00	-0.03	0.06	0.64	0.00	0.64	-	-
Cream, creme fraiche, sour cream	Food	25477.00	0.02	0.06	0.74	0.00	0.47	-	-
Average portion size of vegetables based on photographic illustration of four sizes (smallest to largest)	Food	25477.00	-0.11	0.06	0.07	0.00	0.49	-	-
Overall state of health during the last year	General health	28674.00	-0.10	0.06	0.06	0.00	0.78	-	-
Overall state of health compared to others your age	General health	24938.00	-0.27	0.06	0.00	0.37	0.21	-	-
Parents or siblings had a cerebral hemorrhage/thrombosis or cardiac infarction before the age of 60	General health	28455.00	0.80	0.14	0.00	0.00	0.89	0.37	3.00
Teetotaler	Alcohol	28315.00	-0.50	0.19	0.01	0.00	0.63	-	-
Feel the need to reduce alcohol consumption	Alcohol	25612.00	0.17	0.19	0.37	0.00	0.99	-	-
Feel uneasy or guilty because of your way of drinking	Alcohol	25592.00	-0.10	0.18	0.57	0.74	0.05	-	-
Iron intake (mg/day)	Nutrients	25477.00	-0.01	0.06	0.81	0.00	0.64	-	-
Iodine intake (ug/day)	Nutrients	25477.00	-0.19	0.06	0.00	0.59	0.12	-	-
Calcium intake (mg/day)	Nutrients	25477.00	-0.05	0.06	0.42	0.17	0.27	-	-
Potassium intake (mg/day)	Nutrients	25477.00	-0.06	0.06	0.35	0.10	0.29	-	-
Beta-carotene intake (mg/day)	Nutrients	25477.00	-0.07	0.06	0.29	0.00	0.73	-	-
Cholesterol intake (g/day)	Nutrients	25477.00	0.00	0.06	0.99	0.00	0.99	-	-
Carbohydrates intake (g/day)	Nutrients	25477.00	-0.08	0.06	0.21	0.00	0.41	-	-

Average portion size of meat/fish based on photographic illustration of four sizes (smallest to largest)	Food	25477.00	-0.10	0.06	0.12	0.00	0.97	-	-
Breakfast habits: Only coffee/tea for breakfast vs not breakfast at all	Food	1576.00	1.75	0.80	0.03	0.00	0.85	-	-
Breakfast habits: Coffee/tea and wheat buns or rusk for breakfast vs not breakfast at all	Food	1963.00	-0.76	0.50	0.12	0.00	0.90	-	-
Breakfast habits: Porridge w/o sandwich for breakfast vs not breakfast at all	Food	3409.00	-1.53	0.35	0.00	0.00	0.90	-	-
Breakfast habits: Gruel w/o sandwich for breakfast vs not breakfast at all	Food	1847.00	-0.05	0.58	0.93	0.00	0.84	-	-
Eat breakfast from 2000	Food	2286.00	-1.92	0.59	0.00	0.36	0.21	-	-
Enterodiol intake (ug/day)	Nutrients	25477.00	-0.05	0.06	0.36	0.00	0.78	-	-
Enterolactone intake (ug/day)	Nutrients	25477.00	-0.08	0.06	0.17	0.00	0.39	-	-
Equol intake (ug/day)	Nutrients	25477.00	-0.05	0.06	0.38	0.00	0.56	-	-
Lariciresinol intake (ug/day)	Nutrients	25477.00	-0.10	0.06	0.12	0.00	0.74	-	-
Matairesinol intake (ug/day)	Nutrients	25477.00	-0.08	0.06	0.16	0.24	0.25	-	-
Medioresinol intake (ug/day)	Nutrients	25477.00	0.03	0.06	0.63	0.00	0.38	-	-
Pinoresinol intake (ug/day)	Nutrients	25477.00	-0.10	0.06	0.11	0.00	0.45	-	-
Secoisolariciresinol intake (ug/day)	Nutrients	25477.00	0.01	0.06	0.89	0.23	0.25	-	-
Sum of all ligns intake (ug/day)	Nutrients	25477.00	-0.03	0.06	0.65	0.00	0.42	-	-
Syringaresinol intake (ug/day)	Nutrients	25477.00	-0.01	0.06	0.87	0.00	0.35	-	-
Sum of Lariciresinol, Matairesinol, Pinoresinol, Secoisolariciresinol intake (ug/day)	Nutrients	25477.00	-0.09	0.06	0.14	0.00	0.67	-	-
Satisfaction with home and family situation	Psychosocial	13560.00	0.21	0.08	0.01	0.21	0.26	-	-
Appetite status	Psychosocial	13550.00	-0.05	0.08	0.49	0.00	1.00	-	-
Mood status	Psychosocial	13548.00	0.07	0.08	0.39	0.00	0.96	-	-
Energy status	Psychosocial	13536.00	0.14	0.08	0.07	0.00	0.81	-	-
Patience status	Psychosocial	13552.00	0.12	0.08	0.12	0.00	0.55	-	-
Confidence status	Psychosocial	13545.00	0.08	0.08	0.28	0.00	0.66	-	-
Sleep status	Sleep	13574.00	-0.05	0.08	0.55	0.00	0.46	-	-
Do you feel important and appreciated outside your home?	Psychosocial	13564.00	0.07	0.08	0.39	0.00	0.80	-	-
Do you feel important and appreciated in your home?	Psychosocial	13459.00	0.05	0.08	0.56	0.00	0.67	-	-
Satisfaction with accomodation	Psychosocial	13568.00	0.05	0.08	0.50	0.00	0.50	-	-
Satisfaction with work situation	Psychosocial	13427.00	-0.04	0.08	0.62	0.00	0.88	-	-
Satisfaction with economy	Psychosocial	13551.00	-0.06	0.08	0.44	0.00	0.34	-	-
Satisfaction with leisure time	Psychosocial	13540.00	0.16	0.08	0.04	0.00	0.50	-	-
Hearing status	General health	13565.00	0.04	0.08	0.66	0.00	0.39	-	-
Vision status	General health	13551.00	-0.02	0.08	0.79	0.26	0.24	-	-

Memory status	Psychosocial	13528.00	0.20	0.08	0.01	0.00	0.66	-	-
Fitness status	Physical activity	13542.00	-0.03	0.08	0.68	0.71	0.06	-	-
Magnesium intake (mg/day)	Nutrients	25477.00	-0.18	0.06	0.00	0.00	0.70	-	-
Saturated fat intake (g/day)	Nutrients	25477.00	0.03	0.06	0.59	0.00	0.49	-	-
Monounsaturated fat intake (g/day)	Nutrients	25477.00	0.01	0.06	0.87	0.00	0.84	-	-
Monosaccharides intake (g/day)	Nutrients	25477.00	-0.08	0.06	0.18	0.00	0.92	-	-
Sodium intake (mg/day)	Nutrients	25477.00	-0.07	0.06	0.21	0.01	0.31	-	-
Vitamin B3 intake (mg/day)	Nutrients	25477.00	0.00	0.06	0.99	0.78	0.03	-	-
Cambridge physical activity index	Physical activity	28041.00	-0.06	0.06	0.32	0.00	0.75	-	-
Polyunsaturated fat intake (g/day)	Nutrients	25477.00	-0.08	0.06	0.18	0.00	0.70	-	-
Average portion size of potatoes/rice/pasta based on photographic illustration of four sizes (smallest to largest)	Food	25477.00	-0.26	0.06	0.00	0.00	0.84	-	-
Total protein intake (g/day)	Nutrients	25477.00	0.00	0.06	0.96	0.00	0.65	-	-
Animal based protein intake (g/day)	Nutrients	25477.00	0.03	0.06	0.61	0.00	0.53	-	-
Plant based protein intake (g/day)	Nutrients	25477.00	-0.07	0.06	0.25	0.00	0.62	-	-
Vitamin A intake (mg/day)	Nutrients	25477.00	0.08	0.07	0.24	0.00	0.65	-	-
Sucrose intake (g/day)	Nutrients	25477.00	0.02	0.06	0.71	0.22	0.26	-	-
Cohabitation: Live alone vs Only one adult (spouse, partner)	Social	9144.00	0.11	0.23	0.63	0.00	0.71	-	-
Cohabitation: Live alone vs Only children	Social	3827.00	0.39	0.36	0.27	0.40	0.20	-	-
Cohabitation: Live alone vs Adult and children	Social	20030.00	0.08	0.20	0.67	0.00	0.41	-	-
Cohabitation: Live alone vs Other/others	Social	2947.00	-0.95	0.49	0.05	0.00	0.72	-	-
Selenium intake (ug/day)	Nutrients	25477.00	-0.05	0.06	0.41	0.00	0.67	-	-
Self rate of overall health	General health	352.00	0.21	0.47	0.66	0.00	0.53	-	-
For how much of the time during the last four weeks has your physical health or your emotiol problems limited your ability to interact with others?	General health	350.00	-0.35	0.46	0.45	0.00	0.78	-	-
Get sick more often than other people	General health	351.00	-0.26	0.46	0.58	0.28	0.24	-	-
As healthy as anyone	General health	352.00	-0.12	0.46	0.80	0.82	0.02	-	-
Worsen in health in the future	General health	348.00	0.47	0.46	0.31	0.00	0.32	-	-
Excellent health	General health	351.00	0.15	0.46	0.75	0.93	0.00	-	-
Self rate of overall health compared to a year ago	General health	351.00	-0.76	0.46	0.10	0.72	0.06	-	-
Physical limitation to participate in steneous activities: running, lifting heavy objects, taking part in physically demanding sports	General health	352.00	0.34	0.48	0.48	0.00	0.56	-	-

Physical limitation to participate in moderately demanding activities: moving a table, vacuuming, walking in the forest or gardening	General health	352.00	0.37	0.46	0.43	0.31	0.23	-	-
Physical limitation to participate in moderately demanding activities: lifting or carrying grocery bags	General health	351.00	0.75	0.47	0.11	0.00	0.61	-	-
Physical limitation to participate in moderately demanding activities: walking up several stairs	General health	350.00	-0.27	0.48	0.58	0.00	0.92	-	-
Physical limitation to participate in moderately demanding activities: bending down or kneeling	General health	351.00	0.19	0.47	0.69	0.00	0.54	-	-
Physical limitation to participate in moderately demanding activities: walking more than two km	General health	351.00	0.28	0.48	0.56	0.00	0.73	-	-
Physical limitation that reduced the normal time spent at work or in other activities during the last four weeks	General health	351.00	2.90	1.41	0.04	0.00	0.46	-	-
Physical limitation that made you do less than you wanted during the last four weeks	General health	350.00	2.15	1.13	0.06	0.00	0.52	-	-
Physical limitation that made you not being able to perform certain work tasks or other activities during the last four weeks	General health	348.00	3.30	1.18	0.01	0.00	0.68	-	-
Physical limitation that limited your ability to perform certain work tasks or other activities during the last four weeks	General health	349.00	2.56	1.32	0.05	0.30	0.23	-	-
Emotiol problems that made you do less than you wanted during the last four weeks	General health	351.00	-0.05	1.36	0.97	0.00	0.46	-	-
Extent to what your physical and emotiol health disrupted your usual social life during the last four weeks	General health	354.00	-0.13	0.46	0.78	0.35	0.21	-	-
Pain during the last four weeks	General health	353.00	0.81	0.46	0.08	0.00	0.45	-	-
How much has the pain during the last four weeks disturbed your normal work?	General health	352.00	0.37	0.46	0.42	0.00	0.75	-	-
For how much of the time during the last four weeks have you felt really alert and strong?	General health	354.00	-0.29	0.47	0.53	0.00	0.64	-	-
For how much of the time during the last four weeks have you felt very nervous?	General health	354.00	0.18	0.46	0.70	0.00	0.66	-	-

For how much of the time during the last four weeks have you felt so depressed that nothing could cheer you up?	General health	353.00	-0.19	0.46	0.68	0.00	0.95	-	-
For how much of the time during the last four weeks have you felt calm and serene?	General health	353.00	-0.08	0.46	0.85	0.00	0.90	-	-
For how much of the time during the last four weeks have you felt full of energy?	General health	352.00	-0.48	0.47	0.30	0.00	0.85	-	-
For how much of the time during the last four weeks have you felt gloomy and sad?	General health	351.00	-0.76	0.47	0.11	0.00	0.33	-	-
For how much of the time during the last four weeks have you felt worn out?	General health	350.00	0.59	0.48	0.22	0.19	0.27	-	-
For how much of the time during the last four weeks have you felt happy?	General health	354.00	-0.03	0.46	0.95	0.00	0.98	-	-
For how much of the time during the last four weeks have you felt tired?	General health	352.00	-0.22	0.47	0.65	0.00	0.87	-	-
Long-term sickness	General health	26146.00	0.02	0.18	0.91	0.00	0.86	-	-
Work shifts/weekends	Psychosocial	27060.00	0.20	0.13	0.11	0.00	0.40	-	-
Number of cigarretes smoked per day (in groups)	Tobacco use	23308.00	0.02	0.06	0.70	0.00	0.54	-	-
Years smoking	Tobacco use	25463.00	-0.05	0.06	0.41	0.00	0.36	-	-
Grams of tobacco smoked per week	Tobacco use	17841.00	-0.01	0.07	0.94	0.00	0.34	-	-
Number of cigarretes smoked per day	Tobacco use	18509.00	0.01	0.07	0.84	0.46	0.17	-	-
Number of cigars smoked per day	Tobacco use	17800.00	-0.10	0.07	0.16	0.00	0.55	-	-
Smoking status: Smokers vs non-smokers	Tobacco use	19029.00	-0.04	0.15	0.78	0.00	0.33	-	-
Smoking status: Former smokers vs non-smokers	Tobacco use	19792.00	-0.16	0.14	0.28	0.00	0.94	-	-
Smoking status: Former occasiol smokers vs non-smokers	Tobacco use	16539.00	-0.32	0.20	0.11	0.00	0.89	-	-
Number of snuff boxes per week	Tobacco use	27363.00	0.23	0.06	0.00	0.00	0.37	-	-
Snuff status: Snuff users vs non-snuff users	Tobacco use	24393.00	0.44	0.16	0.01	0.00	0.82	-	-
Snuff status: Former snuff users vs non-snuff users	Tobacco use	22516.00	-0.22	0.19	0.26	0.00	0.73	-	-
Years using snuff	Tobacco use	26603.00	0.15	0.06	0.02	0.00	0.36	-	-
Participation in associations or voluntary organisations	Social	28596.00	-0.26	0.12	0.02	0.00	0.40	-	-
Participation in sports or physical exercise associations	Social	1460.00	0.05	0.45	0.91	0.50	0.16	-	-
Participation in study circles	Social	1460.00	-0.30	0.63	0.64	0.00	0.46	-	-
Participation in other association	Social	1460.00	-0.57	0.45	0.20	0.48	0.16	-	-

People to ask for help apart from the ones at home	Social	28821.00	0.15	0.18	0.40	0.43	0.18	-	-
Number of friends that can come to your home at any time and feel at home	Social	28578.00	-0.05	0.05	0.35	0.00	0.90	-	-
Number of social contacts with the same interests as you	Social	28807.00	-0.11	0.05	0.05	0.30	0.23	-	-
Would you say that the number of people that you meet in your everyday life is enough or would you like to meet more or fewer people?	Social	28879.00	-0.14	0.05	0.01	0.00	0.89	-	-
Close relationship with anyone	Social	28640.00	-0.06	0.06	0.29	0.00	0.63	-	-
Frequency of engaging in clubs, associations or study circles	Social	19301.00	-0.13	0.07	0.05	0.00	0.77	-	-
Number of social interactions during a normal week	Social	28633.00	-0.13	0.05	0.02	0.00	0.85	-	-
Support from others	Social	28906.00	-0.11	0.06	0.05	0.00	0.72	-	-
Number of people with whom you can speak openly	Social	28878.00	-0.03	0.05	0.54	0.00	0.46	-	-
Receive hugs to comfort and support you	Social	28500.00	-0.23	0.15	0.13	0.00	0.37	-	-
Stigmasterol intake (mg/day)	Nutrients	25477.00	-0.04	0.06	0.53	0.15	0.28	-	-
Tiamin intake (mg/day)	Nutrients	25477.00	-0.01	0.06	0.91	0.69	0.07	-	-
Vitamin E intake (mg/day)	Nutrients	25477.00	0.06	0.06	0.33	0.00	0.87	-	-
Trans fat intake (g/day)	Nutrients	25477.00	-0.04	0.07	0.55	0.00	0.39	-	-
Sum of phytosterols intake (mg/day)	Nutrients	25477.00	-0.05	0.06	0.43	0.00	0.48	-	-
Educatiol level	Psychosocial	28864.00	-0.21	0.06	0.00	0.00	0.47	-	-
Zinc intake (mg/day)	Nutrients	25477.00	-0.10	0.06	0.10	0.00	0.59	-	-

Supplementary Table 15. Longitudinal association results for total cholesterol

Description	Group	N	Effect estimate	S.E.	p-value	I^2	Q p-value	adjusted R2	R2 rank
Alcohol intake (g/day)	Alcohol	22482.00	0.03	0.01	0.00	0.00	0.41	0.35	4.00
Permanent employment	Psychosocial	23867.00	0.06	0.01	0.00	0.00	0.64	0.33	11.00
Self-employed	Psychosocial	23867.00	-0.03	0.02	0.09	0.00	0.61	-	-
Distance to work in kilometers (one way)	Physical activity	21494.00	-0.02	0.01	0.01	0.84	0.01	-	-
Last time a colleague visited you at home	Psychosocial	23748.00	0.00	0.01	0.63	0.69	0.07	-	-
Job demands to work very fast	Psychosocial	24037.00	0.00	0.01	0.36	0.00	0.88	-	-
Frequency of social contacts with colleagues during leisure time	Psychosocial	23460.00	-0.01	0.01	0.26	0.00	0.38	-	-
High physical demand from job	Physical activity	24096.00	0.01	0.01	0.17	0.64	0.10	-	-
Enough time for job assignments	Psychosocial	23942.00	-0.01	0.01	0.07	0.00	0.77	-	-
Control over planning and execution of the workday	Psychosocial	24064.00	-0.02	0.01	0.00	0.00	0.98	-	-
Ingenuity or creativity demand from job	Psychosocial	23953.00	-0.01	0.01	0.14	0.00	0.73	-	-
Frequent social contacts with colleagues during work	Psychosocial	23840.00	0.00	0.01	0.45	0.68	0.07	-	-
Contradictory demands in job	Psychosocial	23871.00	0.01	0.01	0.29	0.00	0.47	-	-
Possibility to leave your work for a while to speak with a colleague	Psychosocial	23861.00	-0.01	0.01	0.31	0.42	0.19	-	-
Learn new things at job	Psychosocial	24007.00	0.00	0.01	0.59	0.00	0.78	-	-
High mental demand from job	Psychosocial	23873.00	-0.01	0.01	0.13	0.30	0.23	-	-
Repetitive job	Psychosocial	24037.00	-0.01	0.01	0.16	0.00	0.38	-	-
Skill demand from job	Psychosocial	23994.00	0.00	0.01	0.88	0.00	0.55	-	-
Possibility to speak with colleagues during breaks	Psychosocial	23991.00	0.01	0.01	0.12	0.00	0.54	-	-
Control over own work assignment	Psychosocial	24049.00	-0.01	0.01	0.04	0.00	0.92	-	-
Vitamin C intake (mg/day)	Nutrients	22482.00	-0.02	0.01	0.00	0.00	0.90	-	-
Vitamin B12 intake (ug/day)	Nutrients	22482.00	0.02	0.01	0.00	0.00	0.78	-	-
Vitamin B2 intake (ug/day)	Nutrients	22482.00	0.01	0.01	0.16	0.00	0.95	-	-
Vitamin B6 intake (mg/day)	Nutrients	22482.00	0.01	0.01	0.05	0.00	0.72	-	-
Informed of having high blood pressure	General health	24356.00	-0.02	0.02	0.30	0.00	0.70	-	-
Beta-sitostanol intake (mg/day)	Nutrients	22482.00	0.00	0.01	0.45	0.84	0.01	-	-
Beta-sitosterol intake (mg/day)	Nutrients	22482.00	0.00	0.01	0.83	0.29	0.24	-	-
Marital status: Single vs Married/partner	Social	22575.00	-0.01	0.02	0.63	0.00	0.40	-	-
Marital status: Single vs Divorced/separated	Social	3938.00	0.06	0.03	0.04	0.00	0.95	-	-
Marital status: Single vs Widow/widower	Social	2526.00	0.02	0.09	0.77	0.65	0.09	-	-
Campestanol intake (mg/day)	Nutrients	22482.00	0.00	0.01	0.71	0.84	0.01	-	-
Campesterol intake (mg/day)	Nutrients	22482.00	0.00	0.01	0.65	0.00	0.61	-	-

Parents or siblings have diabetes	General health	24152.00	-0.04	0.01	0.01	0.00	0.68	-	-
Disaccharides intake (g/day)	Nutrients	22482.00	-0.01	0.01	0.23	0.00	1.00	-	-
Vitamin D intake (ug/day)	Nutrients	22482.00	0.01	0.01	0.02	0.00	0.43	-	-
Total energy intake (kcal/day)	Nutrients	22482.00	-0.02	0.01	0.02	0.00	0.45	-	-
Formic acid intaje (g/day)	Nutrients	22482.00	0.00	0.01	0.72	0.50	0.16	-	-
Pentadecanoic acid intake (g/day)	Nutrients	22482.00	0.00	0.01	0.98	0.46	0.17	-	-
Palmitic acid intake (g/day)	Nutrients	22482.00	0.01	0.01	0.17	0.63	0.10	-	-
Heptadecanoic acid intake (g/day)	Nutrients	22482.00	0.00	0.01	0.98	0.46	0.17	-	-
Linoleic acid intake (g/day)	Nutrients	22482.00	0.01	0.01	0.10	0.00	0.91	-	-
Linolenic acid intake (g/day)	Nutrients	22482.00	0.01	0.01	0.17	0.00	0.99	-	-
Arachidonic acid (ARA) intake (g/day)	Nutrients	22482.00	0.02	0.01	0.00	0.00	0.35	-	-
Eicosapentaenoic acid (EPA) intake (g/day)	Nutrients	22482.00	0.01	0.01	0.06	0.00	0.77	-	-
Docosahexaenoic acid (DHA) intake (g/day)	Nutrients	22482.00	0.01	0.01	0.07	0.00	0.65	-	-
Fat intake (g/day)	Nutrients	22482.00	0.00	0.01	0.57	0.58	0.12	-	-
Fibre intake (g/day)	Nutrients	22482.00	-0.01	0.01	0.04	0.54	0.14	-	-
Folic acid intake (ug/day)	Nutrients	22482.00	0.01	0.01	0.36	0.00	0.39	-	-
Phosphate intake (mg/day)	Nutrients	22482.00	0.02	0.01	0.01	0.00	0.61	-	-
Whole grain intake (g/day)	Food	22482.00	0.01	0.01	0.14	0.16	0.27	-	-
Travel to work: Walk to work vs passive travel to work	Physical activity	15729.00	-0.02	0.02	0.46	0.00	0.38	-	-
Travel to work: Cycle to work vs passive travel to work	Physical activity	18929.00	0.01	0.01	0.41	0.63	0.10	-	-
Travel to work: Irregular travel mode to work vs passive travel to work	Physical activity	16025.00	0.00	0.02	0.96	0.00	0.80	-	-
Sedentary or standing work	Physical activity	23954.00	-0.01	0.01	0.49	0.00	0.42	-	-
Light but partly physically active work	Physical activity	23954.00	-0.01	0.01	0.56	0.00	0.38	-	-
Light and physically active work	Physical activity	23954.00	0.00	0.01	0.89	0.00	0.56	-	-
Sometimes physically straining work	Physical activity	23954.00	-0.01	0.01	0.22	0.00	0.47	-	-
Frequency of walking during leisure time	Physical activity	23716.00	-0.01	0.01	0.33	0.78	0.03	-	-
Frequency of cycling during leisure time	Physical activity	20892.00	0.00	0.01	0.95	0.00	0.72	-	-
Frequency of dancing during leisure time	Physical activity	21640.00	0.01	0.01	0.02	0.00	0.38	-	-
Frequency of shoveling snow during leisure time	Physical activity	23305.00	-0.02	0.01	0.00	0.00	0.75	-	-
Frequency of gardening during leisure time	Physical activity	23107.00	-0.02	0.01	0.00	0.00	0.55	-	-
Frequency of hunting or fishing during leisure time	Physical activity	22664.00	-0.01	0.01	0.20	0.00	0.54	-	-
Frequency of picking berries or mushrooms during leisure time	Physical activity	22989.00	-0.05	0.01	0.00	0.00	0.54	0.33	9.00

Changed everyday exercise during the last year	Physical activity	24350.00	0.01	0.01	0.25	0.00	0.76	-	-
Everyday exercise satisfaction	Physical activity	24282.00	0.01	0.01	0.22	0.00	0.97	-	-
Exercise during the last three months	Physical activity	24344.00	0.02	0.01	0.00	0.00	0.61	-	-
If you exercise, change in exercise habits during the last year	Physical activity	21315.00	0.01	0.01	0.03	0.12	0.29	-	-
Bregott on bread	Food	22482.00	0.00	0.01	0.43	0.42	0.19	-	-
Whole grain crisp bread	Food	22482.00	0.00	0.01	0.95	0.00	0.41	-	-
Whole grain soft bread	Food	22482.00	-0.01	0.01	0.08	0.77	0.04	-	-
White (soft) bread, thin crisp bread	Food	22482.00	0.01	0.01	0.05	0.68	0.08	-	-
Coffee rolls/buns, rusk	Food	22482.00	-0.02	0.01	0.00	0.00	0.41	-	-
Cheese 28%	Food	22482.00	0.00	0.01	0.88	0.00	0.49	-	-
Cheese 10-17%	Food	22482.00	0.00	0.01	0.76	0.00	0.95	-	-
Soft cheese	Food	10074.00	0.01	0.01	0.37	0.00	0.53	-	-
Soft whey cheese	Food	10074.00	-0.02	0.01	0.00	0.59	0.12	-	-
Sausage, liver pate on bread	Food	22482.00	-0.01	0.01	0.24	0.61	0.11	-	-
Meat on bread	Food	22482.00	0.01	0.01	0.02	0.00	0.98	-	-
Butter on bread	Food	22482.00	0.00	0.01	0.60	0.00	0.80	-	-
Oatflake, whole wheat, rye or barley porridge	Food	22482.00	0.00	0.01	0.50	0.00	0.72	-	-
Rosehip, sweet syrup soup	Food	22482.00	0.00	0.01	0.57	0.00	0.68	-	-
Sour milk, yoghurt (3% fat)	Food	22482.00	-0.01	0.01	0.04	0.00	0.38	-	-
Sour milk, yoghurt (low fat)	Food	22482.00	0.01	0.01	0.19	0.00	0.99	-	-
Fiber cereals	Food	22482.00	0.00	0.01	0.66	0.00	0.69	-	-
Corn flakes	Food	22482.00	-0.01	0.01	0.08	0.00	0.70	-	-
Berries (fresh or frozen)	Food	22482.00	-0.01	0.01	0.09	0.00	0.36	-	-
Apple, pear, peach, orange, mandarin and grapefruit	Food	22482.00	0.00	0.01	0.70	0.00	0.54	-	-
Ba	Food	22482.00	0.01	0.01	0.09	0.00	0.70	-	-
Root vegetables and carrot	Food	22482.00	0.00	0.01	0.84	0.61	0.11	-	-
Low fat margarine on bread	Food	22482.00	0.01	0.01	0.02	0.56	0.13	-	-
Tomato and cucumber	Food	22482.00	0.00	0.01	0.39	0.00	0.93	-	-
White cabbage, lettuce, lettuce cabbage, spich, borecole and broccoli	Food	22482.00	0.01	0.01	0.34	0.00	0.53	-	-
Mixed frozen vegetables	Food	10074.00	-0.01	0.01	0.33	0.00	0.86	-	-
Boiled or baked potato	Food	22482.00	-0.02	0.01	0.01	0.00	0.70	-	-
Fried potatoes and pommes frites	Food	22482.00	0.01	0.01	0.01	0.00	0.35	-	-
Mashed potato	Food	10074.00	0.00	0.01	0.96	0.74	0.05	-	-
Potato salad	Food	10074.00	0.01	0.01	0.56	0.50	0.16	-	-
Rice	Food	22482.00	0.00	0.01	0.39	0.36	0.21	-	-
Pasta	Food	22482.00	0.02	0.01	0.00	0.77	0.04	-	-
Brown beans and pea soup	Food	22482.00	-0.01	0.01	0.09	0.00	0.57	-	-
Margarine on bread	Food	22482.00	0.00	0.01	0.65	0.00	0.85	-	-
Blota (broth + bread)	Food	10074.00	0.00	0.01	0.84	0.00	0.99	-	-
Pancake, waffle and Swedish dumpling	Food	22482.00	0.01	0.01	0.31	0.00	0.41	-	-
Pizza	Food	22482.00	0.01	0.01	0.20	0.30	0.23	-	-
Minced meat dishes	Food	22482.00	0.01	0.01	0.04	0.89	0.00	-	-
Meat stew	Food	22482.00	0.00	0.01	0.44	0.30	0.23	-	-
Steak, chop, etc.	Food	22482.00	0.00	0.01	0.50	0.88	0.00	-	-
Bacon	Food	22482.00	0.01	0.01	0.03	0.00	0.72	-	-
Sausage as main dish	Food	22482.00	0.01	0.01	0.08	0.00	0.83	-	-

Hamburger	Food	22482.00	0.01	0.01	0.02	0.00	0.96	-	-
White meat (poultry)	Food	22482.00	0.02	0.01	0.01	0.00	0.82	-	-
Butter for cooking	Food	22482.00	0.01	0.01	0.03	0.56	0.13	-	-
Blood based food	Food	10074.00	0.00	0.01	0.97	0.00	0.92	-	-
Liver and kidney	Food	10074.00	0.01	0.01	0.49	0.00	0.87	-	-
Lean fish (e.g. perch, bass, cod)	Food	22482.00	0.00	0.01	0.79	0.84	0.01	-	-
Fatty fish (e.g. herring, whitefish, salmon)	Food	22482.00	0.01	0.01	0.29	0.64	0.09	-	-
Shellfish (e.g. shrimps, scallops)	Food	10074.00	0.03	0.01	0.00	0.00	0.63	-	-
Salty fish	Food	22482.00	0.01	0.01	0.07	0.20	0.26	-	-
Smoked fish/meat	Food	22482.00	0.00	0.01	0.63	0.00	0.92	-	-
Ice cream	Food	22482.00	-0.02	0.01	0.00	0.00	0.91	-	-
Sweets (chocolate and candy)	Food	22482.00	-0.01	0.01	0.07	0.00	0.96	-	-
Sugar, honey, marmelade and jam	Food	22482.00	0.00	0.01	0.94	0.20	0.26	-	-
Margarine for cooking	Food	22482.00	-0.02	0.01	0.01	0.00	0.96	-	-
Cookies and pastry	Food	22482.00	0.00	0.01	0.38	0.00	0.32	-	-
Chips, popcorn and salted nuts	Food	22482.00	0.03	0.01	0.00	0.00	0.99	0.35	3.00
Low fat milk (0.5%)	Beverage	22482.00	0.00	0.01	0.64	0.00	0.99	-	-
Milk, sour milk (1.5%)	Beverage	22482.00	0.01	0.01	0.04	0.00	0.90	-	-
Milk, sour milk (3%)	Beverage	22482.00	0.00	0.01	0.39	0.00	0.47	-	-
Sodas, soft drinks and juice	Beverage	22482.00	-0.01	0.01	0.02	0.52	0.15	-	-
Brewed (filtered) coffee	Beverage	22482.00	0.02	0.01	0.00	0.00	0.87	0.35	5.00
Boiled coffee	Beverage	22482.00	0.01	0.01	0.08	0.69	0.07	-	-
Tea	Beverage	22482.00	-0.02	0.01	0.00	0.78	0.03	-	-
Light beer	Alcohol	22482.00	0.00	0.01	0.40	0.00	0.80	-	-
Oil for cooking	Food	22482.00	0.01	0.01	0.07	0.00	0.44	-	-
Medium beer	Alcohol	22482.00	0.02	0.01	0.00	0.00	0.90	-	-
Strong beer	Alcohol	22482.00	0.04	0.01	0.00	0.30	0.23	0.35	1.00
Wine	Alcohol	22482.00	0.01	0.01	0.01	0.00	0.40	-	-
Liquor and spirits	Alcohol	22482.00	0.02	0.01	0.00	0.00	0.36	-	-
Salad dressing with oil	Food	22482.00	0.01	0.01	0.33	0.53	0.14	-	-
Cream, creme fraiche, sour cream	Food	22482.00	0.01	0.01	0.05	0.00	0.96	-	-
Average portion size of vegetables based on photographic illustration of four sizes (smallest to largest)	Food	22482.00	-0.01	0.01	0.18	0.35	0.21	-	-
Overall state of health during the last year	General health	24330.00	-0.02	0.01	0.00	0.11	0.29	-	-
Overall state of health compared to others your age	General health	23994.00	0.00	0.01	0.88	0.89	0.00	-	-
Parents or siblings had a cerebral hemorrhage/thrombosis or cardiac infarction before the age of 60	General health	24110.00	0.04	0.01	0.01	0.00	0.56	-	-
Teetotaler	Alcohol	24319.00	-0.13	0.02	0.00	0.09	0.30	0.33	10.00
Feel the need to reduce alcohol consumption	Alcohol	22084.00	0.07	0.02	0.00	0.00	0.39	-	-
Feel uneasy or guilty because of your way of drinking	Alcohol	22073.00	0.07	0.02	0.00	0.26	0.25	-	-
Iron intake (mg/day)	Nutrients	22482.00	0.00	0.01	0.59	0.00	0.38	-	-
Iodine intake (ug/day)	Nutrients	22482.00	0.02	0.01	0.00	0.00	0.96	-	-
Calcium intake (mg/day)	Nutrients	22482.00	0.00	0.01	0.98	0.00	0.85	-	-
Potassium intake (mg/day)	Nutrients	22482.00	0.01	0.01	0.40	0.00	0.70	-	-
Beta-carotene intake (mg/day)	Nutrients	22482.00	0.00	0.01	0.91	0.58	0.12	-	-

Cholesterol intake (g/day)	Nutrients	22482.00	0.01	0.01	0.05	0.78	0.03	-	-
Carbohydrates intake (g/day)	Nutrients	22482.00	-0.02	0.01	0.00	0.58	0.12	-	-
Average portion size of meat/fish based on photographic illustration of four sizes (smallest to largest)	Food	22482.00	0.01	0.01	0.21	0.00	0.67	-	-
Breakfast habits: Only coffee/tea for breakfast vs not breakfast at all	Food	1533.00	0.47	0.08	0.00	0.57	0.13	0.30	15.00
Breakfast habits: Coffee/tea and wheat buns or rusk for breakfast vs not breakfast at all	Food	1903.00	-0.09	0.05	0.07	0.34	0.22	-	-
Breakfast habits: Porridge w/o sandwich for breakfast vs not breakfast at all	Food	3293.00	-0.02	0.03	0.48	0.30	0.23	-	-
Breakfast habits: Gruel w/o sandwich for breakfast vs not breakfast at all	Food	1806.00	-0.03	0.06	0.63	0.00	0.89	-	-
Eat breakfast from 2000	Food	2257.00	-0.19	0.06	0.00	0.00	0.53	-	-
Enterodiol intake (ug/day)	Nutrients	22482.00	-0.01	0.01	0.35	0.00	0.65	-	-
Enterolactone intake (ug/day)	Nutrients	22482.00	-0.01	0.01	0.05	0.00	0.37	-	-
Equol intake (ug/day)	Nutrients	22482.00	0.00	0.01	0.77	0.00	1.00	-	-
Lariciresinol intake (ug/day)	Nutrients	22482.00	-0.01	0.01	0.25	0.66	0.09	-	-
Matairesinol intake (ug/day)	Nutrients	22482.00	-0.01	0.01	0.24	0.83	0.02	-	-
Medioresinol intake (ug/day)	Nutrients	22482.00	-0.01	0.01	0.34	0.81	0.02	-	-
Pinoresinol intake (ug/day)	Nutrients	22482.00	-0.01	0.01	0.04	0.77	0.04	-	-
Secoisolariciresinol intake (ug/day)	Nutrients	22482.00	0.02	0.01	0.00	0.00	0.86	-	-
Sum of all ligns intake (ug/day)	Nutrients	22482.00	-0.01	0.01	0.29	0.81	0.02	-	-
Syringaresinol intake (ug/day)	Nutrients	22482.00	-0.01	0.01	0.35	0.82	0.02	-	-
Sum of Lariciresinol, Matairesinol, Pinoresinol, Secoisolariciresinol intake (ug/day)	Nutrients	22482.00	-0.01	0.01	0.25	0.69	0.07	-	-
Satisfaction with home and family situation	Psychosocial	13269.00	-0.02	0.01	0.00	0.00	0.84	-	-
Appetite status	Psychosocial	13258.00	-0.02	0.01	0.00	0.00	0.48	-	-
Mood status	Psychosocial	13257.00	-0.01	0.01	0.13	0.00	0.74	-	-
Energy status	Psychosocial	13246.00	-0.01	0.01	0.18	0.00	0.90	-	-
Patience status	Psychosocial	13260.00	-0.01	0.01	0.06	0.00	0.50	-	-
Confidence status	Psychosocial	13255.00	-0.01	0.01	0.08	0.00	0.77	-	-
Sleep status	Sleep	13284.00	-0.02	0.01	0.02	0.00	0.73	-	-
Do you feel important and appreciated outside your home?	Psychosocial	13276.00	-0.01	0.01	0.41	0.00	0.86	-	-
Do you feel important and appreciated in your home?	Psychosocial	13175.00	-0.01	0.01	0.10	0.00	0.36	-	-
Satisfaction with accomodation	Psychosocial	13277.00	-0.01	0.01	0.10	0.00	0.86	-	-
Satisfaction with work situation	Psychosocial	13139.00	-0.01	0.01	0.38	0.00	0.70	-	-
Satisfaction with economy	Psychosocial	13260.00	-0.01	0.01	0.20	0.00	0.52	-	-

Satisfaction with leisure time	Psychosocial	13252.00	-0.01	0.01	0.13	0.00	0.81	-	-
Hearing status	General health	13275.00	0.00	0.01	0.52	0.00	0.59	-	-
Vision status	General health	13261.00	-0.01	0.01	0.16	0.00	0.94	-	-
Memory status	Psychosocial	13238.00	-0.01	0.01	0.17	0.00	0.95	-	-
Fitness status	Physical activity	13252.00	0.01	0.01	0.16	0.00	0.80	-	-
Magnesium intake (mg/day)	Nutrients	22482.00	0.01	0.01	0.01	0.50	0.16	-	-
Saturated fat intake (g/day)	Nutrients	22482.00	0.00	0.01	0.95	0.66	0.09	-	-
Monounsaturated fat intake (g/day)	Nutrients	22482.00	0.04	0.01	0.00	0.00	0.76	0.35	2.00
Monosaccharides intake (g/day)	Nutrients	22482.00	-0.01	0.01	0.07	0.00	0.49	-	-
Sodium intake (mg/day)	Nutrients	22482.00	0.02	0.01	0.00	0.68	0.08	-	-
Vitamin B3 intake (mg/day)	Nutrients	22482.00	0.02	0.01	0.00	0.00	0.77	-	-
Cambridge physical activity index	Physical activity	23920.00	0.02	0.01	0.00	0.00	0.64	-	-
Polyunsaturated fat intake (g/day)	Nutrients	22482.00	0.01	0.01	0.23	0.00	0.60	-	-
Average portion size of potatoes/rice/pasta based on photographic illustration of four sizes (smallest to largest)	Food	22482.00	0.01	0.01	0.04	0.26	0.25	-	-
Total protein intake (g/day)	Nutrients	22482.00	0.01	0.01	0.04	0.00	0.36	-	-
Animal based protein intake (g/day)	Nutrients	22482.00	0.02	0.01	0.00	0.52	0.15	-	-
Plant based protein intake (g/day)	Nutrients	22482.00	-0.01	0.01	0.02	0.55	0.14	-	-
Vitamin A intake (mg/day)	Nutrients	22482.00	0.01	0.01	0.21	0.00	0.79	-	-
Sucrose intake (g/day)	Nutrients	22482.00	-0.02	0.01	0.00	0.00	0.62	-	-
Cohabitation: Live alone vs Only one adult (spouse, partner)	Social	7411.00	-0.05	0.02	0.02	0.42	0.19	-	-
Cohabitation: Live alone vs Only children	Social	3320.00	0.02	0.04	0.55	0.57	0.13	-	-
Cohabitation: Live alone vs Adult and children	Social	17394.00	-0.03	0.02	0.13	0.87	0.01	-	-
Cohabitation: Live alone vs Other/others	Social	2485.00	-0.15	0.05	0.00	0.00	0.47	-	-
Selenium intake (ug/day)	Nutrients	22482.00	0.01	0.01	0.05	0.00	0.91	-	-
Long-term sickness	General health	23500.00	0.00	0.02	0.95	0.58	0.12	-	-
Work shifts/weekends	Psychosocial	23601.00	0.02	0.01	0.05	0.04	0.31	-	-
Number of cigarretes smoked per day (in groups)	Tobacco use	20415.00	-0.01	0.01	0.10	0.36	0.21	-	-
Years smoking	Tobacco use	21520.00	0.00	0.01	0.55	0.13	0.28	-	-
Grams of tobacco smoked per week	Tobacco use	15034.00	0.11	0.01	0.00	0.00	0.98	0.33	8.00
Number of cigarretes smoked per day	Tobacco use	15034.00	0.11	0.01	0.00	0.00	0.98	0.33	6.00
Number of cigars smoked per day	Tobacco use	15034.00	0.11	0.01	0.00	0.00	0.98	0.33	7.00
Smoking status: Smokers vs non-smokers	Tobacco use	16010.00	0.00	0.02	0.75	0.82	0.02	-	-
Smoking status: Former smokers vs non-smokers	Tobacco use	16768.00	-0.02	0.01	0.16	0.00	0.34	-	-
Smoking status: Former occasiol smokers vs non-smokers	Tobacco use	14010.00	0.02	0.02	0.23	0.10	0.29	-	-

Number of snuff boxes per week	Tobacco use	23047.00	0.05	0.01	0.00	0.61	0.11	0.32	12.00
Snuff status: Snuff users vs non-snuff users	Tobacco use	20554.00	0.14	0.02	0.00	0.76	0.04	0.32	14.00
Snuff status: Former snuff users vs non-snuff users	Tobacco use	18810.00	0.02	0.02	0.25	0.52	0.15	-	-
Years using snuff	Tobacco use	22411.00	0.05	0.01	0.00	0.82	0.02	0.32	13.00
Participation in associations or voluntary organisations	Social	24272.00	-0.01	0.01	0.49	0.46	0.17	-	-
Participation in sports or physical exercise associations	Social	1439.00	0.04	0.04	0.41	0.00	0.43	-	-
Participation in study circles	Social	1439.00	-0.14	0.06	0.03	0.00	0.89	-	-
Participation in other association	Social	1439.00	-0.01	0.04	0.81	0.00	0.47	-	-
People to ask for help apart from the ones at home	Social	24261.00	0.02	0.02	0.29	0.68	0.08	-	-
Number of friends that can come to your home at any time and feel at home	Social	24253.00	0.00	0.01	0.45	0.71	0.06	-	-
Number of social contacts with the same interests as you	Social	24252.00	-0.01	0.01	0.05	0.59	0.12	-	-
Would you say that the number of people that you meet in your everyday life is enough or would you like to meet more or fewer people?	Social	24285.00	0.00	0.01	0.73	0.00	0.63	-	-
Close relationship with anyone	Social	24313.00	-0.01	0.01	0.16	0.83	0.02	-	-
Frequency of engaging in clubs, associations or study circles	Social	16258.00	0.01	0.01	0.40	0.72	0.06	-	-
Number of social interactions during a normal week	Social	24306.00	0.00	0.01	0.50	0.80	0.02	-	-
Support from others	Social	24307.00	-0.01	0.01	0.12	0.00	0.91	-	-
Number of people with whom you can speak openly	Social	24290.00	-0.01	0.01	0.28	0.83	0.02	-	-
Receive hugs to comfort and support you	Social	24197.00	0.01	0.01	0.55	0.04	0.31	-	-
Stigmasterol intake (mg/day)	Nutrients	22482.00	0.00	0.01	0.78	0.00	0.47	-	-
Tiamin intake (mg/day)	Nutrients	22482.00	0.01	0.01	0.03	0.00	0.78	-	-
Vitamin E intake (mg/day)	Nutrients	22482.00	0.00	0.01	0.55	0.00	0.74	-	-
Trans fat intake (g/day)	Nutrients	22482.00	0.00	0.01	0.64	0.00	0.99	-	-
Sum of phytosterols intake (mg/day)	Nutrients	22482.00	0.00	0.01	0.98	0.32	0.22	-	-
Educatiol level	Psychosocial	24335.00	0.02	0.01	0.00	0.37	0.21	-	-
Zinc intake (mg/day)	Nutrients	22482.00	-0.01	0.01	0.04	0.00	0.61	-	-

Supplementary Table 16. Longitudinal association results for triglycerides

Description	Group	N	Effect estimate	S.E.	p-value	I^2	Q p-value	adjusted R2	R2 rank
Alcohol intake (g/day)	Alcohol	18149.00	0.02	0.01	0.00	0.00	0.91	-	-
Permanent employment	Psychosocial	19251.00	0.01	0.01	0.22	0.87	0.01	-	-
Self-employed	Psychosocial	19251.00	-0.03	0.02	0.09	0.00	0.46	-	-
Distance to work in kilometers (one way)	Physical activity	17212.00	0.00	0.01	0.58	0.00	0.55	-	-
Last time a colleague visited you at home	Psychosocial	19141.00	0.00	0.00	0.61	0.00	0.83	-	-
Job demands to work very fast	Psychosocial	19397.00	0.00	0.00	0.66	0.00	0.95	-	-
Frequency of social contacts with colleagues during leisure time	Psychosocial	18915.00	0.00	0.00	0.73	0.00	0.48	-	-
High physical demand from job	Physical activity	19447.00	0.01	0.00	0.06	0.69	0.07	-	-
Enough time for job assignments	Psychosocial	19320.00	-0.01	0.00	0.03	0.00	0.52	-	-
Control over planning and execution of the workday	Psychosocial	19416.00	-0.01	0.00	0.06	0.40	0.20	-	-
Ingenuity or creativity demand from job	Psychosocial	19325.00	-0.01	0.00	0.10	0.00	1.00	-	-
Frequent social contacts with colleagues during work	Psychosocial	19223.00	0.00	0.00	0.66	0.66	0.09	-	-
Contradictory demands in job	Psychosocial	19247.00	0.00	0.00	0.51	0.00	0.93	-	-
Possibility to leave your work for a while to speak with a colleague	Psychosocial	19240.00	0.00	0.00	0.40	0.22	0.26	-	-
Learn new things at job	Psychosocial	19377.00	-0.01	0.00	0.05	0.00	0.64	-	-
High mental demand from job	Psychosocial	19257.00	0.00	0.00	0.89	0.00	0.80	-	-
Repetitive job	Psychosocial	19400.00	0.00	0.00	0.54	0.00	0.36	-	-
Skill demand from job	Psychosocial	19360.00	-0.02	0.00	0.00	0.00	0.45	-	-
Possibility to speak with colleagues during breaks	Psychosocial	19355.00	0.00	0.00	0.90	0.00	0.62	-	-
Control over own work assignment	Psychosocial	19407.00	-0.01	0.00	0.18	0.03	0.31	-	-
Vitamin C intake (mg/day)	Nutrients	18149.00	0.00	0.01	0.75	0.06	0.30	-	-
Vitamin B12 intake (ug/day)	Nutrients	18149.00	0.01	0.01	0.04	0.00	0.36	-	-
Vitamin B2 intake (ug/day)	Nutrients	18149.00	-0.01	0.01	0.12	0.42	0.19	-	-
Vitamin B6 intake (mg/day)	Nutrients	18149.00	0.00	0.01	0.78	0.82	0.02	-	-
Informed of having high blood pressure	General health	19669.00	0.05	0.01	0.00	0.39	0.20	-	-
Beta-sitostanol intake (mg/day)	Nutrients	18149.00	0.00	0.01	0.62	0.00	0.42	-	-
Beta-sitosterol intake (mg/day)	Nutrients	18149.00	-0.01	0.01	0.01	0.00	0.52	-	-
Marital status: Single vs Married/partner	Social	18207.00	-0.02	0.02	0.35	0.00	0.58	-	-
Marital status: Single vs Divorced/separated	Social	3129.00	0.07	0.03	0.03	0.00	0.67	-	-
Marital status: Single vs Widow/widower	Social	1986.00	0.09	0.08	0.25	0.00	0.40	-	-
Campestanol intake (mg/day)	Nutrients	18149.00	0.00	0.01	0.90	0.52	0.15	-	-
Campesterol intake (mg/day)	Nutrients	18149.00	-0.01	0.01	0.14	0.40	0.20	-	-

Parents or siblings have diabetes	General health	19492.00	0.05	0.01	0.00	0.00	0.55	-	-
Disaccharides intake (g/day)	Nutrients	18149.00	0.00	0.01	0.87	0.00	0.69	-	-
Vitamin D intake (ug/day)	Nutrients	18149.00	0.01	0.01	0.22	0.74	0.05	-	-
Total energy intake (kcal/day)	Nutrients	18149.00	-0.02	0.01	0.00	0.00	0.82	-	-
Formic acid intaje (g/day)	Nutrients	18149.00	0.00	0.01	0.56	0.00	0.42	-	-
Pentadecanoic acid intake (g/day)	Nutrients	18149.00	0.00	0.01	0.44	0.48	0.17	-	-
Palmitic acid intake (g/day)	Nutrients	18149.00	0.01	0.01	0.13	0.00	0.78	-	-
Heptadecanoic acid intake (g/day)	Nutrients	18149.00	0.00	0.01	0.44	0.48	0.17	-	-
Linoleic acid intake (g/day)	Nutrients	18149.00	-0.01	0.00	0.12	0.02	0.31	-	-
Linolenic acid intake (g/day)	Nutrients	18149.00	0.00	0.01	0.40	0.58	0.12	-	-
Arachidonic acid (ARA) intake (g/day)	Nutrients	18149.00	0.01	0.01	0.04	0.00	0.57	-	-
Eicosapentaenoic acid (EPA) intake (g/day)	Nutrients	18149.00	0.00	0.01	0.35	0.40	0.20	-	-
Docosahexaenoic acid (DHA) intake (g/day)	Nutrients	18149.00	0.00	0.01	0.37	0.53	0.15	-	-
Fat intake (g/day)	Nutrients	18149.00	0.00	0.01	0.38	0.00	0.83	-	-
Fibre intake (g/day)	Nutrients	18149.00	-0.02	0.01	0.00	0.00	0.62	-	-
Folic acid intake (ug/day)	Nutrients	18149.00	-0.01	0.01	0.06	0.00	0.50	-	-
Phosphate intake (mg/day)	Nutrients	18149.00	-0.01	0.01	0.29	0.00	0.61	-	-
Whole grain intake (g/day)	Food	18149.00	-0.01	0.01	0.24	0.37	0.21	-	-
Travel to work: Walk to work vs passive travel to work	Physical activity	12793.00	-0.03	0.02	0.18	0.00	1.00	-	-
Travel to work: Cycle to work vs passive travel to work	Physical activity	15168.00	-0.03	0.01	0.02	0.00	0.78	-	-
Travel to work: Irregular travel mode to work vs passive travel to work	Physical activity	12993.00	0.02	0.02	0.33	0.00	0.45	-	-
Sedentary or standing work	Physical activity	19318.00	0.00	0.01	0.84	0.72	0.06	-	-
Light but partly physically active work	Physical activity	19318.00	-0.01	0.01	0.58	0.00	0.54	-	-
Light and physically active work	Physical activity	19318.00	0.00	0.01	0.73	0.00	0.67	-	-
Sometimes physically straining work	Physical activity	19318.00	0.00	0.01	0.73	0.31	0.23	-	-
Frequency of walking during leisure time	Physical activity	19152.00	0.00	0.01	0.41	0.00	0.85	-	-
Frequency of cycling during leisure time	Physical activity	16720.00	-0.01	0.01	0.18	0.00	0.44	-	-
Frequency of dancing during leisure time	Physical activity	17418.00	-0.01	0.01	0.32	0.82	0.02	-	-
Frequency of shoveling snow during leisure time	Physical activity	18789.00	-0.02	0.01	0.00	0.00	0.92	-	-
Frequency of gardening during leisure time	Physical activity	18634.00	-0.01	0.01	0.05	0.00	0.85	-	-
Frequency of hunting or fishing during leisure time	Physical activity	18282.00	0.00	0.01	0.59	0.62	0.11	-	-
Frequency of picking berries or mushrooms during leisure time	Physical activity	18524.00	-0.02	0.01	0.00	0.69	0.07	-	-

Changed everyday exercise during the last year	Physical activity	19656.00	0.00	0.00	0.48	0.00	0.37	-	-
Everyday exercise satisfaction	Physical activity	19596.00	0.00	0.00	0.80	0.00	0.84	-	-
Exercise during the last three months	Physical activity	19648.00	-0.01	0.00	0.02	0.60	0.11	-	-
If you exercise, change in exercise habits during the last year	Physical activity	17173.00	0.00	0.01	0.63	0.17	0.27	-	-
Bregott on bread	Food	18149.00	0.00	0.01	0.48	0.00	0.55	-	-
Whole grain crisp bread	Food	18149.00	0.00	0.01	0.34	0.00	0.96	-	-
Whole grain soft bread	Food	18149.00	0.00	0.01	0.53	0.00	0.39	-	-
White (soft) bread, thin crisp bread	Food	18149.00	0.02	0.01	0.00	0.60	0.11	-	-
Coffee rolls/buns, rusk	Food	18149.00	-0.01	0.01	0.01	0.00	0.91	-	-
Cheese 28%	Food	18149.00	-0.01	0.01	0.12	0.00	0.77	-	-
Cheese 10-17%	Food	18149.00	0.00	0.01	0.95	0.39	0.20	-	-
Soft cheese	Food	7782.00	0.01	0.01	0.45	0.00	0.61	-	-
Soft whey cheese	Food	7782.00	-0.01	0.01	0.31	0.00	0.37	-	-
Sausage, liver pate on bread	Food	18149.00	0.01	0.01	0.04	0.00	0.90	-	-
Meat on bread	Food	18149.00	0.02	0.01	0.00	0.29	0.23	-	-
Butter on bread	Food	18149.00	0.01	0.00	0.10	0.76	0.04	-	-
Oatflake, whole wheat, rye or barley porridge	Food	18149.00	-0.01	0.01	0.10	0.08	0.30	-	-
Rosehip, sweet syrup soup	Food	18149.00	0.01	0.00	0.02	0.59	0.12	-	-
Sour milk, yoghurt (3% fat)	Food	18149.00	-0.02	0.01	0.00	0.00	0.53	-	-
Sour milk, yoghurt (low fat)	Food	18149.00	-0.01	0.00	0.03	0.00	0.66	-	-
Fiber cereals	Food	18149.00	-0.03	0.01	0.00	0.00	0.60	0.267683564	1
Corn flakes	Food	18149.00	-0.01	0.01	0.02	0.18	0.27	-	-
Berries (fresh or frozen)	Food	18149.00	-0.01	0.00	0.01	0.00	0.78	-	-
Apple, pear, peach, orange, mandarin and grapefruit	Food	18149.00	-0.01	0.01	0.04	0.00	0.34	-	-
Ba	Food	18149.00	-0.01	0.01	0.20	0.38	0.21	-	-
Root vegetables and carrot	Food	18149.00	-0.01	0.01	0.03	0.00	0.85	-	-
Low fat margarine on bread	Food	18149.00	0.00	0.01	0.38	0.00	0.60	-	-
Tomato and cucumber	Food	18149.00	0.00	0.01	0.85	0.05	0.30	-	-
White cabbage, lettuce, lettuce cabbage, spich, borecole and broccoli	Food	18149.00	0.00	0.01	0.38	0.49	0.16	-	-
Mixed frozen vegetables	Food	7782.00	0.00	0.01	0.73	0.00	0.41	-	-
Boiled or baked potato	Food	18149.00	0.00	0.01	0.96	0.82	0.02	-	-
Fried potatoes and pommes frites	Food	18149.00	0.02	0.01	0.00	0.00	0.76	-	-
Mashed potato	Food	7782.00	0.01	0.01	0.05	0.00	0.83	-	-
Potato salad	Food	7782.00	0.01	0.01	0.27	0.84	0.01	-	-
Rice	Food	18149.00	-0.01	0.01	0.10	0.00	0.67	-	-
Pasta	Food	18149.00	0.00	0.01	0.85	0.00	0.48	-	-
Brown beans and pea soup	Food	18149.00	0.01	0.01	0.19	0.00	0.76	-	-
Margarine on bread	Food	18149.00	-0.01	0.01	0.15	0.00	0.79	-	-
Blota (broth + bread)	Food	7782.00	0.01	0.01	0.21	0.00	0.60	-	-
Pancake, waffle and Swedish dumpling	Food	18149.00	-0.01	0.01	0.01	0.00	0.78	-	-
Pizza	Food	18149.00	0.00	0.01	0.55	0.81	0.02	-	-
Minced meat dishes	Food	18149.00	0.01	0.01	0.14	0.88	0.00	-	-
Meat stew	Food	18149.00	0.00	0.01	0.46	0.68	0.08	-	-

Steak, chop, etc.	Food	18149.00	0.00	0.01	0.72	0.00	0.61	-	-
Bacon	Food	18149.00	0.01	0.00	0.00	0.05	0.30	-	-
Sausage as main dish	Food	18149.00	0.01	0.01	0.12	0.00	0.48	-	-
Hamburger	Food	18149.00	0.01	0.01	0.01	0.00	0.42	-	-
White meat (poultry)	Food	18149.00	0.00	0.01	0.64	0.00	0.51	-	-
Butter for cooking	Food	18149.00	0.01	0.01	0.07	0.25	0.25	-	-
Blood based food	Food	7782.00	-0.01	0.01	0.36	0.00	0.55	-	-
Liver and kidney	Food	7782.00	0.00	0.01	0.69	0.60	0.11	-	-
Lean fish (e.g. perch, bass, cod)	Food	18149.00	0.00	0.01	0.98	0.00	0.39	-	-
Fatty fish (e.g. herring, whitefish, salmon)	Food	18149.00	0.00	0.01	0.56	0.76	0.04	-	-
Shellfish (e.g. shrimps, scallops)	Food	7782.00	0.00	0.01	0.52	0.00	0.46	-	-
Salty fish	Food	18149.00	0.01	0.00	0.10	0.00	0.76	-	-
Smoked fish/meat	Food	18149.00	0.01	0.00	0.09	0.00	0.70	-	-
Ice cream	Food	18149.00	-0.02	0.01	0.00	0.00	0.74	-	-
Sweets (chocolate and candy)	Food	18149.00	0.00	0.01	0.93	0.00	0.82	-	-
Sugar, honey, marmelade and jam	Food	18149.00	0.00	0.00	0.96	0.00	0.51	-	-
Margarine for cooking	Food	18149.00	0.00	0.01	0.70	0.55	0.14	-	-
Cookies and pastry	Food	18149.00	-0.01	0.01	0.00	0.00	0.47	-	-
Chips, popcorn and salted nuts	Food	18149.00	0.02	0.01	0.00	0.00	0.32	-	-
Low fat milk (0.5%)	Beverage	18149.00	0.00	0.01	0.60	0.00	0.74	-	-
Milk, sour milk (1.5%)	Beverage	18149.00	0.01	0.01	0.02	0.00	0.36	-	-
Milk, sour milk (3%)	Beverage	18149.00	0.01	0.01	0.18	0.00	0.33	-	-
Sodas, soft drinks and juice	Beverage	18149.00	0.01	0.01	0.01	0.00	0.40	-	-
Brewed (filtered) coffee	Beverage	18149.00	0.00	0.00	0.65	0.00	1.00	-	-
Boiled coffee	Beverage	18149.00	0.01	0.01	0.22	0.00	0.52	-	-
Tea	Beverage	18149.00	-0.01	0.01	0.01	0.00	0.64	-	-
Light beer	Alcohol	18149.00	-0.01	0.01	0.01	0.82	0.02	-	-
Oil for cooking	Food	18149.00	-0.01	0.00	0.24	0.00	0.37	-	-
Medium beer	Alcohol	18149.00	0.01	0.01	0.24	0.00	0.82	-	-
Strong beer	Alcohol	18149.00	0.02	0.01	0.00	0.00	0.98	-	-
Wine	Alcohol	18149.00	0.01	0.01	0.04	0.00	0.75	-	-
Liquor and spirits	Alcohol	18149.00	0.02	0.01	0.00	0.21	0.26	-	-
Salad dressing with oil	Food	18149.00	-0.01	0.00	0.05	0.00	0.63	-	-
Cream, creme fraiche, sour cream	Food	18149.00	0.00	0.00	0.58	0.02	0.31	-	-
Average portion size of vegetables based on photographic illustration of four sizes (smallest to largest)	Food	18149.00	-0.02	0.01	0.00	0.00	0.83	-	-
Overall state of health during the last year	General health	19643.00	-0.03	0.00	0.00	0.00	0.80	0.259100667	6
Overall state of health compared to others your age	General health	19328.00	-0.01	0.00	0.24	0.00	0.71	-	-
Parents or siblings had a cerebral hemorrhage/thrombosis or cardiac infarction before the age of 60	General health	19466.00	0.04	0.01	0.00	0.00	0.66	-	-
Teetotaler	Alcohol	19625.00	-0.05	0.02	0.01	0.00	0.75	-	-
Feel the need to reduce alcohol consumption	Alcohol	17793.00	0.09	0.02	0.00	0.73	0.06	-	-
Feel uneasy or guilty because of your way of drinking	Alcohol	17787.00	0.06	0.02	0.00	0.85	0.01	-	-
Iron intake (mg/day)	Nutrients	18149.00	-0.01	0.01	0.34	0.32	0.23	-	-
Iodine intake (ug/day)	Nutrients	18149.00	0.01	0.01	0.06	0.00	0.66	-	-

Calcium intake (mg/day)	Nutrients	18149.00	-0.01	0.01	0.12	0.00	0.48	-	-
Potassium intake (mg/day)	Nutrients	18149.00	0.00	0.01	0.43	0.49	0.16	-	-
Beta-carotene intake (mg/day)	Nutrients	18149.00	-0.01	0.01	0.04	0.00	0.77	-	-
Cholesterol intake (g/day)	Nutrients	18149.00	0.00	0.01	0.46	0.00	0.98	-	-
Carbohydrates intake (g/day)	Nutrients	18149.00	-0.01	0.01	0.01	0.00	0.70	-	-
Average portion size of meat/fish based on photographic illustration of four sizes (smallest to largest)	Food	18149.00	0.00	0.01	0.89	0.73	0.06	-	-
Breakfast habits: Only coffee/tea for breakfast vs not breakfast at all	Food	1232.00	0.07	0.08	0.41	0.00	0.92	-	-
Breakfast habits: Coffee/tea and wheat buns or rusk for breakfast vs not breakfast at all	Food	1518.00	-0.06	0.05	0.29	0.00	0.55	-	-
Breakfast habits: Porridge w/o sandwich for breakfast vs not breakfast at all	Food	2604.00	-0.11	0.03	0.00	0.00	0.52	-	-
Breakfast habits: Gruel w/o sandwich for breakfast vs not breakfast at all	Food	1434.00	-0.16	0.06	0.01	0.00	0.52	-	-
Eat breakfast from 2000	Food	2021.00	-0.22	0.06	0.00	0.00	0.52	-	-
Enterodiol intake (ug/day)	Nutrients	18149.00	0.00	0.01	0.66	0.00	0.72	-	-
Enterolactone intake (ug/day)	Nutrients	18149.00	-0.01	0.01	0.05	0.00	0.49	-	-
Equol intake (ug/day)	Nutrients	18149.00	-0.01	0.01	0.11	0.00	0.62	-	-
Lariciresinol intake (ug/day)	Nutrients	18149.00	-0.01	0.01	0.01	0.00	0.62	-	-
Matairesinol intake (ug/day)	Nutrients	18149.00	-0.01	0.01	0.20	0.00	0.39	-	-
Medioresinol intake (ug/day)	Nutrients	18149.00	0.00	0.01	0.45	0.00	0.34	-	-
Pinoresinol intake (ug/day)	Nutrients	18149.00	-0.01	0.01	0.03	0.00	0.80	-	-
Secoisolariciresinol intake (ug/day)	Nutrients	18149.00	0.00	0.01	0.56	0.00	0.62	-	-
Sum of all ligns intake (ug/day)	Nutrients	18149.00	0.00	0.01	0.51	0.00	0.35	-	-
Syringaresinol intake (ug/day)	Nutrients	18149.00	0.00	0.01	0.89	0.26	0.25	-	-
Sum of Lariciresinol, Matairesinol, Pinoresinol, Secoisolariciresinol intake (ug/day)	Nutrients	18149.00	-0.01	0.01	0.02	0.00	0.66	-	-
Satisfaction with home and family situation	Psychosocial	11083.00	-0.02	0.01	0.02	0.00	0.98	-	-
Appetite status	Psychosocial	11074.00	-0.02	0.01	0.00	0.00	0.97	-	-
Mood status	Psychosocial	11069.00	-0.01	0.01	0.07	0.00	0.95	-	-
Energy status	Psychosocial	11067.00	-0.01	0.01	0.33	0.00	0.55	-	-
Patience status	Psychosocial	11078.00	-0.01	0.01	0.15	0.00	0.73	-	-
Confidence status	Psychosocial	11073.00	-0.01	0.01	0.42	0.00	0.58	-	-
Sleep status	Sleep	11094.00	-0.02	0.01	0.00	0.00	0.38	-	-
Do you feel important and appreciated outside your home?	Psychosocial	11091.00	0.00	0.01	0.97	0.00	0.50	-	-

Do you feel important and appreciated in your home?	Psychosocial	11005.00	0.00	0.01	0.46	0.00	0.43	-	-
Satisfaction with accomodation	Psychosocial	11090.00	-0.01	0.01	0.03	0.00	0.66	-	-
Satisfaction with work situation	Psychosocial	10969.00	-0.02	0.01	0.02	0.66	0.09	-	-
Satisfaction with economy	Psychosocial	11075.00	-0.02	0.01	0.02	0.00	0.80	-	-
Satisfaction with leisure time	Psychosocial	11069.00	-0.01	0.01	0.19	0.60	0.12	-	-
Hearing status	General health	11084.00	-0.01	0.01	0.05	0.00	0.47	-	-
Vision status	General health	11079.00	-0.01	0.01	0.36	0.81	0.02	-	-
Memory status	Psychosocial	11057.00	-0.01	0.01	0.03	0.45	0.18	-	-
Fitness status	Physical activity	11062.00	-0.01	0.01	0.46	0.00	0.46	-	-
Magnesium intake (mg/day)	Nutrients	18149.00	-0.01	0.01	0.09	0.00	0.67	-	-
Saturated fat intake (g/day)	Nutrients	18149.00	0.01	0.01	0.22	0.00	0.56	-	-
Monounsaturated fat intake (g/day)	Nutrients	18149.00	0.02	0.01	0.00	0.00	0.96	-	-
Monosaccharides intake (g/day)	Nutrients	18149.00	-0.01	0.01	0.05	0.00	0.95	-	-
Sodium intake (mg/day)	Nutrients	18149.00	0.01	0.01	0.01	0.00	0.79	-	-
Vitamin B3 intake (mg/day)	Nutrients	18149.00	0.01	0.01	0.14	0.09	0.29	-	-
Cambridge physical activity index	Physical activity	19287.00	0.00	0.00	0.97	0.00	0.37	-	-
Polyunsaturated fat intake (g/day)	Nutrients	18149.00	0.00	0.00	0.37	0.59	0.12	-	-
Average portion size of potatoes/rice/pasta based on photographic illustration of four sizes (smallest to largest)	Food	18149.00	-0.01	0.01	0.04	0.00	0.67	-	-
Total protein intake (g/day)	Nutrients	18149.00	0.00	0.01	0.92	0.00	0.90	-	-
Animal based protein intake (g/day)	Nutrients	18149.00	0.00	0.01	0.50	0.00	0.60	-	-
Plant based protein intake (g/day)	Nutrients	18149.00	-0.01	0.01	0.16	0.00	0.32	-	-
Vitamin A intake (mg/day)	Nutrients	18149.00	0.00	0.01	0.57	0.00	0.45	-	-
Sucrose intake (g/day)	Nutrients	18149.00	0.00	0.01	0.94	0.00	0.63	-	-
Cohabitation: Live alone vs Only one adult (spouse, partner)	Social	6018.00	-0.06	0.02	0.01	0.06	0.30	-	-
Cohabitation: Live alone vs Only children	Social	2612.00	0.02	0.03	0.56	0.00	0.41	-	-
Cohabitation: Live alone vs Adult and children	Social	13916.00	-0.05	0.02	0.01	0.00	0.40	-	-
Cohabitation: Live alone vs Other/others	Social	1951.00	-0.06	0.05	0.22	0.00	0.58	-	-
Selenium intake (ug/day)	Nutrients	18149.00	0.00	0.01	0.63	0.00	0.75	-	-
Long-term sickness	General health	18971.00	0.06	0.01	0.00	0.00	0.71	-	-
Work shifts/weekends	Psychosocial	19023.00	0.01	0.01	0.64	0.80	0.02	-	-
Number of cigarretes smoked per day (in groups)	Tobacco use	16449.00	0.04	0.01	0.00	0.18	0.27	0.262496847	5
Years smoking	Tobacco use	17393.00	0.04	0.01	0.00	0.64	0.10	0.252777936	10
Grams of tobacco smoked per week	Tobacco use	11923.00	0.05	0.01	0.00	0.00	0.55	0.254183276	9
Number of cigarretes smoked per day	Tobacco use	11923.00	0.05	0.01	0.00	0.00	0.55	0.254183276	7
Number of cigars smoked per day	Tobacco use	11923.00	0.05	0.01	0.00	0.00	0.55	0.254183276	8

Smoking status: Smokers vs non-smokers	Tobacco use	12934.00	0.12	0.01	0.00	0.00	0.35	0.265536473	2
Smoking status: Former smokers vs non-smokers	Tobacco use	13388.00	0.00	0.01	0.98	0.00	0.79	-	-
Smoking status: Former occasiol smokers vs non-smokers	Tobacco use	11109.00	0.01	0.02	0.56	0.47	0.17	-	-
Number of snuff boxes per week	Tobacco use	18573.00	0.03	0.01	0.00	0.47	0.17	0.264484961	3
Snuff status: Snuff users vs non-snuff users	Tobacco use	16600.00	0.07	0.01	0.00	0.13	0.28	0.262858733	4
Snuff status: Former snuff users vs non-snuff users	Tobacco use	15223.00	0.00	0.02	1.00	0.00	0.65	-	-
Years using snuff	Tobacco use	18051.00	0.02	0.01	0.00	0.00	0.52	-	-
Participation in associations or voluntary organisations	Social	19589.00	-0.04	0.01	0.00	0.19	0.27	-	-
Participation in sports or physical exercise associations	Social	1277.00	-0.01	0.05	0.83	0.35	0.21	-	-
Participation in study circles	Social	1277.00	0.00	0.07	0.96	0.00	0.57	-	-
Participation in other association	Social	1277.00	0.04	0.05	0.47	0.80	0.03	-	-
People to ask for help apart from the ones at home	Social	19583.00	0.01	0.02	0.39	0.00	0.62	-	-
Number of friends that can come to your home at any time and feel at home	Social	19577.00	0.00	0.00	0.40	0.00	0.82	-	-
Number of social contacts with the same interests as you	Social	19574.00	-0.01	0.00	0.13	0.00	0.96	-	-
Would you say that the number of people that you meet in your everyday life is enough or would you like to meet more or fewer people?	Social	19612.00	-0.01	0.00	0.03	0.00	0.96	-	-
Close relationship with anyone	Social	19629.00	-0.01	0.00	0.06	0.00	0.38	-	-
Frequency of engaging in clubs, associations or study circles	Social	13030.00	0.00	0.01	0.84	0.00	0.78	-	-
Number of social interactions during a normal week	Social	19624.00	-0.01	0.00	0.09	0.00	0.58	-	-
Support from others	Social	19620.00	-0.01	0.00	0.10	0.00	1.00	-	-
Number of people with whom you can speak openly	Social	19608.00	0.00	0.00	0.75	0.00	0.74	-	-
Receive hugs to comfort and support you	Social	19532.00	-0.02	0.01	0.17	0.00	0.72	-	-
Stigmasterol intake (mg/day)	Nutrients	18149.00	-0.01	0.01	0.05	0.00	0.33	-	-
Tiamin intake (mg/day)	Nutrients	18149.00	0.00	0.01	0.40	0.00	0.80	-	-
Vitamin E intake (mg/day)	Nutrients	18149.00	-0.01	0.01	0.04	0.00	0.60	-	-
Trans fat intake (g/day)	Nutrients	18149.00	0.00	0.01	0.91	0.00	0.91	-	-
Sum of phytosterols intake (mg/day)	Nutrients	18149.00	-0.01	0.01	0.02	0.00	0.44	-	-
Educatiol level	Psychosocial	19644.00	-0.01	0.01	0.06	0.00	0.88	-	-

Zinc intake (mg/day)	Nutrients	18149.00	-0.01	0.01	0.05	0.12	0.29	-	-

Supplementary Table 17. Longitudinal association results for HDL cholesterol

Description	Group	N	Effect estimate	S.E.	p-value	I^2	Q p-value	adjusted R2	R2 rank
Alcohol intake (g/day)	Alcohol	3473.00	0.02	0.01	0.00	0.04	0.31	-	-
Permanent employment	Psychosocial	3710.00	0.00	0.01	0.77	0.54	0.14	-	-
Self-employed	Psychosocial	3710.00	0.04	0.02	0.04	0.84	0.01	-	-
Distance to work in kilometers (one way)	Physical activity	3201.00	0.00	0.01	0.76	0.00	0.33	-	-
Last time a colleague visited you at home	Psychosocial	3666.00	0.01	0.01	0.04	0.73	0.06	-	-
Job demands to work very fast	Psychosocial	3705.00	0.01	0.01	0.16	0.51	0.15	-	-
Frequency of social contacts with colleagues during leisure time	Psychosocial	3605.00	0.00	0.01	0.53	0.00	0.69	-	-
High physical demand from job	Physical activity	3720.00	0.01	0.01	0.18	0.33	0.22	-	-
Enough time for job assignments	Psychosocial	3691.00	0.00	0.01	0.49	0.00	0.82	-	-
Control over planning and execution of the workday	Psychosocial	3714.00	0.01	0.01	0.29	0.00	0.45	-	-
Ingenuity or creativity demand from job	Psychosocial	3705.00	0.01	0.01	0.35	0.39	0.20	-	-
Frequent social contacts with colleagues during work	Psychosocial	3684.00	0.01	0.01	0.04	0.48	0.17	-	-
Contradictory demands in job	Psychosocial	3680.00	0.00	0.01	0.43	0.41	0.19	-	-
Possibility to leave your work for a while to speak with a colleague	Psychosocial	3679.00	0.00	0.01	0.66	0.76	0.04	-	-
Learn new things at job	Psychosocial	3701.00	0.01	0.01	0.07	0.00	0.47	-	-
High mental demand from job	Psychosocial	3674.00	0.00	0.01	0.76	0.00	0.48	-	-
Repetitive job	Psychosocial	3711.00	-0.01	0.01	0.06	0.00	0.35	-	-
Skill demand from job	Psychosocial	3700.00	0.01	0.01	0.32	0.00	0.88	-	-
Possibility to speak with colleagues during breaks	Psychosocial	3700.00	0.00	0.01	0.85	0.00	0.90	-	-
Control over own work assignment	Psychosocial	3705.00	0.00	0.01	0.85	0.00	0.32	-	-
Vitamin C intake (mg/day)	Nutrients	3473.00	-0.01	0.01	0.19	0.72	0.06	-	-
Vitamin B12 intake (ug/day)	Nutrients	3473.00	0.00	0.01	0.76	0.00	0.51	-	-
Vitamin B2 intake (ug/day)	Nutrients	3473.00	-0.01	0.01	0.34	0.00	0.39	-	-
Vitamin B6 intake (mg/day)	Nutrients	3473.00	0.01	0.01	0.12	0.26	0.25	-	-
Informed of having high blood pressure	General health	3769.00	-0.04	0.02	0.03	0.00	0.74	-	-
Beta-sitostanol intake (mg/day)	Nutrients	3473.00	0.01	0.01	0.16	0.00	0.48	-	-
Beta-sitosterol intake (mg/day)	Nutrients	3473.00	0.00	0.01	0.83	0.45	0.18	-	-
Marital status: Single vs Married/partner	Social	3503.00	0.02	0.02	0.26	0.00	1.00	-	-
Marital status: Single vs Divorced/separated	Social	651.00	-0.03	0.03	0.27	0.00	0.75	-	-
Marital status: Single vs Widow/widower	Social	451.00	-0.02	0.07	0.78	0.72	0.06	-	-
Campestanol intake (mg/day)	Nutrients	3473.00	0.01	0.01	0.24	0.00	0.41	-	-
Campesterol intake (mg/day)	Nutrients	3473.00	0.00	0.01	0.79	0.00	0.76	-	-
Parents or siblings have diabetes	General health	3742.00	-0.01	0.02	0.49	0.60	0.11	-	-
Disaccharides intake (g/day)	Nutrients	3473.00	-0.02	0.01	0.00	0.00	0.32	-	-
Vitamin D intake (ug/day)	Nutrients	3473.00	0.00	0.01	0.95	0.00	0.44	-	-
Total energy intake (kcal/day)	Nutrients	3473.00	0.00	0.01	0.87	0.42	0.19	-	-

Formic acid intaje (g/day)	Nutrients	3473.00	-0.01	0.01	0.22	0.00	0.43	-	-
Pentadecanoic acid intake (g/day)	Nutrients	3473.00	-0.01	0.01	0.19	0.00	0.32	-	-
Palmitic acid intake (g/day)	Nutrients	3473.00	0.00	0.01	0.46	0.00	0.95	-	-
Heptadecanoic acid intake (g/day)	Nutrients	3473.00	-0.01	0.01	0.19	0.00	0.32	-	-
Linoleic acid intake (g/day)	Nutrients	3473.00	0.00	0.01	0.52	0.00	0.39	-	-
Linolenic acid intake (g/day)	Nutrients	3473.00	0.00	0.01	0.62	0.00	0.58	-	-
Arachidonic acid (ARA) intake (g/day)	Nutrients	3473.00	0.00	0.01	0.43	0.74	0.05	-	-
Eicosapentaenoic acid (EPA) intake (g/day)	Nutrients	3473.00	0.01	0.01	0.05	0.00	0.34	-	-
Docosahexaenoic acid (DHA) intake (g/day)	Nutrients	3473.00	0.01	0.01	0.06	0.14	0.28	-	-
Fat intake (g/day)	Nutrients	3473.00	0.00	0.01	0.75	0.00	0.70	-	-
Fibre intake (g/day)	Nutrients	3473.00	0.01	0.01	0.30	0.37	0.21	-	-
Folic acid intake (ug/day)	Nutrients	3473.00	0.01	0.01	0.39	0.81	0.02	-	-
Phosphate intake (mg/day)	Nutrients	3473.00	0.00	0.01	0.52	0.00	0.38	-	-
Whole grain intake (g/day)	Food	3473.00	0.01	0.01	0.26	0.00	0.65	-	-
Travel to work: Walk to work vs passive travel to work	Physical activity	2513.00	0.01	0.02	0.69	0.79	0.03	-	-
Travel to work: Cycle to work vs passive travel to work	Physical activity	2771.00	0.01	0.02	0.66	0.00	0.68	-	-
Travel to work: Irregular travel mode to work vs passive travel to work	Physical activity	2516.00	-0.01	0.02	0.81	0.00	0.89	-	-
Sedentary or standing work	Physical activity	3696.00	-0.02	0.01	0.24	0.00	0.64	-	-
Light but partly physically active work	Physical activity	3696.00	-0.03	0.02	0.13	0.00	0.41	-	-
Light and physically active work	Physical activity	3696.00	0.03	0.01	0.03	0.00	0.71	-	-
Sometimes physically straining work	Physical activity	3696.00	0.02	0.01	0.21	0.00	0.50	-	-
Frequency of walking during leisure time	Physical activity	3632.00	0.01	0.01	0.33	0.00	0.96	-	-
Frequency of cycling during leisure time	Physical activity	3047.00	0.01	0.01	0.07	0.00	0.42	-	-
Frequency of dancing during leisure time	Physical activity	3206.00	0.00	0.01	0.79	0.00	0.80	-	-
Frequency of shoveling snow during leisure time	Physical activity	3565.00	0.00	0.01	0.58	0.00	0.97	-	-
Frequency of gardening during leisure time	Physical activity	3499.00	0.00	0.01	0.95	0.00	0.92	-	-
Frequency of hunting or fishing during leisure time	Physical activity	3461.00	0.00	0.01	0.90	0.00	0.76	-	-
Frequency of picking berries or mushrooms during leisure time	Physical activity	3509.00	-0.01	0.01	0.20	0.00	0.70	-	-
Changed everyday exercise during the last year	Physical activity	3768.00	0.00	0.01	0.68	0.00	0.45	-	-
Everyday exercise satisfaction	Physical activity	3761.00	0.01	0.01	0.13	0.67	0.08	-	-
Exercise during the last three months	Physical activity	3766.00	0.01	0.01	0.04	0.53	0.15	-	-
If you exercise, change in exercise habits during the last year	Physical activity	3314.00	0.01	0.01	0.25	0.00	0.47	-	-
Bregott on bread	Food	3473.00	-0.01	0.01	0.24	0.00	0.40	-	-
Whole grain crisp bread	Food	3473.00	0.01	0.01	0.24	0.00	0.96	-	-
Whole grain soft bread	Food	3473.00	0.01	0.01	0.44	0.00	0.45	-	-
White (soft) bread, thin crisp bread	Food	3473.00	0.00	0.01	0.55	0.00	0.77	-	-

Coffee rolls/buns, rusk	Food	3473.00	-0.01	0.01	0.13	0.00	0.94	-	-
Cheese 28%	Food	3473.00	0.00	0.01	0.74	0.00	0.75	-	-
Cheese 10-17%	Food	3473.00	0.00	0.01	0.59	0.10	0.29	-	-
Soft cheese	Food	1231.00	0.01	0.01	0.30	0.00	0.38	-	-
Soft whey cheese	Food	1231.00	-0.01	0.01	0.60	0.77	0.04	-	-
Sausage, liver pate on bread	Food	3473.00	0.00	0.01	0.91	0.00	0.48	-	-
Meat on bread	Food	3473.00	-0.01	0.01	0.19	0.72	0.06	-	-
Butter on bread	Food	3473.00	0.00	0.01	0.52	0.00	0.34	-	-
Oatflake, whole wheat, rye or barley porridge	Food	3473.00	0.00	0.01	0.56	0.00	0.55	-	-
Rosehip, sweet syrup soup	Food	3473.00	-0.01	0.01	0.10	0.00	0.76	-	-
Sour milk, yoghurt (3% fat)	Food	3473.00	0.00	0.01	0.50	0.40	0.20	-	-
Sour milk, yoghurt (low fat)	Food	3473.00	0.02	0.01	0.01	0.00	0.91	-	-
Fiber cereals	Food	3473.00	0.00	0.01	0.56	0.00	0.38	-	-
Corn flakes	Food	3473.00	0.01	0.01	0.41	0.00	0.99	-	-
Berries (fresh or frozen)	Food	3473.00	0.00	0.01	0.87	0.00	0.56	-	-
Apple, pear, peach, orange, mandarin and grapefruit	Food	3473.00	0.00	0.01	0.66	0.00	0.61	-	-
Ba	Food	3473.00	0.00	0.01	0.54	0.00	0.57	-	-
Root vegetables and carrot	Food	3473.00	0.00	0.01	0.63	0.70	0.07	-	-
Low fat margarine on bread	Food	3473.00	0.01	0.01	0.38	0.18	0.27	-	-
Tomato and cucumber	Food	3473.00	0.00	0.01	0.65	0.00	0.33	-	-
White cabbage, lettuce, lettuce cabbage, spich, borecole and broccoli	Food	3473.00	0.00	0.01	0.60	0.85	0.01	-	-
Mixed frozen vegetables	Food	1231.00	-0.01	0.01	0.56	0.71	0.06	-	-
Boiled or baked potato	Food	3473.00	0.01	0.01	0.24	0.60	0.11	-	-
Fried potatoes and pommes frites	Food	3473.00	0.01	0.01	0.21	0.64	0.09	-	-
Mashed potato	Food	1231.00	-0.01	0.01	0.23	0.00	0.99	-	-
Potato salad	Food	1231.00	-0.01	0.01	0.37	0.00	0.96	-	-
Rice	Food	3473.00	0.00	0.01	0.83	0.00	0.53	-	-
Pasta	Food	3473.00	0.00	0.01	0.46	0.00	0.94	-	-
Brown beans and pea soup	Food	3473.00	0.00	0.01	0.66	0.00	0.52	-	-
Margarine on bread	Food	3473.00	0.00	0.01	0.76	0.00	0.54	-	-
Blota (broth + bread)	Food	1231.00	-0.01	0.01	0.48	0.00	0.42	-	-
Pancake, waffle and Swedish dumpling	Food	3473.00	0.00	0.01	0.82	0.00	0.92	-	-
Pizza	Food	3473.00	-0.01	0.01	0.21	0.00	0.62	-	-
Minced meat dishes	Food	3473.00	0.00	0.01	0.76	0.00	0.63	-	-
Meat stew	Food	3473.00	0.01	0.01	0.15	0.00	0.67	-	-
Steak, chop, etc.	Food	3473.00	0.01	0.01	0.09	0.58	0.12	-	-
Bacon	Food	3473.00	0.01	0.01	0.03	0.45	0.18	-	-
Sausage as main dish	Food	3473.00	0.00	0.01	0.44	0.11	0.29	-	-
Hamburger	Food	3473.00	-0.01	0.01	0.19	0.30	0.23	-	-
White meat (poultry)	Food	3473.00	0.01	0.01	0.15	0.00	0.99	-	-
Butter for cooking	Food	3473.00	0.00	0.01	0.74	0.42	0.19	-	-
Blood based food	Food	1231.00	0.01	0.01	0.43	0.00	0.34	-	-
Liver and kidney	Food	1231.00	0.01	0.01	0.54	0.00	0.45	-	-
Lean fish (e.g. perch, bass, cod)	Food	3473.00	0.00	0.01	0.69	0.74	0.05	-	-
Fatty fish (e.g. herring, whitefish, salmon)	Food	3473.00	0.01	0.01	0.07	0.00	0.87	-	-
Shellfish (e.g. shrimps, scallops)	Food	1231.00	0.01	0.01	0.33	0.00	0.92	-	-
Salty fish	Food	3473.00	0.01	0.01	0.17	0.62	0.11	-	-
Smoked fish/meat	Food	3473.00	-0.01	0.01	0.24	0.00	0.48	-	-
Ice cream	Food	3473.00	0.00	0.01	0.87	0.00	0.78	-	-
Sweets (chocolate and candy)	Food	3473.00	-0.01	0.01	0.31	0.54	0.14	-	-
Sugar, honey, marmelade and jam	Food	3473.00	0.00	0.01	0.49	0.00	0.95	-	-
Margarine for cooking	Food	3473.00	0.01	0.01	0.40	0.52	0.15	-	-
Cookies and pastry	Food	3473.00	-0.01	0.01	0.16	0.00	0.34	-	-

Chips, popcorn and salted nuts	Food	3473.00	0.00	0.01	0.58	0.00	0.89	-	-
Low fat milk (0.5%)	Beverage	3473.00	0.01	0.01	0.28	0.00	0.32	-	-
Milk, sour milk (1.5%)	Beverage	3473.00	-0.02	0.01	0.00	0.00	0.65	-	-
Milk, sour milk (3%)	Beverage	3473.00	0.00	0.01	0.52	0.00	0.84	-	-
Sodas, soft drinks and juice	Beverage	3473.00	-0.01	0.01	0.25	0.00	0.34	-	-
Brewed (filtered) coffee	Beverage	3473.00	0.01	0.01	0.41	0.00	0.47	-	-
Boiled coffee	Beverage	3473.00	0.01	0.01	0.12	0.00	0.55	-	-
Tea	Beverage	3473.00	-0.02	0.01	0.02	0.00	0.71	-	-
Light beer	Alcohol	3473.00	0.01	0.01	0.33	0.28	0.24	-	-
Oil for cooking	Food	3473.00	-0.01	0.01	0.29	0.30	0.23	-	-
Medium beer	Alcohol	3473.00	0.02	0.01	0.00	0.00	0.44	-	-
Strong beer	Alcohol	3473.00	0.01	0.01	0.03	0.37	0.21	-	-
Wine	Alcohol	3473.00	0.02	0.01	0.00	0.38	0.20	-	-
Liquor and spirits	Alcohol	3473.00	0.00	0.01	0.49	0.36	0.21	-	-
Salad dressing with oil	Food	3473.00	0.01	0.01	0.37	0.15	0.28	-	-
Cream, creme fraiche, sour cream	Food	3473.00	0.00	0.01	0.85	0.00	0.73	-	-
Average portion size of vegetables based on photographic illustration of four sizes (smallest to largest)	Food	3473.00	0.01	0.01	0.22	0.39	0.20	-	-
Overall state of health during the last year	General health	3764.00	0.00	0.01	0.69	0.00	0.82	-	-
Overall state of health compared to others your age	General health	3698.00	0.01	0.01	0.25	0.50	0.16	-	-
Parents or siblings had a cerebral hemorrhage/thrombosis or cardiac infarction before the age of 60	General health	3730.00	-0.02	0.02	0.17	0.00	0.42	-	-
Teetotaler	Alcohol	3759.00	-0.03	0.02	0.18	0.46	0.17	-	-
Feel the need to reduce alcohol consumption	Alcohol	3368.00	0.02	0.02	0.28	0.63	0.10	-	-
Feel uneasy or guilty because of your way of drinking	Alcohol	3367.00	0.02	0.02	0.46	0.00	0.62	-	-
Iron intake (mg/day)	Nutrients	3473.00	0.01	0.01	0.17	0.00	0.84	-	-
Iodine intake (ug/day)	Nutrients	3473.00	-0.01	0.01	0.23	0.00	0.56	-	-
Calcium intake (mg/day)	Nutrients	3473.00	-0.01	0.01	0.23	0.54	0.14	-	-
Potassium intake (mg/day)	Nutrients	3473.00	0.01	0.01	0.36	0.00	1.00	-	-
Beta-carotene intake (mg/day)	Nutrients	3473.00	0.00	0.01	0.65	0.73	0.05	-	-
Cholesterol intake (g/day)	Nutrients	3473.00	0.00	0.01	0.79	0.00	0.55	-	-
Carbohydrates intake (g/day)	Nutrients	3473.00	0.00	0.01	0.47	0.00	0.72	-	-
Average portion size of meat/fish based on photographic illustration of four sizes (smallest to largest)	Food	3473.00	0.01	0.01	0.13	0.78	0.03	-	-
Breakfast habits: Only coffee/tea for breakfast vs not breakfast at all	Food	277.00	0.08	0.07	0.29	0.00	0.97	-	-
Breakfast habits: Coffee/tea and wheat buns or rusk for breakfast vs not breakfast at all	Food	355.00	0.02	0.04	0.67	0.00	0.68	-	-
Breakfast habits: Porridge w/o sandwich for breakfast vs not breakfast at all	Food	533.00	0.02	0.03	0.51	0.69	0.07	-	-
Breakfast habits: Gruel w/o sandwich for breakfast vs not breakfast at all	Food	310.00	0.04	0.05	0.49	0.00	0.33	-	-

Enterodiol intake (ug/day)	Nutrients	3473.00	0.01	0.01	0.20	0.00	0.39	-	-
Enterolactone intake (ug/day)	Nutrients	3473.00	0.01	0.01	0.43	0.56	0.13	-	-
Equol intake (ug/day)	Nutrients	3473.00	0.02	0.01	0.02	0.00	0.39	-	-
Lariciresinol intake (ug/day)	Nutrients	3473.00	0.01	0.01	0.25	0.00	0.36	-	-
Matairesinol intake (ug/day)	Nutrients	3473.00	0.01	0.01	0.06	0.00	0.37	-	-
Medioresinol intake (ug/day)	Nutrients	3473.00	0.01	0.01	0.16	0.00	0.59	-	-
Pinoresinol intake (ug/day)	Nutrients	3473.00	0.01	0.01	0.39	0.40	0.20	-	-
Secoisolariciresinol intake (ug/day)	Nutrients	3473.00	0.02	0.01	0.01	0.66	0.08	-	-
Sum of all ligns intake (ug/day)	Nutrients	3473.00	0.01	0.01	0.15	0.00	0.39	-	-
Syringaresinol intake (ug/day)	Nutrients	3473.00	0.01	0.01	0.16	0.00	0.50	-	-
Sum of Lariciresinol, Matairesinol, Pinoresinol, Secoisolariciresinol intake (ug/day)	Nutrients	3473.00	0.01	0.01	0.20	0.32	0.22	-	-
Satisfaction with home and family situation	Psychosocial	2421.00	0.01	0.01	0.17	0.00	0.92	-	-
Appetite status	Psychosocial	2421.00	0.01	0.01	0.15	0.00	0.72	-	-
Mood status	Psychosocial	2415.00	0.01	0.01	0.43	0.16	0.28	-	-
Energy status	Psychosocial	2420.00	0.01	0.01	0.14	0.00	0.60	-	-
Patience status	Psychosocial	2420.00	0.00	0.01	0.95	0.00	0.71	-	-
Confidence status	Psychosocial	2417.00	0.00	0.01	0.88	0.00	0.71	-	-
Sleep status	Sleep	2424.00	0.01	0.01	0.28	0.00	0.67	-	-
Do you feel important and appreciated outside your home?	Psychosocial	2422.00	0.01	0.01	0.32	0.00	0.90	-	-
Do you feel important and appreciated in your home?	Psychosocial	2405.00	0.01	0.01	0.44	0.00	0.75	-	-
Satisfaction with accomodation	Psychosocial	2421.00	0.00	0.01	0.95	0.00	0.85	-	-
Satisfaction with work situation	Psychosocial	2385.00	0.00	0.01	0.86	0.00	0.63	-	-
Satisfaction with economy	Psychosocial	2414.00	0.00	0.01	0.70	0.00	0.39	-	-
Satisfaction with leisure time	Psychosocial	2416.00	0.00	0.01	0.63	0.38	0.20	-	-
Hearing status	General health	2426.00	0.02	0.01	0.01	0.00	0.95	-	-
Vision status	General health	2427.00	0.01	0.01	0.37	0.20	0.26	-	-
Memory status	Psychosocial	2414.00	0.00	0.01	0.74	0.00	0.85	-	-
Fitness status	Physical activity	2417.00	0.03	0.01	0.00	0.00	0.98	-	-
Magnesium intake (mg/day)	Nutrients	3473.00	0.01	0.01	0.06	0.36	0.21	-	-
Saturated fat intake (g/day)	Nutrients	3473.00	-0.01	0.01	0.35	0.00	0.88	-	-
Monounsaturated fat intake (g/day)	Nutrients	3473.00	0.00	0.01	0.80	0.13	0.28	-	-
Monosaccharides intake (g/day)	Nutrients	3473.00	-0.01	0.01	0.42	0.55	0.14	-	-
Sodium intake (mg/day)	Nutrients	3473.00	0.01	0.01	0.07	0.44	0.18	-	-
Vitamin B3 intake (mg/day)	Nutrients	3473.00	0.02	0.01	0.00	0.56	0.13	-	-
Cambridge physical activity index	Physical activity	3688.00	0.01	0.01	0.07	0.63	0.10	-	-
Polyunsaturated fat intake (g/day)	Nutrients	3473.00	0.00	0.01	0.75	0.00	0.82	-	-
Average portion size of potatoes/rice/pasta based on photographic illustration of four sizes (smallest to largest)	Food	3473.00	0.01	0.01	0.35	0.00	0.48	-	-
Total protein intake (g/day)	Nutrients	3473.00	0.01	0.01	0.37	0.00	0.68	-	-

Animal based protein intake (g/day)	Nutrients	3473.00	0.00	0.01	0.72	0.00	0.52	-	-
Plant based protein intake (g/day)	Nutrients	3473.00	0.01	0.01	0.26	0.00	0.51	-	-
Vitamin A intake (mg/day)	Nutrients	3473.00	0.00	0.01	0.95	0.00	0.42	-	-
Sucrose intake (g/day)	Nutrients	3473.00	-0.02	0.01	0.00	0.00	0.43	-	-
Cohabitation: Live alone vs Only one adult (spouse, partner)	Social	1224.00	0.00	0.02	0.85	0.00	0.36	-	-
Cohabitation: Live alone vs Only children	Social	513.00	-0.09	0.03	0.01	0.00	0.38	-	-
Cohabitation: Live alone vs Adult and children	Social	2636.00	0.03	0.02	0.28	0.23	0.26	-	-
Cohabitation: Live alone vs Other/others	Social	433.00	0.07	0.04	0.08	0.00	0.59	-	-
Selenium intake (ug/day)	Nutrients	3473.00	0.00	0.01	0.54	0.00	0.47	-	-
Long-term sickness	General health	3654.00	-0.02	0.02	0.24	0.00	0.84	-	-
Work shifts/weekends	Psychosocial	3641.00	0.01	0.01	0.32	0.04	0.31	-	-
Number of cigarretes smoked per day (in groups)	Tobacco use	3106.00	-0.02	0.01	0.02	0.00	0.79	-	-
Years smoking	Tobacco use	3326.00	-0.02	0.01	0.01	0.00	0.62	-	-
Grams of tobacco smoked per week	Tobacco use	2226.00	0.02	0.01	0.14	0.00	0.34	-	-
Number of cigarretes smoked per day	Tobacco use	2226.00	0.02	0.01	0.14	0.00	0.34	-	-
Number of cigars smoked per day	Tobacco use	2226.00	0.02	0.01	0.14	0.00	0.34	-	-
Smoking status: Smokers vs non-smokers	Tobacco use	2490.00	-0.04	0.02	0.02	0.00	0.41	-	-
Smoking status: Former smokers vs non-smokers	Tobacco use	2614.00	0.00	0.02	0.78	0.00	0.40	-	-
Smoking status: Former occasiol smokers vs non-smokers	Tobacco use	2113.00	0.00	0.02	0.83	0.00	0.50	-	-
Number of snuff boxes per week	Tobacco use	3584.00	0.01	0.01	0.34	0.28	0.24	-	-
Snuff status: Snuff users vs non-snuff users	Tobacco use	3153.00	0.02	0.02	0.21	0.00	0.66	-	-
Snuff status: Former snuff users vs non-snuff users	Tobacco use	2794.00	0.01	0.02	0.49	0.89	0.00	-	-
Years using snuff	Tobacco use	3444.00	0.01	0.01	0.06	0.00	0.90	-	-
Participation in associations or voluntary organisations	Social	3747.00	-0.01	0.01	0.43	0.00	0.76	-	-
People to ask for help apart from the ones at home	Social	3755.00	-0.03	0.02	0.18	0.00	0.83	-	-
Number of friends that can come to your home at any time and feel at home	Social	3751.00	0.00	0.01	0.48	0.00	0.87	-	-
Number of social contacts with the same interests as you	Social	3757.00	0.01	0.01	0.07	0.00	0.84	-	-
Would you say that the number of people that you meet in your everyday life is enough or would you like to meet more or fewer people?	Social	3758.00	0.01	0.01	0.05	0.00	0.74	-	-
Close relationship with anyone	Social	3763.00	0.01	0.01	0.07	0.73	0.06	-	-
Frequency of engaging in clubs, associations or study circles	Social	2512.00	-0.01	0.01	0.34	0.29	0.23	-	-
Number of social interactions during a normal week	Social	3757.00	-0.01	0.01	0.26	0.00	0.97	-	-

Support from others	Social	3759.00	0.01	0.01	0.22	0.85	0.01	-	-
Number of people with whom you can speak openly	Social	3760.00	0.00	0.01	0.98	0.00	1.00	-	-
Receive hugs to comfort and support you	Social	3733.00	0.01	0.02	0.64	0.00	0.95	-	-
Stigmasterol intake (mg/day)	Nutrients	3473.00	0.00	0.01	0.85	0.13	0.28	-	-
Tiamin intake (mg/day)	Nutrients	3473.00	0.01	0.01	0.12	0.00	0.82	-	-
Vitamin E intake (mg/day)	Nutrients	3473.00	0.00	0.01	0.45	0.00	0.42	-	-
Trans fat intake (g/day)	Nutrients	3473.00	-0.01	0.01	0.37	0.14	0.28	-	-
Sum of phytosterols intake (mg/day)	Nutrients	3473.00	0.00	0.01	0.93	0.17	0.27	-	-
Educatiol level	Psychosocial	3766.00	0.01	0.01	0.14	0.14	0.28	-	-
Zinc intake (mg/day)	Nutrients	3473.00	0.01	0.01	0.34	0.00	0.64	-	-

Supplementary Table 18. Longitudinal association results for LDL cholesterol

Description	Group	N	Effect estimate	S.E.	p-value	I^2	Q p-value	adjusted R2	R2 rank
Alcohol intake (g/day)	Alcohol	3405.00	0.01	0.02	0.64	0.00	0.33	-	-
Permanent employment	Psychosocial	3637.00	0.01	0.03	0.73	0.49	0.16	-	-
Self-employed	Psychosocial	3637.00	-0.04	0.05	0.37	0.00	0.80	-	-
Distance to work in kilometers (one way)	Physical activity	3135.00	0.00	0.01	0.77	0.00	0.40	-	-
Last time a colleague visited you at home	Psychosocial	3596.00	-0.02	0.01	0.08	0.00	0.91	-	-
Job demands to work very fast	Psychosocial	3635.00	-0.03	0.01	0.06	0.00	0.84	-	-
Frequency of social contacts with colleagues during leisure time	Psychosocial	3535.00	-0.04	0.01	0.00	0.00	0.68	-	-
High physical demand from job	Physical activity	3650.00	0.01	0.01	0.73	0.00	0.56	-	-
Enough time for job assignments	Psychosocial	3620.00	0.00	0.01	0.77	0.14	0.28	-	-
Control over planning and execution of the workday	Psychosocial	3643.00	-0.03	0.01	0.08	0.00	0.47	-	-
Ingenuity or creativity demand from job	Psychosocial	3634.00	-0.03	0.01	0.03	0.00	0.71	-	-
Frequent social contacts with colleagues during work	Psychosocial	3614.00	0.00	0.01	0.74	0.72	0.06	-	-
Contradictory demands in job	Psychosocial	3610.00	-0.01	0.01	0.52	0.00	0.95	-	-
Possibility to leave your work for a while to speak with a colleague	Psychosocial	3608.00	-0.03	0.01	0.04	0.00	0.37	-	-
Learn new things at job	Psychosocial	3630.00	-0.03	0.01	0.02	0.73	0.05	-	-
High mental demand from job	Psychosocial	3603.00	0.00	0.01	0.87	0.00	0.82	-	-
Repetitive job	Psychosocial	3640.00	0.00	0.01	0.76	0.00	0.65	-	-
Skill demand from job	Psychosocial	3630.00	-0.01	0.01	0.41	0.00	0.57	-	-
Possibility to speak with colleagues during breaks	Psychosocial	3630.00	0.01	0.01	0.44	0.67	0.08	-	-
Control over own work assignment	Psychosocial	3634.00	-0.01	0.01	0.30	0.74	0.05	-	-
Vitamin C intake (mg/day)	Nutrients	3405.00	-0.04	0.02	0.01	0.00	0.75	-	-
Vitamin B12 intake (ug/day)	Nutrients	3405.00	0.01	0.01	0.67	0.00	0.99	-	-
Vitamin B2 intake (ug/day)	Nutrients	3405.00	0.03	0.01	0.08	0.00	0.68	-	-
Vitamin B6 intake (mg/day)	Nutrients	3405.00	-0.01	0.02	0.62	0.00	0.59	-	-
Informed of having high blood pressure	General health	3695.00	-0.06	0.04	0.13	0.00	0.74	-	-
Beta-sitostanol intake (mg/day)	Nutrients	3405.00	-0.01	0.01	0.39	0.00	0.45	-	-
Beta-sitosterol intake (mg/day)	Nutrients	3405.00	-0.01	0.01	0.63	0.00	0.38	-	-
Marital status: Single vs Married/partner	Social	3436.00	-0.02	0.05	0.58	0.57	0.13	-	-
Marital status: Single vs Divorced/separated	Social	633.00	0.08	0.07	0.27	0.00	0.63	-	-
Marital status: Single vs Widow/widower	Social	441.00	-0.24	0.19	0.20	0.37	0.21	-	-
Campestanol intake (mg/day)	Nutrients	3405.00	-0.01	0.01	0.45	0.22	0.26	-	-
Campesterol intake (mg/day)	Nutrients	3405.00	0.00	0.01	0.77	0.47	0.17	-	-

Parents or siblings have diabetes	General health	3667.00	-0.02	0.04	0.67	0.85	0.01	-	-
Disaccharides intake (g/day)	Nutrients	3405.00	0.02	0.02	0.12	0.60	0.12	-	-
Vitamin D intake (ug/day)	Nutrients	3405.00	0.01	0.01	0.58	0.69	0.07	-	-
Total energy intake (kcal/day)	Nutrients	3405.00	0.00	0.02	0.85	0.45	0.18	-	-
Formic acid intaje (g/day)	Nutrients	3405.00	0.01	0.01	0.56	0.00	0.93	-	-
Pentadecanoic acid intake (g/day)	Nutrients	3405.00	0.01	0.01	0.53	0.00	0.97	-	-
Palmitic acid intake (g/day)	Nutrients	3405.00	0.01	0.01	0.44	0.00	0.79	-	-
Heptadecanoic acid intake (g/day)	Nutrients	3405.00	0.01	0.01	0.53	0.00	0.97	-	-
Linoleic acid intake (g/day)	Nutrients	3405.00	0.01	0.01	0.39	0.37	0.21	-	-
Linolenic acid intake (g/day)	Nutrients	3405.00	0.01	0.01	0.67	0.63	0.10	-	-
Arachidonic acid (ARA) intake (g/day)	Nutrients	3405.00	0.01	0.01	0.46	0.00	0.95	-	-
Eicosapentaenoic acid (EPA) intake (g/day)	Nutrients	3405.00	0.00	0.02	0.84	0.00	0.87	-	-
Docosahexaenoic acid (DHA) intake (g/day)	Nutrients	3405.00	0.00	0.02	0.80	0.00	0.87	-	-
Fat intake (g/day)	Nutrients	3405.00	0.00	0.01	0.83	0.00	0.44	-	-
Fibre intake (g/day)	Nutrients	3405.00	-0.02	0.02	0.18	0.00	0.69	-	-
Folic acid intake (ug/day)	Nutrients	3405.00	0.01	0.02	0.70	0.00	0.37	-	-
Phosphate intake (mg/day)	Nutrients	3405.00	0.02	0.02	0.18	0.00	0.93	-	-
Whole grain intake (g/day)	Food	3405.00	0.02	0.01	0.26	0.00	0.66	-	-
Travel to work: Walk to work vs passive travel to work	Physical activity	2468.00	-0.04	0.05	0.46	0.00	0.68	-	-
Travel to work: Cycle to work vs passive travel to work	Physical activity	2717.00	-0.03	0.04	0.40	0.00	0.39	-	-
Travel to work: Irregular travel mode to work vs passive travel to work	Physical activity	2470.00	-0.10	0.05	0.04	0.00	0.63	-	-
Sedentary or standing work	Physical activity	3626.00	0.01	0.03	0.67	0.00	0.99	-	-
Light but partly physically active work	Physical activity	3626.00	-0.03	0.04	0.47	0.85	0.01	-	-
Light and physically active work	Physical activity	3626.00	0.05	0.03	0.17	0.00	0.45	-	-
Sometimes physically straining work	Physical activity	3626.00	-0.05	0.03	0.11	0.06	0.30	-	-
Frequency of walking during leisure time	Physical activity	3561.00	-0.01	0.01	0.44	0.00	0.49	-	-
Frequency of cycling during leisure time	Physical activity	3000.00	-0.01	0.02	0.39	0.76	0.04	-	-
Frequency of dancing during leisure time	Physical activity	3148.00	0.02	0.02	0.27	0.00	0.45	-	-
Frequency of shoveling snow during leisure time	Physical activity	3494.00	-0.01	0.02	0.44	0.02	0.31	-	-
Frequency of gardening during leisure time	Physical activity	3432.00	-0.02	0.02	0.30	0.68	0.08	-	-
Frequency of hunting or fishing during leisure time	Physical activity	3395.00	-0.01	0.02	0.62	0.00	0.88	-	-
Frequency of picking berries or mushrooms during leisure time	Physical activity	3439.00	-0.06	0.02	0.00	0.82	0.02	-	-

Changed everyday exercise during the last year	Physical activity	3695.00	0.01	0.01	0.40	0.00	0.72	-	-
Everyday exercise satisfaction	Physical activity	3688.00	-0.02	0.01	0.20	0.00	0.86	-	-
Exercise during the last three months	Physical activity	3692.00	0.02	0.01	0.24	0.00	0.39	-	-
If you exercise, change in exercise habits during the last year	Physical activity	3252.00	0.02	0.02	0.24	0.63	0.10	-	-
Bregott on bread	Food	3405.00	0.00	0.01	0.90	0.54	0.14	-	-
Whole grain crisp bread	Food	3405.00	0.00	0.01	0.93	0.00	0.66	-	-
Whole grain soft bread	Food	3405.00	-0.03	0.01	0.07	0.00	0.56	-	-
White (soft) bread, thin crisp bread	Food	3405.00	0.02	0.02	0.22	0.44	0.18	-	-
Coffee rolls/buns, rusk	Food	3405.00	0.00	0.01	0.97	0.04	0.31	-	-
Cheese 28%	Food	3405.00	-0.02	0.02	0.29	0.00	0.80	-	-
Cheese 10-17%	Food	3405.00	0.00	0.01	0.91	0.00	0.38	-	-
Soft cheese	Food	1207.00	-0.02	0.02	0.46	0.00	0.89	-	-
Soft whey cheese	Food	1207.00	-0.04	0.02	0.13	0.00	0.72	-	-
Sausage, liver pate on bread	Food	3405.00	0.00	0.01	0.94	0.00	0.70	-	-
Meat on bread	Food	3405.00	0.02	0.01	0.23	0.41	0.19	-	-
Butter on bread	Food	3405.00	0.01	0.01	0.52	0.78	0.03	-	-
Oatflake, whole wheat, rye or barley porridge	Food	3405.00	-0.01	0.01	0.68	0.00	0.90	-	-
Rosehip, sweet syrup soup	Food	3405.00	0.00	0.02	0.90	0.06	0.30	-	-
Sour milk, yoghurt (3% fat)	Food	3405.00	0.01	0.02	0.61	0.00	0.83	-	-
Sour milk, yoghurt (low fat)	Food	3405.00	0.03	0.01	0.07	0.53	0.15	-	-
Fiber cereals	Food	3405.00	0.02	0.02	0.19	0.00	0.90	-	-
Corn flakes	Food	3405.00	0.01	0.01	0.72	0.00	0.36	-	-
Berries (fresh or frozen)	Food	3405.00	0.00	0.01	0.82	0.00	0.71	-	-
Apple, pear, peach, orange, mandarin and grapefruit	Food	3405.00	-0.03	0.02	0.09	0.00	0.32	-	-
Ba	Food	3405.00	0.02	0.02	0.31	0.00	0.80	-	-
Root vegetables and carrot	Food	3405.00	0.00	0.01	0.87	0.11	0.29	-	-
Low fat margarine on bread	Food	3405.00	0.00	0.01	0.77	0.00	0.95	-	-
Tomato and cucumber	Food	3405.00	0.02	0.02	0.21	0.05	0.30	-	-
White cabbage, lettuce, lettuce cabbage, spich, borecole and broccoli	Food	3405.00	0.00	0.02	0.77	0.33	0.22	-	-
Mixed frozen vegetables	Food	1207.00	0.00	0.03	0.93	0.00	0.95	-	-
Boiled or baked potato	Food	3405.00	-0.03	0.01	0.05	0.00	0.50	-	-
Fried potatoes and pommes frites	Food	3405.00	-0.01	0.01	0.24	0.00	0.61	-	-
Mashed potato	Food	1207.00	-0.01	0.03	0.67	0.57	0.13	-	-
Potato salad	Food	1207.00	-0.05	0.03	0.06	0.00	0.59	-	-
Rice	Food	3405.00	0.00	0.01	1.00	0.70	0.07	-	-
Pasta	Food	3405.00	0.02	0.01	0.12	0.00	0.32	-	-
Brown beans and pea soup	Food	3405.00	0.02	0.02	0.32	0.75	0.05	-	-
Margarine on bread	Food	3405.00	0.00	0.01	0.77	0.00	0.97	-	-
Blota (broth + bread)	Food	1207.00	-0.03	0.03	0.19	0.49	0.16	-	-
Pancake, waffle and Swedish dumpling	Food	3405.00	0.01	0.01	0.47	0.64	0.10	-	-
Pizza	Food	3405.00	-0.01	0.02	0.38	0.00	0.35	-	-
Minced meat dishes	Food	3405.00	-0.01	0.01	0.60	0.00	0.40	-	-
Meat stew	Food	3405.00	0.01	0.01	0.69	0.00	0.84	-	-
Steak, chop, etc.	Food	3405.00	0.00	0.01	0.92	0.00	0.42	-	-
Bacon	Food	3405.00	-0.02	0.01	0.08	0.00	0.45	-	-
Sausage as main dish	Food	3405.00	0.01	0.01	0.47	0.56	0.13	-	-

Hamburger	Food	3405.00	0.00	0.02	0.74	0.87	0.01	-	-
White meat (poultry)	Food	3405.00	0.00	0.02	1.00	0.00	0.71	-	-
Butter for cooking	Food	3405.00	0.01	0.01	0.52	0.00	0.59	-	-
Blood based food	Food	1207.00	0.00	0.02	0.93	0.00	0.83	-	-
Liver and kidney	Food	1207.00	-0.03	0.02	0.21	0.00	0.82	-	-
Lean fish (e.g. perch, bass, cod)	Food	3405.00	0.01	0.01	0.56	0.00	0.51	-	-
Fatty fish (e.g. herring, whitefish, salmon)	Food	3405.00	-0.01	0.02	0.57	0.00	0.52	-	-
Shellfish (e.g. shrimps, scallops)	Food	1207.00	0.02	0.03	0.47	0.00	0.64	-	-
Salty fish	Food	3405.00	0.00	0.01	0.95	0.00	0.41	-	-
Smoked fish/meat	Food	3405.00	0.00	0.01	0.88	0.45	0.18	-	-
Ice cream	Food	3405.00	-0.02	0.02	0.17	0.00	0.59	-	-
Sweets (chocolate and candy)	Food	3405.00	0.01	0.01	0.64	0.21	0.26	-	-
Sugar, honey, marmelade and jam	Food	3405.00	0.01	0.01	0.56	0.20	0.26	-	-
Margarine for cooking	Food	3405.00	-0.02	0.01	0.18	0.00	0.70	-	-
Cookies and pastry	Food	3405.00	0.02	0.01	0.11	0.00	0.85	-	-
Chips, popcorn and salted nuts	Food	3405.00	0.01	0.01	0.41	0.00	0.81	-	-
Low fat milk (0.5%)	Beverage	3405.00	-0.01	0.02	0.50	0.00	0.60	-	-
Milk, sour milk (1.5%)	Beverage	3405.00	0.02	0.01	0.21	0.00	0.54	-	-
Milk, sour milk (3%)	Beverage	3405.00	0.00	0.01	0.83	0.00	0.72	-	-
Sodas, soft drinks and juice	Beverage	3405.00	-0.01	0.01	0.60	0.14	0.28	-	-
Brewed (filtered) coffee	Beverage	3405.00	0.00	0.01	0.80	0.00	0.54	-	-
Boiled coffee	Beverage	3405.00	0.03	0.01	0.01	0.00	0.80	-	-
Tea	Beverage	3405.00	-0.02	0.02	0.16	0.18	0.27	-	-
Light beer	Alcohol	3405.00	-0.01	0.02	0.78	0.00	0.33	-	-
Oil for cooking	Food	3405.00	0.02	0.01	0.16	0.47	0.17	-	-
Medium beer	Alcohol	3405.00	0.03	0.02	0.07	0.00	0.79	-	-
Strong beer	Alcohol	3405.00	0.02	0.01	0.12	0.00	0.62	-	-
Wine	Alcohol	3405.00	-0.01	0.02	0.68	0.64	0.10	-	-
Liquor and spirits	Alcohol	3405.00	-0.01	0.01	0.62	0.00	0.46	-	-
Salad dressing with oil	Food	3405.00	-0.01	0.01	0.40	0.00	0.55	-	-
Cream, creme fraiche, sour cream	Food	3405.00	0.01	0.01	0.62	0.00	0.64	-	-
Average portion size of vegetables based on photographic illustration of four sizes (smallest to largest)	Food	3405.00	-0.01	0.02	0.47	0.00	0.36	-	-
Overall state of health during the last year	General health	3691.00	-0.01	0.01	0.33	0.74	0.05	-	-
Overall state of health compared to others your age	General health	3624.00	0.00	0.01	0.83	0.00	0.43	-	-
Parents or siblings had a cerebral hemorrhage/thrombosis or cardiac infarction before the age of 60	General health	3658.00	0.05	0.04	0.21	0.00	0.42	-	-
Teetotaler	Alcohol	3686.00	-0.02	0.05	0.59	0.00	0.32	-	-
Feel the need to reduce alcohol consumption	Alcohol	3297.00	0.04	0.05	0.44	0.00	0.53	-	-
Feel uneasy or guilty because of your way of drinking	Alcohol	3295.00	0.12	0.05	0.01	0.00	0.70	-	-
Iron intake (mg/day)	Nutrients	3405.00	-0.01	0.02	0.65	0.46	0.18	-	-
Iodine intake (ug/day)	Nutrients	3405.00	0.01	0.01	0.32	0.00	0.42	-	-
Calcium intake (mg/day)	Nutrients	3405.00	0.01	0.01	0.53	0.00	0.88	-	-
Potassium intake (mg/day)	Nutrients	3405.00	-0.01	0.02	0.57	0.00	0.95	-	-

Beta-carotene intake (mg/day)	Nutrients	3405.00	0.00	0.01	0.90	0.18	0.27	-	-
Cholesterol intake (g/day)	Nutrients	3405.00	0.01	0.02	0.58	0.00	0.73	-	-
Carbohydrates intake (g/day)	Nutrients	3405.00	-0.01	0.01	0.34	0.00	0.49	-	-
Average portion size of meat/fish based on photographic illustration of four sizes (smallest to largest)	Food	3405.00	-0.01	0.02	0.68	0.46	0.17	-	-
Breakfast habits: Only coffee/tea for breakfast vs not breakfast at all	Food	272.00	0.60	0.17	0.00	0.00	0.45	-	-
Breakfast habits: Coffee/tea and wheat buns or rusk for breakfast vs not breakfast at all	Food	347.00	-0.10	0.10	0.35	0.00	0.42	-	-
Breakfast habits: Porridge w/o sandwich for breakfast vs not breakfast at all	Food	517.00	-0.09	0.08	0.27	0.29	0.24	-	-
Breakfast habits: Gruel w/o sandwich for breakfast vs not breakfast at all	Food	305.00	-0.04	0.14	0.78	0.00	0.86	-	-
Enterodiol intake (ug/day)	Nutrients	3405.00	-0.01	0.02	0.71	0.00	0.45	-	-
Enterolactone intake (ug/day)	Nutrients	3405.00	0.00	0.02	0.96	0.00	0.59	-	-
Equol intake (ug/day)	Nutrients	3405.00	0.01	0.02	0.66	0.37	0.21	-	-
Lariciresinol intake (ug/day)	Nutrients	3405.00	-0.02	0.01	0.23	0.00	0.53	-	-
Matairesinol intake (ug/day)	Nutrients	3405.00	-0.01	0.01	0.39	0.00	0.66	-	-
Medioresinol intake (ug/day)	Nutrients	3405.00	-0.01	0.01	0.34	0.00	0.48	-	-
Pinoresinol intake (ug/day)	Nutrients	3405.00	-0.02	0.01	0.20	0.00	0.95	-	-
Secoisolariciresinol intake (ug/day)	Nutrients	3405.00	0.01	0.01	0.33	0.00	0.58	-	-
Sum of all ligns intake (ug/day)	Nutrients	3405.00	-0.01	0.01	0.39	0.00	0.52	-	-
Syringaresinol intake (ug/day)	Nutrients	3405.00	-0.01	0.01	0.53	0.00	0.41	-	-
Sum of Lariciresinol, Matairesinol, Pinoresinol, Secoisolariciresinol intake (ug/day)	Nutrients	3405.00	-0.02	0.01	0.29	0.00	0.82	-	-
Satisfaction with home and family situation	Psychosocial	2376.00	-0.01	0.02	0.53	0.00	0.78	-	-
Appetite status	Psychosocial	2375.00	-0.01	0.02	0.68	0.45	0.18	-	-
Mood status	Psychosocial	2370.00	-0.01	0.02	0.45	0.75	0.05	-	-
Energy status	Psychosocial	2374.00	-0.03	0.02	0.06	0.70	0.07	-	-
Patience status	Psychosocial	2374.00	0.00	0.02	0.88	0.00	0.76	-	-
Confidence status	Psychosocial	2372.00	-0.01	0.02	0.54	0.60	0.11	-	-
Sleep status	Sleep	2378.00	0.01	0.02	0.67	0.85	0.01	-	-
Do you feel important and appreciated outside your home?	Psychosocial	2375.00	0.01	0.02	0.75	0.00	0.43	-	-
Do you feel important and appreciated in your home?	Psychosocial	2361.00	-0.01	0.02	0.46	0.42	0.19	-	-
Satisfaction with accomodation	Psychosocial	2375.00	0.02	0.02	0.31	0.70	0.07	-	-
Satisfaction with work situation	Psychosocial	2340.00	-0.02	0.02	0.23	0.00	0.93	-	-

Satisfaction with economy	Psychosocial	2369.00	0.00	0.02	0.83	0.49	0.16	-	-
Satisfaction with leisure time	Psychosocial	2371.00	0.00	0.02	0.95	0.00	0.50	-	-
Hearing status	General health	2380.00	-0.02	0.02	0.22	0.00	0.53	-	-
Vision status	General health	2381.00	-0.03	0.02	0.12	0.00	0.49	-	-
Memory status	Psychosocial	2367.00	-0.02	0.02	0.17	0.57	0.13	-	-
Fitness status	Physical activity	2371.00	-0.01	0.02	0.64	0.42	0.19	-	-
Magnesium intake (mg/day)	Nutrients	3405.00	0.01	0.02	0.37	0.00	0.78	-	-
Saturated fat intake (g/day)	Nutrients	3405.00	0.01	0.01	0.61	0.00	0.89	-	-
Monounsaturated fat intake (g/day)	Nutrients	3405.00	0.01	0.02	0.62	0.00	0.47	-	-
Monosaccharides intake (g/day)	Nutrients	3405.00	-0.02	0.02	0.14	0.00	0.55	-	-
Sodium intake (mg/day)	Nutrients	3405.00	0.00	0.01	0.79	0.29	0.24	-	-
Vitamin B3 intake (mg/day)	Nutrients	3405.00	-0.01	0.01	0.72	0.00	0.60	-	-
Cambridge physical activity index	Physical activity	3617.00	0.01	0.01	0.69	0.00	0.62	-	-
Polyunsaturated fat intake (g/day)	Nutrients	3405.00	0.01	0.01	0.60	0.55	0.14	-	-
Average portion size of potatoes/rice/pasta based on photographic illustration of four sizes (smallest to largest)	Food	3405.00	0.01	0.02	0.67	0.72	0.06	-	-
Total protein intake (g/day)	Nutrients	3405.00	0.01	0.01	0.51	0.00	0.98	-	-
Animal based protein intake (g/day)	Nutrients	3405.00	0.01	0.01	0.42	0.00	0.47	-	-
Plant based protein intake (g/day)	Nutrients	3405.00	-0.01	0.01	0.61	0.67	0.08	-	-
Vitamin A intake (mg/day)	Nutrients	3405.00	-0.03	0.02	0.13	0.05	0.30	-	-
Sucrose intake (g/day)	Nutrients	3405.00	0.01	0.01	0.72	0.71	0.06	-	-
Cohabitation: Live alone vs Only one adult (spouse, partner)	Social	1198.00	-0.03	0.06	0.65	0.81	0.02	-	-
Cohabitation: Live alone vs Only children	Social	498.00	0.13	0.09	0.12	0.72	0.06	-	-
Cohabitation: Live alone vs Adult and children	Social	2578.00	-0.02	0.05	0.69	0.89	0.00	-	-
Cohabitation: Live alone vs Other/others	Social	422.00	-0.11	0.10	0.29	0.00	0.38	-	-
Selenium intake (ug/day)	Nutrients	3405.00	0.01	0.02	0.51	0.00	0.91	-	-
Long-term sickness	General health	3583.00	-0.01	0.04	0.81	0.38	0.20	-	-
Work shifts/weekends	Psychosocial	3570.00	0.03	0.03	0.29	0.85	0.01	-	-
Number of cigarretes smoked per day (in groups)	Tobacco use	3044.00	-0.03	0.01	0.02	0.86	0.01	-	-
Years smoking	Tobacco use	3264.00	-0.02	0.02	0.17	0.90	0.00	-	-
Grams of tobacco smoked per week	Tobacco use	2178.00	0.06	0.03	0.05	0.00	0.64	-	-
Number of cigarretes smoked per day	Tobacco use	2178.00	0.06	0.03	0.05	0.00	0.64	-	-
Number of cigars smoked per day	Tobacco use	2178.00	0.06	0.03	0.05	0.00	0.64	-	-
Smoking status: Smokers vs non-smokers	Tobacco use	2443.00	-0.05	0.04	0.18	0.92	0.00	-	-
Smoking status: Former smokers vs non-smokers	Tobacco use	2565.00	0.00	0.04	0.99	0.69	0.07	-	-

Smoking status: Former occasiol smokers vs non-smokers	Tobacco use	2070.00	0.12	0.05	0.02	0.00	0.95	-	-
Number of snuff boxes per week	Tobacco use	3519.00	0.05	0.01	0.00	0.47	0.17	-	-
Snuff status: Snuff users vs non-snuff users	Tobacco use	3091.00	0.15	0.04	0.00	0.78	0.03	-	-
Snuff status: Former snuff users vs non-snuff users	Tobacco use	2733.00	0.05	0.05	0.28	0.03	0.31	-	-
Years using snuff	Tobacco use	3382.00	0.05	0.01	0.00	0.56	0.13	-	-
Participation in associations or voluntary organisations	Social	3673.00	0.00	0.03	0.99	0.55	0.13	-	-
People to ask for help apart from the ones at home	Social	3682.00	0.00	0.05	0.99	0.00	1.00	-	-
Number of friends that can come to your home at any time and feel at home	Social	3677.00	0.00	0.01	0.93	0.00	0.37	-	-
Number of social contacts with the same interests as you	Social	3683.00	-0.03	0.01	0.02	0.00	0.67	-	-
Would you say that the number of people that you meet in your everyday life is enough or would you like to meet more or fewer people?	Social	3684.00	0.00	0.01	0.96	0.00	0.94	-	-
Close relationship with anyone	Social	3690.00	-0.01	0.01	0.54	0.00	0.36	-	-
Frequency of engaging in clubs, associations or study circles	Social	2461.00	0.00	0.02	0.79	0.40	0.20	-	-
Number of social interactions during a normal week	Social	3683.00	0.00	0.01	0.98	0.00	0.85	-	-
Support from others	Social	3685.00	0.00	0.01	0.89	0.00	0.76	-	-
Number of people with whom you can speak openly	Social	3686.00	0.01	0.01	0.70	0.00	0.47	-	-
Receive hugs to comfort and support you	Social	3660.00	-0.04	0.04	0.30	0.00	0.58	-	-
Stigmasterol intake (mg/day)	Nutrients	3405.00	0.00	0.01	0.96	0.69	0.07	-	-
Tiamin intake (mg/day)	Nutrients	3405.00	0.03	0.02	0.06	0.00	0.97	-	-
Vitamin E intake (mg/day)	Nutrients	3405.00	-0.02	0.01	0.25	0.00	0.43	-	-
Trans fat intake (g/day)	Nutrients	3405.00	-0.05	0.02	0.02	0.00	0.75	-	-
Sum of phytosterols intake (mg/day)	Nutrients	3405.00	-0.01	0.01	0.63	0.02	0.31	-	-
Educatiol level	Psychosocial	3693.00	0.03	0.01	0.03	0.00	0.35	-	-
Zinc intake (mg/day)	Nutrients	3405.00	-0.01	0.02	0.38	0.00	0.98	-	-

Supplementary Table 19. Longitudinal association results for fasting glucose

Description	Group	N	Effect estimate	S.E.	p-value	I^2	Q p-value	adjusted R2	R2 rank
Alcohol intake (g/day)	Alcohol	22495.00	0.01	0.01	0.04	0.00	0.96	-	-
Permanent employment	Psychosocial	23886.00	0.01	0.01	0.57	0.29	0.23	-	-
Self-employed	Psychosocial	23886.00	-0.04	0.02	0.03	0.43	0.18	-	-
Distance to work in kilometers (one way)	Physical activity	21498.00	0.02	0.01	0.00	0.71	0.06	-	-
Last time a colleague visited you at home	Psychosocial	23764.00	0.01	0.01	0.04	0.51	0.15	-	-
Job demands to work very fast	Psychosocial	24052.00	0.01	0.01	0.19	0.34	0.22	-	-
Frequency of social contacts with colleagues during leisure time	Psychosocial	23477.00	-0.01	0.01	0.29	0.00	0.58	-	-
High physical demand from job	Physical activity	24113.00	0.00	0.01	0.43	0.82	0.02	-	-
Enough time for job assignments	Psychosocial	23962.00	0.01	0.01	0.07	0.00	0.79	-	-
Control over planning and execution of the workday	Psychosocial	24082.00	0.01	0.01	0.14	0.29	0.24	-	-
Ingenuity or creativity demand from job	Psychosocial	23969.00	-0.01	0.01	0.07	0.69	0.07	-	-
Frequent social contacts with colleagues during work	Psychosocial	23856.00	-0.01	0.01	0.05	0.13	0.28	-	-
Contradictory demands in job	Psychosocial	23890.00	0.00	0.01	0.45	0.00	0.65	-	-
Possibility to leave your work for a while to speak with a colleague	Psychosocial	23878.00	0.00	0.01	0.67	0.00	0.81	-	-
Learn new things at job	Psychosocial	24024.00	0.00	0.01	0.65	0.00	0.68	-	-
High mental demand from job	Psychosocial	23892.00	0.01	0.01	0.12	0.00	0.74	-	-
Repetitive job	Psychosocial	24056.00	0.01	0.01	0.25	0.00	0.42	-	-
Skill demand from job	Psychosocial	24012.00	0.00	0.01	0.46	0.00	0.52	-	-
Possibility to speak with colleagues during breaks	Psychosocial	24008.00	-0.01	0.01	0.28	0.00	0.82	-	-
Control over own work assignment	Psychosocial	24068.00	0.01	0.01	0.06	0.55	0.14	-	-
Vitamin C intake (mg/day)	Nutrients	22495.00	0.00	0.01	0.53	0.00	0.70	-	-
Vitamin B12 intake (ug/day)	Nutrients	22495.00	0.02	0.01	0.00	0.46	0.17	-	-
Vitamin B2 intake (ug/day)	Nutrients	22495.00	0.00	0.01	0.68	0.35	0.21	-	-
Vitamin B6 intake (mg/day)	Nutrients	22495.00	0.01	0.01	0.07	0.17	0.27	-	-
Informed of having high blood pressure	General health	24373.00	0.04	0.01	0.01	0.00	0.85	-	-
Beta-sitostanol intake (mg/day)	Nutrients	22495.00	0.00	0.01	0.39	0.00	0.90	-	-
Beta-sitosterol intake (mg/day)	Nutrients	22495.00	-0.01	0.01	0.34	0.50	0.16	-	-
Marital status: Single vs Married/partner	Social	22595.00	-0.03	0.02	0.11	0.00	0.57	-	-
Marital status: Single vs Divorced/separated	Social	3945.00	0.02	0.03	0.52	0.00	0.93	-	-
Marital status: Single vs Widow/widower	Social	2534.00	-0.04	0.08	0.63	0.00	0.63	-	-
Campestanol intake (mg/day)	Nutrients	22495.00	-0.01	0.01	0.32	0.00	0.92	-	-
Campesterol intake (mg/day)	Nutrients	22495.00	0.00	0.01	0.85	0.78	0.03	-	-
Parents or siblings have diabetes	General health	24171.00	0.09	0.01	0.00	0.00	0.43	0.09	4.00

Disaccharides intake (g/day)	Nutrients	22495.00	0.00	0.01	0.64	0.00	0.79	-	-
Vitamin D intake (ug/day)	Nutrients	22495.00	0.01	0.01	0.01	0.82	0.02	-	-
Total energy intake (kcal/day)	Nutrients	22495.00	0.01	0.01	0.29	0.00	0.59	-	-
Formic acid intaje (g/day)	Nutrients	22495.00	0.00	0.01	0.80	0.00	0.68	-	-
Pentadecanoic acid intake (g/day)	Nutrients	22495.00	0.00	0.01	0.66	0.04	0.31	-	-
Palmitic acid intake (g/day)	Nutrients	22495.00	0.01	0.01	0.11	0.00	0.60	-	-
Heptadecanoic acid intake (g/day)	Nutrients	22495.00	0.00	0.01	0.66	0.04	0.31	-	-
Linoleic acid intake (g/day)	Nutrients	22495.00	0.00	0.01	0.48	0.00	0.85	-	-
Linolenic acid intake (g/day)	Nutrients	22495.00	0.01	0.01	0.22	0.36	0.21	-	-
Arachidonic acid (ARA) intake (g/day)	Nutrients	22495.00	0.02	0.01	0.00	0.58	0.12	-	-
Eicosapentaenoic acid (EPA) intake (g/day)	Nutrients	22495.00	0.01	0.01	0.01	0.00	0.92	-	-
Docosahexaenoic acid (DHA) intake (g/day)	Nutrients	22495.00	0.01	0.01	0.01	0.00	0.79	-	-
Fat intake (g/day)	Nutrients	22495.00	0.01	0.01	0.02	0.00	0.61	-	-
Fibre intake (g/day)	Nutrients	22495.00	-0.01	0.01	0.06	0.00	0.46	-	-
Folic acid intake (ug/day)	Nutrients	22495.00	0.00	0.01	0.65	0.00	0.83	-	-
Phosphate intake (mg/day)	Nutrients	22495.00	0.00	0.01	0.68	0.65	0.09	-	-
Whole grain intake (g/day)	Food	22495.00	-0.02	0.01	0.00	0.00	0.82	-	-
Travel to work: Walk to work vs passive travel to work	Physical activity	15740.00	-0.01	0.02	0.70	0.00	0.44	-	-
Travel to work: Cycle to work vs passive travel to work	Physical activity	18928.00	0.00	0.01	0.89	0.66	0.08	-	-
Travel to work: Irregular travel mode to work vs passive travel to work	Physical activity	16039.00	0.01	0.02	0.72	0.00	0.41	-	-
Sedentary or standing work	Physical activity	23970.00	0.01	0.01	0.29	0.00	0.89	-	-
Light but partly physically active work	Physical activity	23970.00	-0.01	0.01	0.43	0.81	0.02	-	-
Light and physically active work	Physical activity	23970.00	-0.02	0.01	0.14	0.00	0.49	-	-
Sometimes physically straining work	Physical activity	23970.00	0.01	0.01	0.35	0.49	0.16	-	-
Frequency of walking during leisure time	Physical activity	23729.00	0.00	0.01	0.42	0.00	0.98	-	-
Frequency of cycling during leisure time	Physical activity	20902.00	0.00	0.01	0.49	0.00	0.81	-	-
Frequency of dancing during leisure time	Physical activity	21653.00	-0.01	0.01	0.07	0.80	0.03	-	-
Frequency of shoveling snow during leisure time	Physical activity	23319.00	0.00	0.01	0.86	0.00	0.75	-	-
Frequency of gardening during leisure time	Physical activity	23120.00	0.01	0.01	0.07	0.00	0.58	-	-
Frequency of hunting or fishing during leisure time	Physical activity	22680.00	0.01	0.01	0.24	0.00	0.50	-	-
Frequency of picking berries or mushrooms during leisure time	Physical activity	23003.00	0.02	0.01	0.00	0.00	0.84	-	-
Changed everyday exercise during the last year	Physical activity	24366.00	0.00	0.01	0.47	0.29	0.24	-	-

Everyday exercise satisfaction	Physical activity	24298.00	0.01	0.01	0.02	0.37	0.21	-	-
Exercise during the last three months	Physical activity	24360.00	-0.02	0.01	0.00	0.00	0.55	-	-
If you exercise, change in exercise habits during the last year	Physical activity	21337.00	0.00	0.01	0.86	0.00	0.79	-	-
Bregott on bread	Food	22495.00	0.00	0.01	0.72	0.00	0.95	-	-
Whole grain crisp bread	Food	22495.00	0.00	0.01	0.52	0.00	0.42	-	-
Whole grain soft bread	Food	22495.00	0.00	0.01	0.49	0.00	0.87	-	-
White (soft) bread, thin crisp bread	Food	22495.00	0.00	0.01	0.39	0.61	0.11	-	-
Coffee rolls/buns, rusk	Food	22495.00	0.00	0.01	0.74	0.00	0.46	-	-
Cheese 28%	Food	22495.00	-0.01	0.01	0.31	0.48	0.17	-	-
Cheese 10-17%	Food	22495.00	0.00	0.01	0.56	0.00	0.71	-	-
Soft cheese	Food	10063.00	0.01	0.01	0.53	0.00	0.37	-	-
Soft whey cheese	Food	10063.00	-0.02	0.01	0.01	0.00	0.35	-	-
Sausage, liver pate on bread	Food	22495.00	0.02	0.01	0.00	0.74	0.05	-	-
Meat on bread	Food	22495.00	0.00	0.01	0.41	0.00	0.75	-	-
Butter on bread	Food	22495.00	0.00	0.01	0.48	0.49	0.16	-	-
Oatflake, whole wheat, rye or barley porridge	Food	22495.00	-0.02	0.01	0.00	0.00	0.45	-	-
Rosehip, sweet syrup soup	Food	22495.00	0.00	0.01	0.79	0.00	0.62	-	-
Sour milk, yoghurt (3% fat)	Food	22495.00	-0.01	0.01	0.01	0.00	0.89	-	-
Sour milk, yoghurt (low fat)	Food	22495.00	-0.01	0.01	0.13	0.00	0.38	-	-
Fiber cereals	Food	22495.00	-0.02	0.01	0.00	0.00	0.68	-	-
Corn flakes	Food	22495.00	-0.01	0.01	0.18	0.00	0.65	-	-
Berries (fresh or frozen)	Food	22495.00	-0.01	0.01	0.33	0.72	0.06	-	-
Apple, pear, peach, orange, mandarin and grapefruit	Food	22495.00	-0.01	0.01	0.02	0.00	0.41	-	-
Ba	Food	22495.00	0.01	0.01	0.21	0.13	0.28	-	-
Root vegetables and carrot	Food	22495.00	-0.01	0.01	0.34	0.00	0.85	-	-
Low fat margarine on bread	Food	22495.00	0.00	0.01	0.76	0.56	0.13	-	-
Tomato and cucumber	Food	22495.00	0.01	0.01	0.27	0.00	0.72	-	-
White cabbage, lettuce, lettuce cabbage, spich, borecole and broccoli	Food	22495.00	0.00	0.01	0.92	0.00	0.97	-	-
Mixed frozen vegetables	Food	10063.00	0.00	0.01	0.53	0.00	0.46	-	-
Boiled or baked potato	Food	22495.00	0.00	0.01	0.94	0.00	0.84	-	-
Fried potatoes and pommes frites	Food	22495.00	0.01	0.01	0.29	0.00	0.86	-	-
Mashed potato	Food	10063.00	0.01	0.01	0.51	0.00	0.64	-	-
Potato salad	Food	10063.00	-0.01	0.01	0.51	0.68	0.08	-	-
Rice	Food	22495.00	-0.01	0.01	0.10	0.50	0.16	-	-
Pasta	Food	22495.00	-0.01	0.01	0.36	0.08	0.30	-	-
Brown beans and pea soup	Food	22495.00	0.00	0.01	0.73	0.00	0.80	-	-
Margarine on bread	Food	22495.00	0.00	0.01	0.86	0.00	0.41	-	-
Blota (broth + bread)	Food	10063.00	0.00	0.01	0.99	0.00	0.80	-	-
Pancake, waffle and Swedish dumpling	Food	22495.00	-0.01	0.01	0.02	0.00	0.65	-	-
Pizza	Food	22495.00	0.00	0.01	0.61	0.00	0.47	-	-
Minced meat dishes	Food	22495.00	0.02	0.01	0.00	0.00	0.39	-	-
Meat stew	Food	22495.00	0.00	0.01	0.45	0.82	0.02	-	-
Steak, chop, etc.	Food	22495.00	0.01	0.01	0.03	0.19	0.27	-	-
Bacon	Food	22495.00	0.01	0.01	0.01	0.00	0.84	-	-

Sausage as main dish	Food	22495.00	0.01	0.01	0.09	0.00	0.88	-	-
Hamburger	Food	22495.00	0.01	0.01	0.02	0.00	0.71	-	-
White meat (poultry)	Food	22495.00	0.01	0.01	0.21	0.00	0.94	-	-
Butter for cooking	Food	22495.00	0.00	0.01	0.71	0.48	0.16	-	-
Blood based food	Food	10063.00	0.01	0.01	0.41	0.37	0.21	-	-
Liver and kidney	Food	10063.00	0.01	0.01	0.52	0.00	0.49	-	-
Lean fish (e.g. perch, bass, cod)	Food	22495.00	0.00	0.01	0.91	0.00	0.81	-	-
Fatty fish (e.g. herring, whitefish, salmon)	Food	22495.00	0.02	0.01	0.00	0.00	0.44	-	-
Shellfish (e.g. shrimps, scallops)	Food	10063.00	0.01	0.01	0.11	0.00	0.53	-	-
Salty fish	Food	22495.00	0.00	0.01	0.35	0.00	0.72	-	-
Smoked fish/meat	Food	22495.00	0.00	0.01	0.56	0.00	0.93	-	-
Ice cream	Food	22495.00	0.01	0.01	0.20	0.86	0.01	-	-
Sweets (chocolate and candy)	Food	22495.00	0.00	0.01	0.92	0.00	0.92	-	-
Sugar, honey, marmelade and jam	Food	22495.00	-0.01	0.01	0.04	0.00	0.44	-	-
Margarine for cooking	Food	22495.00	0.01	0.01	0.05	0.84	0.01	-	-
Cookies and pastry	Food	22495.00	-0.02	0.01	0.00	0.00	0.67	-	-
Chips, popcorn and salted nuts	Food	22495.00	0.01	0.01	0.17	0.00	0.36	-	-
Low fat milk (0.5%)	Beverage	22495.00	0.00	0.01	1.00	0.00	0.91	-	-
Milk, sour milk (1.5%)	Beverage	22495.00	0.00	0.01	0.62	0.17	0.27	-	-
Milk, sour milk (3%)	Beverage	22495.00	0.01	0.01	0.05	0.58	0.12	-	-
Sodas, soft drinks and juice	Beverage	22495.00	0.01	0.01	0.02	0.00	0.65	-	-
Brewed (filtered) coffee	Beverage	22495.00	0.01	0.01	0.30	0.61	0.11	-	-
Boiled coffee	Beverage	22495.00	0.00	0.01	0.90	0.58	0.12	-	-
Tea	Beverage	22495.00	-0.01	0.01	0.01	0.00	0.78	-	-
Light beer	Alcohol	22495.00	0.01	0.01	0.23	0.00	0.58	-	-
Oil for cooking	Food	22495.00	0.00	0.01	0.73	0.00	0.83	-	-
Medium beer	Alcohol	22495.00	0.01	0.01	0.08	0.80	0.02	-	-
Strong beer	Alcohol	22495.00	0.00	0.01	0.48	0.00	0.46	-	-
Wine	Alcohol	22495.00	0.01	0.01	0.19	0.00	0.48	-	-
Liquor and spirits	Alcohol	22495.00	0.02	0.01	0.01	0.66	0.08	-	-
Salad dressing with oil	Food	22495.00	0.00	0.01	0.87	0.00	0.97	-	-
Cream, creme fraiche, sour cream	Food	22495.00	-0.01	0.01	0.05	0.58	0.12	-	-
Average portion size of vegetables based on photographic illustration of four sizes (smallest to largest)	Food	22495.00	-0.01	0.01	0.01	0.00	0.42	-	-
Overall state of health during the last year	General health	24347.00	-0.01	0.01	0.06	0.66	0.08	-	-
Overall state of health compared to others your age	General health	24014.00	-0.01	0.01	0.06	0.75	0.04	-	-
Parents or siblings had a cerebral hemorrhage/thrombosis or cardiac infarction before the age of 60	General health	24127.00	0.01	0.01	0.62	0.00	0.63	-	-
Teetotaler	Alcohol	24336.00	-0.02	0.02	0.28	0.00	0.73	-	-
Feel the need to reduce alcohol consumption	Alcohol	22105.00	0.04	0.02	0.03	0.00	0.43	-	-
Feel uneasy or guilty because of your way of drinking	Alcohol	22093.00	0.03	0.02	0.09	0.00	0.90	-	-
Iron intake (mg/day)	Nutrients	22495.00	0.00	0.01	0.58	0.00	0.59	-	-
Iodine intake (ug/day)	Nutrients	22495.00	0.00	0.01	0.46	0.27	0.24	-	-
Calcium intake (mg/day)	Nutrients	22495.00	-0.01	0.01	0.23	0.00	0.51	-	-

Potassium intake (mg/day)	Nutrients	22495.00	0.00	0.01	0.93	0.00	0.59	-	-
Beta-carotene intake (mg/day)	Nutrients	22495.00	0.00	0.01	0.36	0.00	0.76	-	-
Cholesterol intake (g/day)	Nutrients	22495.00	0.01	0.01	0.02	0.28	0.24	-	-
Carbohydrates intake (g/day)	Nutrients	22495.00	-0.01	0.01	0.01	0.00	0.80	-	-
Average portion size of meat/fish based on photographic illustration of four sizes (smallest to largest)	Food	22495.00	0.01	0.01	0.08	0.72	0.06	-	-
Breakfast habits: Only coffee/tea for breakfast vs not breakfast at all	Food	1534.00	-0.37	0.07	0.00	0.11	0.29	-	-
Breakfast habits: Coffee/tea and wheat buns or rusk for breakfast vs not breakfast at all	Food	1909.00	-0.06	0.05	0.17	0.00	0.73	-	-
Breakfast habits: Porridge w/o sandwich for breakfast vs not breakfast at all	Food	3303.00	-0.09	0.03	0.00	0.00	0.34	-	-
Breakfast habits: Gruel w/o sandwich for breakfast vs not breakfast at all	Food	1806.00	-0.08	0.05	0.10	0.00	0.72	-	-
Eat breakfast from 2000	Food	2260.00	-0.03	0.06	0.65	0.43	0.18	-	-
Enterodiol intake (ug/day)	Nutrients	22495.00	0.01	0.01	0.21	0.00	0.38	-	-
Enterolactone intake (ug/day)	Nutrients	22495.00	0.00	0.01	0.70	0.00	0.49	-	-
Equol intake (ug/day)	Nutrients	22495.00	0.00	0.01	0.43	0.00	0.71	-	-
Lariciresinol intake (ug/day)	Nutrients	22495.00	-0.01	0.01	0.02	0.32	0.23	-	-
Matairesinol intake (ug/day)	Nutrients	22495.00	-0.01	0.01	0.07	0.00	0.60	-	-
Medioresinol intake (ug/day)	Nutrients	22495.00	0.00	0.01	0.67	0.00	0.53	-	-
Pinoresinol intake (ug/day)	Nutrients	22495.00	-0.01	0.01	0.04	0.27	0.24	-	-
Secoisolariciresinol intake (ug/day)	Nutrients	22495.00	0.00	0.01	0.38	0.00	0.80	-	-
Sum of all ligns intake (ug/day)	Nutrients	22495.00	-0.01	0.01	0.17	0.00	0.47	-	-
Syringaresinol intake (ug/day)	Nutrients	22495.00	-0.01	0.01	0.27	0.00	0.67	-	-
Sum of Lariciresinol, Matairesinol, Pinoresinol, Secoisolariciresinol intake (ug/day)	Nutrients	22495.00	-0.01	0.01	0.03	0.22	0.26	-	-
Satisfaction with home and family situation	Psychosocial	13295.00	0.01	0.01	0.40	0.04	0.31	-	-
Appetite status	Psychosocial	13284.00	0.01	0.01	0.38	0.00	0.72	-	-
Mood status	Psychosocial	13283.00	0.01	0.01	0.29	0.00	0.56	-	-
Energy status	Psychosocial	13272.00	0.01	0.01	0.27	0.00	0.79	-	-
Patience status	Psychosocial	13286.00	0.00	0.01	0.76	0.00	0.64	-	-
Confidence status	Psychosocial	13281.00	0.00	0.01	0.99	0.00	0.64	-	-
Sleep status	Sleep	13309.00	-0.01	0.01	0.25	0.00	0.32	-	-
Do you feel important and appreciated outside your home?	Psychosocial	13302.00	0.00	0.01	0.89	0.49	0.16	-	-

Do you feel important and appreciated in your home?	Psychosocial	13196.00	0.00	0.01	0.53	0.65	0.09	-	-
Satisfaction with accomodation	Psychosocial	13303.00	0.00	0.01	0.99	0.11	0.29	-	-
Satisfaction with work situation	Psychosocial	13166.00	0.00	0.01	0.62	0.00	0.69	-	-
Satisfaction with economy	Psychosocial	13286.00	0.01	0.01	0.12	0.00	0.98	-	-
Satisfaction with leisure time	Psychosocial	13277.00	0.01	0.01	0.27	0.00	0.71	-	-
Hearing status	General health	13300.00	0.00	0.01	0.87	0.00	0.69	-	-
Vision status	General health	13287.00	-0.01	0.01	0.27	0.00	0.61	-	-
Memory status	Psychosocial	13263.00	0.01	0.01	0.20	0.00	0.73	-	-
Fitness status	Physical activity	13278.00	0.00	0.01	0.76	0.00	0.64	-	-
Magnesium intake (mg/day)	Nutrients	22495.00	0.00	0.01	0.57	0.40	0.20	-	-
Saturated fat intake (g/day)	Nutrients	22495.00	0.01	0.01	0.24	0.00	0.99	-	-
Monounsaturated fat intake (g/day)	Nutrients	22495.00	0.02	0.01	0.00	0.00	0.44	-	-
Monosaccharides intake (g/day)	Nutrients	22495.00	-0.01	0.01	0.36	0.00	0.56	-	-
Sodium intake (mg/day)	Nutrients	22495.00	0.01	0.01	0.24	0.27	0.24	-	-
Vitamin B3 intake (mg/day)	Nutrients	22495.00	0.01	0.01	0.05	0.48	0.16	-	-
Cambridge physical activity index	Physical activity	23936.00	-0.01	0.01	0.02	0.00	0.72	-	-
Polyunsaturated fat intake (g/day)	Nutrients	22495.00	0.01	0.01	0.09	0.00	0.54	-	-
Average portion size of potatoes/rice/pasta based on photographic illustration of four sizes (smallest to largest)	Food	22495.00	-0.02	0.01	0.00	0.43	0.19	-	-
Total protein intake (g/day)	Nutrients	22495.00	0.01	0.01	0.14	0.65	0.09	-	-
Animal based protein intake (g/day)	Nutrients	22495.00	0.01	0.01	0.02	0.45	0.18	-	-
Plant based protein intake (g/day)	Nutrients	22495.00	-0.01	0.01	0.01	0.00	0.63	-	-
Vitamin A intake (mg/day)	Nutrients	22495.00	0.02	0.01	0.01	0.00	0.83	-	-
Sucrose intake (g/day)	Nutrients	22495.00	0.00	0.01	0.73	0.00	0.35	-	-
Cohabitation: Live alone vs Only one adult (spouse, partner)	Social	7416.00	-0.02	0.02	0.29	0.00	0.86	-	-
Cohabitation: Live alone vs Only children	Social	3321.00	-0.01	0.04	0.88	0.36	0.21	-	-
Cohabitation: Live alone vs Adult and children	Social	17405.00	-0.03	0.02	0.07	0.00	0.79	-	-
Cohabitation: Live alone vs Other/others	Social	2487.00	0.03	0.06	0.65	0.31	0.23	-	-
Selenium intake (ug/day)	Nutrients	22495.00	0.01	0.01	0.01	0.00	0.44	-	-
Long-term sickness	General health	23519.00	0.02	0.02	0.19	0.00	0.71	-	-
Work shifts/weekends	Psychosocial	23617.00	-0.02	0.01	0.08	0.56	0.13	-	-
Number of cigarretes smoked per day (in groups)	Tobacco use	20419.00	0.05	0.01	0.00	0.55	0.14	0.09	2.00
Years smoking	Tobacco use	21534.00	0.05	0.01	0.00	0.00	0.83	0.09	3.00
Grams of tobacco smoked per week	Tobacco use	15027.00	-0.05	0.01	0.00	0.00	0.51	-	-
Number of cigarretes smoked per day	Tobacco use	15027.00	-0.05	0.01	0.00	0.00	0.51	-	-
Number of cigars smoked per day	Tobacco use	15027.00	-0.05	0.01	0.00	0.00	0.51	-	-

Smoking status: Smokers vs non-smokers	Tobacco use	16022.00	0.15	0.01	0.00	0.00	0.56	0.09	1.00
Smoking status: Former smokers vs non-smokers	Tobacco use	16778.00	0.03	0.01	0.02	0.00	0.85	-	-
Smoking status: Former occasiol smokers vs non-smokers	Tobacco use	14005.00	0.02	0.02	0.35	0.00	0.53	-	-
Number of snuff boxes per week	Tobacco use	23058.00	0.00	0.01	0.97	0.00	0.88	-	-
Snuff status: Snuff users vs non-snuff users	Tobacco use	20563.00	0.00	0.02	0.96	0.00	0.52	-	-
Snuff status: Former snuff users vs non-snuff users	Tobacco use	18819.00	0.02	0.02	0.34	0.05	0.31	-	-
Years using snuff	Tobacco use	22423.00	-0.01	0.01	0.10	0.00	0.59	-	-
Participation in associations or voluntary organisations	Social	24291.00	-0.04	0.01	0.00	0.00	0.75	-	-
Participation in sports or physical exercise associations	Social	1443.00	-0.05	0.04	0.22	0.47	0.17	-	-
Participation in study circles	Social	1443.00	0.07	0.06	0.21	0.00	0.90	-	-
Participation in other association	Social	1443.00	0.03	0.04	0.41	0.00	0.45	-	-
People to ask for help apart from the ones at home	Social	24278.00	0.01	0.02	0.64	0.00	0.80	-	-
Number of friends that can come to your home at any time and feel at home	Social	24272.00	0.01	0.01	0.04	0.00	0.32	-	-
Number of social contacts with the same interests as you	Social	24273.00	0.00	0.01	0.52	0.00	0.54	-	-
Would you say that the number of people that you meet in your everyday life is enough or would you like to meet more or fewer people?	Social	24304.00	-0.01	0.01	0.16	0.00	0.54	-	-
Close relationship with anyone	Social	24332.00	-0.01	0.01	0.18	0.52	0.15	-	-
Frequency of engaging in clubs, associations or study circles	Social	16269.00	-0.01	0.01	0.02	0.00	0.45	-	-
Number of social interactions during a normal week	Social	24327.00	0.01	0.01	0.04	0.00	0.59	-	-
Support from others	Social	24327.00	-0.01	0.01	0.08	0.00	0.88	-	-
Number of people with whom you can speak openly	Social	24310.00	0.00	0.01	0.56	0.12	0.29	-	-
Receive hugs to comfort and support you	Social	24211.00	-0.01	0.01	0.62	0.00	0.73	-	-
Stigmasterol intake (mg/day)	Nutrients	22495.00	0.00	0.01	0.56	0.00	0.54	-	-
Tiamin intake (mg/day)	Nutrients	22495.00	0.00	0.01	0.81	0.00	0.99	-	-
Vitamin E intake (mg/day)	Nutrients	22495.00	0.01	0.01	0.26	0.28	0.24	-	-
Trans fat intake (g/day)	Nutrients	22495.00	0.01	0.01	0.34	0.08	0.30	-	-

Sum of phytosterols intake (mg/day)	Nutrients	22495.00	0.00	0.01	0.53	0.62	0.11	-	-
Educatiol level	Psychosocial	24350.00	-0.03	0.01	0.00	0.00	0.43	0.09	5.00
Zinc intake (mg/day)	Nutrients	22495.00	0.00	0.01	0.85	0.38	0.20	-	-

Supplementary Table 20. Longitudinal association results for 2h glucose

Description	Group	N	Effect estimate	S.E.	p-value	I^2	Q p-value	adjusted R2	R2 rank
Alcohol intake (g/day)	Alcohol	21079.00	-0.01	0.01	0.33	0.00	0.69	-	-
Permanent employment	Psychosocial	22349.00	0.02	0.02	0.35	0.00	0.61	-	-
Self-employed	Psychosocial	22349.00	-0.03	0.03	0.31	0.82	0.02	-	-
Distance to work in kilometers (one way)	Physical activity	20160.00	0.03	0.01	0.01	0.00	0.39	-	-
Last time a colleague visited you at home	Psychosocial	22247.00	0.00	0.01	1.00	0.34	0.22	-	-
Job demands to work very fast	Psychosocial	22502.00	-0.01	0.01	0.44	0.45	0.18	-	-
Frequency of social contacts with colleagues during leisure time	Psychosocial	21989.00	-0.01	0.01	0.49	0.12	0.29	-	-
High physical demand from job	Physical activity	22556.00	-0.01	0.01	0.18	0.00	0.79	-	-
Enough time for job assignments	Psychosocial	22422.00	0.01	0.01	0.27	0.00	0.52	-	-
Control over planning and execution of the workday	Psychosocial	22529.00	0.00	0.01	0.99	0.00	0.49	-	-
Ingenuity or creativity demand from job	Psychosocial	22423.00	-0.02	0.01	0.05	0.37	0.21	-	-
Frequent social contacts with colleagues during work	Psychosocial	22328.00	0.00	0.01	0.63	0.00	0.42	-	-
Contradictory demands in job	Psychosocial	22358.00	-0.02	0.01	0.09	0.00	0.69	-	-
Possibility to leave your work for a while to speak with a colleague	Psychosocial	22354.00	0.02	0.01	0.02	0.00	0.61	-	-
Learn new things at job	Psychosocial	22476.00	0.00	0.01	0.70	0.00	0.76	-	-
High mental demand from job	Psychosocial	22356.00	0.03	0.01	0.00	0.00	0.69	-	-
Repetitive job	Psychosocial	22506.00	0.02	0.01	0.03	0.00	0.38	-	-
Skill demand from job	Psychosocial	22468.00	-0.03	0.01	0.01	0.00	0.64	-	-
Possibility to speak with colleagues during breaks	Psychosocial	22460.00	0.01	0.01	0.18	0.00	0.98	-	-
Control over own work assignment	Psychosocial	22515.00	0.01	0.01	0.27	0.00	0.58	-	-
Vitamin C intake (mg/day)	Nutrients	21079.00	0.00	0.01	0.86	0.29	0.24	-	-
Vitamin B12 intake (ug/day)	Nutrients	21079.00	0.02	0.01	0.08	0.00	0.62	-	-
Vitamin B2 intake (ug/day)	Nutrients	21079.00	0.01	0.01	0.26	0.00	0.65	-	-
Vitamin B6 intake (mg/day)	Nutrients	21079.00	0.01	0.01	0.32	0.00	0.72	-	-
Informed of having high blood pressure	General health	22791.00	0.20	0.03	0.00	0.00	0.43	0.18	2.00
Beta-sitostanol intake (mg/day)	Nutrients	21079.00	0.01	0.01	0.55	0.00	0.50	-	-
Beta-sitosterol intake (mg/day)	Nutrients	21079.00	-0.01	0.01	0.25	0.00	0.41	-	-
Marital status: Single vs Married/partner	Social	21124.00	-0.10	0.03	0.00	0.05	0.30	-	-
Marital status: Single vs Divorced/separated	Social	3654.00	-0.10	0.05	0.05	0.67	0.08	-	-
Marital status: Single vs Widow/widower	Social	2326.00	-0.01	0.14	0.93	0.54	0.14	-	-
Campestanol intake (mg/day)	Nutrients	21079.00	0.01	0.01	0.45	0.00	0.53	-	-
Campesterol intake (mg/day)	Nutrients	21079.00	-0.01	0.01	0.48	0.47	0.17	-	-
Parents or siblings have diabetes	General health	22602.00	0.17	0.03	0.00	0.67	0.08	0.18	3.00

Disaccharides intake (g/day)	Nutrients	21079.00	0.02	0.01	0.05	0.16	0.28	-	-
Vitamin D intake (ug/day)	Nutrients	21079.00	0.01	0.01	0.47	0.37	0.21	-	-
Total energy intake (kcal/day)	Nutrients	21079.00	-0.03	0.01	0.00	0.44	0.18	-	-
Formic acid intaje (g/day)	Nutrients	21079.00	0.00	0.01	0.89	0.00	0.73	-	-
Pentadecanoic acid intake (g/day)	Nutrients	21079.00	0.00	0.01	0.87	0.00	0.59	-	-
Palmitic acid intake (g/day)	Nutrients	21079.00	0.00	0.01	0.90	0.00	0.64	-	-
Heptadecanoic acid intake (g/day)	Nutrients	21079.00	0.00	0.01	0.87	0.00	0.59	-	-
Linoleic acid intake (g/day)	Nutrients	21079.00	0.00	0.01	0.65	0.00	0.77	-	-
Linolenic acid intake (g/day)	Nutrients	21079.00	0.00	0.01	0.84	0.00	0.78	-	-
Arachidonic acid (ARA) intake (g/day)	Nutrients	21079.00	0.00	0.01	0.80	0.00	0.88	-	-
Eicosapentaenoic acid (EPA) intake (g/day)	Nutrients	21079.00	0.00	0.01	0.68	0.00	0.62	-	-
Docosahexaenoic acid (DHA) intake (g/day)	Nutrients	21079.00	-0.01	0.01	0.50	0.00	0.52	-	-
Fat intake (g/day)	Nutrients	21079.00	0.00	0.01	0.81	0.00	0.99	-	-
Fibre intake (g/day)	Nutrients	21079.00	-0.01	0.01	0.58	0.00	0.90	-	-
Folic acid intake (ug/day)	Nutrients	21079.00	-0.01	0.01	0.53	0.00	0.99	-	-
Phosphate intake (mg/day)	Nutrients	21079.00	0.00	0.01	0.90	0.00	0.62	-	-
Whole grain intake (g/day)	Food	21079.00	-0.02	0.01	0.08	0.00	0.92	-	-
Travel to work: Walk to work vs passive travel to work	Physical activity	14738.00	-0.04	0.04	0.33	0.49	0.16	-	-
Travel to work: Cycle to work vs passive travel to work	Physical activity	17742.00	-0.06	0.03	0.02	0.45	0.18	-	-
Travel to work: Irregular travel mode to work vs passive travel to work	Physical activity	15013.00	-0.04	0.04	0.23	0.43	0.18	-	-
Sedentary or standing work	Physical activity	22433.00	0.01	0.02	0.55	0.00	0.56	-	-
Light but partly physically active work	Physical activity	22433.00	0.04	0.03	0.15	0.41	0.19	-	-
Light and physically active work	Physical activity	22433.00	-0.01	0.02	0.70	0.00	0.86	-	-
Sometimes physically straining work	Physical activity	22433.00	0.00	0.02	0.85	0.00	0.53	-	-
Frequency of walking during leisure time	Physical activity	22205.00	0.01	0.01	0.15	0.00	0.70	-	-
Frequency of cycling during leisure time	Physical activity	19564.00	-0.02	0.01	0.02	0.77	0.04	-	-
Frequency of dancing during leisure time	Physical activity	20264.00	0.00	0.01	0.78	0.28	0.24	-	-
Frequency of shoveling snow during leisure time	Physical activity	21827.00	-0.02	0.01	0.13	0.44	0.18	-	-
Frequency of gardening during leisure time	Physical activity	21626.00	-0.01	0.01	0.43	0.00	0.57	-	-
Frequency of hunting or fishing during leisure time	Physical activity	21226.00	-0.01	0.01	0.26	0.00	0.66	-	-
Frequency of picking berries or mushrooms during leisure time	Physical activity	21534.00	0.01	0.01	0.26	0.00	0.77	-	-
Changed everyday exercise during the last year	Physical activity	22791.00	0.00	0.01	0.79	0.74	0.05	-	-

Everyday exercise satisfaction	Physical activity	22730.00	0.02	0.01	0.05	0.00	0.77	-	-
Exercise during the last three months	Physical activity	22786.00	-0.06	0.01	0.00	0.00	0.40	0.18	4.00
If you exercise, change in exercise habits during the last year	Physical activity	19972.00	-0.01	0.01	0.40	0.61	0.11	-	-
Bregott on bread	Food	21079.00	-0.01	0.01	0.45	0.00	0.39	-	-
Whole grain crisp bread	Food	21079.00	0.01	0.01	0.31	0.00	0.90	-	-
Whole grain soft bread	Food	21079.00	0.00	0.01	0.75	0.00	0.59	-	-
White (soft) bread, thin crisp bread	Food	21079.00	0.00	0.01	0.96	0.00	0.67	-	-
Coffee rolls/buns, rusk	Food	21079.00	0.01	0.01	0.40	0.00	0.74	-	-
Cheese 28%	Food	21079.00	-0.02	0.01	0.03	0.00	0.41	-	-
Cheese 10-17%	Food	21079.00	0.01	0.01	0.56	0.52	0.15	-	-
Soft cheese	Food	9349.00	0.03	0.01	0.07	0.00	0.55	-	-
Soft whey cheese	Food	9349.00	-0.02	0.01	0.09	0.00	0.97	-	-
Sausage, liver pate on bread	Food	21079.00	0.01	0.01	0.20	0.70	0.07	-	-
Meat on bread	Food	21079.00	0.03	0.01	0.00	0.00	0.89	-	-
Butter on bread	Food	21079.00	-0.01	0.01	0.58	0.00	0.74	-	-
Oatflake, whole wheat, rye or barley porridge	Food	21079.00	-0.01	0.01	0.19	0.00	0.69	-	-
Rosehip, sweet syrup soup	Food	21079.00	0.01	0.01	0.32	0.00	0.64	-	-
Sour milk, yoghurt (3% fat)	Food	21079.00	-0.01	0.01	0.45	0.43	0.18	-	-
Sour milk, yoghurt (low fat)	Food	21079.00	0.01	0.01	0.16	0.00	0.70	-	-
Fiber cereals	Food	21079.00	-0.02	0.01	0.04	0.00	0.62	-	-
Corn flakes	Food	21079.00	0.00	0.01	0.85	0.14	0.28	-	-
Berries (fresh or frozen)	Food	21079.00	-0.01	0.01	0.13	0.00	0.57	-	-
Apple, pear, peach, orange, mandarin and grapefruit	Food	21079.00	-0.02	0.01	0.03	0.00	0.71	-	-
Ba	Food	21079.00	0.02	0.01	0.04	0.00	0.78	-	-
Root vegetables and carrot	Food	21079.00	-0.02	0.01	0.05	0.00	0.39	-	-
Low fat margarine on bread	Food	21079.00	0.02	0.01	0.06	0.00	0.35	-	-
Tomato and cucumber	Food	21079.00	0.00	0.01	0.69	0.00	0.92	-	-
White cabbage, lettuce, lettuce cabbage, spich, borecole and broccoli	Food	21079.00	-0.01	0.01	0.21	0.00	0.61	-	-
Mixed frozen vegetables	Food	9349.00	0.00	0.01	0.97	0.00	0.82	-	-
Boiled or baked potato	Food	21079.00	-0.01	0.01	0.48	0.00	0.70	-	-
Fried potatoes and pommes frites	Food	21079.00	0.02	0.01	0.12	0.00	0.38	-	-
Mashed potato	Food	9349.00	0.01	0.01	0.29	0.00	0.65	-	-
Potato salad	Food	9349.00	0.00	0.01	1.00	0.35	0.22	-	-
Rice	Food	21079.00	0.01	0.01	0.51	0.00	0.63	-	-
Pasta	Food	21079.00	-0.01	0.01	0.44	0.00	0.88	-	-
Brown beans and pea soup	Food	21079.00	0.01	0.01	0.33	0.00	0.47	-	-
Margarine on bread	Food	21079.00	0.01	0.01	0.45	0.00	0.88	-	-
Blota (broth + bread)	Food	9349.00	0.02	0.01	0.30	0.00	0.91	-	-
Pancake, waffle and Swedish dumpling	Food	21079.00	-0.03	0.01	0.00	0.00	0.49	-	-
Pizza	Food	21079.00	0.00	0.01	0.87	0.29	0.23	-	-
Minced meat dishes	Food	21079.00	-0.01	0.01	0.44	0.00	0.97	-	-
Meat stew	Food	21079.00	0.00	0.01	0.90	0.44	0.18	-	-
Steak, chop, etc.	Food	21079.00	-0.01	0.01	0.22	0.87	0.01	-	-

Bacon	Food	21079.00	0.01	0.01	0.24	0.00	0.68	-	-
Sausage as main dish	Food	21079.00	0.00	0.01	0.73	0.00	0.62	-	-
Hamburger	Food	21079.00	0.03	0.01	0.00	0.00	0.83	-	-
White meat (poultry)	Food	21079.00	-0.02	0.01	0.11	0.00	0.43	-	-
Butter for cooking	Food	21079.00	0.02	0.01	0.04	0.56	0.13	-	-
Blood based food	Food	9349.00	0.01	0.01	0.68	0.60	0.11	-	-
Liver and kidney	Food	9349.00	0.02	0.01	0.12	0.00	0.92	-	-
Lean fish (e.g. perch, bass, cod)	Food	21079.00	-0.03	0.01	0.01	0.00	0.44	-	-
Fatty fish (e.g. herring, whitefish, salmon)	Food	21079.00	0.00	0.01	0.67	0.30	0.23	-	-
Shellfish (e.g. shrimps, scallops)	Food	9349.00	0.01	0.01	0.61	0.00	0.52	-	-
Salty fish	Food	21079.00	0.00	0.01	0.76	0.00	0.86	-	-
Smoked fish/meat	Food	21079.00	0.00	0.01	0.71	0.00	0.77	-	-
Ice cream	Food	21079.00	0.00	0.01	0.67	0.00	0.87	-	-
Sweets (chocolate and candy)	Food	21079.00	0.01	0.01	0.15	0.19	0.27	-	-
Sugar, honey, marmelade and jam	Food	21079.00	0.00	0.01	0.70	0.00	0.80	-	-
Margarine for cooking	Food	21079.00	-0.01	0.01	0.52	0.68	0.08	-	-
Cookies and pastry	Food	21079.00	-0.02	0.01	0.02	0.00	0.97	-	-
Chips, popcorn and salted nuts	Food	21079.00	-0.01	0.01	0.37	0.47	0.17	-	-
Low fat milk (0.5%)	Beverage	21079.00	0.00	0.01	0.99	0.00	0.80	-	-
Milk, sour milk (1.5%)	Beverage	21079.00	0.01	0.01	0.17	0.00	0.53	-	-
Milk, sour milk (3%)	Beverage	21079.00	0.01	0.01	0.22	0.00	0.44	-	-
Sodas, soft drinks and juice	Beverage	21079.00	0.03	0.01	0.02	0.00	0.86	-	-
Brewed (filtered) coffee	Beverage	21079.00	-0.04	0.01	0.00	0.00	0.74	-	-
Boiled coffee	Beverage	21079.00	-0.02	0.01	0.13	0.00	0.93	-	-
Tea	Beverage	21079.00	0.01	0.01	0.24	0.00	0.90	-	-
Light beer	Alcohol	21079.00	-0.02	0.01	0.02	0.00	0.36	-	-
Oil for cooking	Food	21079.00	0.00	0.01	0.87	0.00	0.42	-	-
Medium beer	Alcohol	21079.00	0.01	0.01	0.45	0.00	0.91	-	-
Strong beer	Alcohol	21079.00	-0.02	0.01	0.11	0.00	0.68	-	-
Wine	Alcohol	21079.00	-0.01	0.01	0.38	0.00	0.49	-	-
Liquor and spirits	Alcohol	21079.00	0.01	0.01	0.41	0.00	0.58	-	-
Salad dressing with oil	Food	21079.00	-0.01	0.01	0.51	0.00	0.59	-	-
Cream, creme fraiche, sour cream	Food	21079.00	-0.01	0.01	0.32	0.64	0.09	-	-
Average portion size of vegetables based on photographic illustration of four sizes (smallest to largest)	Food	21079.00	-0.05	0.01	0.00	0.00	0.72	-	-
Overall state of health during the last year	General health	22767.00	-0.02	0.01	0.05	0.00	0.57	-	-
Overall state of health compared to others your age	General health	22453.00	-0.03	0.01	0.01	0.00	0.70	-	-
Parents or siblings had a cerebral hemorrhage/thrombosis or cardiac infarction before the age of 60	General health	22562.00	0.06	0.02	0.01	0.16	0.28	-	-
Teetotaler	Alcohol	22757.00	0.13	0.03	0.00	0.17	0.27	-	-
Feel the need to reduce alcohol consumption	Alcohol	20695.00	0.03	0.03	0.38	0.45	0.18	-	-
Feel uneasy or guilty because of your way of drinking	Alcohol	20688.00	0.01	0.03	0.66	0.00	0.67	-	-
Iron intake (mg/day)	Nutrients	21079.00	0.00	0.01	0.81	0.00	0.59	-	-
Iodine intake (ug/day)	Nutrients	21079.00	0.00	0.01	0.76	0.00	0.68	-	-

Calcium intake (mg/day)	Nutrients	21079.00	0.01	0.01	0.37	0.00	0.70	-	-
Potassium intake (mg/day)	Nutrients	21079.00	-0.01	0.01	0.57	0.00	0.83	-	-
Beta-carotene intake (mg/day)	Nutrients	21079.00	-0.02	0.01	0.05	0.00	0.49	-	-
Cholesterol intake (g/day)	Nutrients	21079.00	-0.02	0.01	0.07	0.00	0.44	-	-
Carbohydrates intake (g/day)	Nutrients	21079.00	0.01	0.01	0.40	0.00	0.55	-	-
Average portion size of meat/fish based on photographic illustration of four sizes (smallest to largest)	Food	21079.00	-0.05	0.01	0.00	0.00	0.94	0.18	8.00
Breakfast habits: Only coffee/tea for breakfast vs not breakfast at all	Food	1413.00	-0.40	0.13	0.00	0.00	0.69	-	-
Breakfast habits: Coffee/tea and wheat buns or rusk for breakfast vs not breakfast at all	Food	1755.00	-0.08	0.08	0.32	0.00	0.84	-	-
Breakfast habits: Porridge w/o sandwich for breakfast vs not breakfast at all	Food	3041.00	-0.01	0.06	0.92	0.00	0.98	-	-
Breakfast habits: Gruel w/o sandwich for breakfast vs not breakfast at all	Food	1658.00	0.01	0.10	0.94	0.00	0.87	-	-
Eat breakfast from 2000	Food	2147.00	-0.20	0.11	0.07	0.00	0.69	-	-
Enterodiol intake (ug/day)	Nutrients	21079.00	0.01	0.01	0.42	0.00	0.46	-	-
Enterolactone intake (ug/day)	Nutrients	21079.00	0.00	0.01	0.79	0.00	0.93	-	-
Equol intake (ug/day)	Nutrients	21079.00	0.01	0.01	0.46	0.00	0.98	-	-
Lariciresinol intake (ug/day)	Nutrients	21079.00	-0.02	0.01	0.06	0.00	0.56	-	-
Matairesinol intake (ug/day)	Nutrients	21079.00	0.00	0.01	0.79	0.00	0.75	-	-
Medioresinol intake (ug/day)	Nutrients	21079.00	0.01	0.01	0.38	0.00	0.56	-	-
Pinoresinol intake (ug/day)	Nutrients	21079.00	0.00	0.01	0.67	0.00	0.59	-	-
Secoisolariciresinol intake (ug/day)	Nutrients	21079.00	-0.05	0.01	0.00	0.00	0.86	0.19	1.00
Sum of all ligns intake (ug/day)	Nutrients	21079.00	0.00	0.01	0.93	0.00	0.58	-	-
Syringaresinol intake (ug/day)	Nutrients	21079.00	0.00	0.01	0.67	0.00	0.63	-	-
Sum of Lariciresinol, Matairesinol, Pinoresinol, Secoisolariciresinol intake (ug/day)	Nutrients	21079.00	-0.02	0.01	0.12	0.00	0.59	-	-
Satisfaction with home and family situation	Psychosocial	12520.00	0.02	0.01	0.10	0.00	0.55	-	-
Appetite status	Psychosocial	12516.00	-0.01	0.01	0.36	0.00	0.47	-	-
Mood status	Psychosocial	12512.00	0.03	0.01	0.02	0.00	0.40	-	-
Energy status	Psychosocial	12500.00	0.01	0.01	0.50	0.00	0.98	-	-
Patience status	Psychosocial	12511.00	0.03	0.01	0.07	0.00	0.48	-	-
Confidence status	Psychosocial	12509.00	0.00	0.01	0.80	0.00	0.84	-	-
Sleep status	Sleep	12533.00	0.02	0.01	0.08	0.50	0.16	-	-
Do you feel important and appreciated outside your home?	Psychosocial	12529.00	0.01	0.01	0.30	0.00	0.37	-	-

Do you feel important and appreciated in your home?	Psychosocial	12426.00	0.01	0.01	0.47	0.00	0.83	-	-
Satisfaction with accomodation	Psychosocial	12528.00	0.03	0.01	0.04	0.00	0.92	-	-
Satisfaction with work situation	Psychosocial	12400.00	0.01	0.01	0.32	0.48	0.16	-	-
Satisfaction with economy	Psychosocial	12514.00	0.02	0.01	0.13	0.00	0.48	-	-
Satisfaction with leisure time	Psychosocial	12504.00	0.02	0.01	0.11	0.90	0.00	-	-
Hearing status	General health	12527.00	0.00	0.01	0.79	0.53	0.14	-	-
Vision status	General health	12514.00	-0.02	0.01	0.25	0.00	0.89	-	-
Memory status	Psychosocial	12491.00	0.02	0.01	0.17	0.29	0.24	-	-
Fitness status	Physical activity	12504.00	-0.03	0.01	0.02	0.00	0.41	-	-
Magnesium intake (mg/day)	Nutrients	21079.00	-0.01	0.01	0.22	0.00	0.65	-	-
Saturated fat intake (g/day)	Nutrients	21079.00	0.00	0.01	0.96	0.00	0.72	-	-
Monounsaturated fat intake (g/day)	Nutrients	21079.00	0.00	0.01	0.79	0.00	0.65	-	-
Monosaccharides intake (g/day)	Nutrients	21079.00	0.00	0.01	0.82	0.00	0.96	-	-
Sodium intake (mg/day)	Nutrients	21079.00	0.01	0.01	0.49	0.00	0.88	-	-
Vitamin B3 intake (mg/day)	Nutrients	21079.00	-0.02	0.01	0.10	0.00	0.79	-	-
Cambridge physical activity index	Physical activity	22405.00	-0.05	0.01	0.00	0.00	0.38	0.18	6.00
Polyunsaturated fat intake (g/day)	Nutrients	21079.00	-0.01	0.01	0.44	0.00	0.73	-	-
Average portion size of potatoes/rice/pasta based on photographic illustration of four sizes (smallest to largest)	Food	21079.00	-0.07	0.01	0.00	0.00	0.49	0.18	7.00
Total protein intake (g/day)	Nutrients	21079.00	0.00	0.01	0.79	0.00	0.67	-	-
Animal based protein intake (g/day)	Nutrients	21079.00	0.00	0.01	0.74	0.00	0.75	-	-
Plant based protein intake (g/day)	Nutrients	21079.00	0.00	0.01	0.82	0.00	0.87	-	-
Vitamin A intake (mg/day)	Nutrients	21079.00	0.02	0.01	0.05	0.05	0.31	-	-
Sucrose intake (g/day)	Nutrients	21079.00	0.02	0.01	0.12	0.00	0.60	-	-
Cohabitation: Live alone vs Only one adult (spouse, partner)	Social	6851.00	-0.02	0.04	0.58	0.00	0.99	-	-
Cohabitation: Live alone vs Only children	Social	3082.00	-0.16	0.06	0.01	0.00	0.67	-	-
Cohabitation: Live alone vs Adult and children	Social	16331.00	-0.09	0.04	0.01	0.00	0.80	-	-
Cohabitation: Live alone vs Other/others	Social	2298.00	0.11	0.09	0.21	0.43	0.18	-	-
Selenium intake (ug/day)	Nutrients	21079.00	-0.01	0.01	0.47	0.00	0.97	-	-
Long-term sickness	General health	22006.00	0.06	0.03	0.05	0.00	0.56	-	-
Work shifts/weekends	Psychosocial	22098.00	-0.05	0.02	0.01	0.00	0.37	-	-
Number of cigarretes smoked per day (in groups)	Tobacco use	19116.00	0.01	0.01	0.24	0.00	0.38	-	-
Years smoking	Tobacco use	20133.00	-0.02	0.01	0.05	0.00	0.64	-	-
Grams of tobacco smoked per week	Tobacco use	14141.00	-0.09	0.02	0.00	0.00	0.36	-	-
Number of cigarretes smoked per day	Tobacco use	14141.00	-0.09	0.02	0.00	0.00	0.36	-	-
Number of cigars smoked per day	Tobacco use	14141.00	-0.09	0.02	0.00	0.00	0.36	-	-

Smoking status: Smokers vs non-smokers	Tobacco use	14978.00	-0.02	0.03	0.57	0.00	0.81	-	-
Smoking status: Former smokers vs non-smokers	Tobacco use	15737.00	-0.06	0.03	0.01	0.30	0.23	-	-
Smoking status: Former occasiol smokers vs non-smokers	Tobacco use	13182.00	-0.08	0.03	0.03	0.00	0.87	-	-
Number of snuff boxes per week	Tobacco use	21545.00	-0.02	0.01	0.08	0.00	0.43	-	-
Snuff status: Snuff users vs non-snuff users	Tobacco use	19189.00	-0.09	0.03	0.00	0.00	0.73	-	-
Snuff status: Former snuff users vs non-snuff users	Tobacco use	17584.00	-0.09	0.03	0.01	0.51	0.15	-	-
Years using snuff	Tobacco use	20949.00	-0.04	0.01	0.00	0.00	0.33	-	-
Participation in associations or voluntary organisations	Social	22715.00	-0.04	0.02	0.04	0.05	0.31	-	-
Participation in sports or physical exercise associations	Social	1367.00	-0.04	0.08	0.61	0.00	0.51	-	-
Participation in study circles	Social	1367.00	0.13	0.12	0.26	0.50	0.16	-	-
Participation in other association	Social	1367.00	-0.10	0.08	0.21	0.62	0.10	-	-
People to ask for help apart from the ones at home	Social	22706.00	-0.02	0.03	0.63	0.00	0.71	-	-
Number of friends that can come to your home at any time and feel at home	Social	22703.00	0.01	0.01	0.53	0.00	0.62	-	-
Number of social contacts with the same interests as you	Social	22704.00	0.00	0.01	0.74	0.00	0.38	-	-
Would you say that the number of people that you meet in your everyday life is enough or would you like to meet more or fewer people?	Social	22731.00	-0.02	0.01	0.02	0.00	0.75	-	-
Close relationship with anyone	Social	22761.00	-0.01	0.01	0.15	0.00	0.76	-	-
Frequency of engaging in clubs, associations or study circles	Social	15241.00	0.00	0.01	0.70	0.00	0.34	-	-
Number of social interactions during a normal week	Social	22753.00	0.02	0.01	0.06	0.00	0.68	-	-
Support from others	Social	22750.00	-0.01	0.01	0.13	0.83	0.02	-	-
Number of people with whom you can speak openly	Social	22739.00	0.00	0.01	0.61	0.00	0.50	-	-
Receive hugs to comfort and support you	Social	22642.00	-0.03	0.03	0.21	0.00	0.85	-	-
Stigmasterol intake (mg/day)	Nutrients	21079.00	0.00	0.01	0.96	0.00	0.36	-	-
Tiamin intake (mg/day)	Nutrients	21079.00	0.00	0.01	0.77	0.00	0.47	-	-
Vitamin E intake (mg/day)	Nutrients	21079.00	-0.01	0.01	0.54	0.00	0.35	-	-
Trans fat intake (g/day)	Nutrients	21079.00	-0.01	0.01	0.45	0.00	0.51	-	-

Sum of phytosterols intake (mg/day)	Nutrients	21079.00	-0.01	0.01	0.37	0.13	0.28	-	-
Educatiol level	Psychosocial	22781.00	-0.04	0.01	0.00	0.00	0.87	0.18	5.00
Zinc intake (mg/day)	Nutrients	21079.00	0.00	0.01	0.86	0.00	0.82	-	-

Supplementary Table 21. Summary of significant top 5 lifestyle variables regarding variance explained for all the cardiometabolic traits in linear mixed model analyses

Description	Group	BMI	HDL-C	LDL-C	Tot Chol	Trigl	DBP	SBP	F Glu	2h Glu	N of tentative associations
Alcohol intake (g/day)	Alcohol	-	+		+				+	-	5
Permanent employment	Psychosocial	-			+						2
Job demands to work very fast	Psychosocial	-									1
Frequency of social contacts with colleagues during leisure time	Psychosocial						+				1
Ingenuity or creativity demand from job	Psychosocial									-	1
Possibility to speak with colleagues during breaks	Psychosocial	-									1
Vitamin C intake (mg/day)	Nutrients					-					1
Informed of having high blood pressure	General health	+	-			+	+	+	+	+	7
Beta-sitostanol intake (mg/day)	Nutrients							+			1
Marital status: Single vs Married/partner	Social						-	-			2
Marital status: Single vs Divorced/separated	Social	-						-			2
Parents or siblings have diabetes	General health		-						+	+	3
Disaccharides intake (g/day)	Nutrients		-								1
Total energy intake (kcal/day)	Nutrients						-				1
Formic acid intaje (g/day)	Nutrients			+							1
Palmitic acid intake (g/day)	Nutrients			+	+						2
Arachidonic acid (ARA) intake (g/day)	Nutrients	+									1
Fibre intake (g/day)	Nutrients	-				-			-		3
Folic acid intake (ug/day)	Nutrients					-					1
Whole grain intake (g/day)	Food							+	-		2
Travel to work: Cycle to work vs passive travel to work	Physical activity	-	+			-					3
Frequency of walking during leisure time	Physical activity						+				1
Frequency of cycling during leisure time	Physical activity		+			-					2
Frequency of shoveling snow during leisure time	Physical activity									-	1
Frequency of hunting or fishing during leisure time	Physical activity				+		+	+	-	-	5
Frequency of picking berries or mushrooms during leisure time	Physical activity				+				-	-	3
Everyday exercise satisfaction	Physical activity	-									1
Exercise during the last three months	Physical activity	-	+	-	-	-	-		-	-	8
If you exercise, change in exercise habits during the last year	Physical activity				-						1
Bregott on bread	Food				+						1
Whole grain crisp bread	Food						+	+			2
Whole grain soft bread	Food								-		1
Coffee rolls/buns, rusk	Food		-								1
Oatflake, whole wheat, rye or barley porridge	Food								-		1
Rosehip, sweet syrup soup	Food									+	1
Fiber cereals	Food				-	-			-		3
Apple, pear, peach, orange, mandarin and grapefruit	Food					-			-	-	3
Ba	Food			-	-						2
Low fat margarine on bread	Food							+			1
Fried potatoes and pommes frites	Food					+					1
Pizza	Food					+					1
Bacon	Food		+		+						2
Sausage as main dish	Food	+									1
White meat (poultry)	Food							-			1
Butter for cooking	Food		+								1
Sugar, honey, marmelade and jam	Food		-								1
Margarine for cooking	Food				+	+					2
Cookies and pastry	Food		-								1
Low fat milk (0.5%)	Beverage	+		-	-						3
Milk, sour milk (3%)	Beverage	-			+						2
Brewed (filtered) coffee	Beverage	-				-	-	-		-	5
Boiled coffee	Beverage	+		+	+	+				-	5
Tea	Beverage	-			-					+	3
Medium beer	Alcohol	-	+		+						3
Strong beer	Alcohol		+		+	+				-	4
Wine	Alcohol	-	+		+				+		4
Liquor and spirits	Alcohol	+	+		+						3
Cream, creme fraiche, sour cream	Food			+							1

Average portion size of vegetables based on photographic illustration of four sizes (smallest to largest)	Food	-					-			-	3
Overall state of health during the last year	General health	-	+			-	-	-	-	-	7
Overall state of health compared to others your age	General health	-			-			-		-	4
Parents or siblings had a cerebral hemorrhage/thrombosis or cardiac infarction before the age of 60	General health				+		+	+			3
Teetotaler	Alcohol								-	+	2
Feel the need to reduce alcohol consumption	Alcohol					+	+				2
Iodine intake (ug/day)	Nutrients						-				1
Cholesterol intake (g/day)	Nutrients		+					-			2
Carbohydrates intake (g/day)	Nutrients		-		-						2
Average portion size of meat/fish based on photographic illustration of four sizes (smallest to largest)	Food	+		+				-		-	4
Breakfast habits: Gruel w/o sandwich for breakfast vs not breakfast at all	Food	-									1
Eat breakfast from 2000	Food									+	1
Lariciresinol intake (ug/day)	Nutrients								-	-	2
Medioresinol intake (ug/day)	Nutrients							+			1
Pinoresinol intake (ug/day)	Nutrients								-		1
Secoisolariciresinol intake (ug/day)	Nutrients		+			-				-	3
Syringaresinol intake (ug/day)	Nutrients							+			1
Sum of Lariciresinol, Matairesinol, Pinoresinol, Secoisolariciresinol intake (ug/day)	Nutrients								-	-	2
Satisfaction with home and family situation	Psychosocial							+			1
Appetite status	Psychosocial					-			-		2
Mood status	Psychosocial					-					1
Energy status	Psychosocial	-	+			-				-	4
Sleep status	Sleep	-				-					2
Satisfaction with work situation	Psychosocial					-					1
Satisfaction with economy	Psychosocial					-	+	+			3
Satisfaction with leisure time	Psychosocial							+			1
Vision status	General health					-					1
Memory status	Psychosocial							+			1
Fitness status	Physical activity	-	+			-	-	-	-		6
Magnesium intake (mg/day)	Nutrients									-	1
Monounsaturated fat intake (g/day)	Nutrients			+	+	+					3
Monosaccharides intake (g/day)	Nutrients									-	1
Sodium intake (mg/day)	Nutrients	+									1
Vitamin B3 intake (mg/day)	Nutrients	+									1
Cambridge physical activity index	Physical activity	-	+		-	-			-	-	6
Average portion size of potatoes/rice/pasta based on photographic illustration of four sizes (smallest to largest)	Food	+					-				2
Animal based protein intake (g/day)	Nutrients	+									1
Plant based protein intake (g/day)	Nutrients			-	-				-		3
Sucrose intake (g/day)	Nutrients		-								1
Cohabitation: Live alone vs Only children	Social				-		-	-			3
Cohabitation: Live alone vs Adult and children	Social					-	-	-			3
Self rate of overall health	General health				-	-	-	-			4
Excellent health	General health				-		-				2
Physical limitation to participate in steneous activities: running, lifting heavy objects, taking part in physically demanding sports	General health				+						1

Physical limitation to participate in moderately demanding activities: walking up several stairs	General health	+									1
Physical limitation to participate in moderately demanding activities: bending down or kneeling	General health	+									1
For how much of the time during the last four weeks have you felt tired?	General health		-								1
Long-term sickness	General health					+					1
Work shifts/weekends	Psychosocial	+									1
Number of cigarretes smoked per day (in groups)	Tobacco use		-		+	+	-		+	-	6
Years smoking	Tobacco use		-		+	+	-	-	+	-	7
Number of cigarretes smoked per day	Tobacco use	-					-	-			3
Smoking status: Smokers vs non-smokers	Tobacco use		-		+	+	-		+	-	6
Smoking status: Former smokers vs non-smokers	Tobacco use	+				+	-	-	+	-	6
Smoking status: Former occasiol smokers vs non-smokers	Tobacco use							-			1
Number of snuff boxes per week	Tobacco use							-			1
Snuff status: Snuff users vs non-snuff users	Tobacco use		+			+				-	3
Snuff status: Former snuff users vs non-snuff users	Tobacco use				+						1
Years using snuff	Tobacco use	+	+								2
Participation in associations or voluntary organisations	Social					-	-		-		3
Participation in sports or physical exercise associations	Social	-								-	2
Participation in study circles	Social	+									1
Participation in other association	Social	+									1
Number of friends that can come to your home at any time and feel at home	Social				+						1
Number of social contacts with the same interests as you	Social				+						1
Receive hugs to comfort and support you	Social					-					1
Trans fat intake (g/day)	Nutrients			+	+						2

BMI: Body Mass Index; SBP: Systolic blood pressure; DBP: Diastolic blood pressure; Tot Chol: Total cholesterol; Trigl: Triglycerides; HDL-C: HDL cholesterol; LDL-C: LDL cholesterol; F Glu: Fasting glucose; 2h Glu: 2h glucose

Supplementary Table 22. Number of tentative signals ranked among the top 5 regarding variance explained in linear mixed model ananalyses that are shared between the different cardiometabolic traits

	BMI	SBP	DBP	Tot Chol	Trigl	HDL-C	LDL-C	F Glu	2h Glu
**BMI**	45								
**SBP**	10	35							
**DBP**	13	18	34						
**Tot Chol**	10	9	10	36					
**Trigl**	13	10	16	11	38				
**HDL-C**	15	8	11	10	14	32			
**LDL-C**	5	2	2	9	3	3	13		
**F Glu**	11	9	11	7	14	10	2	26	
**2h Glu**	16	10	13	10	17	13	4	16	37

Supplementary Table 23. Summary of significant top 5 lifestyle variables regarding variance explained for all the cardiometabolic traits in longitudinal analyses

Description	Group	BMI	Tot Chol	Trigl	DBP	SBP	F glu	2h glu	Score
rachidonic acid (ARA) intake (g/day	Nutrients	+							1
Enterodiol intake (ug/day)	Nutrients	+							1
Equol intake (ug/day)	Nutrients	+							1
Matairesinol intake (ug/day)	Nutrients	-							1
Secoisolariciresinol intake (ug/day)	Nutrients							-	1
Monounsaturated fat intake (g/day)	Nutrients		+						1
Tiamin intake (mg/day)	Nutrients	+							1
Alcohol intake (g/day)	Alcohol		+						1
Permanent employment	Psychosocial		+						1
High physical demand from job	Physical activity	+							1
Repetitive job	Psychosocial	+							1
formed of having high blood pressu	General health	+			+	+		+	4
arital status: Single vs Married/partn	Social	-							1
Parents or siblings have diabetes	General health						+	+	2
work: Cycle to work vs passive trav	Physical activity	-							1
picking berries or mushrooms durin	Physical activity		-						1
ged everyday exercise during the las	Physical activity	+							1
Exercise during the last three months	Physical activity							-	1
ise, change in exercise habits during	Physical activity	+							1
lake, whole wheat, rye or barley porr	Food				-				1
Sour milk, yoghurt (3% fat)	Food	-							1
Fiber cereals	Food	-		-					2
Tomato and cucumber	Food	+							1
Fried potatoes and pommes frites	Food	+							1
Chips, popcorn and salted nuts	Food		+						1
Brewed (filtered) coffee	Beverage		+						1
Strong beer	Alcohol		+						1
verall state of health during the last y	General health	-		-					2
al hemorrhage/thrombosis or cardiac	General health				+	+			2
Teetotaler	Alcohol		-						1
h based on photographic illustration	Food							-	1
: Only coffee/tea for breakfast vs no	Food		+						1
isfaction with home and family situat	Psychosocial	-							1
Sleep status	Sleep	-							1
Satisfaction with accomodation	Psychosocial	-							1
Satisfaction with economy	Psychosocial	-							1
Fitness status	Physical activity	-							1
Cambridge physical activity index	Physical activity							-	1
/pasta based on photographic illustra	Food							-	1
: Live alone vs Only one adult (spou	Social	-							1
bitation: Live alone vs Adult and chi	Social	-							1
Work shifts/weekends	Psychosocial	+							1
er of cigarretes smoked per day (in g	Tobacco use	+		+			+		3
Years smoking	Tobacco use						+		1
Grams of tobacco smoked per week	Tobacco use		+	+					2
Number of cigarretes smoked per day	Tobacco use	+	+	+					3
Number of cigars smoked per day	Tobacco use		+	+					2
oking status: Smokers vs non-smok	Tobacco use	+		+			+		3
Number of snuff boxes per week	Tobacco use		+	+					2
ff status: Snuff users vs non-snuff u	Tobacco use	+	+	+					3
Years using snuff	Tobacco use	+	+						2
Close relationship with anyone	Social	-							1
Educatiol level	Psychosocial					-	-	-	3

BMI: Body Mass Index; SBP: Systolic blood pressure; DBP: Diastolic blood pressure; Tot Chol: Total cholesterol; Trigl: Triglycerides; F Glu: Fasting glucose; 2h Glu: 2h glucose

Supplementary Table 24. Number of tentative signals ranked among the top 5 regarding variance explained in longitudinal analyses that are shared between the different cardiometabolic traits

	BMI	SBP	DBP	Tot Chol	Trigl	HDL-C	LDL-C	F Glu	2h Glu
**BMI**	31								
**SBP**	1	3							
**DBP**	1	2	3						
**Tot Chol**	3	0	0	15					
**Trigl**	6	0	0	5	9				
**HDL-C**	0	0	0	0	0	0			
**LDL-C**	0	0	0	0	0	0	0		
**F Glu**	2	1	0	0	2	0	0	5	
**2h Glu**	1	2	1	0	0	0	0	2	8

Supplementary Table 25. Reference for the numerical labels in the figures

Number	Description
**1**	Fitness status
**2**	Physical limitation to participate in moderately demanding activities: bending down or kneeling
**3**	Physical limitation to participate in moderately demanding activities: walking up several stairs
**4**	Physical limitation to participate in steneous activities: running, lifting heavy objects, taking part in physically demanding sports
**5**	Self rate of overall health
**6**	Everyday exercise satisfaction
**7**	Physical limitation to participate in moderately demanding activities: walking more than two km
**8**	Overall state of health during the last year
**9**	Informed of having high blood pressure
**10**	Excellent health
**11**	Exercise during the last three months
**12**	Energy status
**13**	Overall state of health compared to others your age
**14**	Physical limitation to participate in moderately demanding activities: moving a table, vacuuming, walking in the forest or gardening
**15**	Cambridge physical activity index
**16**	Pain during the last four weeks
**17**	Average portion size of meat/fish based on photographic illustration of four sizes (smallest to largest)
**18**	As healthy as anyone
**19**	How much has the pain during the last four weeks disturbed your normal work?
**20**	For how much of the time during the last four weeks have you felt really alert and strong?
**21**	Travel to work: Cycle to work vs passive travel to work
**22**	Physical limitation that made you do less than you wanted during the last four weeks
**23**	Frequency of walking during leisure time
**24**	Average portion size of potatoes/rice/pasta based on photographic illustration of four sizes (smallest to largest)
**25**	Animal based protein intake (g/day)
**26**	For how much of the time during the last four weeks have you felt worn out?
**27**	For how much of the time during the last four weeks have you felt full of energy?
**28**	Fibre intake (g/day)
**29**	Secoisolariciresinol intake (ug/day)
**30**	Arachidonic acid (ARA) intake (g/day)
**31**	For how much of the time during the last four weeks have you felt tired?
**32**	Sodium intake (mg/day)
**33**	Frequency of cycling during leisure time
**34**	Physical limitation that made you not being able to perform certain work tasks or other activities during the last four weeks
**35**	Get sick more often than other people
**36**	Sum of Lariciresinol, Matairesinol, Pinoresinol, Secoisolariciresinol intake (ug/day)
**37**	Vitamin B3 intake (mg/day)
**38**	Sausage as main dish
**39**	Vitamin D intake (ug/day)
**40**	Physical limitation that limited your ability to perform certain work tasks or other activities during the last four weeks
**41**	Parents or siblings have diabetes
**42**	Long-term sickness
**43**	Pinoresinol intake (ug/day)
**44**	Lariciresinol intake (ug/day)
**45**	Monounsaturated fat intake (g/day)
**46**	Participation in sports or physical exercise associations
**47**	Total protein intake (g/day)
**48**	Plant based protein intake (g/day)
**49**	Changed everyday exercise during the last year
**50**	Worsen in health in the future
**51**	Folic acid intake (ug/day)
**52**	Steak, chop, etc.
**53**	Meat stew
**54**	Physical limitation to participate in moderately demanding activities: lifting or carrying grocery bags
**55**	If you exercise, change in exercise habits during the last year
**56**	Whole grain intake (g/day)
**57**	Cholesterol intake (g/day)
**58**	Monosaccharides intake (g/day)
**59**	Bacon
**60**	Carbohydrates intake (g/day)
**61**	Matairesinol intake (ug/day)
**62**	Extent to what your physical and emotiol health disrupted your usual social life during the last four weeks
**63**	Minced meat dishes
**64**	Wine
**65**	For how much of the time during the last four weeks has your physical health or your emotiol problems limited your ability to interact with others?
**66**	Vitamin B12 intake (ug/day)
**67**	Breakfast habits: Porridge w/o sandwich for breakfast vs not breakfast at all
**68**	Emotiol problems that made you do less than you wanted during the last four weeks
**69**	Permanent employment
**70**	Stigmasterol intake (mg/day)
**71**	Satisfaction with leisure time
**72**	Physical limitation that reduced the normal time spent at work or in other activities during the last four weeks
**73**	Sum of all ligns intake (ug/day)
**74**	Breakfast habits: Gruel w/o sandwich for breakfast vs not breakfast at all
**75**	Hamburger
**76**	Oatflake, whole wheat, rye or barley porridge
**77**	Vitamin C intake (mg/day)
**78**	Tea
**79**	Fat intake (g/day)
**80**	Selenium intake (ug/day)
**81**	Frequency of dancing during leisure time
**82**	Participation in other association

**83**	Fiber cereals
**84**	Beta-sitosterol intake (mg/day)
**85**	Frequency of picking berries or mushrooms during leisure time
**86**	White cabbage, lettuce, lettuce cabbage, spich, borecole and broccoli
**87**	Root vegetables and carrot
**88**	Smoking status: Former smokers vs non-smokers
**89**	Beta-carotene intake (mg/day)
**90**	Salad dressing with oil
**91**	Palmitic acid intake (g/day)
**92**	Distance to work in kilometers (one way)
**93**	White meat (poultry)
**94**	Magnesium intake (mg/day)
**95**	Low fat milk (0.5%)
**96**	Tomato and cucumber
**97**	Pancake, waffle and Swedish dumpling
**98**	For how much of the time during the last four weeks have you felt gloomy and sad?
**99**	Salty fish
**100**	Eat breakfast from 2000
**101**	Fried potatoes and pommes frites
**102**	Sum of phytosterols intake (mg/day)
**103**	Syringaresinol intake (ug/day)
**104**	Self rate of overall health compared to a year ago
**105**	Sour milk, yoghurt (3% fat)
**106**	Possibility to speak with colleagues during breaks
**107**	Sucrose intake (g/day)
**108**	Alcohol intake (g/day)
**109**	Frequency of hunting or fishing during leisure time
**110**	Margarine for cooking
**111**	For how much of the time during the last four weeks have you felt happy?
**112**	Vitamin B6 intake (mg/day)
**113**	Sausage, liver pate on bread
**114**	Travel to work: Walk to work vs passive travel to work
**115**	Apple, pear, peach, orange, mandarin and grapefruit
**116**	Eicosapentaenoic acid (EPA) intake (g/day)
**117**	Years using snuff
**118**	Average portion size of vegetables based on photographic illustration of four sizes (smallest to largest)
**119**	Docosahexaenoic acid (DHA) intake (g/day)
**120**	Pizza
**121**	Boiled coffee
**122**	Light and physically active work
**123**	For how much of the time during the last four weeks have you felt so depressed that nothing could cheer you up?
**124**	Marital status: Single vs Divorced/separated
**125**	Berries (fresh or frozen)
**126**	Beta-sitostanol intake (mg/day)
**127**	Boiled or baked potato
**128**	Number of cigarretes smoked per day
**129**	Breakfast habits: Coffee/tea and wheat buns or rusk for breakfast vs not breakfast at all
**130**	Sedentary or standing work
**131**	Campestanol intake (mg/day)
**132**	Brewed (filtered) coffee
**133**	Job demands to work very fast
**134**	Medioresinol intake (ug/day)
**135**	Participation in study circles
**136**	Equol intake (ug/day)
**137**	Total energy intake (kcal/day)
**138**	Ba
**139**	Brown beans and pea soup
**140**	Patience status
**141**	Vitamin A intake (mg/day)
**142**	Work shifts/weekends
**143**	Liquor and spirits
**144**	Medium beer
**145**	Satisfaction with economy
**146**	Sugar, honey, marmelade and jam
**147**	For how much of the time during the last four weeks have you felt calm and serene?
**148**	Parents or siblings had a cerebral hemorrhage/thrombosis or cardiac infarction before the age of 60
**149**	Pentadecanoic acid intake (g/day)
**150**	Heptadecanoic acid intake (g/day)
**151**	Low fat margarine on bread
**152**	Zinc intake (mg/day)
**153**	Travel to work: Irregular travel mode to work vs passive travel to work
**154**	Pasta
**155**	Years smoking
**156**	Whole grain crisp bread
**157**	Mood status
**158**	Sleep status
**159**	Snuff status: Former snuff users vs non-snuff users
**160**	Milk, sour milk (3%)
**161**	Enterodiol intake (ug/day)
**162**	Polyunsaturated fat intake (g/day)
**163**	Saturated fat intake (g/day)
**164**	Receive hugs to comfort and support you
**165**	Frequent social contacts with colleagues during work
**166**	Hearing status

**167**	Whole grain soft bread
**168**	Frequency of gardening during leisure time
**169**	Milk, sour milk (1.5%)
**170**	Control over own work assignment
**171**	Number of cigarretes smoked per day (in groups)
**172**	Vitamin B2 intake (ug/day)
**173**	Satisfaction with work situation
**174**	Appetite status
**175**	Would you say that the number of people that you meet in your everyday life is enough or would you like to meet more or fewer people?
**176**	Coffee rolls/buns, rusk
**177**	Phosphate intake (mg/day)
**178**	Number of friends that can come to your home at any time and feel at home
**179**	Iodine intake (ug/day)
**180**	Cream, creme fraiche, sour cream
**181**	Disaccharides intake (g/day)
**182**	Teetotaler
**183**	Lean fish (e.g. perch, bass, cod)
**184**	Breakfast habits: Only coffee/tea for breakfast vs not breakfast at all
**185**	Light beer
**186**	Support from others
**187**	Light but partly physically active work
**188**	Repetitive job
**189**	Smoked fish/meat
**190**	Fatty fish (e.g. herring, whitefish, salmon)
**191**	Grams of tobacco smoked per week
**192**	Snuff status: Snuff users vs non-snuff users
**193**	Smoking status: Smokers vs non-smokers
**194**	Rosehip, sweet syrup soup
**195**	Do you feel important and appreciated in your home?
**196**	Control over planning and execution of the workday
**197**	Number of social contacts with the same interests as you
**198**	For how much of the time during the last four weeks have you felt very nervous?
**199**	Cohabitation: Live alone vs Only children
**200**	Sometimes physically straining work
**201**	Cheese 28%
**202**	White (soft) bread, thin crisp bread
**203**	Skill demand from job
**204**	Cookies and pastry
**205**	Smoking status: Former occasiol smokers vs non-smokers
**206**	Sodas, soft drinks and juice
**207**	Ice cream
**208**	Enough time for job assignments
**209**	Participation in associations or voluntary organisations
**210**	Number of social interactions during a normal week
**211**	Calcium intake (mg/day)
**212**	Linolenic acid intake (g/day)
**213**	High mental demand from job
**214**	Confidence status
**215**	Trans fat intake (g/day)
**216**	Sweets
**217**	Frequency of social contacts with colleagues during leisure time
**218**	Contradictory demands in job
**219**	Satisfaction with accomodation
**220**	Do you feel important and appreciated outside your home?
**221**	Bregott on bread
**222**	Vitamin E intake (mg/day)
**223**	Frequency of shoveling snow during leisure time
**224**	Feel uneasy or guilty because of your way of drinking
**225**	Marital status: Single vs Widow/widower
**226**	Iron intake (mg/day)
**227**	Last time a colleague visited you at home
**228**	Possibility to leave your work for a while to speak with a colleague
**229**	Butter on bread
**230**	Marital status: Single vs Married/partner
**231**	Oil for cooking
**232**	Strong beer
**233**	Cohabitation: Live alone vs Other/others
**234**	Memory status
**235**	Formic acid intaje (g/day)
**236**	Meat on bread
**237**	Corn flakes
**238**	Sour milk, yoghurt (low fat)
**239**	Number of people with whom you can speak openly
**240**	Satisfaction with home and family situation
**241**	Campesterol intake (mg/day)
**242**	Potato salad
**243**	Learn new things at job
**244**	Feel the need to reduce alcohol consumption
**245**	Cheese 10-17%
**246**	High physical demand from job
**247**	Enterolactone intake (ug/day)
**248**	Butter for cooking
**249**	People to ask for help apart from the ones at home
**250**	Rice

**251**	Self-employed
**252**	Ingenuity or creativity demand from job
**253**	Linoleic acid intake (g/day)
**254**	Tiamin intake (mg/day)
**255**	Number of snuff boxes per week
**256**	Number of cigars smoked per day
**257**	Close relationship with anyone
**258**	Cohabitation: Live alone vs Adult and children
**259**	Frequency of engaging in clubs, associations or study circles
**260**	Chips, popcorn and salted nuts
**261**	Potassium intake (mg/day)
**262**	Margarine on bread
**263**	Cohabitation: Live alone vs Only one adult (spouse, partner)
**264**	Vision status
**265**	Time spent in a week in moderately steneous activities
**266**	Amount of exercise during the last 12 months
**267**	Soft cheese
**268**	Soft whey cheese
**269**	Mixed frozen vegetables
**270**	Mashed potato
**271**	Blota (broth + bread)
**272**	Blood based food
**273**	Liver and kidney
**274**	Shellfish (e.g. shrimps, scallops)
**275**	Frequency of alcohol consumption
**276**	Amount of alcohol drunk in a day
**277**	Frequency of drinking six or more glasses at the same occasion
**278**	Times during last year that you felt guilty because of your drinking
**279**	Risk of sleeping while sitting and reading
**280**	Risk of sleeping while watching TV
**281**	Risk of sleeping while sitting ictive in a public place
**282**	Risk of sleeping as a passenger in a car for one hour without break
**283**	Risk of sleeping while lying down resting in the afternoon
**284**	Risk of sleeping while sitting still after having lunch
**285**	Snore during sleep
**286**	Breath-holds during sleep

Supplementary Table 26. Modifiable (generic and specific) and non-modifiable categories

Category	Description
Modifiable (generic)	Fitness status
Modifiable (generic)	Physical limitation to participate in moderately demanding activities: bending down or kneeling
Modifiable (generic)	Physical limitation to participate in moderately demanding activities: walking up several stairs
Modifiable (generic)	Physical limitation to participate in steneous activities: running, lifting heavy objects, taking part in physically demanding sports
Modifiable (generic)	Self rate of overall health
Modifiable (generic)	Everyday exercise satisfaction
Modifiable (generic)	Physical limitation to participate in moderately demanding activities: walking more than two km
Modifiable (generic)	Overall state of health during the last year
Non-modifiable	Informed of having high blood pressure
Modifiable (generic)	Excellent health
Modifiable (generic)	Exercise during the last three months
Modifiable (generic)	Energy status
Modifiable (generic)	Overall state of health compared to others your age
Modifiable (generic)	Physical limitation to participate in moderately demanding activities: moving a table, vacuuming, walking in the forest or gardening
Modifiable (generic)	Cambridge physical activity index
Modifiable (generic)	Pain during the last four weeks
Modifiable (generic)	Average portion size of meat/fish based on photographic illustration of four sizes (smallest to largest)
Modifiable (generic)	As healthy as anyone
Modifiable (generic)	How much has the pain during the last four weeks disturbed your normal work?
Modifiable (generic)	For how much of the time during the last four weeks have you felt really alert and strong?
Modifiable (generic)	Travel to work: Cycle to work vs passive travel to work
Modifiable (generic)	Physical limitation that made you do less than you wanted during the last four weeks

Modifiable (generic)	Frequency of walking during leisure time
Modifiable (generic)	Average portion size of potatoes/rice/pasta based on photographic illustration of four sizes (smallest to largest)
Modifiable (generic)	Animal based protein intake (g/day)
Modifiable (generic)	For how much of the time during the last four weeks have you felt worn out?
Modifiable (generic)	For how much of the time during the last four weeks have you felt full of energy?
Modifiable (generic)	Fibre intake (g/day)
Modifiable (generic)	Secoisolariciresinol intake (ug/day)
Modifiable (generic)	Arachidonic acid (ARA) intake (g/day)
Modifiable (generic)	For how much of the time during the last four weeks have you felt tired?
Modifiable (generic)	Sodium intake (mg/day)
Modifiable (generic)	Frequency of cycling during leisure time
Modifiable (generic)	Physical limitation that made you not being able to perform certain work tasks or other activities during the last four weeks
Modifiable (generic)	Get sick more often than other people
Modifiable (generic)	Sum of Lariciresinol, Matairesinol, Pinoresinol, Secoisolariciresinol intake (ug/day)
Modifiable (generic)	Vitamin B3 intake (mg/day)
Modifiable (generic)	Sausage as main dish
Modifiable (generic)	Vitamin D intake (ug/day)
Modifiable (generic)	Physical limitation that limited your ability to perform certain work tasks or other activities during the last four weeks
Non-modifiable	Parents or siblings have diabetes
Modifiable (generic)	Long-term sickness
Modifiable (generic)	Pinoresinol intake (ug/day)
Modifiable (generic)	Lariciresinol intake (ug/day)
Modifiable (generic)	Monounsaturated fat intake (g/day)

Modifiable (generic)	Participation in sports or physical exercise associations
Modifiable (generic)	Total protein intake (g/day)
Modifiable (generic)	Plant based protein intake (g/day)
Modifiable (generic)	Changed everyday exercise during the last year
Modifiable (generic)	Worsen in health in the future
Modifiable (generic)	Folic acid intake (ug/day)
Modifiable (generic)	Steak, chop, etc.
Modifiable (generic)	Meat stew
Modifiable (generic)	Physical limitation to participate in moderately demanding activities: lifting or carrying grocery bags
Modifiable (generic)	If you exercise, change in exercise habits during the last year
Modifiable (generic)	Whole grain intake (g/day)
Modifiable (generic)	Cholesterol intake (g/day)
Modifiable (generic)	Monosaccharides intake (g/day)
Modifiable (generic)	Bacon
Modifiable (generic)	Carbohydrates intake (g/day)
Modifiable (generic)	Matairesinol intake (ug/day)
Modifiable (generic)	Extent to what your physical and emotiol health disrupted your usual social life during the last four weeks
Modifiable (generic)	Minced meat dishes
Modifiable (generic)	Wine
Modifiable (generic)	For how much of the time during the last four weeks has your physical health or your emotiol problems limited your ability to interact with others?
Modifiable (generic)	Vitamin B12 intake (ug/day)
Modifiable (generic)	Breakfast habits: Porridge w/o sandwich for breakfast vs not breakfast at all

Modifiable (generic)	Emotiol problems that made you do less than you wanted during the last four weeks
Modifiable (generic)	Permanent employment
Modifiable (generic)	Stigmasterol intake (mg/day)
Modifiable (generic)	Satisfaction with leisure time
Modifiable (generic)	Physical limitation that reduced the normal time spent at work or in other activities during the last four weeks
Modifiable (generic)	Sum of all ligns intake (ug/day)
Modifiable (generic)	Breakfast habits: Gruel w/o sandwich for breakfast vs not breakfast at all
Modifiable (generic)	Hamburger
Modifiable (generic)	Oatflake, whole wheat, rye or barley porridge
Modifiable (generic)	Vitamin C intake (mg/day)
Modifiable (generic)	Tea
Modifiable (generic)	Fat intake (g/day)
Modifiable (generic)	Selenium intake (ug/day)
Modifiable (generic)	Frequency of dancing during leisure time
Modifiable (generic)	Participation in other association
Modifiable (generic)	Fiber cereals
Modifiable (generic)	Beta-sitosterol intake (mg/day)
Modifiable (generic)	Frequency of picking berries or mushrooms during leisure time
Modifiable (generic)	White cabbage, lettuce, lettuce cabbage, spich, borecole and broccoli
Modifiable (generic)	Root vegetables and carrot
Modifiable (generic)	Smoking status: Former smokers vs non-smokers
Modifiable (generic)	Beta-carotene intake (mg/day)

Modifiable (generic)	Salad dressing with oil
Modifiable (generic)	Palmitic acid intake (g/day)
Modifiable (generic)	Distance to work in kilometers (one way)
Modifiable (generic)	White meat (poultry)
Modifiable (generic)	Magnesium intake (mg/day)
Modifiable (generic)	Low fat milk (0.5%)
Modifiable (generic)	Tomato and cucumber
Modifiable (generic)	Pancake, waffle and Swedish dumpling
Modifiable (generic)	For how much of the time during the last four weeks have you felt gloomy and sad?
Modifiable (generic)	Salty fish
Modifiable (generic)	Eat breakfast from 2000
Modifiable (generic)	Fried potatoes and pommes frites
Modifiable (generic)	Sum of phytosterols intake (mg/day)
Modifiable (generic)	Syringaresinol intake (ug/day)
Modifiable (generic)	Self rate of overall health compared to a year ago
Modifiable (generic)	Sour milk, yoghurt (3% fat)
Modifiable (generic)	Possibility to speak with colleagues during breaks
Modifiable (generic)	Sucrose intake (g/day)
Modifiable (generic)	Alcohol intake (g/day)
Modifiable (specific)	Frequency of hunting or fishing during leisure time
Modifiable (generic)	Margarine for cooking
Modifiable (generic)	For how much of the time during the last four weeks have you felt happy?

Modifiable (generic)	Vitamin B6 intake (mg/day)
Modifiable (generic)	Sausage, liver pate on bread
Modifiable (generic)	Travel to work: Walk to work vs passive travel to work
Modifiable (generic)	Apple, pear, peach, orange, mandarin and grapefruit
Modifiable (generic)	Eicosapentaenoic acid (EPA) intake (g/day)
Modifiable (specific)	Years using snuff
Modifiable (generic)	Average portion size of vegetables based on photographic illustration of four sizes (smallest to largest)
Modifiable (generic)	Docosahexaenoic acid (DHA) intake (g/day)
Modifiable (generic)	Pizza
Modifiable (specific)	Boiled coffee
Modifiable (generic)	Light and physically active work
Modifiable (generic)	For how much of the time during the last four weeks have you felt so depressed that nothing could cheer you up?
Modifiable (generic)	Marital status: Single vs Divorced/separated
Modifiable (generic)	Berries (fresh or frozen)
Modifiable (generic)	Beta-sitostanol intake (mg/day)
Modifiable (generic)	Boiled or baked potato
Modifiable (generic)	Number of cigarretes smoked per day
Modifiable (generic)	Breakfast habits: Coffee/tea and wheat buns or rusk for breakfast vs not breakfast at all
Modifiable (generic)	Sedentary or standing work
Modifiable (generic)	Campestanol intake (mg/day)
Modifiable (generic)	Brewed (filtered) coffee
Modifiable (generic)	Job demands to work very fast

Modifiable (generic)	Medioresinol intake (ug/day)
Modifiable (generic)	Participation in study circles
Modifiable (generic)	Equol intake (ug/day)
Modifiable (generic)	Total energy intake (kcal/day)
Modifiable (generic)	Brown beans and pea soup
Modifiable (generic)	Patience status
Modifiable (generic)	Vitamin A intake (mg/day)
Modifiable (generic)	Work shifts/weekends
Modifiable (generic)	Liquor and spirits
Modifiable (generic)	Medium beer
Modifiable (generic)	Satisfaction with economy
Modifiable (generic)	Sugar, honey, marmelade and jam
Modifiable (generic)	For how much of the time during the last four weeks have you felt calm and serene?
Non-modifiable	Parents or siblings had a cerebral hemorrhage/thrombosis or cardiac infarction before the age of 60
Modifiable (generic)	Pentadecanoic acid intake (g/day)
Modifiable (generic)	Heptadecanoic acid intake (g/day)
Modifiable (generic)	Low fat margarine on bread
Modifiable (generic)	Zinc intake (mg/day)
Modifiable (generic)	Travel to work: Irregular travel mode to work vs passive travel to work
Modifiable (generic)	Pasta
Modifiable (generic)	Years smoking
Modifiable (generic)	Whole grain crisp bread

Modifiable (generic)	Mood status
Modifiable (generic)	Sleep status
Modifiable (specific)	Snuff status: Former snuff users vs non-snuff users
Modifiable (generic)	Milk, sour milk (3%)
Modifiable (generic)	Enterodiol intake (ug/day)
Modifiable (generic)	Polyunsaturated fat intake (g/day)
Modifiable (generic)	Saturated fat intake (g/day)
Modifiable (generic)	Receive hugs to comfort and support you
Modifiable (generic)	Frequent social contacts with colleagues during work
Modifiable (generic)	Hearing status
Modifiable (generic)	Whole grain soft bread
Modifiable (specific)	Frequency of gardening during leisure time
Modifiable (generic)	Milk, sour milk (1.5%)
Modifiable (generic)	Control over own work assignment
Modifiable (generic)	Number of cigarretes smoked per day (in groups)
Modifiable (generic)	Vitamin B2 intake (ug/day)
Modifiable (generic)	Satisfaction with work situation
Modifiable (generic)	Appetite status
Modifiable (generic)	Would you say that the number of people that you meet in your everyday life is enough or would you like to meet more or fewer people?
Modifiable (specific)	Coffee rolls/buns, rusk
Modifiable (generic)	Phosphate intake (mg/day)
Modifiable (generic)	Number of friends that can come to your home at any time and feel at home

Modifiable (generic)	Iodine intake (ug/day)
Modifiable (generic)	Cream, creme fraiche, sour cream
Modifiable (generic)	Disaccharides intake (g/day)
Modifiable (generic)	Teetotaler
Modifiable (generic)	Lean fish (e.g. perch, bass, cod)
Modifiable (generic)	Breakfast habits: Only coffee/tea for breakfast vs not breakfast at all
Modifiable (generic)	Light beer
Modifiable (generic)	Support from others
Modifiable (generic)	Light but partly physically active work
Modifiable (generic)	Repetitive job
Modifiable (generic)	Smoked fish/meat
Modifiable (generic)	Fatty fish (e.g. herring, whitefish, salmon)
Modifiable (generic)	Grams of tobacco smoked per week
Modifiable (specific)	Snuff status: Snuff users vs non-snuff users
Modifiable (generic)	Smoking status: Smokers vs non-smokers
Modifiable (generic)	Rosehip, sweet syrup soup
Modifiable (generic)	Do you feel important and appreciated in your home?
Modifiable (generic)	Control over planning and execution of the workday
Modifiable (generic)	Number of social contacts with the same interests as you
Modifiable (generic)	For how much of the time during the last four weeks have you felt very nervous?
Modifiable (generic)	Cohabitation: Live alone vs Only children
Modifiable (generic)	Sometimes physically straining work

Modifiable (generic)	Cheese 28%
Modifiable (generic)	White (soft) bread, thin crisp bread
Modifiable (generic)	Skill demand from job
Modifiable (generic)	Cookies and pastry
Modifiable (generic)	Smoking status: Former occasiol smokers vs non-smokers
Modifiable (generic)	Sodas, soft drinks and juice
Modifiable (generic)	Ice cream
Modifiable (generic)	Enough time for job assignments
Modifiable (generic)	Participation in associations or voluntary organisations
Modifiable (generic)	Number of social interactions during a normal week
Modifiable (generic)	Calcium intake (mg/day)
Modifiable (generic)	Linolenic acid intake (g/day)
Modifiable (generic)	High mental demand from job
Modifiable (generic)	Confidence status
Modifiable (generic)	Trans fat intake (g/day)
Modifiable (generic)	Sweets
Modifiable (generic)	Frequency of social contacts with colleagues during leisure time
Modifiable (generic)	Contradictory demands in job
Modifiable (generic)	Satisfaction with accomodation
Modifiable (generic)	Do you feel important and appreciated outside your home?
Modifiable (specific)	Bregott on bread
Modifiable (generic)	Vitamin E intake (mg/day)

Modifiable (specific)	Frequency of shoveling snow during leisure time
Modifiable (generic)	Feel uneasy or guilty because of your way of drinking
Modifiable (generic)	Marital status: Single vs Widow/widower
Modifiable (generic)	Iron intake (mg/day)
Modifiable (generic)	Last time a colleague visited you at home
Modifiable (generic)	Possibility to leave your work for a while to speak with a colleague
Modifiable (generic)	Butter on bread
Modifiable (generic)	Marital status: Single vs Married/partner
Modifiable (generic)	Oil for cooking
Modifiable (generic)	Strong beer
Modifiable (generic)	Cohabitation: Live alone vs Other/others
Modifiable (generic)	Memory status
Modifiable (generic)	Formic acid intake (g/day)
Modifiable (generic)	Meat on bread
Modifiable (generic)	Corn flakes
Modifiable (generic)	Sour milk, yoghurt (low fat)
Modifiable (generic)	Number of people with whom you can speak openly
Modifiable (generic)	Satisfaction with home and family situation
Modifiable (generic)	Campesterol intake (mg/day)
Modifiable (generic)	Potato salad
Modifiable (generic)	Learn new things at job
Modifiable (generic)	Feel the need to reduce alcohol consumption
Modifiable (generic)	Cheese 10-17%

Modifiable (generic)	High physical demand from job
Modifiable (generic)	Enterolactone intake (ug/day)
Modifiable (generic)	Butter for cooking
Modifiable (generic)	People to ask for help apart from the ones at home
Modifiable (generic)	Rice
Modifiable (generic)	Self-employed
Modifiable (generic)	Ingenuity or creativity demand from job
Modifiable (generic)	Linoleic acid intake (g/day)
Modifiable (generic)	Tiamin intake (mg/day)
Modifiable (specific)	Number of snuff boxes per week
Modifiable (generic)	Number of cigars smoked per day
Modifiable (generic)	Close relationship with anyone
Modifiable (generic)	Cohabitation: Live alone vs Adult and children
Modifiable (generic)	Frequency of engaging in clubs, associations or study circles
Modifiable (generic)	Chips, popcorn and salted nuts
Modifiable (generic)	Potassium intake (mg/day)
Modifiable (generic)	Margarine on bread
Modifiable (generic)	Cohabitation: Live alone vs Only one adult (spouse, partner)
Modifiable (generic)	Vision status
Modifiable (generic)	Time spent in a week in moderately steneous activities
Modifiable (generic)	Amount of exercise during the last 12 months
Modifiable (generic)	Soft cheese
Modifiable (generic)	Soft whey cheese
Modifiable (generic)	Mixed frozen vegetables
Modifiable (generic)	Mashed potato
Modifiable (specific)	Blota (broth + bread)
Modifiable (specific)	Blood based food
Modifiable (specific)	Liver and kidney
Modifiable (generic)	Shellfish (e.g. shrimps, scallops)
Modifiable (generic)	Frequency of alcohol consumption
Modifiable (generic)	Amount of alcohol drunk in a day
Modifiable (generic)	Frequency of drinking six or more glasses at the same occasion
Modifiable (generic)	Times during last year that you felt guilty because of your drinking
Modifiable (generic)	Risk of sleeping while sitting and reading
Modifiable (generic)	Risk of sleeping while watching TV
Modifiable (generic)	Risk of sleeping while sitting ictive in a public place
Modifiable (generic)	Risk of sleeping as a passenger in a car for one hour without break
Modifiable (generic)	Risk of sleeping while lying down resting in the afternoon
Modifiable (generic)	Risk of sleeping while sitting still after having lunch
Modifiable (generic)	Snore during sleep
Modifiable (generic)	Breath-holds during sleep
